# A Reliable Strategy to Unravel the Structure and Composition of Semiconducting Conjugated Donor‐Acceptor Block Copolymers

**DOI:** 10.1002/advs.202522268

**Published:** 2026-03-19

**Authors:** Antoine Curé, Pierre‐Alain Bayle, Lucie Rivet, Yann Kervella, Renaud Demadrille, Cyril Aumaître

**Affiliations:** ^1^ Univ. Grenoble Alpes CEA CNRS Grenoble INP IRIG, SyMMES Grenoble France; ^2^ Univ. Grenoble Alpes CEA IRGI MEM Grenoble France

**Keywords:** conjugated block copolymers, nuclear magnetic resonance, organic solar cells, single‐component materials

## Abstract

Single‐component (SC) polymers are a promising new generation of materials for organic photovoltaics. They offer the potential to combine high power conversion efficiencies with significantly improved stability compared to traditional heterojunction systems that incorporate non‐fullerene acceptors (NFAs). Despite this potential, the structural characterization of SC polymers is limited in the literature and is often insufficiently detailed. In this study, we present a rigorous and comprehensive strategy for elucidating the structure of these materials, which enables a deeper understanding of their architecture and structure‐property relationships. We describe a characterization protocol for a model SC polymer based on the donor polymer PTQ10 and an NFA‐based polymer PIDTe. Using a model small molecule approach, we identify the characteristic heterojunctions’ proton NMR signals and confirm their formation within the materials with the help of various 2D NMR methods. For the first time, we quantify the average number of heterojunctions (NoH) in the copolymers. We synthesize two additional SC polymers and demonstrate that our method can be applied to these systems, with the potential to be applied to others. This study establishes a reliable strategy for the accurate characterization of SC polymers intended for use in organic electronics and organic photovoltaics applications. A. C carried out the synthesis and performed the characterizations and analyzed the raw data. P‐A. B developed the NMR pulse sequences and optimized the NMR protocols. L. R and Y. K contributed to the synthesis. C. A acquired the funding and supervised the study. A. C wrote the manuscript, R. D and C. A corrected and edited the manuscript and all the authors reviewed the manuscript and have given their approval to the final version.

## Graphical abstract

This work proposes a reliable strategy to elucidate the structure and composition of conjugated donor‐acceptor block copolymers. The method, based on NMR analysis, allows to extract key structural information such as the number of heterojunctions (NoH) and the number of blocks (NoB) of the copolymers. The broad applicability of the method is demonstrated by its successful use with several materials.

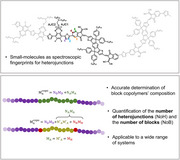

## 1 Introduction

The development of organic solar cells (OSCs) based on bulk heterojunction (BHJ) active layers has accelerated significantly with the advent of non‐fullerene acceptors (NFAs). These NFAs often incorporate central cores such as indacenodithiophene (A‐D‐A type, e.g., IDIC [1], IEICO‐4F [2], and O‐IDTBR [3]), indacenodithienothiophene (A‐D‐A type, e.g., ITIC [4], IT‐4F [5], and IT‐M [6]), and dithienothiophen[3,2‐b]pyrrolobenzothiadiazole (A‐D‐A’‐D‐A type, e.g., Y6 [7], L8‐BO [8], and BTP‐eC9 [9]), paired with 2‐(2,3‐dihydro‐3‐oxo‐1H‐inden‐1‐ylidene) (IC) flanking groups derivatives. Through advanced device engineering, OSCs have achieved a remarkable power conversion efficiency of 21% at the cell level [10, 11] and 18% on large‐area modules [12]. Despite the many advantages of OSCs, such as lightweight, low manufacturing costs, energy payback time [13, 14] and high efficiency in low light conditions [15, 16, 17], the poor morphological stability of the active layer remains a significant barrier to commercialization [18, 19, 20]. The thermal stability and performance of organic active layers are particularly influenced by the chemical compatibility between the donor (D) and acceptor (A) materials, as this dictates the extent and nature of phase separation during thermal annealing [21, 22, 23]. The Ade group showed that the intrinsic diffusion coefficient of matter within the active layer is directly related to the Flory‐Huggins parameter (χ) of the D/A pair, which in turn is a function of the glass transition temperature (Tg) of the acceptor and the elastic modulus (Ea) of the donor [24]. A strong D/A affinity (low χ) results in a stable but overmixed active layer, while a low affinity (high χ) usually results in adequate phase separation but low thermodynamic stability [21, 25, 26]. However, Ade also demonstrated that a very high χ value (typically χ > 5) enables the active layer to be stabilized kinetically [24, 27]. Therefore, introducing all‐polymer organic solar cells to stabilize the active layer morphology is a sensible approach. Since oligomerized/polymerized NFAs have significantly higher Tg than their monomeric counterparts [19, 28, 29], their diffusion coefficient is considered negligible compared to the average cell lifetime [30]. However, there are several challenges associated with all‐polymer solar cells, including suboptimal phase separation and unfavorable interactions between different polymer chains, particularly in polydisperse materials [30]. On the other hand, it has been shown that polymers with a dual D/A function, named single‐component (SC) materials, could provide superior stability with appropriate phase separation [31, 32]. Double‐cable SC polymers containing a D/A repeating unit, as well as block copolymers, have been proposed [33, 34, 35]. Block copolymers are generally easier to synthesize and have so far shown superior efficiency compared to double‐cable polymers. Also, Zheng et al. demonstrated that SC block copolymers with intramolecular heterojunctions could enhance charge transport and extraction and exciton dissociation, while reducing recombination, compared to BHJ solar cells [36].

Although these materials have regained attention with the rise of NFAs, their development has rapidly stagnated. This stagnation is largely attributed to their structural complexity and lacks of accurate characterization which makes the study of their structure–property relationship difficult. In addition to the number‐average (Mn) and weight‐average (Mw) molecular weights and the dispersity index (Đ), which are readily determined by size‐exclusion chromatography (SEC) or gel permeation chromatography (GPC), The comprehensive characterization of SC block copolymers also requires the quantification of additional parameters, such as the average number of blocks (NoB) and the average size of each donor and acceptor block. These parameters are rarely, if ever, specifically investigated.

These materials are typically synthesized via one‐pot or two‐pot Stille polymerizations, yielding statistical multiblock copolymers with variable properties [34, 37]. In the one‐pot method, the first polymer block is synthesized, after which the precursor for the second block is added to continue the polymerization in the same vessel. In the two‐pot method, the two polymer blocks are synthesized separately before being coupled in a subsequent step. While Cheng et al. demonstrated that the two‐pot method can produce materials with superior photovoltaic properties [37], recent studies suggest the opposite [34, 38]. Nevertheless, the number of unknown and uncontrolled parameters is too large to allow solid conclusions to be drawn. Notably, the average block number and the size of each block are likely to vary significantly depending on the reaction conditions, resulting in unpredictable differences in the thermal and electronic performance of the resulting materials. In fact, in SC systems, the intramolecular heterojunctions offer an additional pathway for charge transport and exciton dissociation, making the average number of heterojunctions (NoH) a critical factor for optoelectronic performance [36, 39]. In practice, however, this parameter is difficult to measure and has always been left unreported. More critically, there is currently no definitive evidence confirming that donor and acceptor blocks are covalently linked during synthesis. Verifying the formation of a carbon–carbon bond between blocks remains particularly challenging. This is mainly due to the inherent difficulties in acquiring high‐quality NMR spectra of conjugated polymers, stemming from their poor solubility in conventional deuterated solvents at room temperature, strong aggregation tendencies, and the long acquisition times required to achieve an adequate signal‐to‐noise ratio which deteriorate the quality of the spectrum [40, 41]. The issue of SC copolymer characterization was recently addressed by Theunissen et al., who noted that basic techniques such as ^1^H NMR have so far failed to resolve the structural complexity of these materials [41].

**FIGURE 1:**
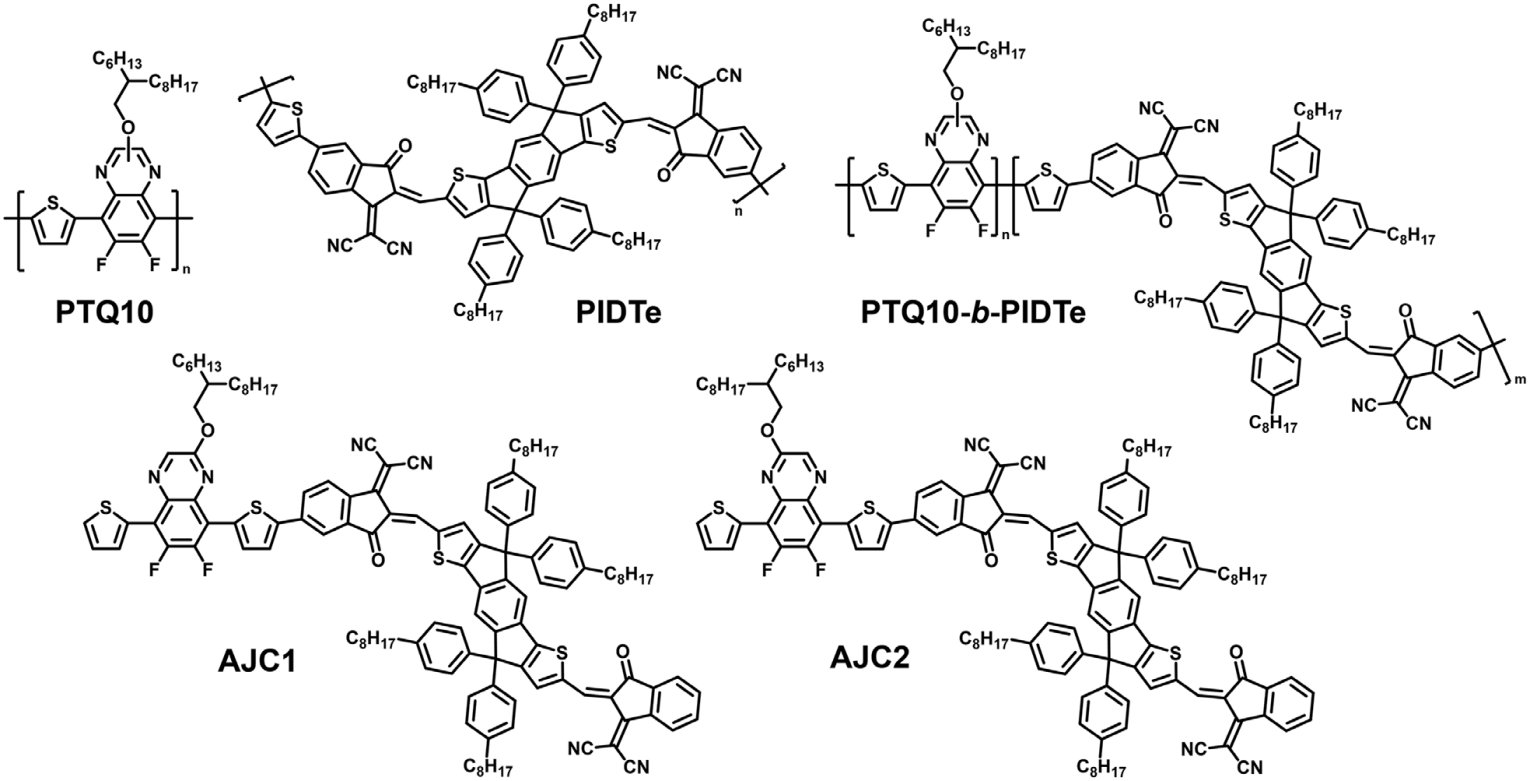
Chemical structure of the molecules studied in this work.

Here, we developed a model conjugated SC block copolymer based on the PTQ10/PIDTe system (Figure 1) which we fully characterized using a combination of 1D and 2D NMR techniques. PTQ10 is a well‐established *p*‐type polymer that is widely used in high‐efficiency organic photovoltaic applications [42, 43], while PIDTe is an *n*‐type polymer comprising repeating units with an IDT central core, *n*‐phenyloctyl side chains, IC acceptor flanking groups and a thiophene comonomer unit [44]. We synthesized two model donor‐acceptor molecules called **AJC1** and **AJC2** which served as NMR fingerprints for the signals of the protons located at the heterojunctions between the PTQ10 and the PIDTe blocks. Through proper signal identification and integration, we successfully assessed the formation of the heterojunctions within the material determining the average number of heterojunctions in the copolymers for the first time. We synthesized two additional block copolymers comprising acceptor polymers with repeating units whose structure are similar to that of ITIC and L8‐BO and proved that the heterojunctions’ signals could also be identified in their ^1^H NMR spectra. Our study provides a comprehensive characterization method for several SC conjugated block copolymers based on PTQ10 as the polymer donor. These materials can be used as benchmark systems for establishing accurate structure‐property relationship with the goal to understand the complex behavior of this class of semiconductors.

## 2 Results and Discussions

### 2.1 Synthesis and Characterization of the Model Donor‐Acceptor Small Molecules AJC1 and AJC2

To unambiguously demonstrate that the two blocks are covalently linked within a donor‐acceptor copolymer backbone, it is essential to identify a distinct spectroscopic signature corresponding to the junction. To this end, we synthesized two model molecules **AJC1** and **AJC2** (Figure 1), to mimic the junctions that would be formed if SC copolymers were obtained. Each model molecule represents a possible heterojunction scenario that may occur during the formation of copolymer chains, the alkyl side chain of the quinoxaline moiety being either closest or furthest from the acceptor moiety (**AJC1** and **AJC2**, respectively). Taking into account the weak mesomeric effect of the oxygen atom in the side chain of the quinoxaline moiety, the NMR signals of the molecules might shift slightly, resulting in two different characteristic signals for the heterojunctions. **AJC1** and **AJC2** were synthesized following the synthetic route shown in Figures S1 and S2 respectively. Compound **2** was obtained via a standard Stille cross‐coupling reaction between compounds **1** and 2‐(tributylstannyl)thiophene, followed by subsequent bromination to desymmetrize the molecule. This reaction afforded compounds **3** and **3’** in acceptable yields, 35% and 19% respectively. However, due to the spatial separation between the alkyl side chain of the quinoxaline unit and the thiophenes’ protons, NMR analysis could not determine the exact nature of the products. To resolve this, single crystals of product **3** were grown by slow diffusion of methanol into a tetrahydrofuran solution, and their structure was analyzed by X‐ray diffraction (Figures S17 and S18). The results showed that the product obtained at higher yield indeed corresponds to compound **3** in which the side chain is spatially closer to the bromine atom. Due to the instability of compounds **4** and **4’** during the chromatographic purification on silica and alumina, the crude products were merely purified via selective precipitation followed by filtration through a short plug of neutral alumina to remove the excess of hexabutyldistannane. While compound **4** showed relative stability on the alumina, compound **4’** degraded rapidly upon purification. NMR analysis revealed that only 24% of the desired product was obtained, with the remainder corresponding to compound **2**, indicating the removal of the SnBu_3_ group from the thiophene. The compounds were subsequently used without further purification. Owing to the limited selectivity of the reaction, compound **6** was obtained in a modest yield of 21%. Additionally, the limited purity of compound **4** and **4’** contributed to the formation of various by‐products during the final steps, providing **AJC1** and **AJC2** in a 24% and 20% yield after careful purification respectively.

**FIGURE 2:**
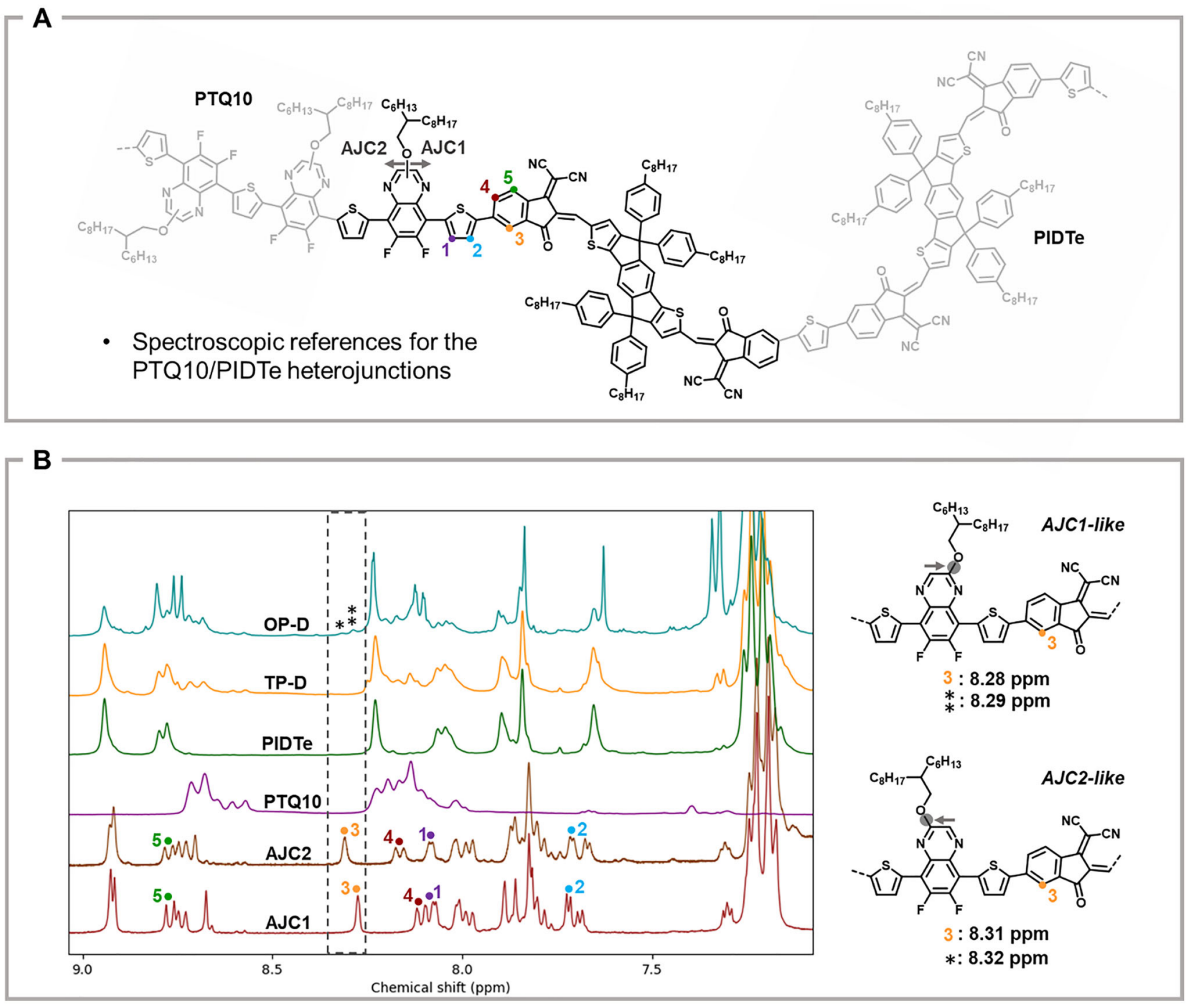
A. Chemical structures of AJC1 and AJC2 represented as PTQ10/PIDTe heterojunctions. B. Overlay NMR spectra of the block copolymers, homopolymers and small‐molecules and the expected correspondence of the NMR shifts.

As previously mentioned, the chemical structures of **AJC1** and **AJC2** mimic the polymer structure at the PTQ10 and PIDTe interface and are expected to provide insight into the ^1^H NMR shifts of the protons located at these heterojunctions. The total ^1^H NMR assignment of **AJC1** (Figure S45) and **AJC2** (Figure S61) and the other relevant small molecules was carried out using 1D, ^1^H and ^13^C NMR and 2D homonuclear correlation spectroscopy (COSY), heteronuclear single quantum coherence spectroscopy (HSQC) and heteronuclear multiple bond correlation (HMBC) analysis. Among the protons of the model molecules, only those closest to the D/A interface are expected to be similar in the final copolymers, these are numbered from **1** to **5** (Figure 2A). When the ^1^H NMR spectrum of **AJC1** is compared with those of the individual polymers PTQ10 and PIDTe, it is found that only the proton **3** signal at 8.28 ppm is sufficiently separated from the other peaks to allow its area to be quantified. This is because the ^1^H NMR spectra of PTQ10 and PIDTe obscure the other signals of **AJC1**. This suggests that the NMR signatures of intramolecular heterojunctions may appear in this region when PTQ10‐*b*‐PIDTe copolymer chains (Figure 1) are formed during synthesis. This singlet peak could then be easily identified in **AJC2**, with a reported difference of 0.03 ppm compared to the 8.28 ppm peak in **AJC1**. This result suggests that the PTQ10‐*b*‐PIDTe heterojunctions’ signatures should manifest as two singlet peaks appearing at approximately 8.28 and 8.31 ppm.

### 2.2 Synthesis of the SC Conjugated Polymers, Size‐Exclusion Chromatography (SEC) Measurements and UV–vis and NMR Spectroscopy

As previously mentioned, the two main approaches, namely the one‐pot and the two‐pot method, for preparing SC materials were compared. Currently, no studies clearly indicate which method is superior. This uncertainty is compounded by the lack of unambiguous spectroscopic evidence confirming junction formation, making it difficult to reliably assess the number of junctions present in the final materials. We therefore chose to compare both methods in our study, using the spectroscopic signature identified in **AJC1** and **AJC2** as a reliable and unambiguous marker to confirm the structure of our materials. The synthesis procedures for **TP‐D**, **TP‐C**, **OP‐D** and **OP‐C** are provided in Supporting Information. In a previous work, our group demonstrated that the catalyst/phosphine pair Pd_2_dba_3_/P(*o*‐tolyl)_3_ was a suitable system for the polymerization of NFAs [45]. We compared this system with the commonly used pair Pd_2_dba_3_/PPh_3_ for the polymerization of PTQ10. We found that using P(*o*‐tolyl)_3_ as the phosphine source significantly improved the reaction kinetics, yielding high molecular weight polymer chains within minutes as opposed to a few hours when PPh_3_ was used (Figure S3).

For the two‐pot method, a standard monomer concentration of 20 mm was used for each polymer. In our case, the PIDTe was hot injected directly into the PTQ10 active polymerization. The one‐pot method was carried out by polymerization of PTQ10 followed by hot injecting on the PIDTe monomers. All polymers were precipitated in methanol after synthesis and purified by Soxhlet extraction with methanol, acetone, ethyl acetate, dichloromethane and chloroform. For the two‐pot polymerization, two different fractions were obtained in dichloromethane and chloroform. As shown by its UV–vis spectrum, the chloroform fraction **TP‐C** consisted mainly of PTQ10 and was not further characterized (Figure S14). The dichloromethane fraction contains both PTQ10 and PIDTe blocks and is designated **TP‐D**. Two different fractions were also obtained in dichloromethane and chloroform for the one‐pot synthesis, designated **OP‐D** and **OP‐C** respectively. The average mass composition of the copolymers was obtained through SEC measurements in chloroform using 1 mg/mL solutions (Figure S8 and Table S3). **TP‐D** shows an Mn of 10 000 g/mol and a relatively large Đ of 3.7. Even though the material's molecular weights are centered around 30 000 g/mol, residual small molecular weight chains of 3 000 g/mol attributed to unreacted PIDTe dimers derivatives lower the average molecular weight and enlarge the Đ. The same conclusion can be drawn for **OP‐D**, which have a Mn of 5 300 g/mol and a Đ of 2.5. Its masses are centered around 10 000 g/mol but the overall molecular weight have a large contribution from the unreacted PIDTe dimers. Regarding **OP‐C**, the material has a Mn of 28 600 g/mol and a Đ of 3.1. Its mass distribution shows that the material is almost free from PIDTe unreacted dimers but contains a certain amount of lower molecular weight chain populations.

We then performed UV–vis spectroscopy to characterize the materials. (Figure S15). The UV–vis spectra of the materials clearly show contributions from both blocks: the PTQ10 donor block absorbs in the 400–650 nm range, while the PIDTe block contributes in the 650–800 nm range (Figure S12). Based on this, and the fact that chromatographic data which are often used as the primary evidence for SC formation, one might conclude that the SCs have indeed formed. However, these results alone do not provide definitive proof of a covalent junction between the two blocks. Additional NMR analysis is therefore essential to confirm the presence of the junction unambiguously. After synthesis the copolymers were also analyzed by NMR spectroscopy using the same conditions as before. Interestingly, the ^1^H spectrum of **TP‐D** does not show the presence of singlets peaks in the 8.28‐8.31 ppm range, which, at first sight, would indicate that the polymers may not have linked during the reaction. This suggests that **TP‐D** consists only of a mixture of separated PTQ10 and PIDTe chains, contrary to initial indications obtained by size exclusion chromatography and UV–vis spectroscopy. *This emphasizes the imperative need for NMR analysis to fully confirm the formation of single‐component materials*. On the other hand, two singlet peaks can be observed in the ^1^H NMR spectra of **OP‐D** and **OP‐C** at 8.29 ppm (^*^) and 8.32 ppm (^**^) respectively. The proximity of these peaks with the signals corresponding to proton **3** (8.28 and 8.31 ppm) in **AJC1** and **AJC2** suggests that some of the polymer chains may have copolymerized in this case (Figure 2B). This result is particularly important, as the UV–vis spectra and SEC distribution measurements provide inconclusive evidence as to the nature of the materials. Notably, **OP‐D** and **TP‐D** have similar UV–vis spectra, despite one probably being a copolymer and the other not.

### 2.3 Further ^1^H NMR Analysis of TP‐D, OP‐D and OP‐C

**FIGURE 3:**
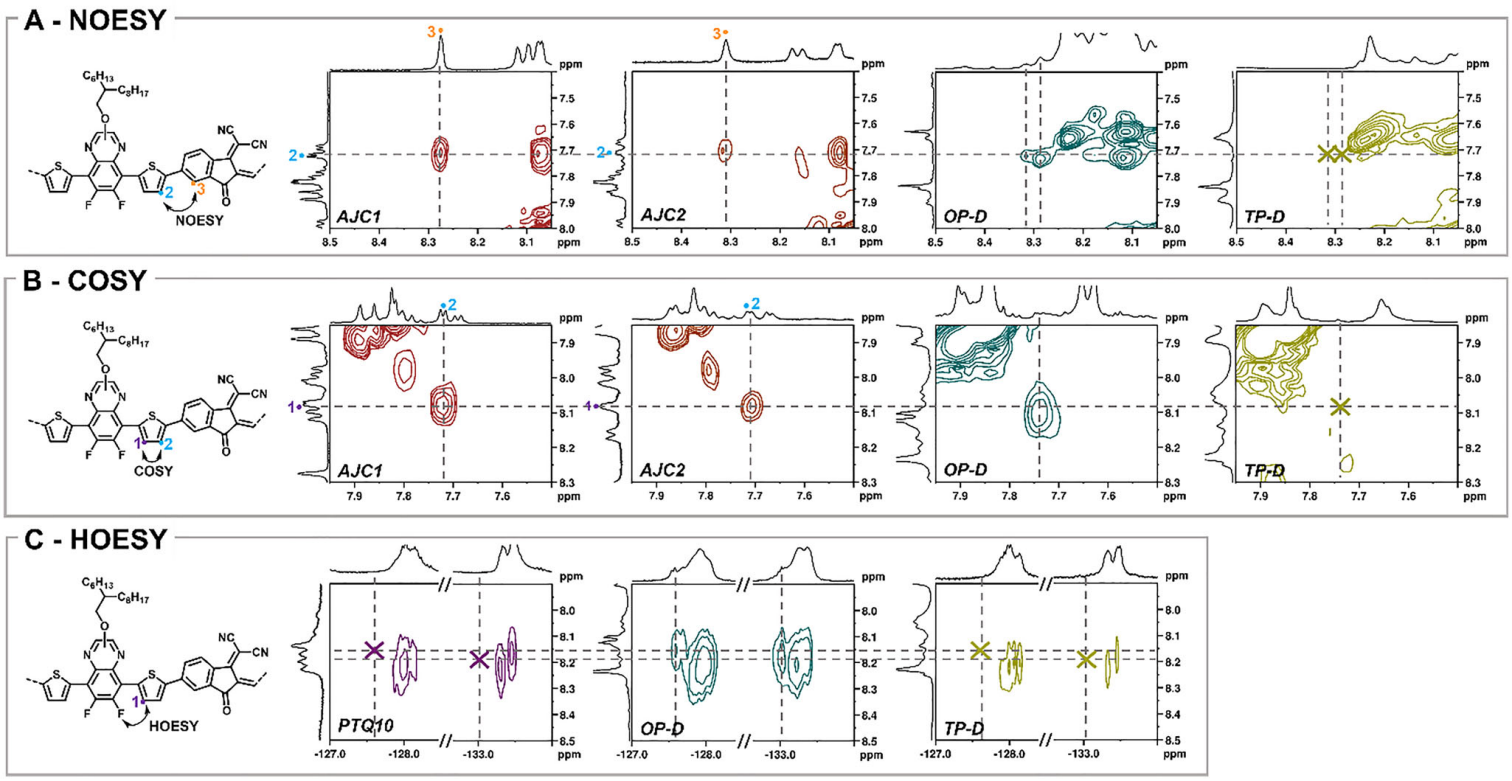
Comparison of A. The NOESY correlations showing the IC↔thiophene sequence in the materials. B. The COSY correlations showing the presence of a thiophene unit with an asymmetrical environment in the materials. C. The HOESY correlations showing the thiophene↔quinoxaline sequence in the materials.

To gain further insight, a COSY analysis was performed on **TP‐D**, **OP‐D** and **OP‐C** (Figures S90–S114). For both **OP‐D** and **OP‐C**, all the correlation spots of the protons of interest (**1↔2**, **4↔5** and **3↔4**) of **AJC1** and **AJC2** can also be observed in their COSY spectrum (Figure 3B; Figures S122 and S123). Since most of the hydrogen atoms attached to the thiophene comonomer units in the homopolymers sections have a symmetrical environment, their chemical shifts are similar and their COSY correlation spot is on the diagonal line. However, in **AJC1** and **AJC2**, the central thiophene unit has an asymmetric environment. This results in different chemical shifts for its hydrogen atoms (protons **1** and **2**) as well as a characteristic off‐diagonal COSY correlation spot. This correlation is evident in **OP‐D** and **OP‐C** with very good agreement (coordinates are [8.10:7.74] ppm versus [8:08:7.72] ppm in **AJC1** and [8.08:7.71] ppm in **AJC2**), which suggests the presence of a thiophene located between a quinoxaline and an IC unit (Figure 3B). As the **3↔4** COSY correlation signal appears to be also present for both the 8.29 and 8.32 ppm peaks but with a low intensity and resolution, further analysis is required to ensure that these signals are indeed equivalent to hydrogen **3** in **AJC1** and **AJC2** (Figure S122). Oppositely, even though the **4↔5** correlation can be observed, the **1↔2** and **3↔4** correlations are absent from the COSY spectrum of **TP‐D**, reinforcing the hypothesis that the materials consist only of a mixture of single homopolymer chains (Figure 3B; Figures S122 and S123).

Using nuclear Overhauser effect spectroscopy (NOESY), we found a clear correlation between protons **2** and **3** in **AJC1** and **AJC2**, indicating the close spatial proximity of the two protons in the molecules. The same correlation spot was also evident for both the 8.29 and 8.32 ppm peaks in **OP‐D** at almost exactly the same chemical shifts as in **AJC1**’s and **AJC2**’s spectra. The correlation was also verified to be absent in **TP‐D** (Figure 3A). This result provides strong evidence in favor of a structural link between these singlet peaks and the signal of the previous COSY correlation spot at 7.74 ppm, which we assign to a thiophene moiety. To confirm that the copolymers’ singlet peaks arise from hydrogen atoms belonging to an IC unit, HSQC and HMBC experiments were conducted on **OP‐D** (Figures S104–S107). HSQC analysis shows that the two singlet peaks appear to correspond to similar carbons, with a chemical shift identical to that of the carbon bearing proton **3** in **AJC1** and **AJC2** (Figure S124). Moreover, HMBC analysis shows that the 8.29 ppm singlet peak has a similar correlation pattern to proton **3**, again with very close chemical shifts (Figure S125). This indicates that this specific peak most probably belongs to an IC unit. Regarding the 8.32 ppm peak, its intensity is low and we could not verify its HMBC pattern with certainty. However, considering that the previous correlations were also found for this peak, in particular the very close NOESY correlation spot, we assume both the 8.29 ppm and the 8.32 ppm signals are of the same nature. With this assumption, the 8.29 ppm peak could be attributed to the **AJC1**‐like heterojunction signal where the side chains of the quinoxaline moiety are furthest from the acceptor while the 8.29 ppm singlet belongs to a heterojunction with a structure identical to **AJC2**. To consolidate this hypothesis, DFT calculations of **AJC1** and **AJC2** were performed. Their geometry was optimized at the PBE D3BJ/def2‐svp level of theory (Figure S5) and the B3LYP functional with the commonly used pcSseg‐2 basis set served to simulate their proton NMR spectrum [46]. To account for the influence of the solvent, the NMR shifts were also calculated in chloroform, as tetrachloroethane was not available for simulation. As shown in the results in Table S1. The NMR shifts predicted by the pcSseg‐2 basis set with and without chloroform are close to the experimental values, 8.45 and 8.48 ppm versus 8.29 ppm for **AJC1** and 8.47 ppm and 8.51 ppm versus 8.32 ppm for **AJC2**. In addition, the pcSseg‐2 basis set and the pcSseg‐2 basis set in chloroform predict that the signals should have a difference of respectively 0.02 ppm and 0.03 ppm. These results are in line with the experimental observations.

Finally, to unambiguously confirm the nature of the signals, we used ^1^H‐^19^F heteronuclear Overhauser effect spectroscopy (HOESY) to verify that the 8.10 ppm signal of the copolymer is correlated to a fluorine atom. Since only the quinoxaline units carry fluorine atoms and only the acceptor polymers contain IC units, the identification of the **IC↔thiophene↔quinoxaline** sequence would provide a definitive argument for the connection of the polymer chains during the reaction. This would confirm that the singlet peaks observed in **OP‐D** and **OP‐C** at 8.29 and 8.32 ppm, respectively, are indeed the characteristic signals of protons located at the heterojunction between a PIDTe and a PTQ10 repeating unit. For **AJC1** and **AJC2**, we could only acquire signal at 25°C (Figures S58–S73). This shows that this correlation can be monitored but prevents the use of the small molecules as references. Nevertheless, the NMR shifts of the fluorine atom located the closest to proton **1** in **AJC1** and **AJC2** are −127.8 and −133.2 ppm respectively, which corresponds to an expected difference of 5.4 ppm. The HOESY spectrum of **OP‐D** is reported in Figure S108. Interestingly, two correlation signals with respective coordinates [8.14, −127.6] ppm and [8.14, −133.0] ppm can be observed on the spectrum. These two distinct correlations have a chemical shift difference of 5.4 ppm and appear to correspond to two sharp signals emerging from the shoulder of the copolymer's broad fluoride signals. The DFT prediction of the ^19^F NMR shifts of the fluoride atom located closest to the heterojunction for **AJC1** and **AJC2** using different basis sets is reported in Table S2. Interestingly, the 6–31+G(d,p) basis set, recommended for the modeling of fluorinated aromatic compounds [47], provides a chemical shift difference between **AJC1** and **AJC2** of 5.5 ppm in the gas phase and 4.7 ppm in chloroform, again supporting the experimental value of 5.4 ppm observed in **OP‐D**. Unfortunately, the positions of the correlation spots are not very accurate along the ^1^H axis, as the spots are quite spread out. However, considering these correlations are absent from the ^1^H‐^19^F HOESY spectra of pure PTQ10 and **TP‐D** (Figure 3C), it is likely that they correspond to the two characteristic signatures of the heterojunctions. Ultimately, this set of NMR experiments and DFT calculations provides almost irrefutable evidence that the two NMR signals identified at 8.29 and 8.32 ppm originate from PTQ10/PIDTe heterojunctions. Also, given the absence of heterojunctions in **TP‐D**, our study demonstrates that chain linkage during copolymerization cannot be taken for granted and that the connection between polymer chains must be systematically verified in each SC copolymer report. To eliminate the possibility of operator‐dependent errors, the **TP‐D** synthesis was repeated using the same protocol. The only difference was the phosphine used to prepare the PIDTe polymer precursor, PPh_3_ instead of P(*o*‐tolyl)_3_. This substitution was expected to produce shorter polymer chains that may exhibit higher reactivity [45]. The resulting materials exhibited comparable macromolecular parameters to **TP‐D** (Table S3 and Figure S8). However, as with **TP‐D**, the ^1^H NMR spectrum of the material did not exhibit the two characteristic heterojunction signals (Figures S86 and S87). This demonstrates that conjugated block copolymers may not form under certain synthesis conditions adapted from literature [34, 38].

### 2.4 Quantification of the D/A Molar Ratio and the Average Number of Intramolecular Heterojunctions (NoH)

In most cases, the determination of the D/A molar ratio of a copolymer or a polymer blend, which, by definition, is the ratio between the number of donor (N_D_) and acceptor (N_A_) repeating units, is straightforward. This information can be obtained through elemental analysis [37, 48], XPS [31, 49] or quantitative NMR for instance [50, 51]. In our case, the D/A molar ratio can be determined by NMR, owing to the presence of NMR signals that correspond solely to PTQ10 or PIDTe and that can be easily identified and integrated. For PTQ10, the signal corresponding to the –CH_2_– group (around 4.7 ppm) linked to the oxygen atom of the alkyl side chain is chosen. For PIDTe the signal corresponding to the –CH_2_– group (around 2.7 ppm) attached to the benzene ring of the alkyl side chain is used. Where the Mn of the copolymer is known, this data can be used to estimate the average size of each polymer block in the material. Regarding the heterojunction signals, integration without a well‐defined baseline would probably lead to large discrepancies. Therefore, signal deconvolution was used, using the embedded “dcon” module of TopSpin 4.4.1. The selected signals for PTQ10, PIDTe and the heterojunctions were expressed as a sum of Lorentzian functions, the size of which were adjusted to achieve accurate and comparable integrations with the best fit. Guidelines for proper signal deconvolution are provided in Supplementary Note S1.

**FIGURE 4:**
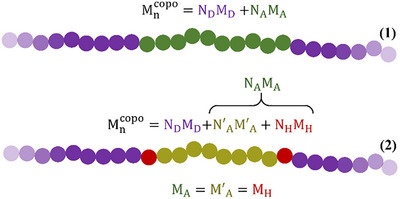
Illustration of the copolymer's decomposition into its individual repeating units and the equations used for the NoH calculation.

Figure S126 and Table S4 provide an illustration of signal deconvolution and the area determined by this method. The D/A molar ratios obtained through deconvolution are comparable to those obtained with standard integration (Table S5). The most relevant results obtained in this section are summarized in Table 1. For the synthesis of the copolymers a 1/0.48 D/A molar ratio (corresponding to a standard 1/1.5 D/A mass ratio) of monomers was used. The ratio of 1/0.76 determined for **TP‐D** is in line with the previous observation that a significant fraction of the PTQ10 was retrieved in the chloroform fraction of the Soxhlet purification. For a copolymer, this value corresponds to 6.3 repeating units of PTQ10 and 4.7 repeating units of PIDTe in average. Regarding **OP‐D** and **OP‐C**, while a large part of the PIDTe is incorporated in **OP‐D**, (D/A molar ratio of 1/1.06), **OP‐C** have a majority of PTQ10 with a ratio of 1/0.20. On average, this corresponds to 36 repeating unit of PTQ10 and 7.3 of PIDTe, while **OP‐D** has almost the same number of repeating units of both polymers (2.5 for PTQ10 and 2.7 for PIDTe). *However, because the number of blocks (NoB) of the copolymers is unknown, the average size of each block calculated by NMR is inaccurate*. To determine the NoB of the copolymers, the areas below the heterojunction protons’ signal are quantified to extract the value of the NoH (by definition, NoH = NoB‐1). In accordance with the previous notations, these areas are denoted A_*_ and A_**_ respectively, with A_*_ +  A_**_ = A_H_ , the total area of heterojunction signals. By equivalence, we have N_*_ +  N_**_ = N_H_ , the total number of heterojunctions repeating units. In the discussion, N_H_ will be referred as NoH. This value provides information about the average number of repeating units of PIDTe involved in heterojunctions. As the chemical shift of the –CH_2_– group used for PIDTe in the calculation of the D/A ratio is not expected to change significantly, even when the molecule is located at the heterojunction, the integral (A_A_) also accounts for the contribution of the heterojunctions A_H_ (see Figure 4). Therefore, once the areas of the heterojunction signals have been determined, they must be subtracted from the area of PIDTe to yield the corrected value A′_A_ = A_A_  − A_H_, which corresponds to the acceptor repeating units that are not located at a heterojunction. The NoH (or N_H_) of the polymers were calculated using Equation 1 and Equation 2, as shown in Figure 4, by decomposing the copolymer's molecular weight into the sum of the molecular weights of its individual repeating units. The number of “heterojunction” repeating units (N_H_) was determined using the ratios with the number of donor repeating units (N_D_) and acceptor repeating units not located at a heterojunction (N′_A_), under the assumption that N_H_/ N_D_ = A_H_ /A_D_ and N_H_/ N′_A_ = A_H_ /A′_A_ (Table S5). An example of the NoH calculation is provided for **OP‐C** in Supplementary Note S2.

**TABLE 1:** Composition parameters of the SC polymers determined by SEC (Mn, Mw and Đ) and ^1^H NMR (N_D_/N_A_, NoH, NoB, N_D_ and N_A_).

Material	Mn (kg/mol)	Mw (kg/mol)	Đ	N_D_/N_A_	NoH	NoB	N_D_	N_A_
TP‐D	10.2	36.4	3.6	1/0.76	—	—	—	—
OP‐D	5.3	13.1	2.5	1/1.06	**0.3**	**1.3**	2.5	2.7
OP‐C	28.6	87.1	3.1	1/0.20	**4.1**	**5.1**	14.2	2.9

The low NoH value of 0.3 determined for **OP‐D** can be explained by the probable contamination of the material by a large amount of unreacted homopolymers. Based on the signal deconvolution, we found that more heterojunctions were formed with the alkyl side chain of the quinoxaline unit located closest to the acceptor (**AJC1**‐like structure). In fact, two heterojunctions out of three in the material were formed in this way (N_**_/ N_*_ = 1/0.7). Regarding **OP‐C**, the material contains an average of 4.1 heterojunctions, indicating that it is approximately a molar mixture of 90% five‐block and 10% hexa‐block copolymers. Interestingly, the ratio of the two heterojunction isomers appears to differ among the various Soxhlet fractions of the same polymerization batch. Indeed, the calculated ratio of the two isomers in **OP‐C** is 1/0.4, meaning that three out of five heterojunctions were formed with an **AJC1**‐like structure.

This work in the first to provide a precise determination of the NoH (and the NoB) of a conjugated copolymer. This parameter is expected to be critical for the exciton splitting and charge extraction properties of the materials. Additionally, the NoB can effectively be used to recalculate the average length of each block in the copolymers. This is particularly interesting in the case of **OP‐C**, which is believed to consist almost entirely of copolymer chains. Initially, **OP‐C**’s polymer chains were found to comprise approximately 36 PTQ10 repeating units and 7.3 PIDTe repeating units. When divided into five blocks, the chains can be composed of either three PTQ10 blocks and two PIDTe blocks, or two PTQ10 blocks and three PIDTe blocks. Conversely, hexa‐blocks copolymers always contain three blocks of each component. With a determined NoB of 5.1, the average number of repeating units and the size of each individual polymer in **OP‐C** can be recalculated as 14.2 for N_D_ (PTQ10) and 2.9 for N_A_ (PIDTe). This corresponds to approximate molecular weights of 6.9 and 4.4 kg/mol, respectively. These relatively low molecular weights, recalculated for each block, suggest that a critical view of the materials is necessary. In fact, these results demonstrate that only oligomers of acceptors and short donor chains were incorporated in the material at the best. Also, since the NoH of **OP‐D** is below 1, the values for N_D_ and N_A_ remain unchanged. We hope that this result will raise awareness among the SC community about the true nature of these materials. Our research has shown that the linkage of molecules is not systematic during polymerization, resulting in materials that differ significantly in structure and composition from what is expected.

As deconvolution approximates the shape of NMR signals using Lorentzian functions with a certain degree of accuracy, a statistical analysis was performed to evaluate the uncertainty in the NoH and NoB values (Tables S8 and S9). This analysis shows that the NoH and NoB of donor‐acceptor conjugated block copolymers are determined with low uncertainty in the results. In fact, the uncertainty in the NoH values of **OP‐D** and **OP‐C** were found to be 7.1%, 4.8%, respectively.

### 2.5 Generalization of the Method to Other Materials

As such, **OP‐D** and **OP‐C** were merely used as proofs of concept to demonstrate that the proper characterization of conjugated block copolymers is achievable. Nevertheless, systems using acceptor polymers based on indacenodithiophene repeating units typically exhibit significantly lower efficiencies than active layers fabricated with acceptor polymers containing repeating units similar to ITIC or Y6 [52, 53, 54]. The latter materials currently represent the state of the art for A‐D‐A and A‐D‐A'‐D‐A acceptors, respectively. Demonstrating the generality of our methods to these materials would represent a significant milestone. We therefore synthesized two additional conjugated block copolymers with repeating units based on ITIC and L8‐BO, a derivative of Y6. These materials are referred as **PTQ10‐*b*‐PITIC** and **PTQ10‐*b*‐PL8‐BO** respectively (Figure S6). The copolymers were synthesized using the one‐pot method (see Supporting Information). For **PTQ10‐*b*‐PITIC**, we achieved a Mn of 17.1 kg/mol and a Đ of 3.7 while a relatively small Mn of 6.9 kg/mol and a Đ of 2.7 was obtained for **PTQ10‐*b*‐PL8‐BO** (Table S3 and Figure S9). The materials’ ^1^H and ^19^F NMR spectra were acquired using the same conditions as before (Figures S116–S121). The results of the NMR analysis are given in Table 2 and Table S6.

**TABLE 2:** Chemical shift of the heterojunctions’ signals and the NoH and NoB of PTQ10‐*b*‐PITIC and PTQ10‐*b*‐PL8‐BO.

Material	σ* (ppm)	σ** (ppm)	NoH	NoB
PTQ10‐*b*‐PITIC	8.31	8.28	2.1	3.1
PTQ10‐*b*‐PL8‐BO	8.33	8.30	0.1	1.1

**FIGURE 5:**
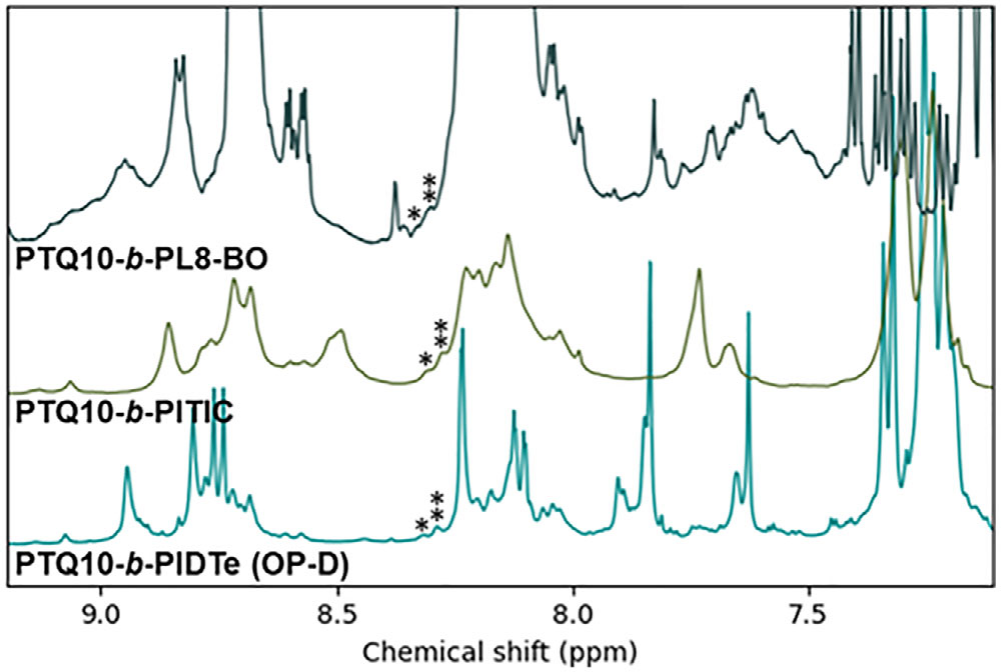
Identification of the heterojunctions’ peaks in PTQ10‐*b*‐PIDTe (OP‐D), PTQ10‐*b*‐PITIC and PTQ10‐*b*‐PL8‐BO.

Regarding **PTQ10‐*b*‐PITIC**, a rapid analysis of its ^1^H NMR spectrum shows that the two characteristic heterojunctions’ signals can be clearly identified in the material at almost exactly the same chemical shifts (8.28 ppm and 8.31 ppm) as in **OP‐D** and **OP‐C**. Similarly, the two signals are found in **PTQ10‐*b*‐PL8‐BO** with good agreements at 8.33 ppm and 8.30 ppm respectively (Figure 5).

Using the same deconvolution protocol as before, we determined the NoH and NoB of **PTQ10‐*b*‐PITIC** to be 2.1 and 3.1 respectively. Given the D/A molar ratio of 1/0.18 and the Mn of 17.1 kg/mol, this corresponds to a polymer having an average N_D_ of 14.2 and N_A_ of 2.6. Interestingly, these values are very close from the numbers obtained with **OP‐C**. The low NoH of 0.1 determined for **PTQ10‐*b*‐PL8‐BO** indicates the high content of homopolymers in the materials and shows once again that materials retrieved in the early fractions of the Soxhlet purification (such as dichloromethane) usually contains a low amount of copolymer chains. The statistical analysis performed to evaluate the uncertainty in the NoH and NoB values for **PTQ10‐*b*‐PITIC** led to an uncertainty of just 1.4%. By contrast, an uncertainty of around 22% was found for **PTQ10‐*b*‐PL8‐BO**, where the heterojunction signals are weaker (Tables S8 and S9).

This outstanding result demonstrates the broad applicability of our characterization method to block copolymers based on PTQ10 as the polymer donor. This work paves the way for more accurate and reliable structure‐property studies on this complex class of materials. Furthermore, we demonstrate that the model small molecule approach can successfully help to accurately characterize conjugated block copolymers.

## 3 Conclusion

In conclusion, we present a comprehensive methodology for fully characterizing single‐component conjugated copolymers. Using a reference system comprising PTQ10 as the donor and PIDTe as the acceptor, as well as various 1D and 2D NMR techniques and two model molecules named AJC1 and AJC2, we successfully identified and quantified two specific proton signals with each signal corresponding to a unique type of heterojunction between the donor and acceptor blocks. This analysis confirms the formation of a covalent bond between the two components during synthesis. It quantifies the average number of heterojunctions (NoH) and number of blocks (NoB) within the materials and determines the ratio between the two types, providing valuable insights into the intrinsic reactivity of the molecular components. Furthermore, recalculating the size of the donor and acceptor blocks provides the most detailed description ever achieved for this type of material. In addition, statistical analysis of the NoH and NoB calculations demonstrates that using signal deconvolution for the determination of the area of the heterojunction signals allows for low uncertainty on the results. Finally, by synthesizing two additional block copolymers based on state‐of‐the‐art acceptor polymers, we demonstrate that our method can also be applied to a wide range of systems. This study shows that the model small molecule approach can be a powerful strategy for the proper characterization of single‐component block copolymers. These insights are expected to facilitate the rational design of these complex semiconducting materials and lead to more efficient single‐component conjugated polymers for use in high‐performance organic solar cells.

[CCDC ###### contains the supplementary crystallographic data for this paper. These data can be obtained free of charge from The Cambridge Crystallographic Data Centre via www.ccdc.cam.ac.uk/data_request/cif.]

Material and polymer synthesis and characterization (structures, procedures, MS, UV–vis, SEC, 1D and 2D NMR). DFT calculations; crystal structure; NMR deconvolution analysis; number of heterojunctions and block length calculation method (PDF). Crystallographic data for the single‐crystals of compound 3 (CIF).

## Funding

A. C. acknowledges CEA for funding through a CFR PhD grant. The authors are grateful to Agence National de la Recherche (ANR) for the grant ANR‐22‐CE06‐0018, acronym: MONOPOLY.

## Conflicts of Interest

The authors declare no conflicts of interest.

## Supporting information




**Supporting File**: advs74635‐sup‐0001‐SuppMat.docx.

## Data Availability

The data that support the findings of this study are available in the supplementary material of this article.
