# Supplementary material for: A Reliable Strategy to Unravel the Structure and Composition of Semiconducting Conjugated Donor‐Acceptor Block Copolymers
Source: Adv Sci (Weinh). 2026 Mar 19;13(27):e22268. doi: 10.1002/advs.202522268 (PMC13170251; doi:10.1002/advs.202522268)
Supplement: Supplementary file 1 — Supporting File: advs74635‐sup‐0001‐SuppMat.docx. [file ADVS-13-e22268-s001.docx]

**A Reliable Strategy to Unravel the Structure and Composition of Semiconducting Conjugated Donor-Acceptor Block Copolymers**

Antoine Curé^a*^, Pierre-Alain Bayle^b^, Lucie Rivet^a^, Yann Kervella^a^, Renaud Demadrille^a^,

and Cyril Aumaître^a^*

^a^Univ. Grenoble Alpes, CNRS, CEA, SyMMES, 17 Avenue des Martyrs, 38000 Grenoble, France.

^b^Univ. Grenoble Alpes, CNRS, CEA, MEM, 17 Avenue des Martyrs, 38000 Grenoble, France.

**Supporting information**

Table of contents

[General methods 2](#_Toc221206781)

[Materials 2](#_Toc221206782)

[SEC measurements 2](#_Toc221206783)

[DFT calculations 2](#_Toc221206784)

[NMR Characterizations 2](#_Toc221206785)

[1D NMRs 2](#_Toc221206786)

[2D NMRs 3](#_Toc221206787)

[Synthesis of the materials 3](#_Toc221206788)

[Synthesis of AJC1 3](#_Toc221206789)

[Synthesis of AJC2 7](#_Toc221206790)

[Synthesis of the monomers 9](#_Toc221206791)

[Synthesis of the polymers 10](#_Toc221206792)

[Synthesis of the block copolymers 12](#_Toc221206793)

[DFT calculations 14](#_Toc221206794)

[Color code of the molecules 16](#_Toc221206795)

[SEC distribution of the materials and UV-Vis spectra 17](#_Toc221206796)

[Single-crystal growth of compound 3 22](#_Toc221206797)

[Protocol for single-crystal growth of compound 3 22](#_Toc221206798)

[Crystal structure of compound 3 22](#_Toc221206799)

[NMR spectra and total ^1^H attributions of the materials 23](#_Toc221206800)

[NMR correlations for the principal signals 74](#_Toc221206801)

[NMR signal deconvolution analysis 75](#_Toc221206802)

[Supplementary Note 1: guidelines for NMR signal deconvolution 75](#_Toc221206803)

[Supplementary Note 2: NoH calculation example for OP-C and block length recalibration 76](#_Toc221206804)

[Uncertainty calculations with TopSpin 78](#_Toc221206805)

[References 79](#_Toc221206806)

# General methods

## Materials

Products **1**^[1]^, **IC-Br**^[2]^ and **ITIC-2CHO**^[3]^ were synthesized according to published protocols. Product **5** was purchased from Ikamba Organics. 2-(3-oxo-2,3-dihydro-1H-inden-1-ylidene)malononitrile (IC) and L8-BO-2CHO were purchased from Brilliant Matters. Deuterated solvents (CDCl_3_ and C_2_D_2_Cl_4_) for NMR analysis were purchased from Eurisotop. All catalysts were bought from Strem Chemicals Inc. All other reagents were purchased from Sigma-Aldrich.

## SEC measurements

The SEC measurements were performed using an Agilent PLgel 5 µm MIXED-D column with chloroform as the mobile phase. The temperature was set to 30 °C, the flow rate to 1.0 mL/min and the pressure was allowed to stabilize between 77 bar and 80 bar prior to measurement. 30 µL of 0.45 µm PTFE filtered 1 mg/mL solutions in chloroform were injected for each measurement. The data treatment was performed using the Agilent SEC-OFFLINE software and the Agilent polystyrene calibration samples.

## DFT calculations

DFT calculations were performed for AJC1 and AJC2 using ORCA 5.0. The 3D geometries were optimized at the PBE D3BJ/def2-svp level of theory. The NMR shifts of the hydrogen atoms were calculated using the B3LYP functional and the pcSseg-2 basis set. The NMR shifts of the fluoride atoms were calculated using the B3LYP functional and the pcSseg-2, def2-tzvp, 6-31G and 6-31+G(d,p) basis sets. The NMR shift calculations were also performed using chloroform as solvent. For simplicity’s sake, the side chains were replaced by methyl groups, including the benzene side groups of the IDTe. For the calculation of the fluoride NMR shifts, a correlation determined by Saunders *et al.* for the 6-31G and 6-31+G(d,p) basis sets is used.^[4]^

## NMR Characterizations

The ^1^H, ^19^F and ^13^C 1D NMR spectra were acquired using a 400 MHz Bruker NMR spectrometer equipped with a 5 mm BBO-like probe (Smart probe). The HOESY 2D NMR spectra were acquired using the same 400 MHz Bruker NMR spectrometer. The COSY, HSQC, HMBC and NOESY 2D NMR spectra were acquired using a 500 MHz Bruker NMR spectrometer equipped with a 5 mm inverse BBI probe. All CDCl_3_ NMR spectra were acquired at a temperature of 298 K. All C_2_D_2_Cl_4_ spectra were acquired at a temperature of 393 K, except for the HOESY spectra of AJC1 and AJC2 that were performed at 298 K. For the reference spectrum of PTQ10 a 70/30 w%. mixture of a 3 kg/mol and a 40 kg/mol samples was used.

### 1D NMRs

The 1D ^1^H NMR spectra of AJC1, AJC2 and the polymers were acquired using a relaxation delay (D1) parameter) corresponding to 4.5-5 times the value of the relaxation time (T_1_) of the longer-relaxing proton (about 20 s) to ensure the recovery of 99% of the signal. The number of scans was adapted for each spectrum to ensure enough signal-to-noise ratio. For spectra acquired at high temperatures, sufficient time was allowed for the system (sample and spectrometer parts) to reach equilibrium prior to measurement. When possible, the multiplicity as well as the carbon-fluoride coupling constants of the ^13^C NMR signals are reported.

### 2D NMRs

For all the 2D experiments, and especially at 393 K, the 90° pulses were recalibrated to account for the variation in the RF circuit caused by the high temperature, thus optimizing the signal. For the HOESY experiments, the mixing time (D8 parameter) was set approximately as the relaxation time of the fluorine atoms (about 400 ms).

## Synthesis of the materials

### Synthesis of AJC1

Figure S1. Synthesis pathway of AJC1.

**Synthesis of 6,7-difluoro-2-((2-hexyldecyl)oxy)-5,8-di(thiophen-2-yl)quinoxaline (2).**

In a reaction flask, a solution of compound **1** (1000 mg, 1.77 mmol, 1 eq.), tributyl(thiophen-2-yl)stannane (1.4 mL, 4.43 mmol, 2.5 eq.) and Pd(PPh_3_)_4_ (82 mg, 0.07 mmol, 4 mol%) in dry degassed toluene (80 mL) was set to react at reflux under inert atmosphere. After 17 h, the reaction mixture was diluted in water, transferred to a separatory funnel and extracted with DCM. The organic layers were washed with water and brine, dried over Na_2_SO_4_ and evaporated under reduced pressure. The crude product was purified by flash column chromatography on silica (hexane:DCM 90:10 vol.%) to afford the desired product as a yellow oil (994 mg, 1.74 mmol, 98 % yield). ^1^H NMR (400 MHz, CDCl_3_, ppm): δ = 8.57 (s, 1H), 7.94 (m, 1H), 7.87 (m, 1H), 7.61 (m, *J* = 5.2 Hz, 1H), 7.59 (m, *J* = 5.2 Hz, 1H), 7.25 – 7.20 (m, 2H), 4.51 (d, *J* = 5.7 Hz, 2H), 1.92 (m, 1H), 1.53 – 1.14 (m, 26H), 1.00 – 0.73 (m, 7H). ^19^F NMR (400 MHz, CDCl_3_, ppm): δ = -129.20 (d, *J* = 19.3 Hz), -134.79 (d, *J* = 19.4 Hz). ^13^C NMR (400 MHz, CDCl_3_, ppm): δ = 157.1, 149.9 (dd, *J* = 256.0 Hz, 17.2 Hz), 147.4 (dd, *J* = 251.1 Hz, 17.5 Hz), 137.6 (d, *J* = 2.7 Hz), 134.6 (d, *J* = 7.6 Hz), 132.4 (d, *J* = 6.6 Hz), 130.9 (m), 130.3 (d, *J* = 14.6 Hz), 129.5 (d, *J* = 2.7 Hz), 128.8 (d, *J* = 2.6 Hz), 126.6 (d, *J* = 23.1 Hz), 118.9 (d, *J* = 10.0 Hz), 117.1 (d, *J* = 8.9 Hz), 71.2, 37.5, 31.9, 31.9, 31.4, 30.0, 29.7, 29.6, 29.3, 26.8, 26.8, 22.7, 14.1. HRMS (ESI) calcd for C_32_H_40_F_2_N_2_OS_2_ [M^+.^]: 570.25446; found: 570.2542.

**Synthesis of 8-(5-bromothiophen-2-yl)-6,7-difluoro-2-((2-hexyldecyl)oxy)-5-(thiophen-2-yl)quinoxaline (3).**

To a solution of compound **2** (995 mg, 1.74 mmol, 1 eq.) in CHCl_3_ (100 mL), a solution of N‑bromosuccinimide (310 mg, 1.74 mmol, 1 eq.) in CHCl_3_:AcOH (17:1 mL) was added over the course of 10 min at RT and under an argon atmosphere. After 16 h, the reaction was diluted in water, transferred to a separatory funnel and extracted with DCM. The organic layers were washed with water and dried over Na_2_SO_4_ and evaporated under reduced pressure. The crude product was purified by flash column chromatography on silica (hexane:toluene 100:0 vol.% to 97:3 vol.%). The product was obtained as a greenish oil (396 mg, 0.61 mmol, 35% yield). ^1^H NMR (400 MHz, CDCl_3_, ppm): δ = 8.58 (s, 1H), 7.87 (m, 1H), 7.72 (m, 1H), 7.61 (d, *J* = 5.1 Hz, 1H), 7.25 – 7.21 (m, 1H), 7.18 (d, *J* = 4.1 Hz, 1H), 4.51 (d, *J* = 6.2 Hz, 2H), 1.97 (m, 1H), 1.51 – 1.12 (m, 26H), 0.86 (m, 8H). ^19^F NMR (400 MHz, CDCl_3_, ppm): δ = -129.0 (d, *J* = 18.6 Hz), -134.7 (d, *J* = 18.6 Hz). ^13^C NMR (400 MHz, CDCl_3_, ppm): δ = 157.0, 147.4 (dd, *J* = 250.1 Hz, 17.3 Hz), 149.9 (dd, *J* = 257.6 Hz, 17.5 Hz), 137.8 (d, *J* = 2.8 Hz), 134.0 (d, *J* = 8.0 Hz), 132.5 (m), 132.2 (d, *J* = 6.5 Hz), 131.0 (d, *J* = 11.2 Hz), 130.7 (m), 130.3 (d, *J* = 17.9 Hz), 129.7 (d, *J* = 2.5 Hz), 129.2, 126.7, 119.1 (d, *J* = 9.4 Hz), 117.3 (d, *J* = 4.3 Hz), 116.2 (d, *J* = 8.7 Hz), 72.2, 37.3, 31.9, 31.9, 31.3, 30.1, 29.8, 29.6, 29.4, 26.7, 26.7, 22.7, 22.7, 14.1. HRMS (ESI) calcd for C_32_H_39_BrF_2_N_2_OS_2_ [M^+.^]: 648.16498; found: 648.1651. Rf=0.15 (silica gel, hexane/toluene, 97:3 vol.%)

**Synthesis of 6,7-difluoro-2-((2-hexyldecyl)oxy)-5-(thiophen-2-yl)-8-(5-(tributylstannyl)thiophen-2-yl)quinoxaline (4).**

In a reaction flask, a mixture of compound **3** (150 mg, 0.23 mmol, 1 eq.), Sn_2_Bu_6_ (0.35 mL, 0.69 mmol, 3 eq.), Pd(OAc)_2_ (2.6 mg, 5 % mol.) and PCy3 (6.4 mg, 10 % mol.) was set to react in degassed dry 1,4-Dioxane (5 mL) at reflux and under an argon atmosphere. After 16 h, the reaction was left to cool down to RT. The reaction mixture was precipitated and filtered subsequently in cold DCM and cold pentane. The residual Sn_2_Bu_6_ was removed using a short plug of neutral Al_2_O_3_ with hexane as eluent. The product was subsequently retrieved with pure Et_2_O. The product was obtained as a dark orange oil and used without further purification for the next step. HRMS (ESI) calcd for C_44_H_66_F_2_N_2_OS_2_Sn [M^+.^]: 861.36794; found: 861.3681.

**Synthesis of 2-((Z)-2-((7-(((Z)-5-bromo-1-(dicyanomethylene)-3-oxo-1,3-dihydro-2H-inden-2-ylidene)methyl)-4,4,9,9-tetrakis(4-octylphenyl)-4,9-dihydro-s-indaceno[1,2-b:5,6-b']dithiophen-2-yl)methylene)-3-oxo-2,3-dihydro-1H-inden-1-ylidene)malononitrile (6).**

To a mixture of compound **5** (500 mg, 0.46 mmol, 1 eq.) and BF_3_·OEt_2_ (0.35 mL, 2.79 mmol, 6 eq) in toluene:Ac_2_O (160:3.2 mL) at 0 °C, a 40.8 mL solution of IC-Br (152 mg, 0.56 mmol, 1.2 eq.) in the same solvent ratio was added dropwise over the course of 30 min. After stirring for 30 min, the reaction was left to warm up at RT. After 2 h, IC (135 mg, 0.70 mmol, 1.5 eq.) and BF_3_·OEt_2_ (0.43 mL, 3.49 mmol, 7.5 eq) were added to the reaction. After a total of 24 h the reaction mixture was precipitated in methanol and filtered. The crude product was purified by flash column chromatography (hexane:toluene 6:4 to 5:5 vol.%) The pure product was obtained as a blue solid (150 mg, 0.10 mmol, 21 % yield). ^1^H NMR (400 MHz, CDCl_3_, ppm): δ = 8.93 (d, *J* = 2.8 Hz, 2H), 8.72 (d, *J* = 7.4 Hz, 1H), 8.57 (d, *J* = 8.5 Hz, 1H), 8.03 (d, *J* = 1.5 Hz, 1H), 7.98 – 7.92 (m, 1H), 7.88 (dd, *J* = 8.5 Hz, 1.8 Hz, 1H), 7.85 – 7.69 (m, 6H), 7.24 – 7.05 (m, 17H), 2.61 (t, *J* = 7.7 Hz, 4H), 1.62 (dd, *J* = 14.4, 7.0 Hz, 6H), 1.39 – 1.23 (m, 26H), 0.89 (t, *J* = 6.6 Hz, 8H). ^13^C NMR (400 MHz, CDCl_3_, ppm): δ = 187.2, 185.7, 159.1, 158.4, 158.1, 157.3, 156.9, 156.8, 155.3, 141.2, 140.4, 140.3, 139.0, 139.0, 138.7, 138.6, 138.2, 138.1, 137.8, 137.7, 137.2, 137.2, 137.0, 136.7, 136.2, 135.7, 134.0, 133.3, 128.6, 127.6, 126.4, 125.8, 125.3, 124.2, 122.6, 121.4, 120.5, 118.9, 118.8, 113.3, 113.2, 68.3, 68.3, 61.8, 61.7, 34.3, 30.7, 30.1, 28.5, 28.2, 28.2, 28.0, 21.4, 12.9. MALDI-TOF calcd for C_98_H_97_BrN_4_O_2_S_2_ [M+H]^+^: 1508.62; found: 1508.0682.

**Synthesis of 2-((Z)-2-((7-(((Z)-1-(dicyanomethylene)-3-oxo-1,3-dihydro-2H-inden-2-ylidene)methyl)-4,4,9,9-tetrakis(4-octylphenyl)-4,9-dihydro-s-indaceno[1,2-b:5,6-b']dithiophen-2-yl)methylene)-5-(5-(6,7-difluoro-3-((2-hexyldecyl)oxy)-8-(thiophen-2-yl)quinoxalin-5-yl)thiophen-2-yl)-3-oxo-2,3-dihydro-1H-inden-1-ylidene)malononitrile (AJC1).**

In a reaction vial, a mixture of compound **6** (93 mg, 0.06 mmol, 1 eq.), compound **4** (64 mg, 0.07 mmol, 1.2 eq.), Pd_2_dba_3_ (1.1 mg, 0.001 mmol, 2 mol%.) and P(*o*-tolyl)_3_ (1.5 mg, 0.005 mmol, 8 mol%.) was set to react in degassed dry toluene at reflux and under an argon atmosphere. After 16 h, the solvent was evaporated under reduced pressure and the crude product was purified by flash column chromatography on silica (hexane:toluene 40:60 vol.%). **AJC1** was obtained as a greenish blue solid (30 mg, 0.015 mmol, 24% yield). ^1^H NMR (400 MHz, C_2_D_2_Cl_4_, ppm): δ = 8.92 (m, 2H), 8.75 (m, *J* = 8.5 Hz, 7.9 Hz, 2H), 8.67 (s, 1H), 8.28 (s, 1H), 8.09 (m, *J* = 8.6 Hz, 3.1 Hz, 2H), 7.99 (m, *J* = 7.8 Hz, 3.1 Hz, 2H), 7.93 – 7.75 (m, 5H), 7.72 (d, *J* = 4.0 Hz, 1H), 7.66 (d, *J* = 5.1 Hz, 1H), 7.30 (m, 1H), 7.21 (m, 16H), 4.67 (d, *J* = 6.0 Hz, 2H), 2.67 (t, *J* = 7.7 Hz, 8H), 2.06 (m, 1H), 1.85 – 1.18 (m, 95H), 1.17 – 0.71 (m, 20H). ^19^F NMR (400 MHz, C_2_D_2_Cl_4_, ppm): δ = -127.8 (d, *J* = 19.0 Hz), -134.2 (d, *J* = 19.1 Hz). ^13^C NMR (400 MHz, CDCl_3_, ppm): δ = 188.0, 187.8, 187.7, 161.2, 160.5, 159.7, 158.9, 158.8, 158.5, 158.3, 157.5, 156.9, 144.2, 142.3, 141.8, 141.7, 141.0, 140.7, 140.3, 139.0, 138.7, 138.4, 138.2, 137.9, 137.2, 135.2, 135.0, 134.5, 134.3, 132.8, 132.8, 132.1, 132.0, 131.7, 131.5, 131.4, 129.9, 128.8, 128.0, 127.8, 127.0, 126.8, 126.2, 126.1, 125.8, 125.3, 123.9, 123.6, 123.3, 123.0, 120.3, 120.1, 119.9, 119.8, 116.4, 116.3, 114.8, 114.7, 114.6, 114.5, 72.1, 69.0, 63.4, 37.9, 35.6, 31.9, 31.0, 30.0, 29.7, 29.6, 29.5, 29.4, 27.0, 22.6, 13.9.

^1^H NMR (400 MHz, CDCl_3_, ppm): δ = 8.90 (m, 2H), 8.70 (m, *J* = 8.5 Hz, 7.8 Hz, 2H), 8.63 (s, 1H), 8.17 (s, 1H), 8.02 (m, 2H), 7.92 (m, 2H), 7.82 – 7.70 (m, 5H), 7.67 (d, *J* = 4.1 Hz, 1H), 7.63 (d, *J* = 5.3 Hz, 1H), 7.14 (m, 1H), 7.14 (m, 16H), 4.58 (d, *J* = 5.9 Hz, 2H), 2.59 (t, *J* = 7.8 Hz, 8H), 1.99 (m, 1H), 1.83 – 0.98 (m, 95H), 0.98 – 0.74 (m, 20H). ^19^F NMR (400 MHz, CDCl_3_, ppm): δ = -128.2 (d, *J* = 19.2 Hz), -134.5 (d, *J* = 19.1 Hz). ^13^C NMR (400 MHz, CDCl_3_, ppm): δ = 188.4, 188.0, 160.3, 159.4, 158.7, 158.7, 158.0, 158.0, 157.1, 156.4, 156.4, 144.0, 142.4, 141.7, 141.7, 141.5, 140.6, 140.3, 140.0, 139.4, 138.5, 138.4, 138.2, 138.0, 137.9, 137.1, 136.9, 135.5, 135.3, 134.6, 134.5, 134.4, 133.9, 133.8, 132.3, 132.0, 131.8, 131.5, 131.3, 131.2, 130.6, 130.0, 128.8, 127.7, 126.8, 126.1, 125.7, 125.4, 123.8, 122.8, 122.5, 120.0, 119.8, 119.7, 119.5, 118.0, 116.1, 114.9, 114.7, 114.6, 74.1, 73.8, 73.6, 71.9, 69.5, 68.4, 63.0, 37.4, 35.6, 31.9, 31.8, 31.4, 30.1, 29.7, 29.6, 29.5, 29.4, 29.4, 29.2, 26.8, 26.8, 22.7, 14.1.

MALDI-TOF calcd for C_130_H_136_F_2_N_6_O_3_S_4_ [M+H]^+^: 1996.96; found: 1996.7661.

### Synthesis of AJC2

Figure S2. Synthesis pathway of AJC2.

**Synthesis of 5-(5-bromothiophen-2-yl)-6,7-difluoro-2-((2-hexyldecyl)oxy)-8-(thiophen-2-yl)quinoxaline (3’).**

To a solution of compound **2** (995 mg, 1.74 mmol, 1 eq.) in CHCl_3_ (100 mL), a solution of N‑bromosuccinimide (310 mg, 1.74 mmol, 1 eq.) in CHCl_3_:AcOH (17:1 mL) was added over the course of 10 min at RT and under an argon atmosphere. After 16 h, the reaction was diluted in water, transferred to a separatory funnel and extracted with DCM. The organic layers were washed with water and dried over Na_2_SO_4_ and evaporated under reduced pressure. The crude product was purified by flash column chromatography on silica (hexane:toluene 100:0 vol.% to 97:3 vol.%). The product was obtained as a greenish oil (218 mg, 0.34 mmol, 19% yield). ^1^H NMR (400 MHz, CDCl_3_, ppm): δ = 8.56 (s, 1H), 7.94 (m, 1H), 7.71 (dd, *J* = 4.2, 1.2 Hz 1H), 7.59 (dd, *J* = 5.2, 0.9 Hz, 1H), 7.25 – 7.22 (dd, *J* = 5.2, 3.9 Hz, 1H), 7.17 (d, *J* = 4.2 Hz, 1H), 4.50 (d, *J* = 5.7 Hz, 2H), 1.92 (m, 1H), 1.55 – 1.14 (m, 26H), 0.86 (m, 8H). ^19^F NMR (400 MHz, CDCl_3_, ppm): δ = -129.1 (d, *J* = 18.4 Hz), -134.5 (d, *J* = 18.1 Hz). ^13^C NMR (400 MHz, CDCl_3_, ppm): δ = 157.2, 150.0 (dd, *J* = 251.2 Hz, 17.3 Hz), 147.8 (dd, *J* = 247.0 Hz, 17.7 Hz), 137.1 (d, *J* = 2.8 Hz), 134.5 (d, *J* = 7.4 Hz), 132.8 (m), 131.6 (d, *J* = 6.8 Hz), 130.9 (d, *J* = 16.0 Hz), 130.7 (m), 130.4 (d, *J* = 14.6 Hz), 129.4, 129.0 (d, *J* = 2.5 Hz), 126.5, 118.3 (d, *J* = 4.4 Hz), 117.9 (d, *J* = 8.8 Hz), 117.1 (d, *J* = 9.1 Hz), 71.3, 37.5, 31.9, 31.9, 31.4, 30.0, 29.7, 29.6, 29.3, 26.8, 26.8, 22.7, 14.1. HRMS (ESI) calcd for C_32_H_40_BrF_2_N_2_OS_2_ [M+H]^+^: 649.17335; found: 648.1729. Rf=0.32 (silica gel, hexane/toluene, 97:3 vol.%)

**Synthesis of 6,7-difluoro-2-((2-hexyldecyl)oxy)-8-(thiophen-2-yl)-5-(5-(tributylstannyl)thiophen-2-yl)quinoxaline (4’).**

In a reaction flask, a mixture of compound **3’** (50 mg, 0.08 mmol, 1 eq.), Sn_2_Bu_6_ (0.12 mL, 0.23 mmol, 3 eq.), Pd(OAc)_2_ (0.9 mg, 5 % mol.) and PCy3 (2.2 mg, 10 % mol.) was set to react in degassed dry 1,4-Dioxane (1.5 mL) at reflux and under an argon atmosphere. After 6 h, the reaction was left to cool down to RT. The residual Sn_2_Bu_6_ was removed using a short plug of neutral Al_2_O_3_ with hexane as eluent. The product was subsequently retrieved with pure Et_2_O. The product was obtained as a dark yellow oil and used without further purification for the next step.

**Synthesis of 2-((Z)-2-((7-(((Z)-1-(dicyanomethylene)-3-oxo-1,3-dihydro-2H-inden-2-ylidene)methyl)-4,4,9,9-tetrakis(4-octylphenyl)-4,9-dihydro-s-indaceno[1,2-b:5,6-b']dithiophen-2-yl)methylene)-5-(5-(6,7-difluoro-2-((2-hexyldecyl)oxy)-8-(thiophen-2-yl)quinoxalin-5-yl)thiophen-2-yl)-3-oxo-2,3-dihydro-1H-inden-1-ylidene)malononitrile (AJC2).**

In a reaction vial, a mixture of compound **6** (15 mg, 0.01 mmol, 1 eq.), compound **4’** (9.4 mg, 0.07 mmol, 1.2 eq.), Pd_2_dba_3_ (1.1 mg, 0.001 mmol, 2 mol%.) and P(*o*-tolyl)_3_ (1.5 mg, 0.005 mmol, 8 mol%.) was set to react in degassed dry toluene at reflux and under an argon atmosphere. After 16 h, the solvent was evaporated under reduced pressure and the crude product was purified by flash column chromatography on silica (hexane:toluene 40:60 vol.%). **AJC2** was obtained as a greenish blue solid (4 mg, 0.002 mmol, 20% yield). ^1^H NMR (400 MHz, C_2_D_2_Cl_4_, ppm): δ = 8.92 (m, 2H), 8.77 (d, *J* = 8.5 Hz, 1H), 8.73 (d, *J* = 7.8 Hz, 1H), 8.70 (s, 1H), 8.31 (d, *J* = 1.9 Hz, 1H), 8.16 (d, *J* = 8.4 1H), 8.08 (d, *J* = 4.1 Hz, 1H), 8.02 (m, 1H), 7.98 (d, *J* = 7.4 1H), 7.90 – 7.76 (m, 5H), 7.71 (d, *J* = 4.1 Hz, 1H), 7.67 (d, *J* = 5.1 Hz, 1H), 7.30 (m,1H), 7.26 – 7.14 (m, 16H), 4.63 (d, *J* = 6.0 Hz, 2H), 2.67 (t, *J* = 7.7 Hz, 8H), 2.02 (m, 1H), 1.70 (m, 8H), 1.56 – 1.25 (m, 5H), 1.05 – 0.85 (m, 20H). ^19^F NMR (400 MHz, C_2_D_2_Cl_4_, ppm): δ = -128.6 (*J* = 18.2 Hz), -133.2 (*J* = 18.1 Hz). ^13^C NMR (400 MHz, C_2_D_2_Cl_4_, ppm): δ = 188.7, 159.4, 158.4, 156.9, 156.1, 142.3, 140.3, 139.1, 138.6, 138.3, 138.1, 137.7, 135.1, 134.5, 132.4, 131.8, 130.9, 130.7, 129.1, 128.8, 127.9, 125.8, 125.7, 125.5, 125.3, 124.0, 123.8, 123.2, 122.3, 120.2, 118.5, 118.3, 99.8, 71.8, 63.4, 39.4, 37.2, 35.6, 34.6, 34.3, 33.8, 32.9, 31.9, 31.9, 30.9, 30.2, 30.1, 29.7, 29.5, 29.4, 29.3, 29.1, 27.2, 27.0, 22.6, 22.6, 19.8, 13.9. MALDI-TOF calcd for C_130_H_136_F_2_N_6_O_3_S_4_ [M+H]^+^: 1996.96; found: 1997.3789

### Synthesis of the monomers

**Synthesis of 2,2'-((2Z,2'Z)-((4,4,9,9-tetrakis(4-octylphenyl)-4,9-dihydro-s-indaceno[1,2-b:5,6-b']dithiophene-2,7-diyl)bis(methaneylylidene))bis(5-bromo-3-oxo-2,3-dihydro-1H-indene-2,1-diylidene))dimalononitrile (IDTe-2Br).**

To a mixture of compound **5** (699 mg, 0.65 mmol, 1 eq.) and **IC-Br** (335 mg, 1.3 mmol, 2 eq.) in toluene:Ac_2_O (33:0.7 mL) at RT, boron trifluoride etherate (0.29 mL, 2.37 mmol, 10 eq.) was added dropwise over the course of 2 min. After 16 h, the reaction was precipitated on methanol and filtered. The crude product was purified via column chromatography on silica (hexane:toluene 60:40 to 40:60 vol. %). The product was obtained as a black powder (1.03 g, 96% yield). ^1^H NMR (400 MHz, CDCl_3_, ppm): δ = 8.91 (s, 2H), 8.54 (d, *J* = 8.5 Hz, 2H), 8.00 (d, *J* = 1.8 Hz, 2H), 7.86 (dd, *J* = 1.9, 8.5 Hz, 2H), 7.73 (m, 4H), 7.13 (m, 16H), 2.58 (t, *J* = 7.7 Hz, 8H), 1.59 (m, 8H), 1.28 (m, 40H), 0.86 (t, *J* = 6.8 Hz, 12H). ^13^C NMR (400 MHz, CDCl_3_, ppm): δ = 187.0, 159.6, 158.4, 156.7, 142.6, 141.7, 140.3, 139.3, 139.0, 138.5, 138.36, 138.0, 137.3, 129.9, 129.0, 127.8, 127.1, 126.7, 122.0, 120.3, 114.6, 114.5, 69.7, 63.1, 35.7, 32.2, 31.5, 29.6, 29.5, 29.4, 22.8, 14.2. MALDI-TOF calcd for C_98_H_96_Br_2_N_4_O_2_S_2_ [M+H]^+^: 1586.54; found: 1587.0521

**Synthesis of 3,9-bis(2-methylene-((3-(1,1-dicyanomethylene)-7-bromo)-indanone))-5,5,11,11-tetrakis(4-octylphenyl)-dithieno[2,3-d:2’,3’-d’]-s-indaceno[1,2-b:5,6-b’]dithiophene (ITIC-2Br).**

To a mixture of **ITIC-2CHO** (200 mg, 0.17 mmol, 1 eq.) and **IC-Br** (92 mg, 0.47 mmol, 2 eq.) in toluene:Ac_2_O (8.5:0.15 mL) at RT, boron trifluoride etherate (0.21 mL, 1.68 mmol, 10 eq.) was added dropwise over the course of 2 min. After 16 h, the reaction was precipitated on methanol and filtered. The crude product was purified via column chromatography on silica (hexane:toluene 60:40 to 40:60 vol. %). The product was obtained as a dark blue powder (277 mg, 97% yield). ^1^H NMR (400 MHz, CDCl_3_, ppm): δ = 8.86 (s, 2H), 8.53 (d, *J* = 8.3 Hz, 2H), 8.23 (s, 2H), 8.00 (d, *J* = 1.9 Hz, 2H), 7.84 (dd, *J* = 8.4, 1.9 Hz, 2H), 7.66 (s, 2H), 7.18 (m, 16H), 2.57 (t, *J* = 7.8 Hz, 8H), 1.59 (m, 11H), 1.28 (m, 44H), 0.86 (m, 13H). ^13^C NMR (400 MHz, CDCl_3_, ppm): δ = 186.7, 159.4, 155.8, 153.5, 147.7, 147.5, 143.9, 142.6, 139.7, 138.8, 138.7, 138.5, 138.2, 137.9, 137.3, 137.0, 129.8, 128.9, 127.9, 127.0, 126.5, 122.0, 118.7, 114.5, 114.4, 77.2, 69.5, 63.3, 35.6, 31.9, 31.3, 29.5, 29.5, 29.2, 22.7, 14.1.

**Synthesis of 2,2'-((2Z,2'Z)-((3,9-bis(2-butyloctyl)-12,13-bis(2-ethylhexyl)-12,13-dihydro-[1,2,5]thiadiazolo[3,4-e]thieno[2'',3'':4',5']thieno[2',3':4,5]pyrrolo[3,2-g]thieno[2',3':4,5]thieno[3,2-b]indole-2,10-diyl)bis(methaneylylidene))bis(5-bromo-3-oxo-2,3-dihydro-1H-indene-2,1-diylidene))dimalononitrile (L8-BO-2Br).**

To a mixture of **L8-BO-2CHO** (250 mg, 0.24 mmol, 1 eq.) and **IC-Br** (129 mg, 0.47 mmol, 2 eq.) in toluene:Ac_2_O (12:0.2 mL) at RT, boron trifluoride etherate (0.29 mL, 2.37 mmol, 10 eq.) was added dropwise over the course of 2 min. After 16 h, the reaction was precipitated on methanol and filtered. The crude product was purified via column chromatography on silica (hexane:toluene 60:40 to 40:60 vol. %). The product was obtained as a black powder (290 mg, 78% yield). ^1^H NMR (400 MHz, CDCl_3_, ppm): δ = 9.18 (s, 2H), 8.59 (d, *J* = 8.5 Hz, 2H), 8.06 (d, *J* = 1.8 Hz, 2H), 7.88 (dd, *J* = 8.5, 1.9 Hz, 2H), 4.81 (d, *J* = 7.8 Hz, 4H), 3.19 (d, *J* = 7.6 Hz, 4H), 2.11 (m, 4H), 1.54 – 0.74 (m, 75H), 0.68 (m, 6H). ^13^C NMR (400 MHz, CDCl_3_, ppm): δ = 187.0, 160.2, 153.3, 147.6, 145.3, 140.2, 138.5, 138.3, 137.7, 136.0, 135.6, 134.2, 133.8, 131.7, 130.6, 129.5, 126.8, 126.4, 120.3, 115.5, 114.9, 113.5, 70.4, 68.4, 59.0, 55.6, 40.4, 40.1, 34.7, 33.6, 33.3, 31.8, 29.6, 28.9, 27.6, 26.6, 23.3. MALDI-TOF calcd for C_84_H_92_Br_2_N_8_O_2_S_5_ [M+.]: 1564.43; found: 1564.9985

### Synthesis of the polymers

**Typical procedure for the synthesis of PTQ10.**

In a reaction vial, a mixture of **1** (100 mg, 0.18 mmol, 1 eq.), 2,5-bis(trimethylstannyl)thiophene (72.6 mg, 0.18 mmol, 1 eq.), Pd_2_dba_3_ (3.2 mg, 0.004 mmol, 2 mol%.) and P(*o*-tolyl)_3_ (4.3 mg, 0.014, 8 mol%.) was diluted in degassed dry toluene (8.9 mL, 20 mM). The reaction mixture is then heated at reflux and stirred. After 4 min (3 kg/mol) or 8 min (40 kg/mol), the reaction mixture was precipitated in a large volume of methanol and filtered. The crude product was purified through a Soxhlet apparatus with methanol, acetone, ethyl acetate, dichloromethane and chloroform. The relevant fractions were concentrated under reduced pressure and precipitated in methanol to yield PTQ10 as a dark purple solid. Yields are typically 73-93%.


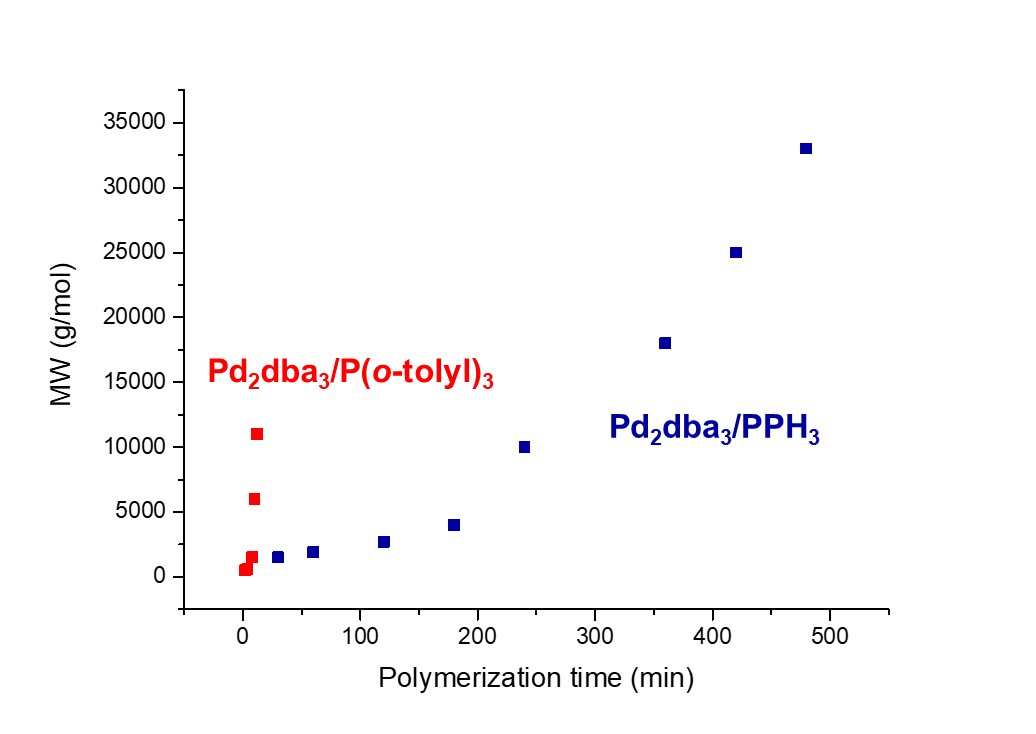


Figure S3. Kinetic study of the polymerization of PTQ10 performed with either PPH_3_ (blue) or P(*o*-tolyl)_3_ (red) as the phosphine source.

**Typical procedure for the synthesis of PIDTe.**

In a reaction vial, a mixture of **IDTe-2Br** (100 mg, 0.06 mmol, 1 eq.), 2,5-bis(trimethylstannyl)thiophene (25.9 mg, 0.06 mmol, 1 eq.), Pd_2_dba_3_ (1.2 mg, 0.001 mmol, 2 mol%.) and P(*o*-tolyl)_3_ (1.5 mg, 0.005 mmol, 8 mol%.) was diluted in degassed dry toluene (3.2 mL, 20 mM). The reaction mixture is then heated at reflux and stirred. After 16 h, the reaction mixture was precipitated in a large volume of methanol and filtered. The crude product was purified through a Soxhlet apparatus with methanol, acetone, ethyl acetate, dichloromethane and chloroform. The relevant fractions were concentrated under reduced pressure and precipitated in methanol to yield PIDTe as a dark blue solid. Yields are typically 75-98%.

### Synthesis of the block copolymers


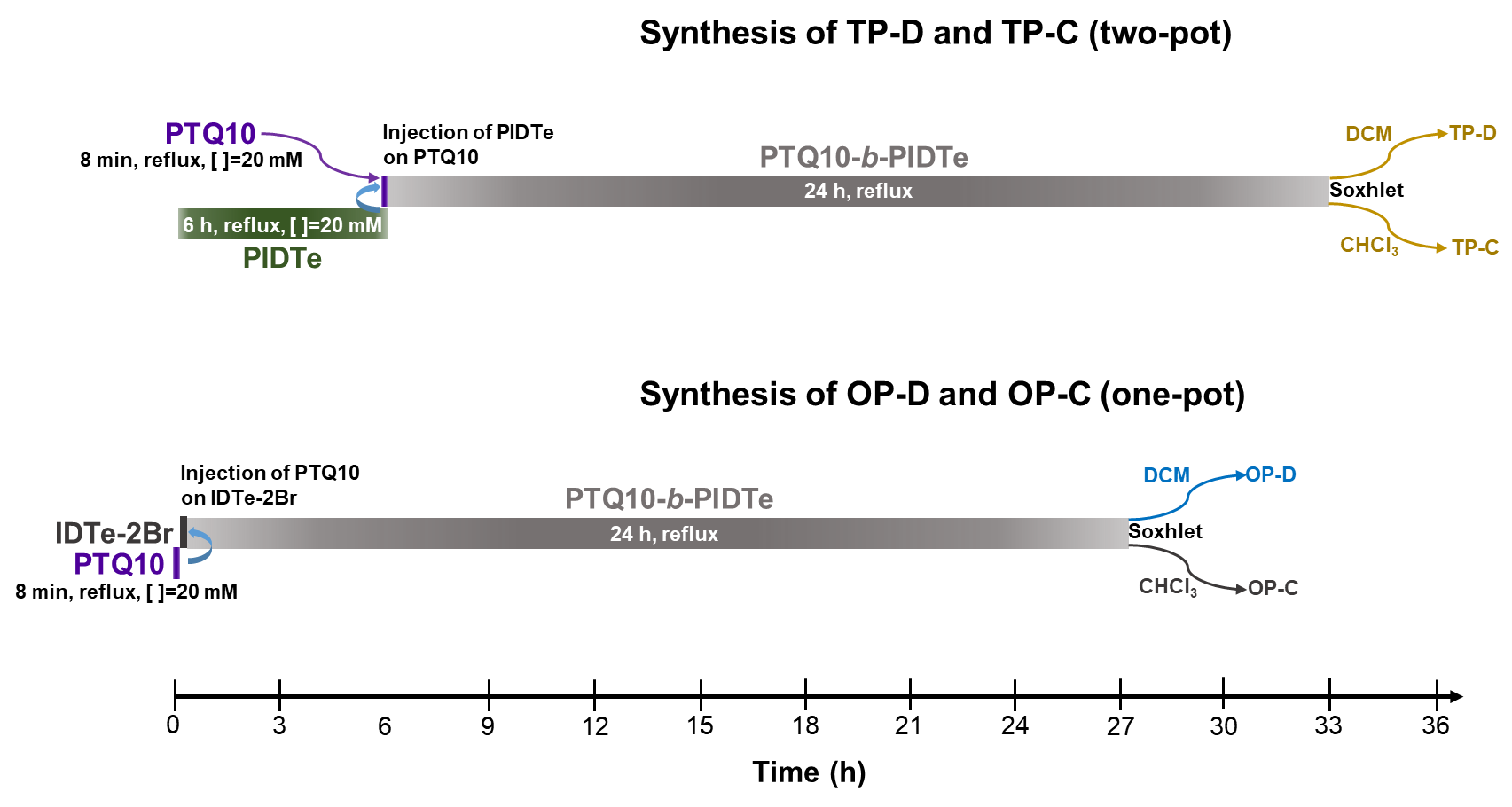


Figure S4. Illustration of the procedures used for the synthesis of the block copolymers.

**Synthesis of TP-D and TP-C.**

In a reaction vial, a 2.5 mL toluene solution of an active polymerization of **PIDTe** (80 mg of **IDTe-2Br**, 20 mM, 6 h, prepared according to the previous protocol) was added with a syringe to a 5.9 mL toluene solution of an active polymerization of **PTQ10** (66.7 mg of **1**, 20 mM, 8 min, prepared according to the previous protocol). The reaction mixture was stirred at reflux for 24 h and precipitated in a large volume of methanol. The crude product was purified through a Soxhlet apparatus with methanol, acetone, ethyl acetate, dichloromethane and chloroform. The dichloromethane and chloroform fractions were concentrated under reduced pressure and precipitated in methanol to yield respectively TP-D (50 mg) and TP-C (16 mg) as black solids. The overall yield is 54%.

**Synthesis of OP-D and OP-C.**

In a reaction vial, a 2.5 mL toluene solution of an active polymerization of **PTQ10** (33.3 mg of **1**, 20 mM, 8 min, prepared according to the previous protocol) was added with a syringe to a reaction vial containing **IDTe-2Br** (50 mg, 0.03 mmol, 1 eq.), 2,5-bis(trimethylstannyl)thiophene (12.9 mg, 0.03 mmol, 1 eq.), Pd_2_dba_3_ (0.6 mg, 0.001 mmol, 2 mol%.) and P(*o*-tolyl)_3_ (0.8 mg, 0.005 mmol, 8 mol%.). The reaction mixture was stirred at reflux for 24 h and precipitated in a large volume of methanol. The crude product was purified through a Soxhlet apparatus with methanol, acetone, ethyl acetate, dichloromethane and chloroform. The dichloromethane and chloroform fractions were concentrated under reduced pressure and precipitated in methanol to yield respectively OP-D (22 mg) and OP-C (35 mg) as black solids. The overall yield is 47%.

**Synthesis of PTQ10-*b*-PITIC.**

In a reaction vial, a 2.5 mL toluene solution of an active polymerization of **PTQ10** (40 mg of **1**, 20 mM, 8 min, prepared according to the previous protocol) was added with a syringe to a reaction vial containing **ITIC-2Br** (60 mg, 0.04 mmol, 1 eq.), 2,5-bis(trimethylstannyl)thiophene (14.5 mg, 0.04 mmol, 1 eq.), Pd_2_dba_3_ (0.7 mg, 0.001 mmol, 2 mol%.) and P(*o*-tolyl)_3_ (0.9 mg, 0.003 mmol, 8 mol%.). The reaction mixture was stirred et reflux for 24 h and precipitated in a large volume of methanol. The crude product was purified through a Soxhlet apparatus with methanol, acetone, ethyl acetate, dichloromethane and chloroform. The chloroform fraction (20 mg) was concentrated under reduced pressure and precipitated in methanol to yield PTQ10-*b*-PITIC as a dark blue solid. The overall yield is 57%.

**Synthesis of PTQ10-*b*-PL8-BO.**

In a reaction vial, a 2.5 mL toluene solution of an active polymerization of **PTQ10** (40 mg of **1**, 20 mM, 8 min, prepared according to the previous protocol) was added with a syringe to a reaction vial containing **L8-BO-2Br** (48 mg, 0.03 mmol, 1 eq.), 2,5-bis(trimethylstannyl)thiophene (12.6 mg, 0.03 mmol, 1 eq.), Pd_2_dba_3_ (0.6 mg, 0.001 mmol, 2 mol%.) and P(*o*-tolyl)_3_ (0.8 mg, 0.005 mmol, 8 mol%.). The reaction mixture was stirred at reflux for 24 h and precipitated in a large volume of methanol. The crude product was purified through a Soxhlet apparatus with methanol, acetone, ethyl acetate, dichloromethane and chloroform. The dichloromethane fraction (25 mg) was concentrated under reduced pressure and precipitated in methanol to yield PTQ10-*b*-PL8-BO as a black solid. The overall yield is 75%.

## DFT calculations


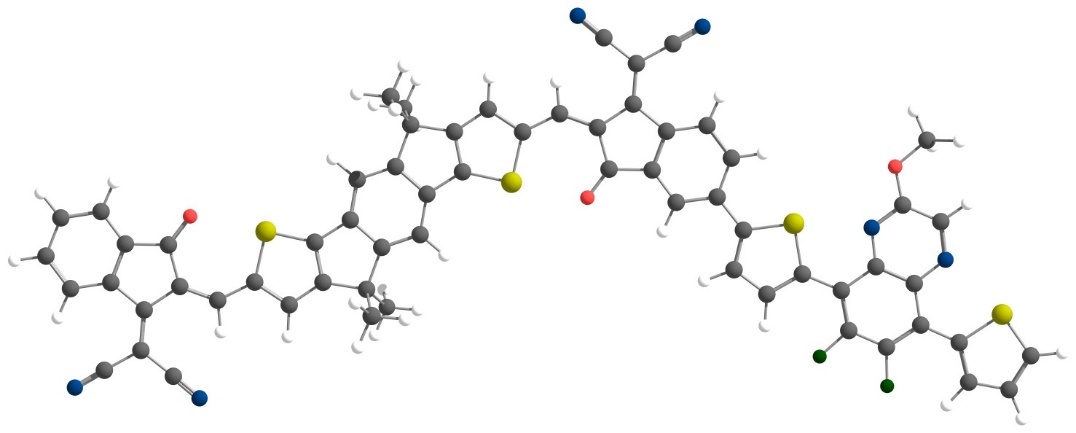

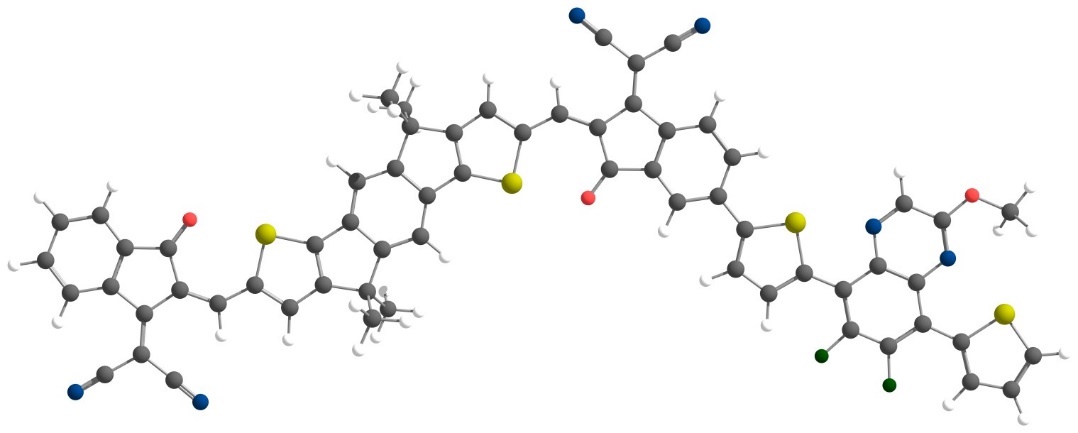


Figure S5. Optimized 3D geometries of AJC1 (top) and AJC2 (bottom).

Table S1. Summary of the TMS protons and proton 3 NMR shifts calculated by DFT

| Basis set | TMS (ppm) | AJC1 (ppm)^a^ | Calc. shift (ppm)^b^ | AJC2 (ppm)^a^ | Calc. shift (ppm)^b^ | Δ (ppm) |
| --- | --- | --- | --- | --- | --- | --- |
| pcSseg-2 | 31.13 | 22.68 | 8.45 | 22.66 | 8.47 | 0.02 |
| pcSseg-2 (CHCl_3_) | 31.05 | 22.63 | 8.48 | 22.60 | 8.51 | 0.03 |
| ^a^Only the chemical shift corresponding to proton **3** is reported ^b^Calculated based on the formula σ_calc_.= σ_TMS_-σ_DFT_ | | | | | |  |

Table S2. Summary of the isotropic fluoride NMR shifts of AJC1 and AJC2 calculated by DFT

| Basis set | AJC1 (ppm)^a^ | AJC2 (ppm)^a^ | Δ (ppm) |  |
| --- | --- | --- | --- | --- |
| pcSseg-2 | 292.2 | 301.1 | 8.9 |  |
| pcSseg-2 (CHCl_3_) | 295.8 | 304.4 | 8.7 |  |
| def2-tzvp | 295.2 | 304.1 | 8.9 |  |
| def2-tzvp (CHCl_3_) | 298.2 | 307.1 | 8.8 |  |
| 6-31G^b^ | 308.8 (148.6) | 316.8 (156.3) | 8.0 (7.7) |  |
| 6-31G (CHCl_3_)^b^ | 312.0 | 319.9 | 7.8 |  |
| 6-31G+G(d,p) | 334.5 (150.0) | 339.9 (155.5) | 5.4 (5.5) |  |
| 6-31G+G(d,p) (CHCl_3_) | 334.8 | 339.5 | 4.7 |  |
| ^a^Only the chemical shift of the fluoride atom located closest to the acceptor moiety is reported  ^b^The values in brackets correspond to the corrected shifts calculated using correlation given in Ref.^[4]^ | | | |  |

# Color code of the molecules


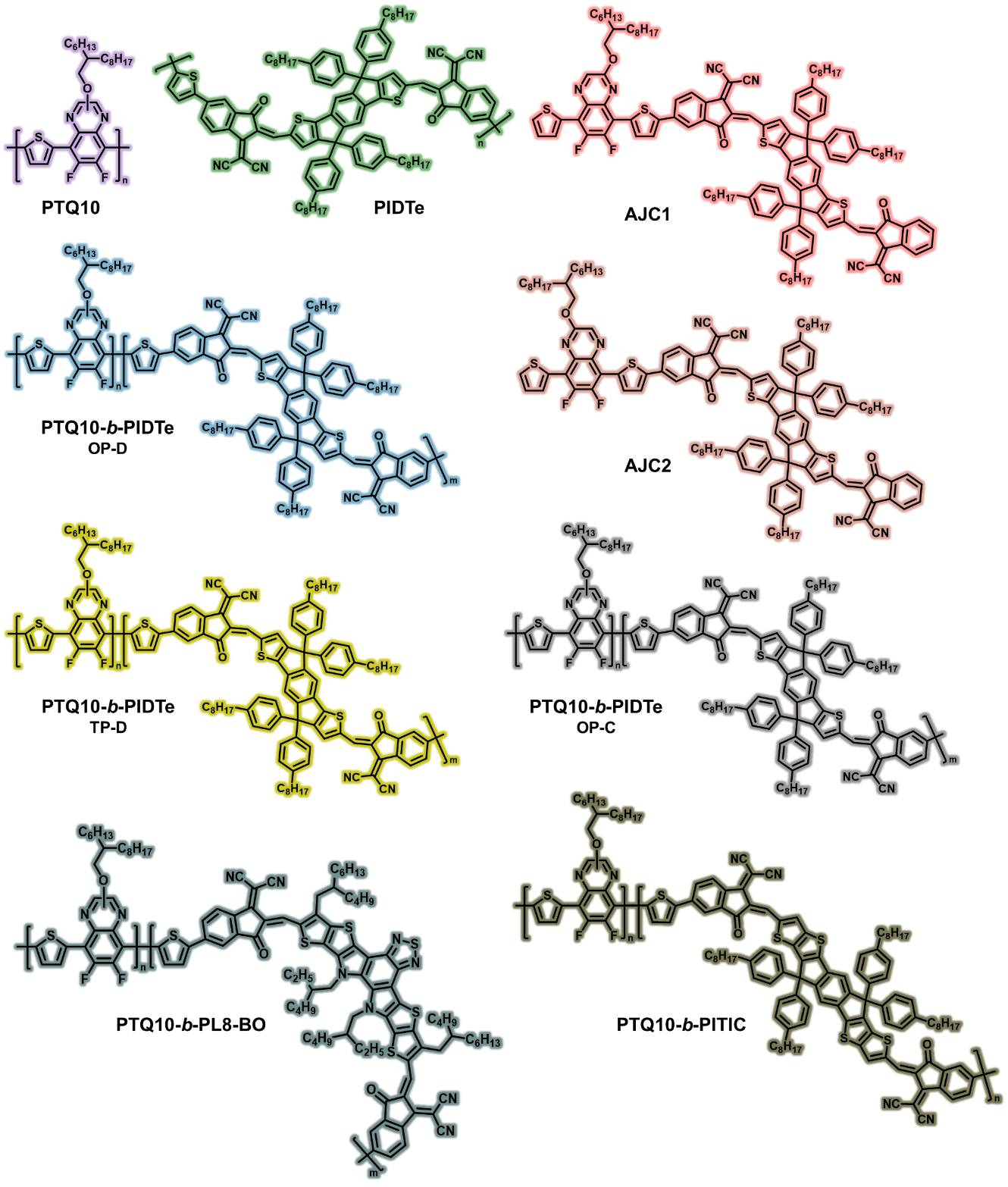


Figure S6. Color code for the molecules studied in this work.

# SEC distribution of the materials and UV-Vis spectra

Table S3. Summary of the molecular weight and molecular weight distribution parameters of the materials determined by SEC

| Material | Mn (kg/mol) | Mw (kg/mol) | Đ |
| --- | --- | --- | --- |
| TP-D (1st trial) | 10.2 | 36.4 | 3.6 |
| TP-D (2nd trial) | 8.0 | 21.8 | 2.7 |
| OP-C | 28.6 | 87.1 | 3.1 |
| OP-D | 5.3 | 13.1 | 2.5 |
| PTQ10-*b*-PL8-BO | 6.9 | 18.3 | 2.7 |
| PTQ10-*b*-PITIC | 17.1 | 63.9 | 3.7 |
| PTQ10 (1) | 3.4 | 5.9 | 1.8 |
| PTQ10 (2) | 40.0 | 110.4 | 2.8 |
| PIDTe | 12.7 | 31.4 | 2.5 |
|  |  |  |  |


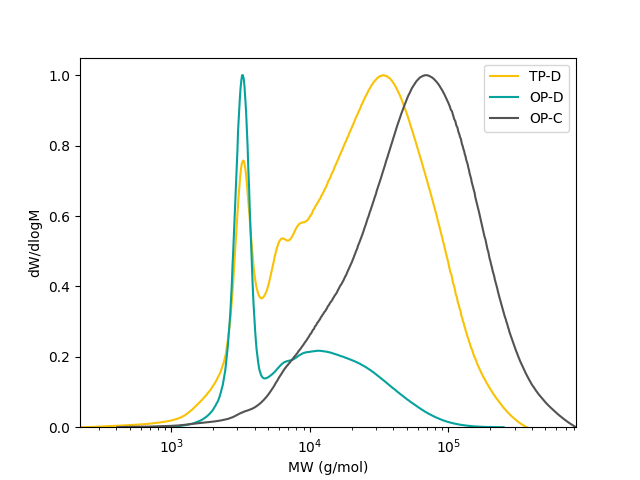


Figure S7. Molecular weight distributions of TP-D, OP-C and OP-D.


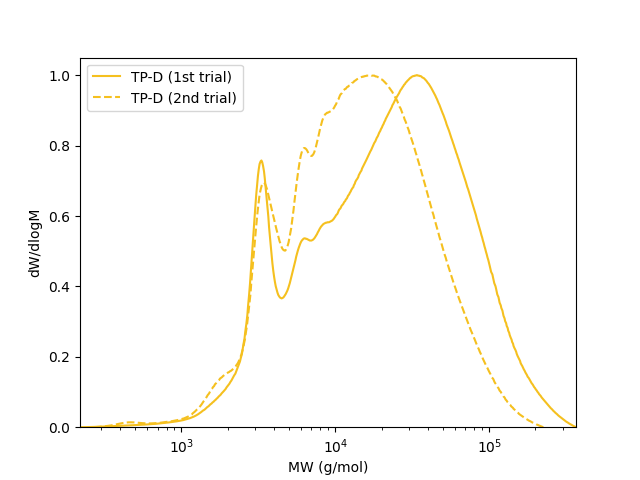

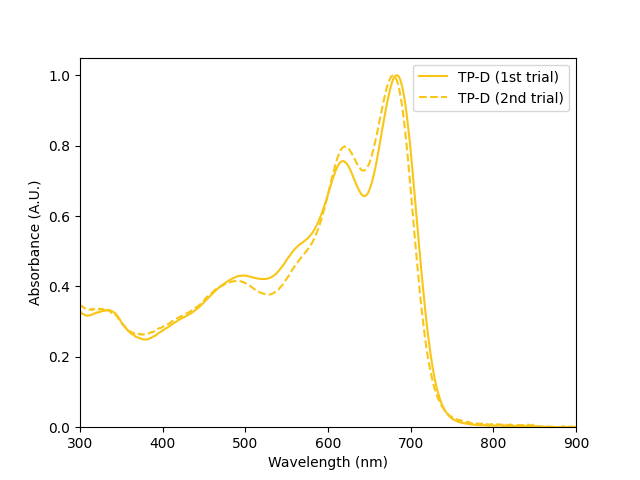


Figure S8. Molecular weight distributions (left) and UV-Vis spectra (right) of the two different synthesis of TP-D.


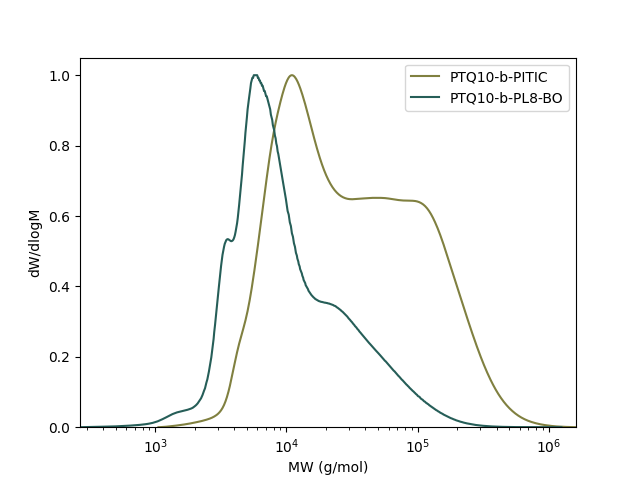


Figure S9. Molecular weight distributions of PTQ10-*b*-PITIC and PTQ10-*b*-PL8-BO


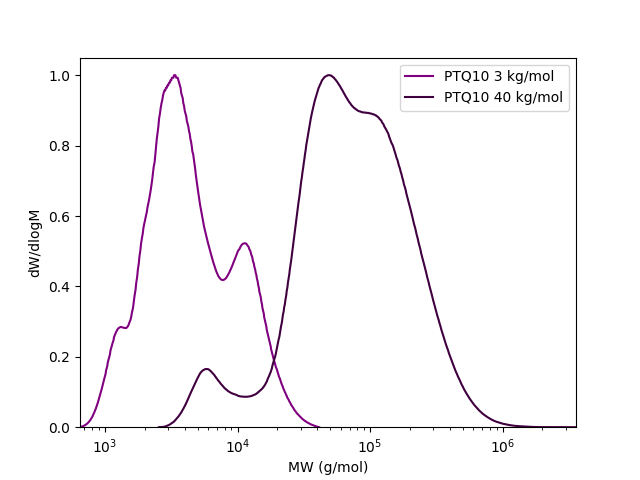


Figure S10. Molecular weight distributions of the 3 kg/mol and 40 kg/mol PTQ10.


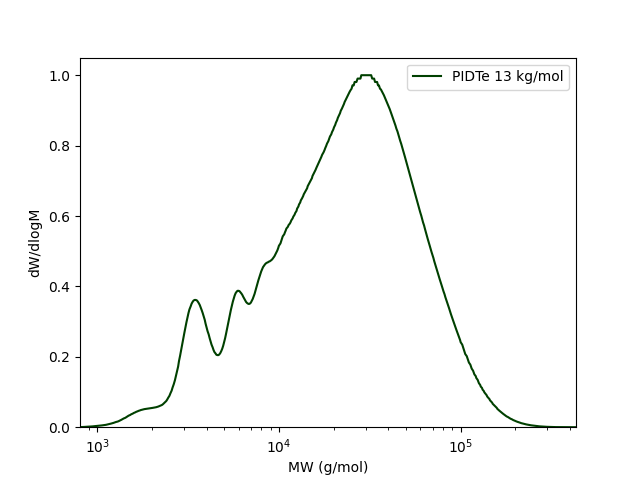


Figure S11. Molecular weight distributions of the 13 kg/mol PIDTe.


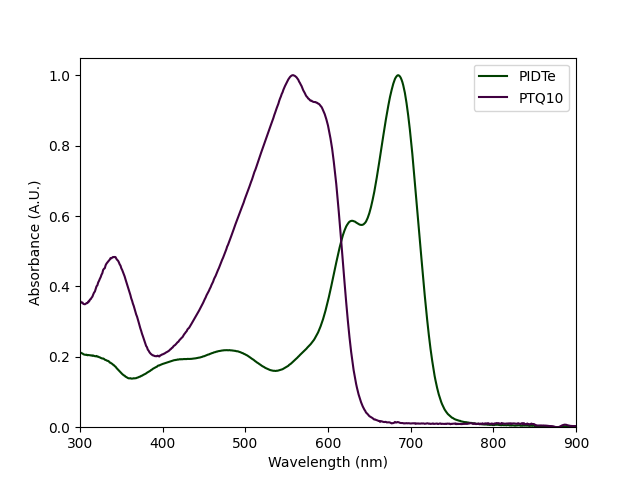


Figure S12. Normalized UV-Vis spectra of PTQ10 and PIDTe in CHCl_3_.


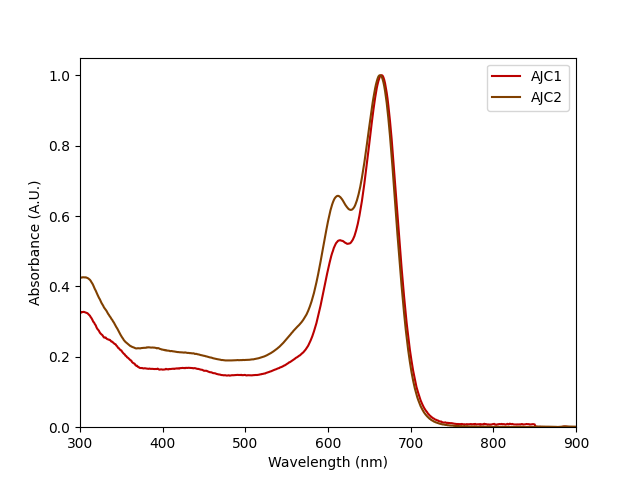


Figure S13. Normalized UV-Vis spectra of AJC1 and AJC2 in CHCl_3_.


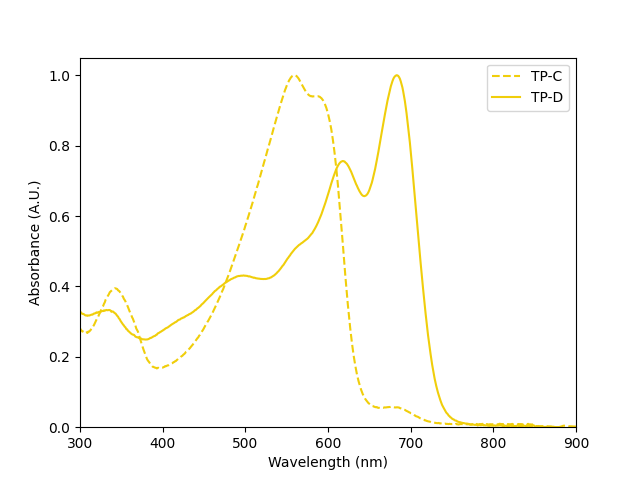


Figure S14. Normalized UV-Vis spectra of TP-D and TP-C in CHCl_3_.


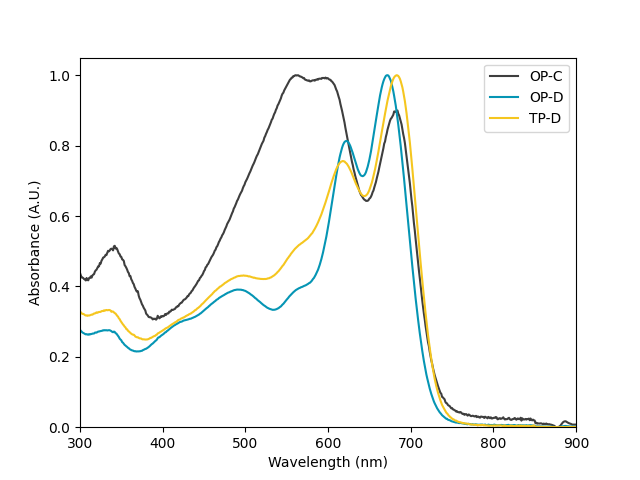


Figure S15. Normalized UV-Vis spectra of TP-D, OP-C and OP-D in CHCl_3_.


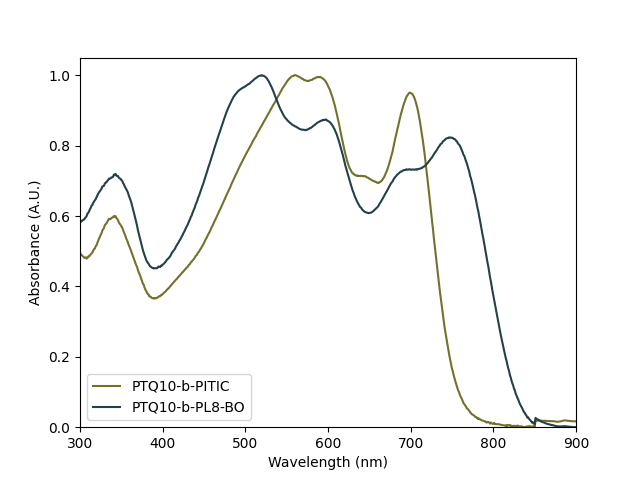


Figure S16. Normalized UV-Vis spectra of PTQ10-*b*-PITIC and PTQ10-*b*-PL8-BO in CHCl_3_.

# Single-crystal growth of compound 3

## Protocol for single-crystal growth of compound 3

In a 2 mL vial, 4 mg of compound **3** were dissolved in 0.5 mL of THF. The vial was then closed with a pierced screw cap. The vial was placed in a 50 mL container and 7 mL of MeOH where added at the bottom of the container. The container was closed and left untouched for 2 weeks. The fine yellow needles obtained were analyzed by X-ray diffraction to obtain the crystal structure of compound **3**.

## Crystal structure of compound 3


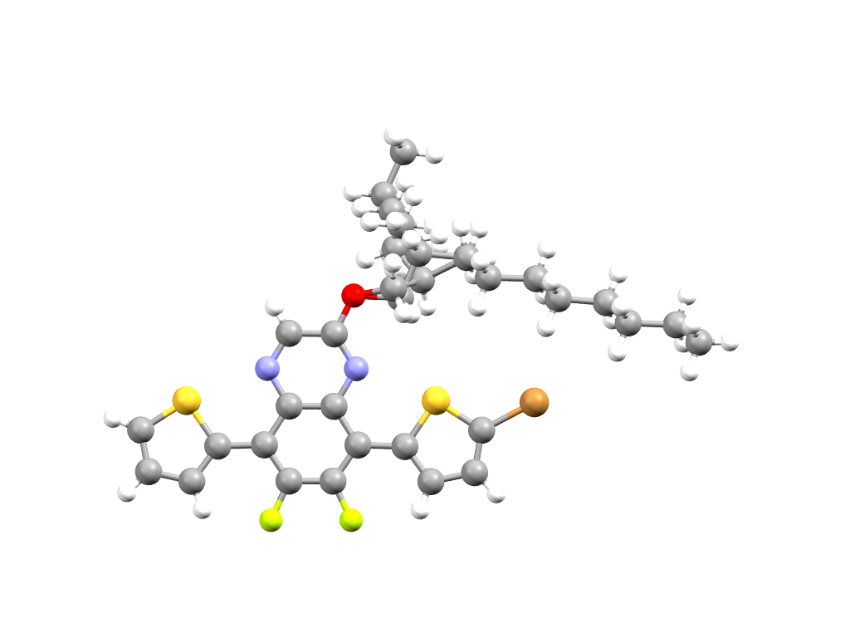

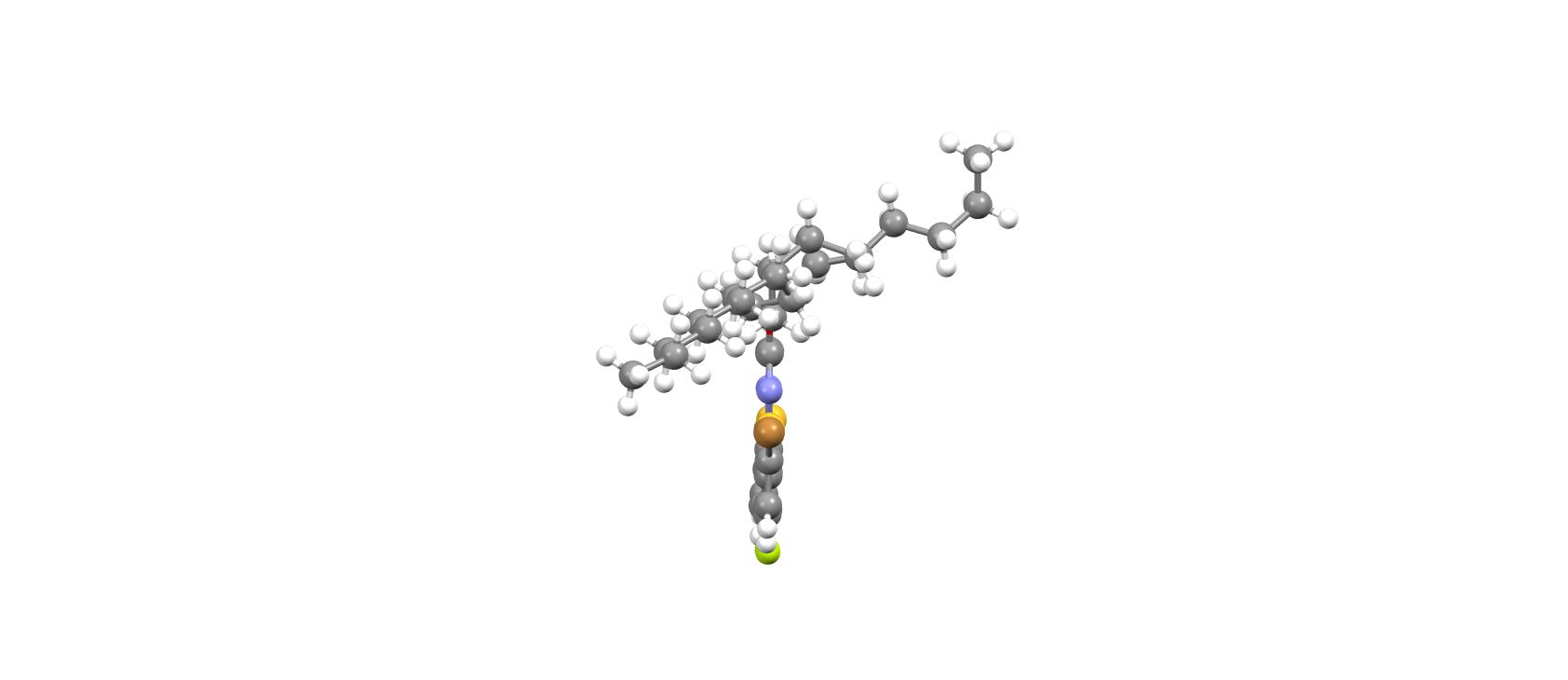


Figure S17. Front and side view of the crystal structure of compound **3**. Atoms are showed as grey (carbon), white (hydrogen), yellow (sulfur), blue (nitrogen), red (oxygen), green (fluoride) and orange (bromide) spheres.


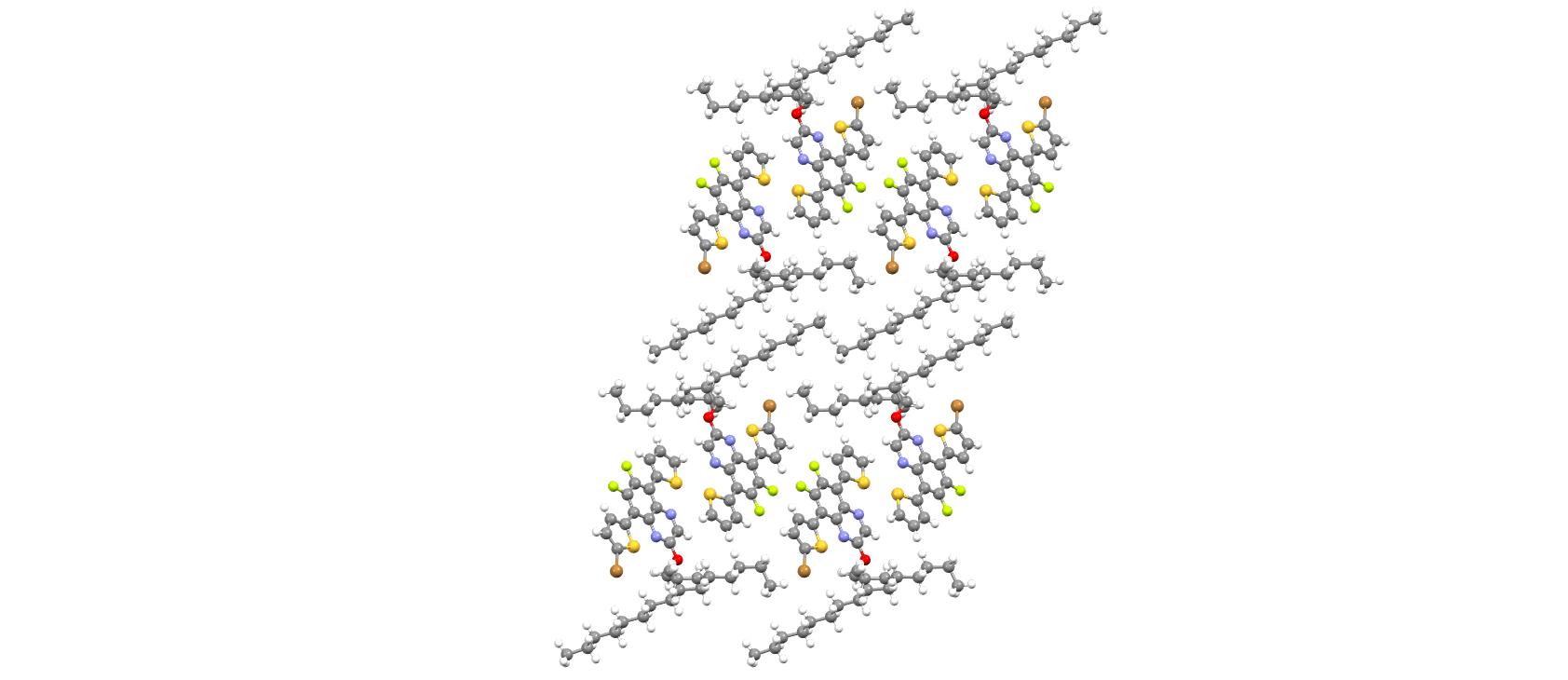


Figure S18. Crystal packing structure of compound **3**.

# NMR spectra and total ^1^H attributions of the materials

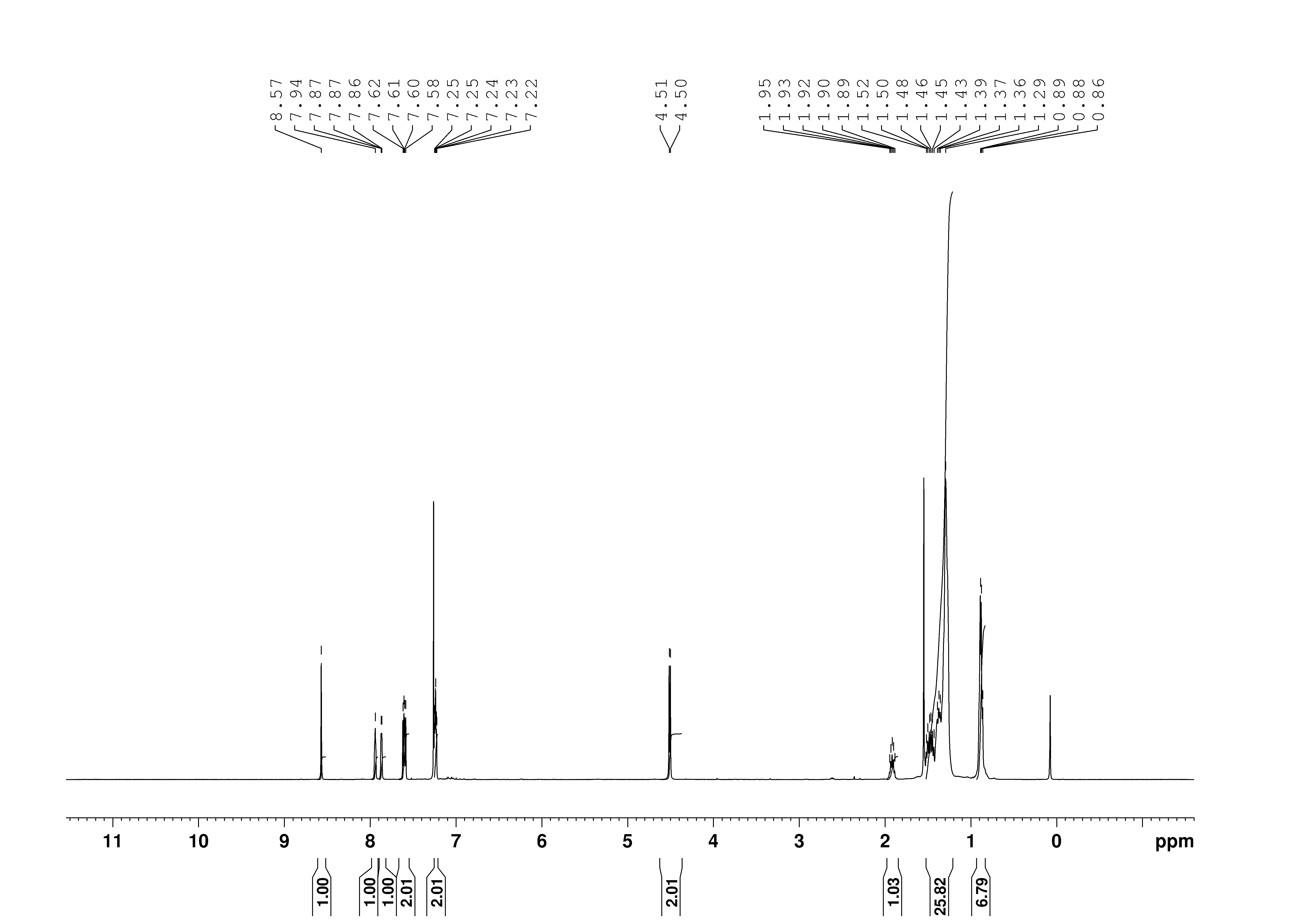


Figure S19. ^1^H NMR spectrum of compound **2** in CDCl_3_ at 298 K.

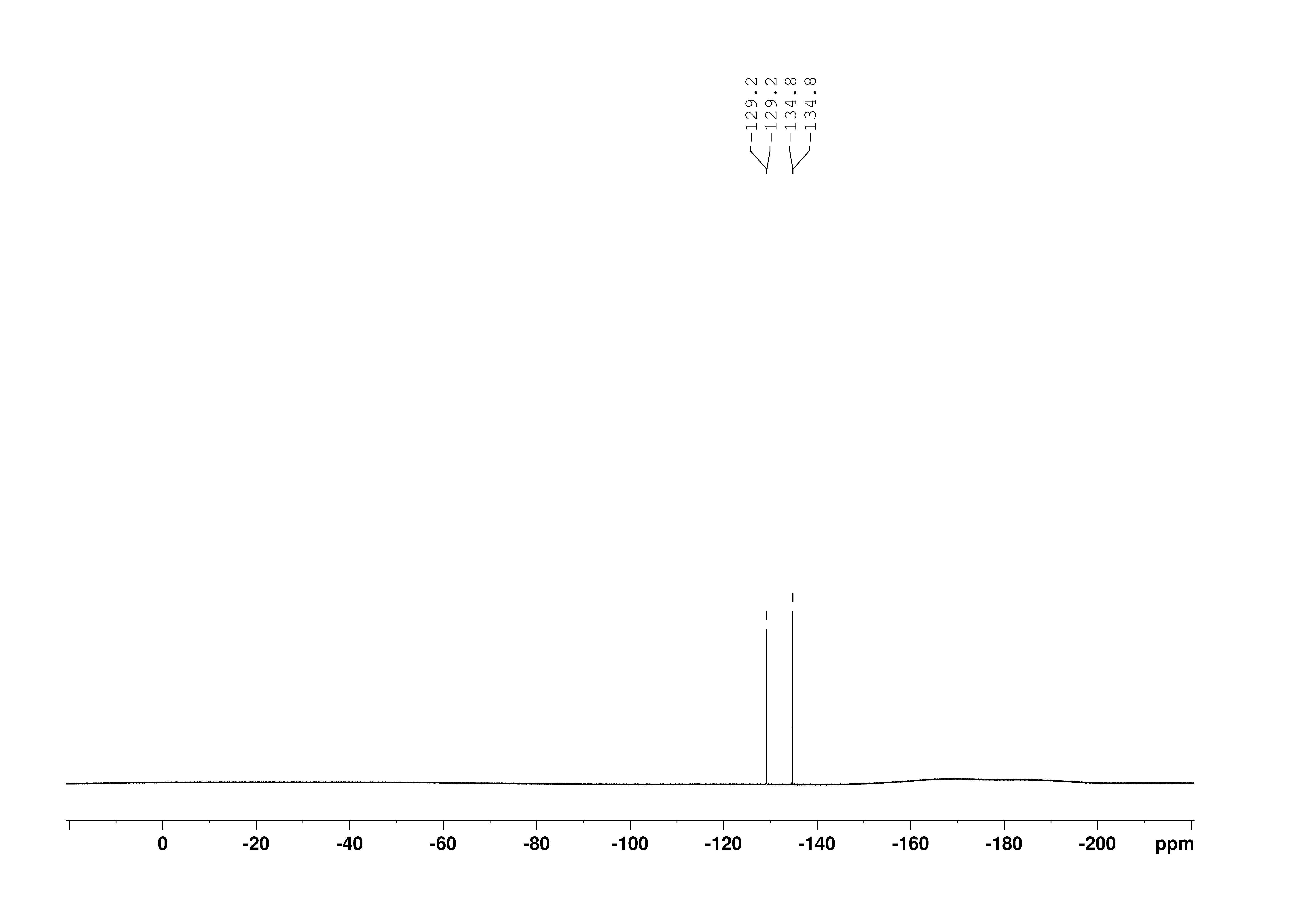


Figure S20. ^19^F NMR spectrum of compound **2** in CDCl_3_ at 298 K.


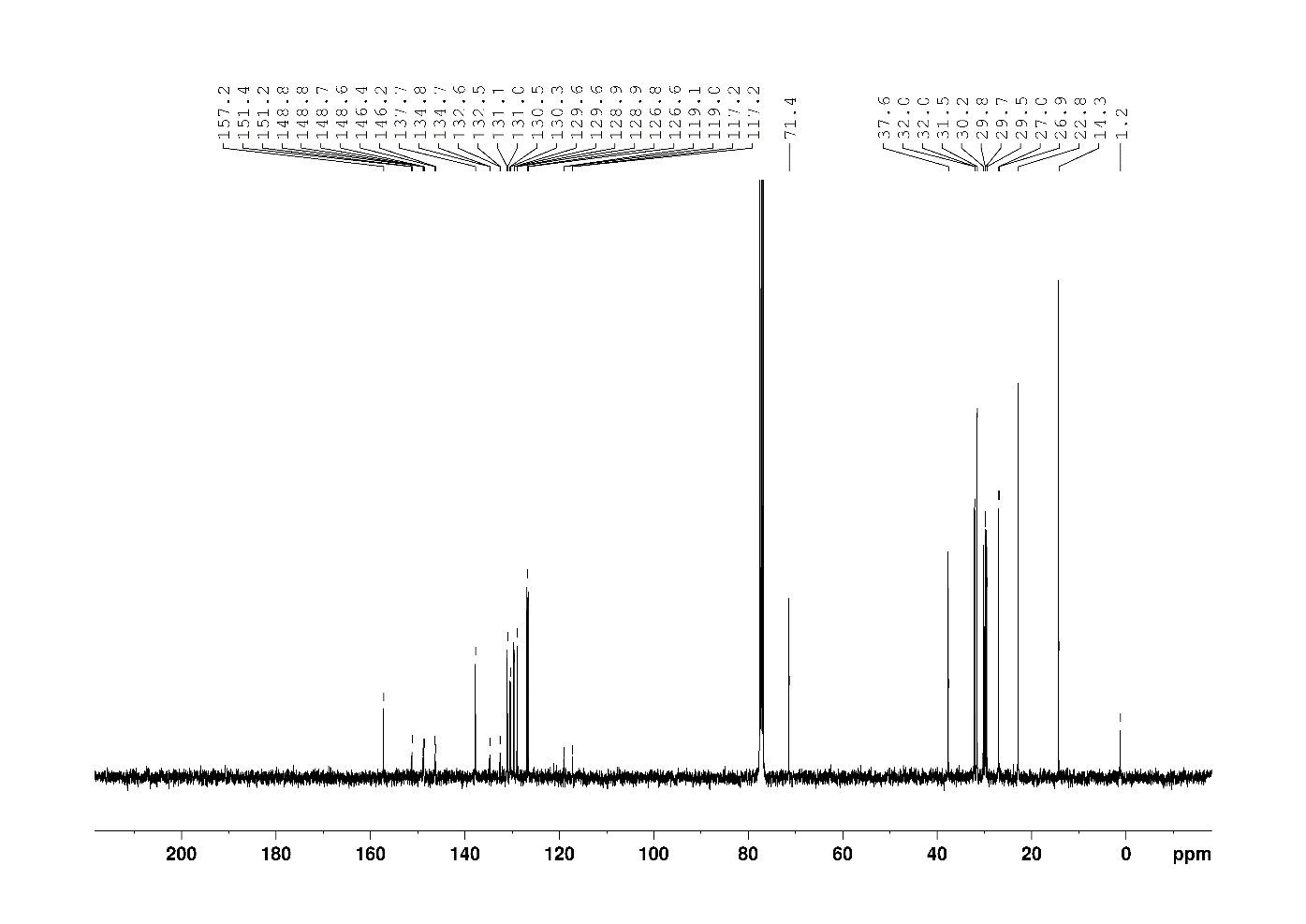


Figure S21. ^13^C NMR spectrum of compound **2** in CDCl_3_ at 298 K.


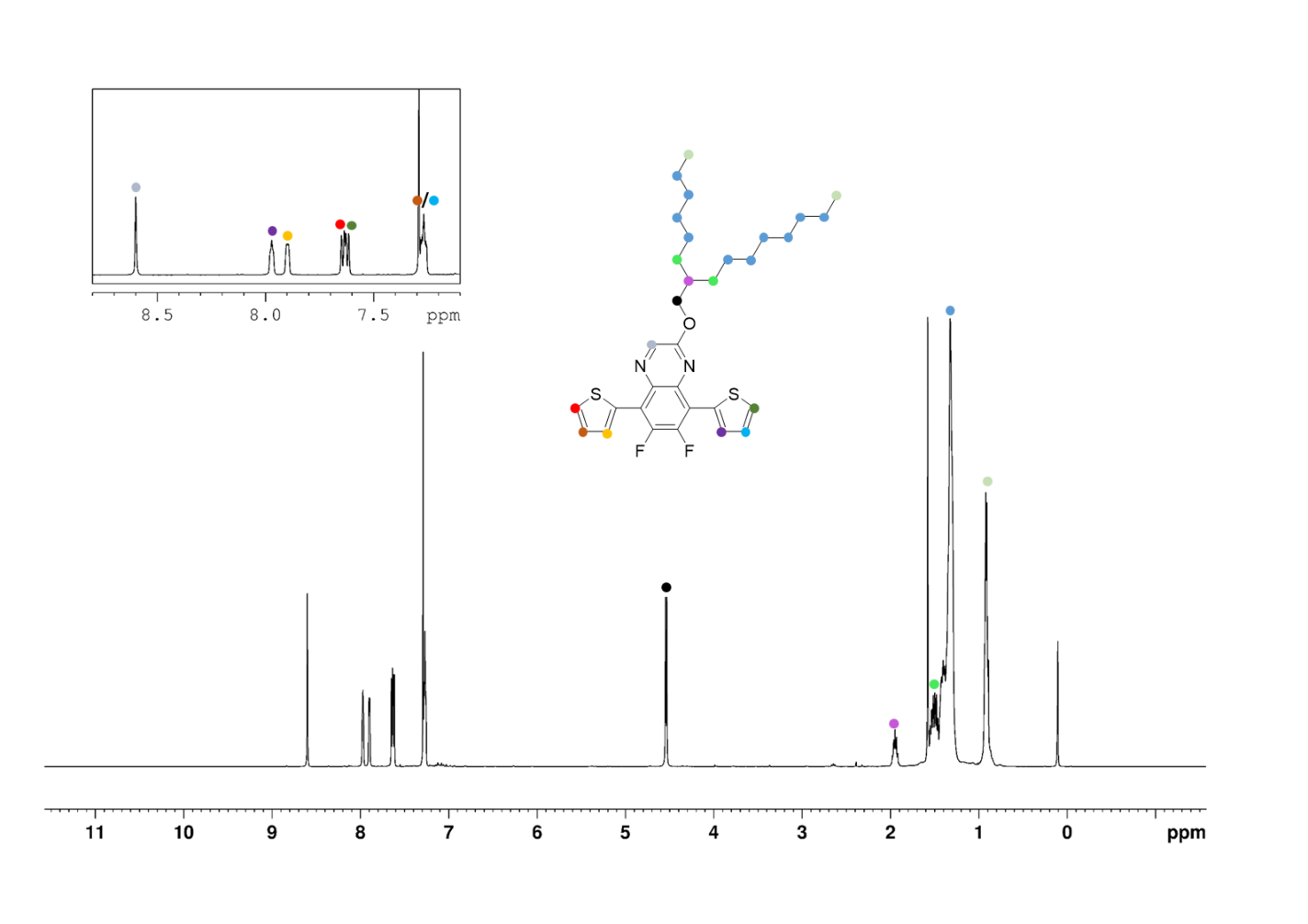


Figure S22. Total ^1^H NMR attribution for compound **2**.

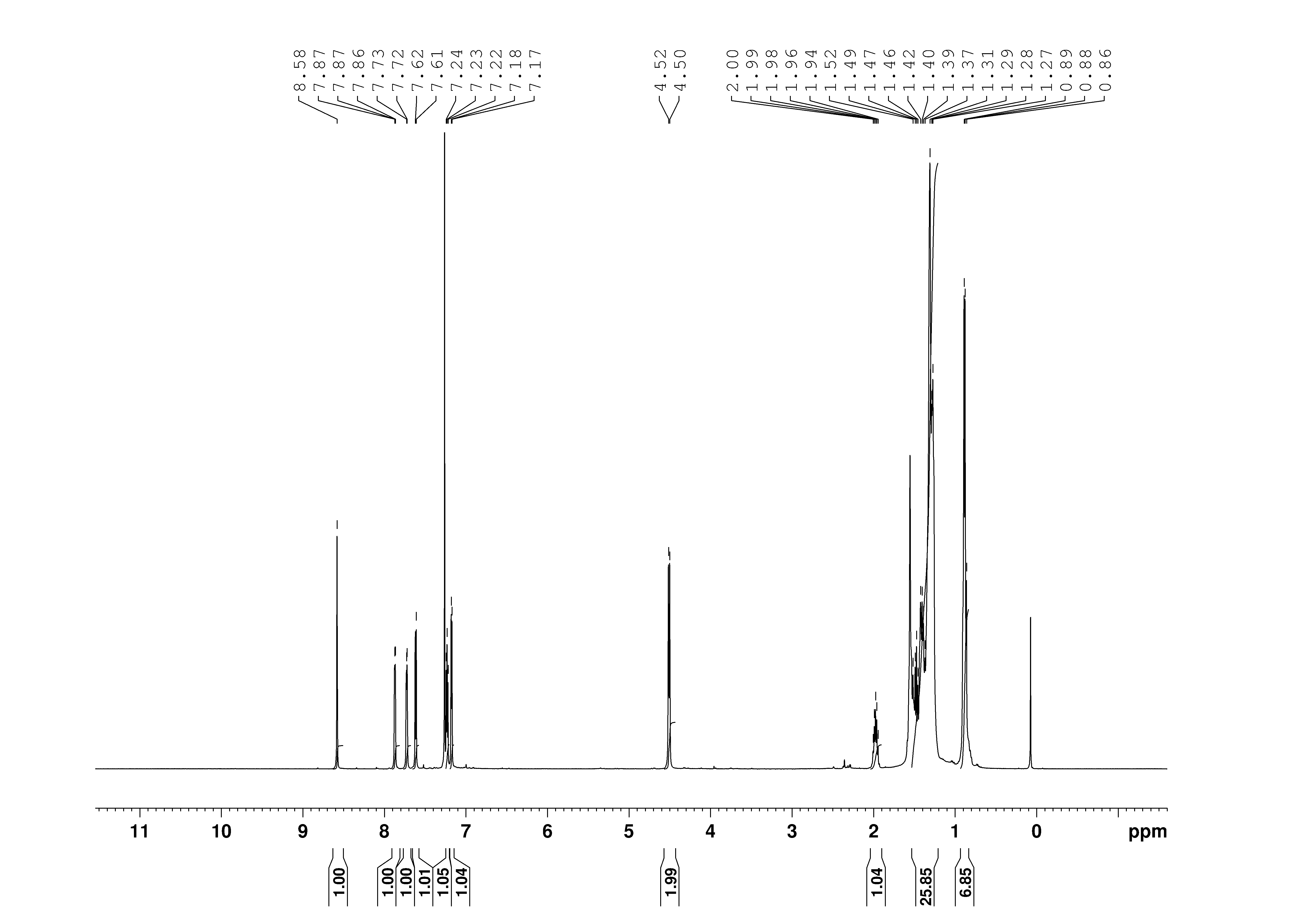


Figure S23. ^1^H NMR spectrum of compound **3** in CDCl_3_ at 298 K.


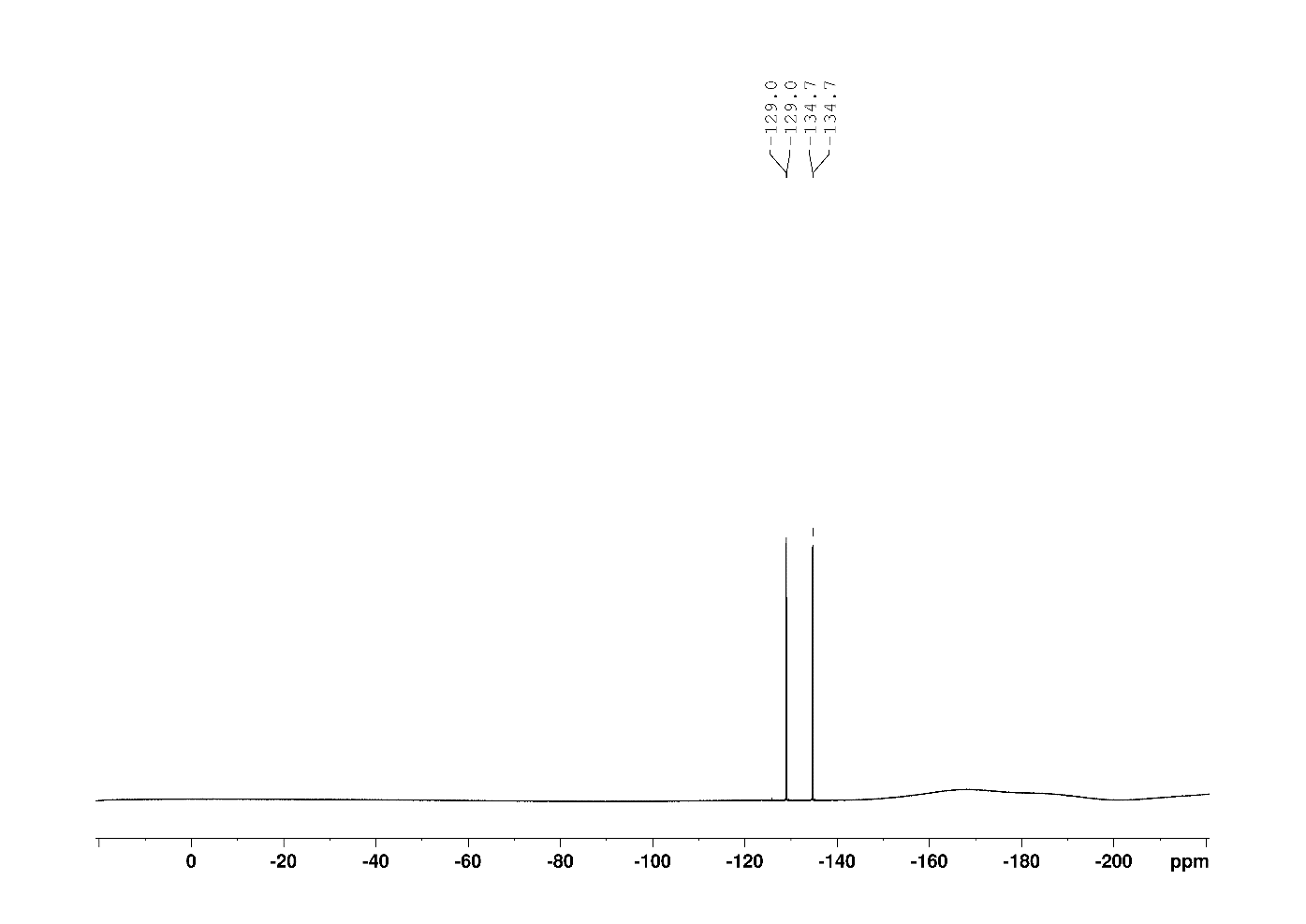


Figure S24. ^19^F NMR spectrum of compound **3** in CDCl_3_ at 298 K.


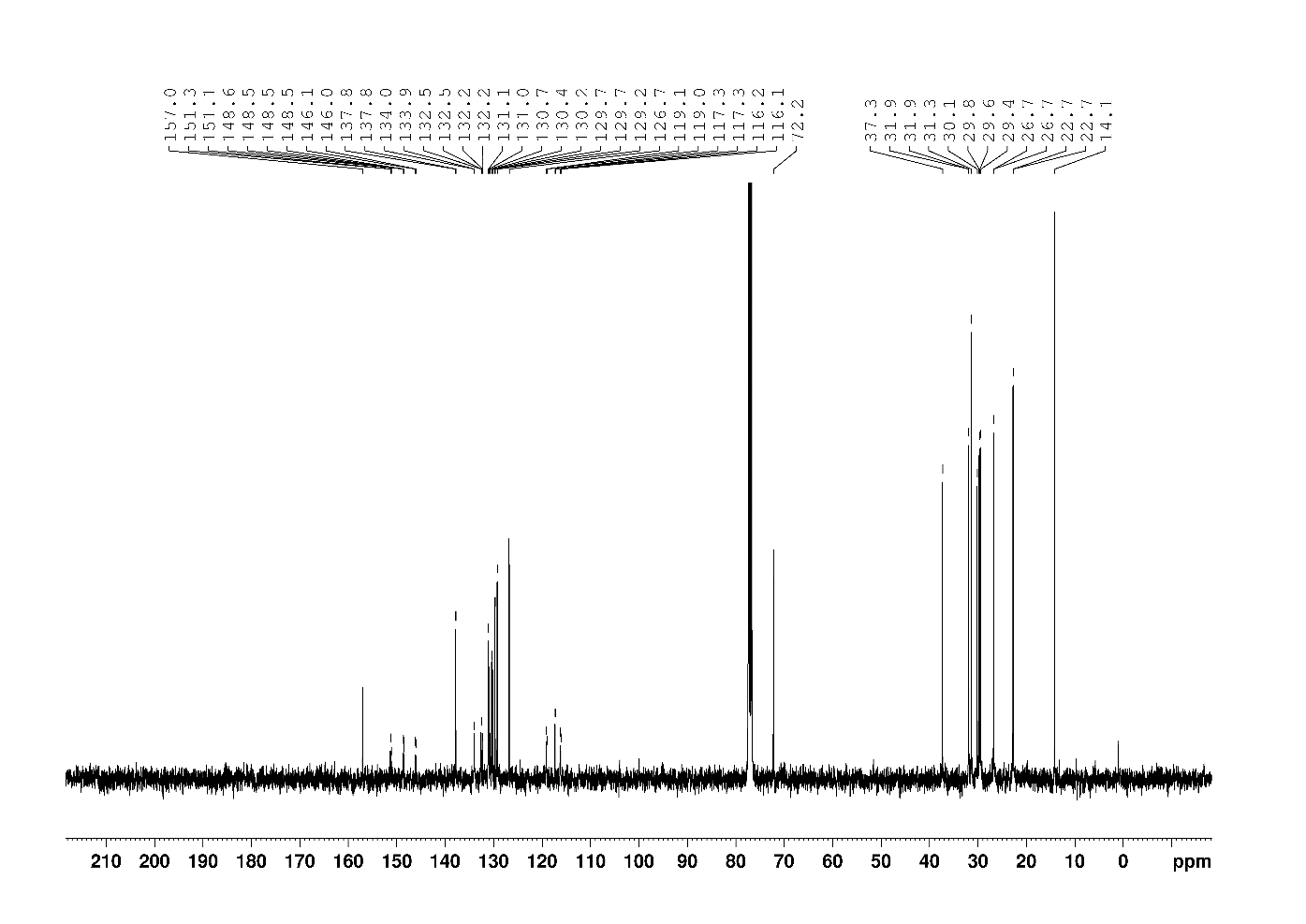


Figure S25. ^13^C NMR spectrum of compound **3** in CDCl_3_ at 298 K.


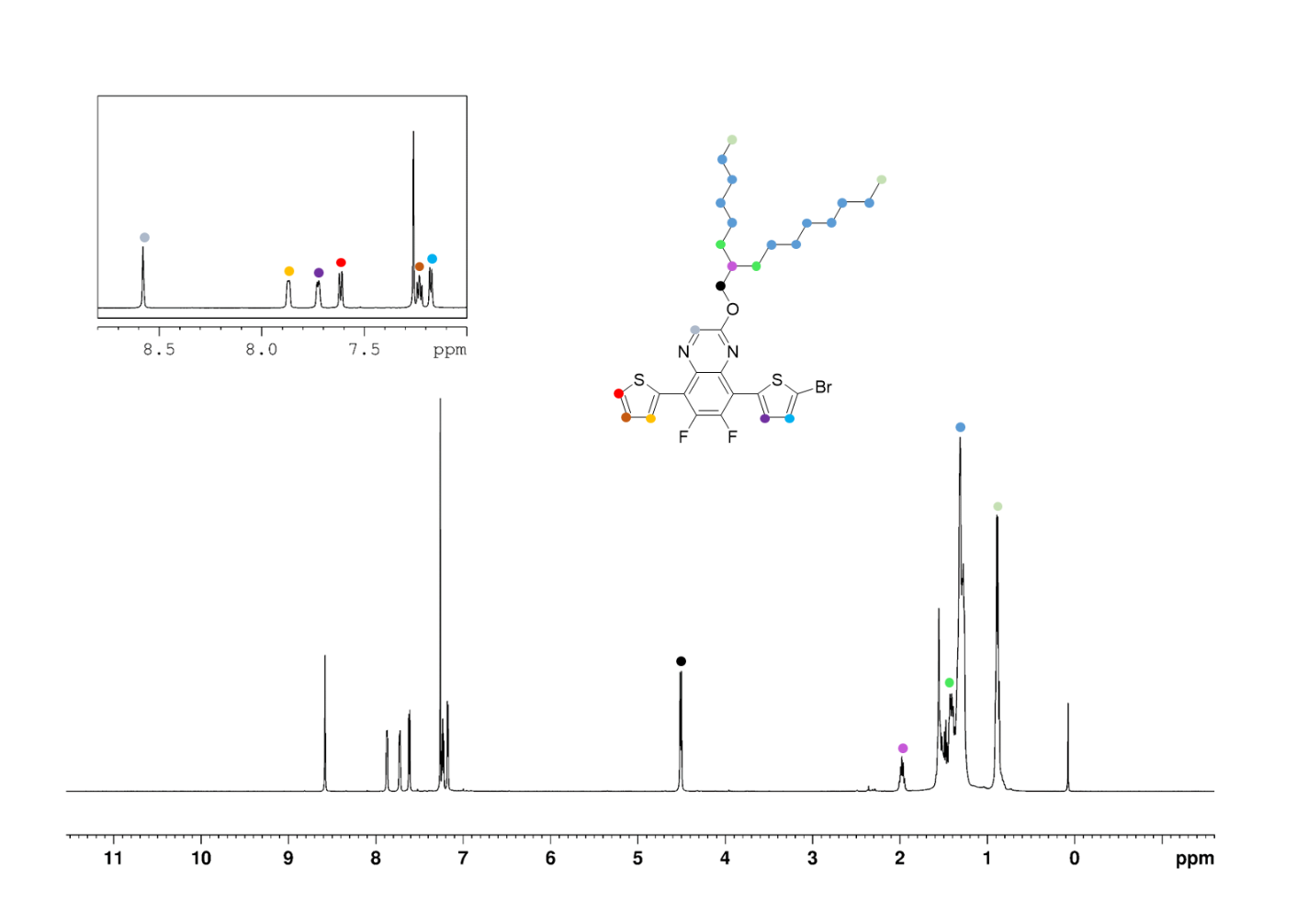


Figure S26. Total ^1^H NMR attribution for compound **3**.


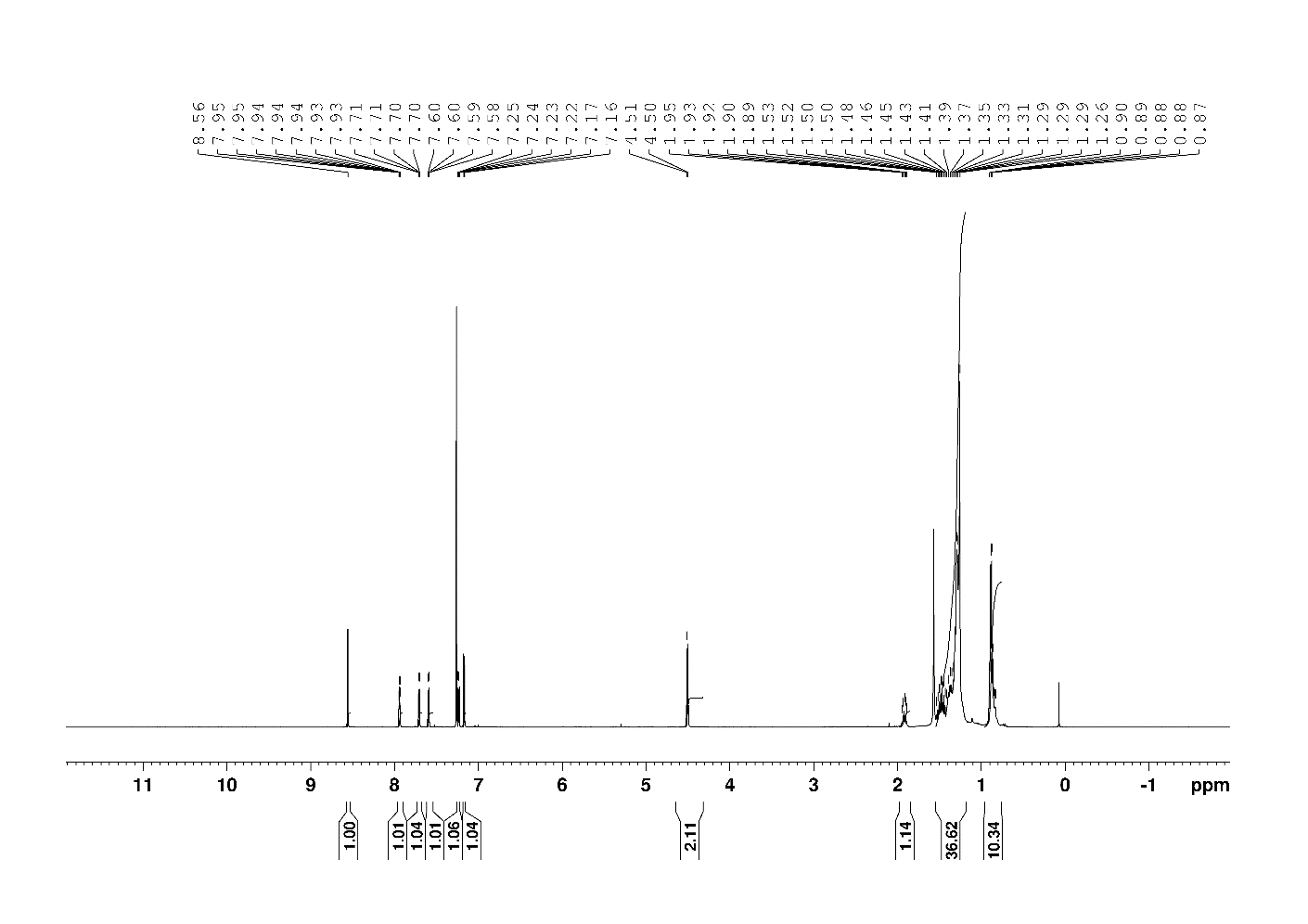


Figure S27. ^1^H NMR spectrum of compound **3’** in CDCl_3_ at 298 K.


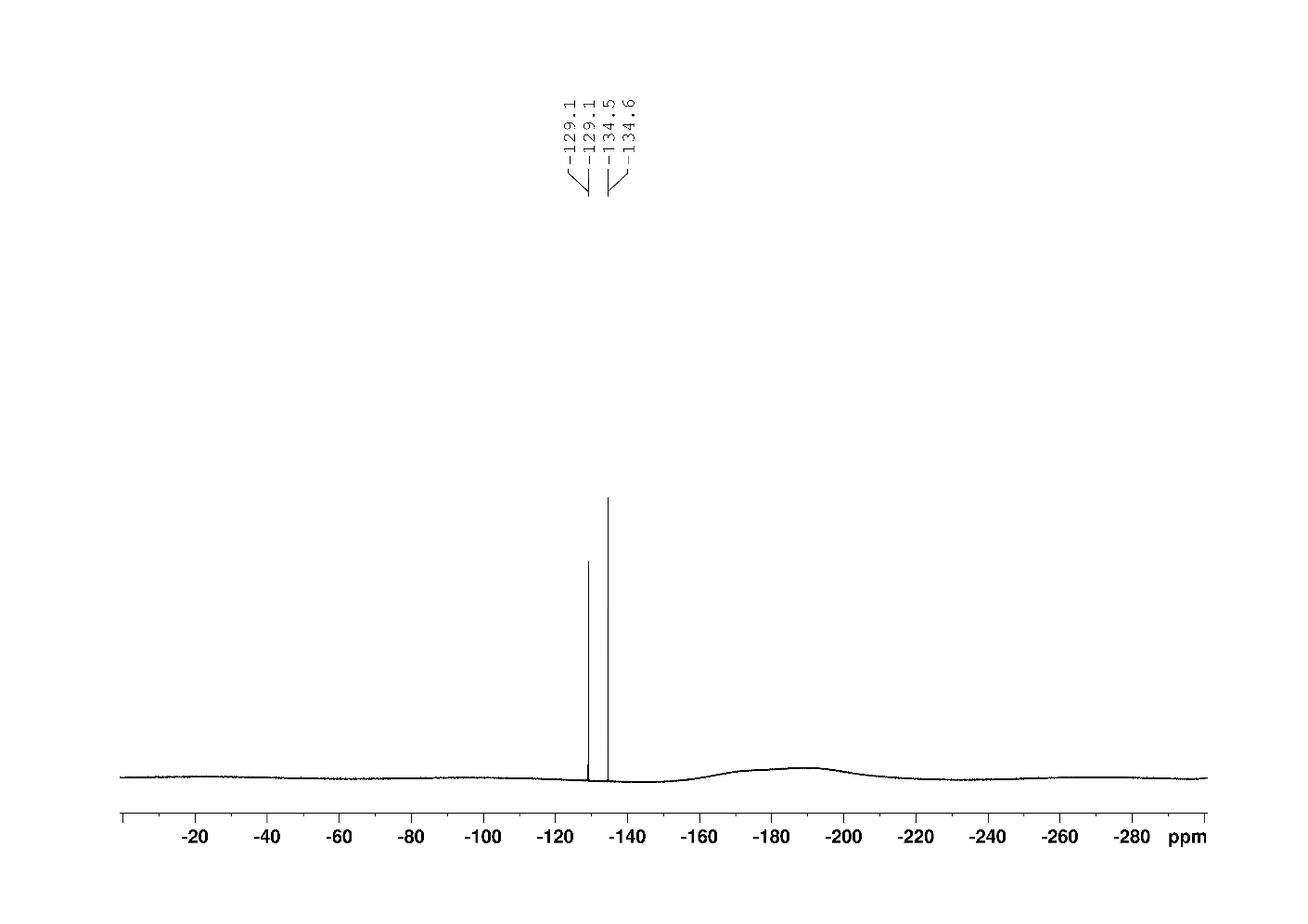


Figure S28. ^19^F NMR spectrum of compound **3’** in CDCl_3_ at 298 K.


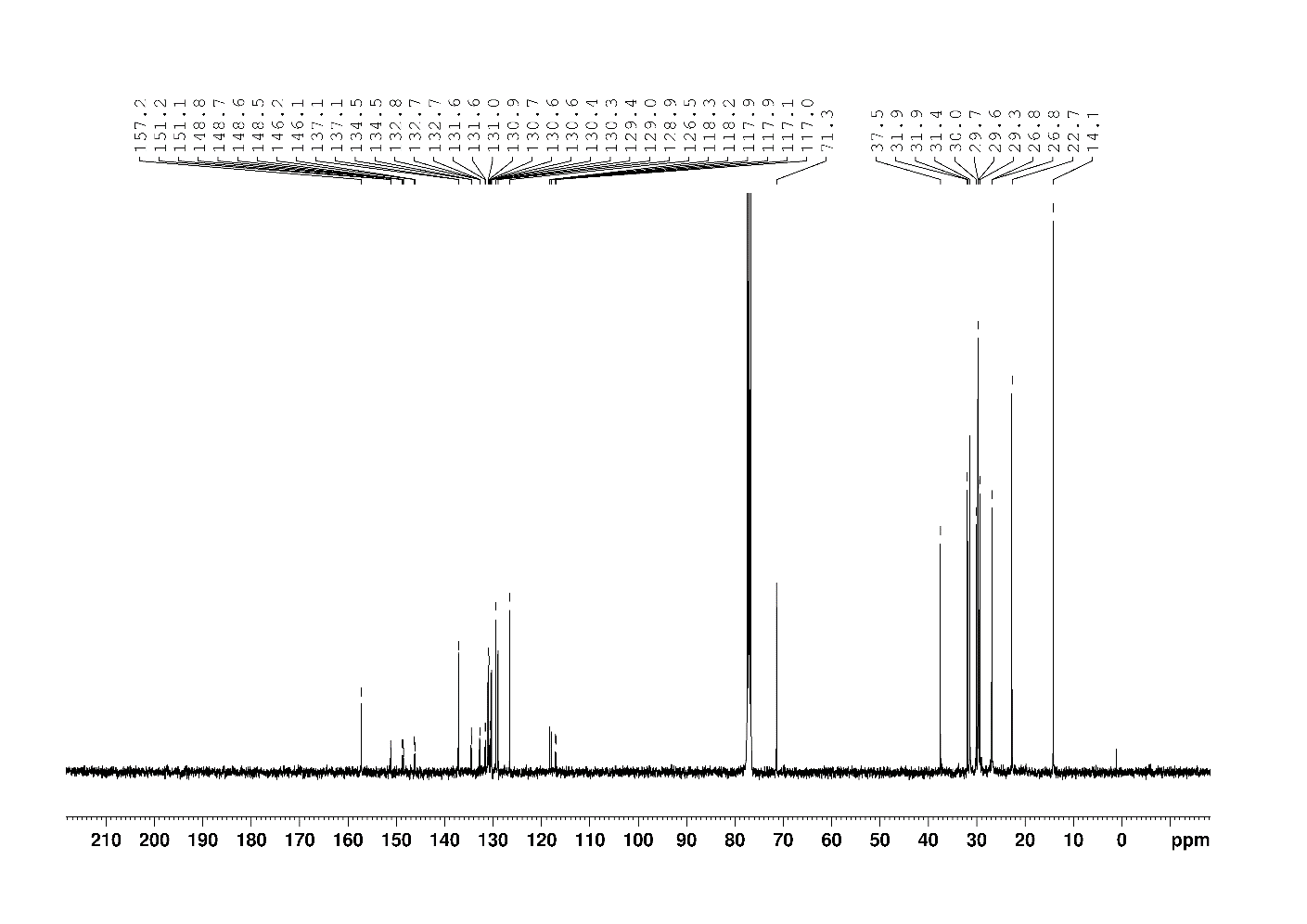


Figure S29. ^13^C NMR spectrum of compound **3’** in CDCl_3_ at 298 K.


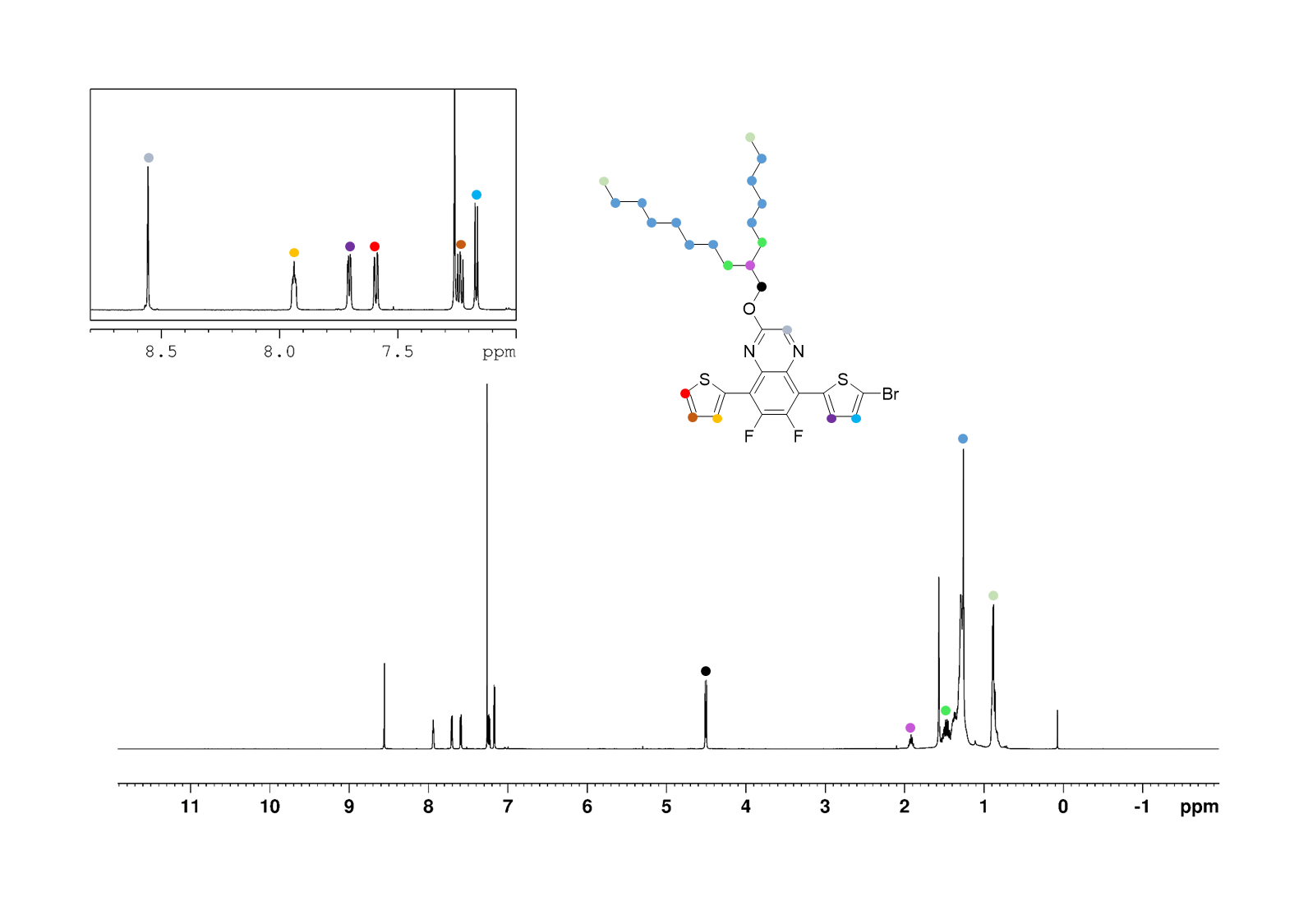


Figure S30. Total ^1^H NMR attribution for compound **3’**.

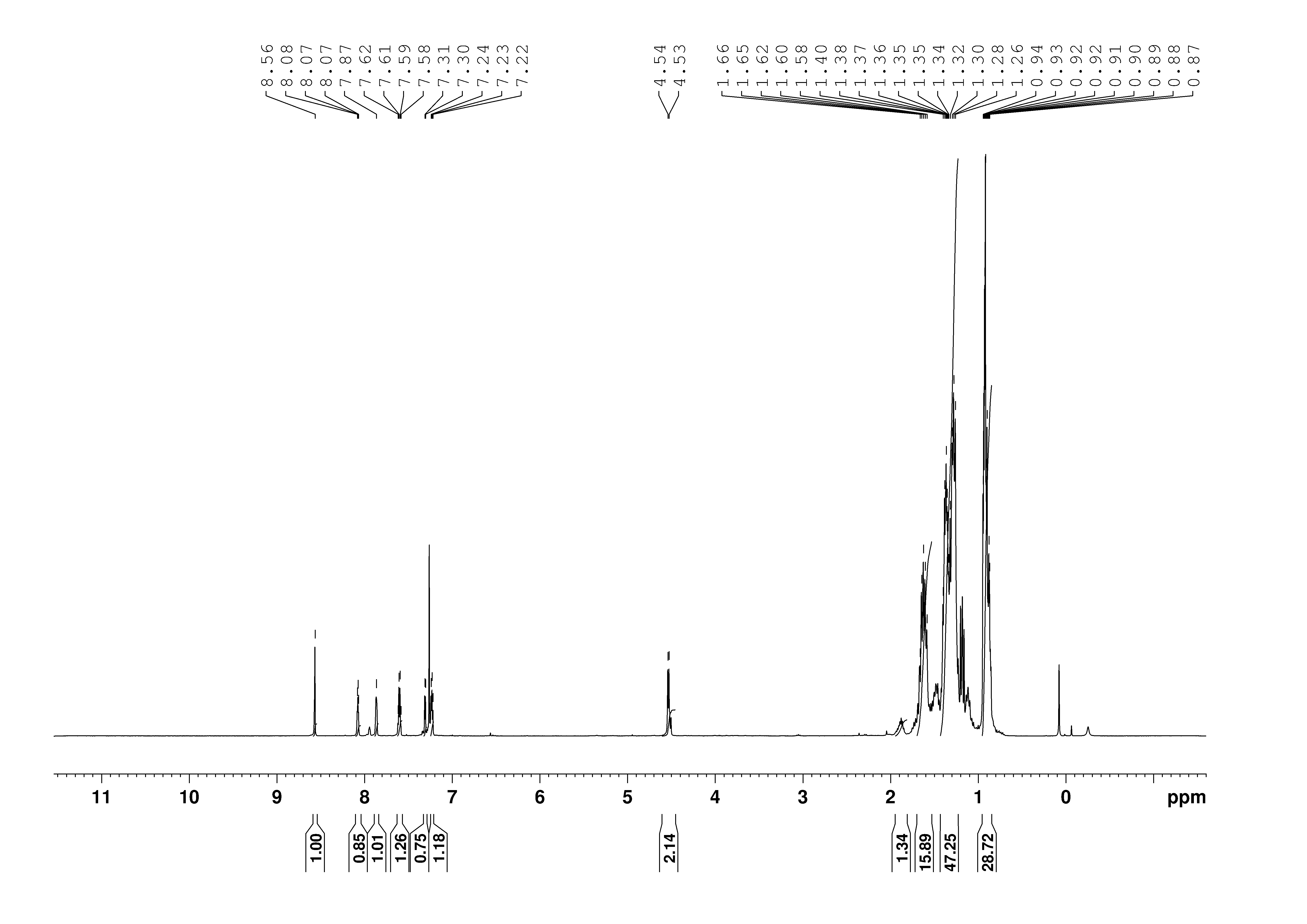


Figure S31. ^1^H NMR spectrum of compound **4** in CDCl_3_ at 298 K.


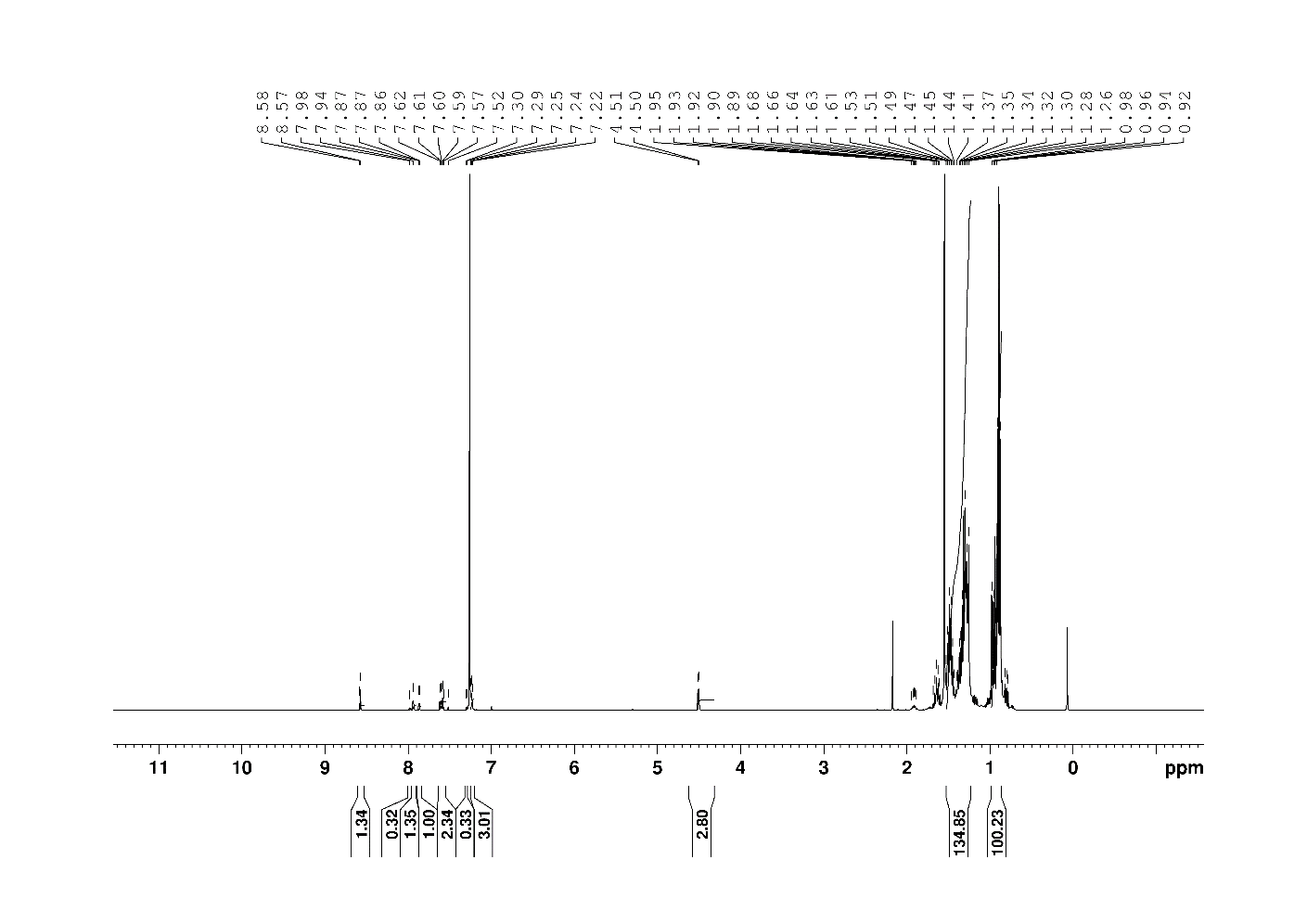

 Figure S32. ^1^H NMR spectrum of compound **4’** in CDCl_3_ at 298 K.

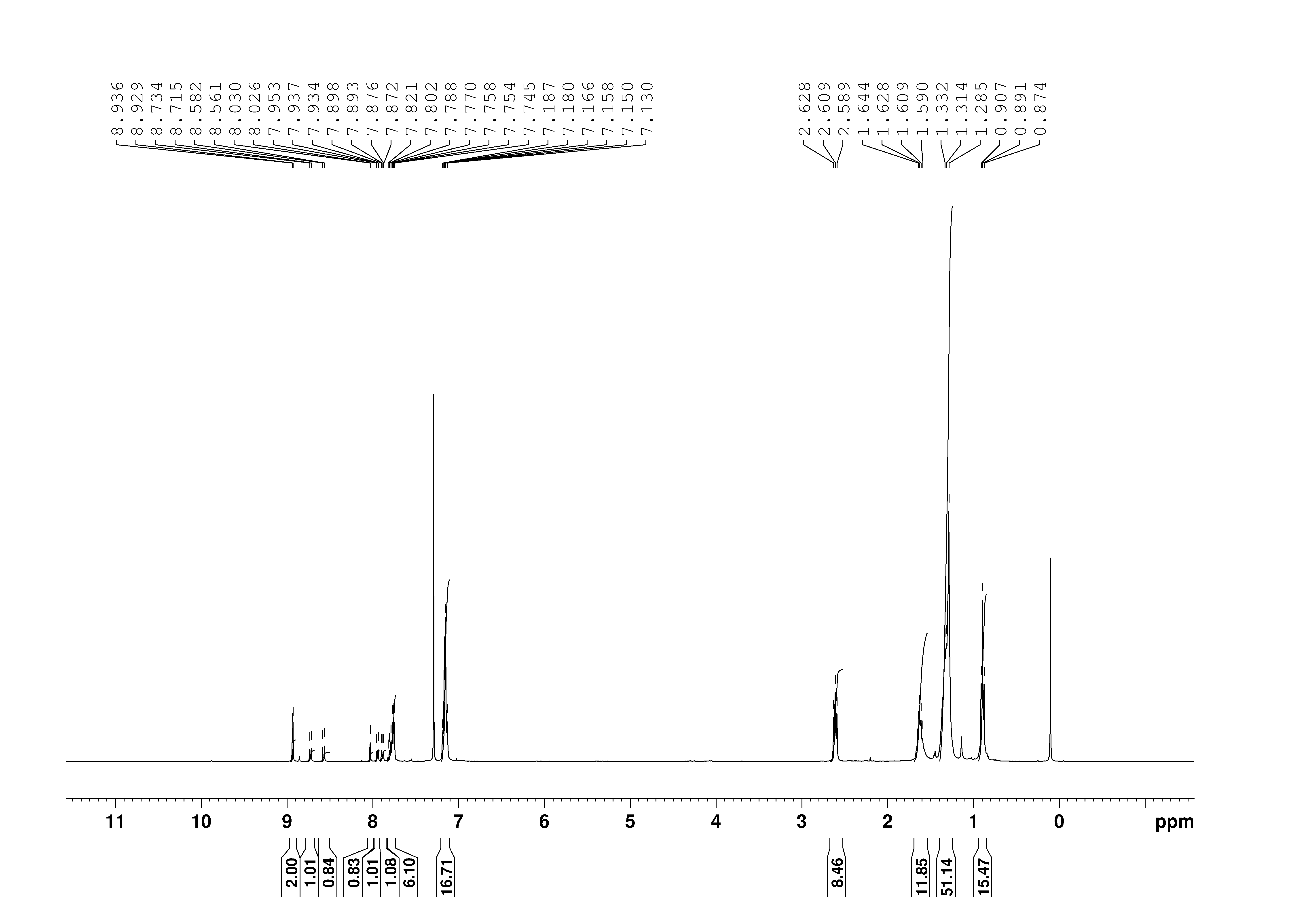


Figure S33. ^1^H NMR spectrum of compound **6** in CDCl_3_ at 298 K.


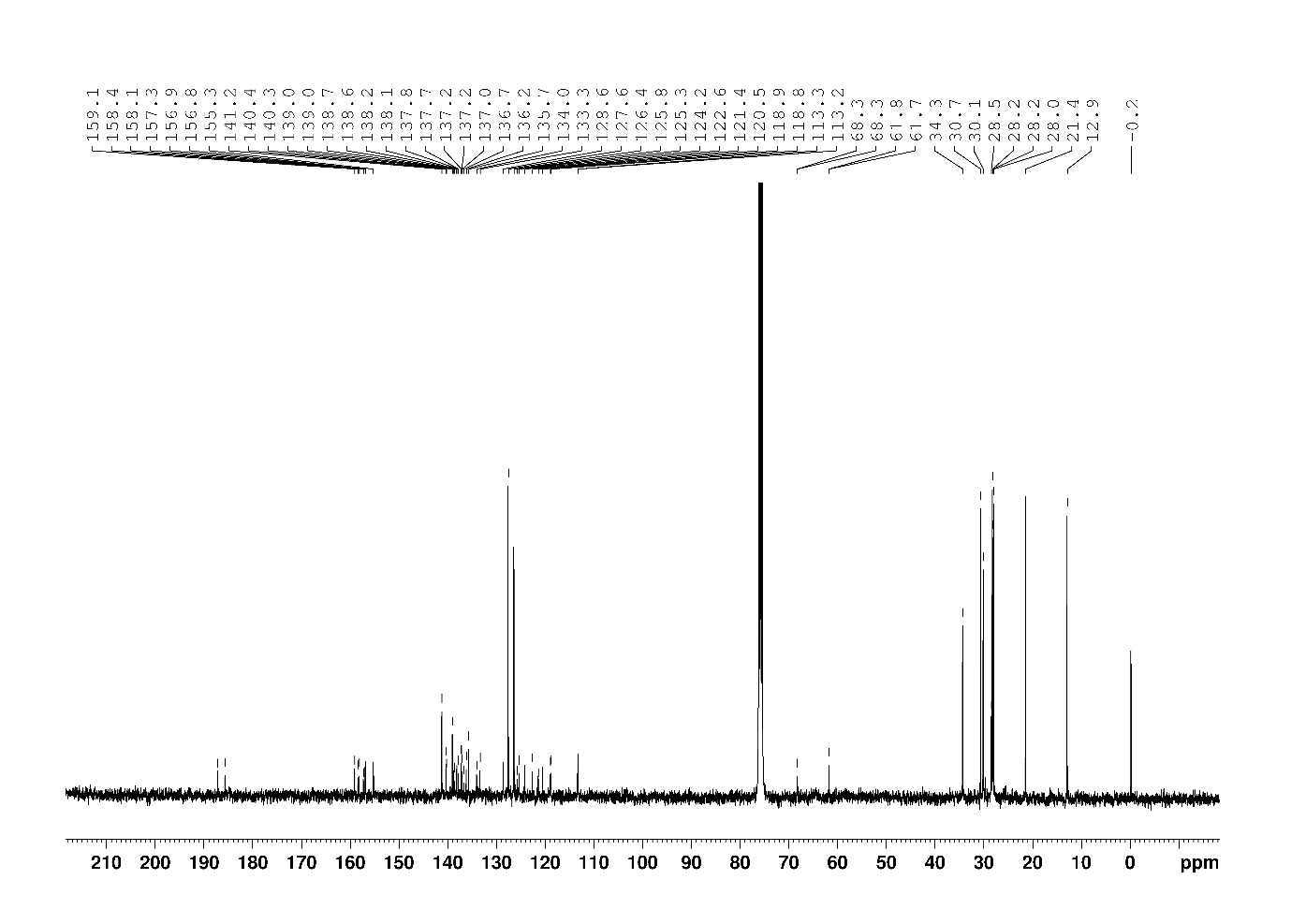


Figure S34. ^13^C NMR spectrum of compound **6** in CDCl_3_ at 298 K.


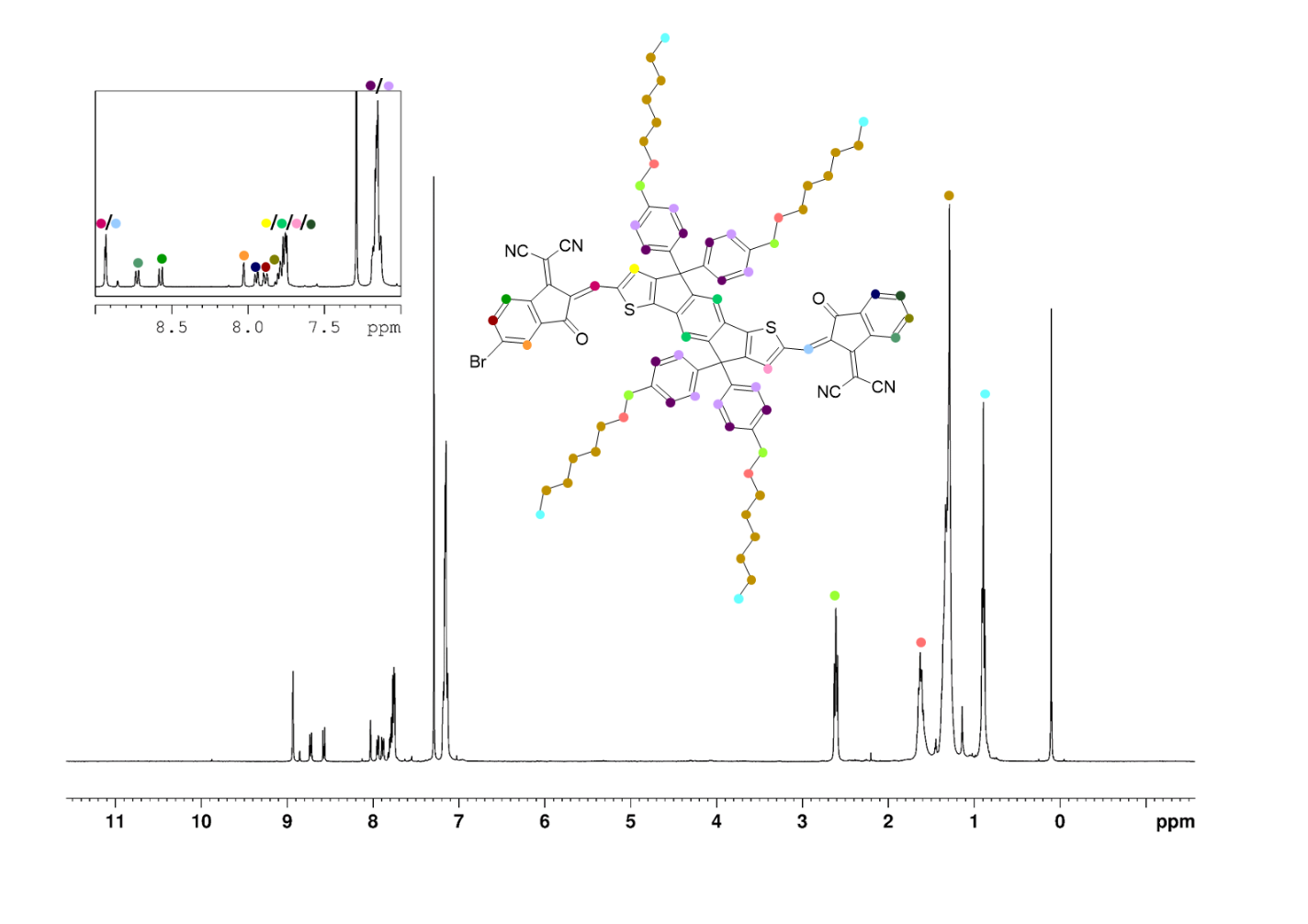


Figure S35. Total ^1^H NMR attribution for compound **6**.


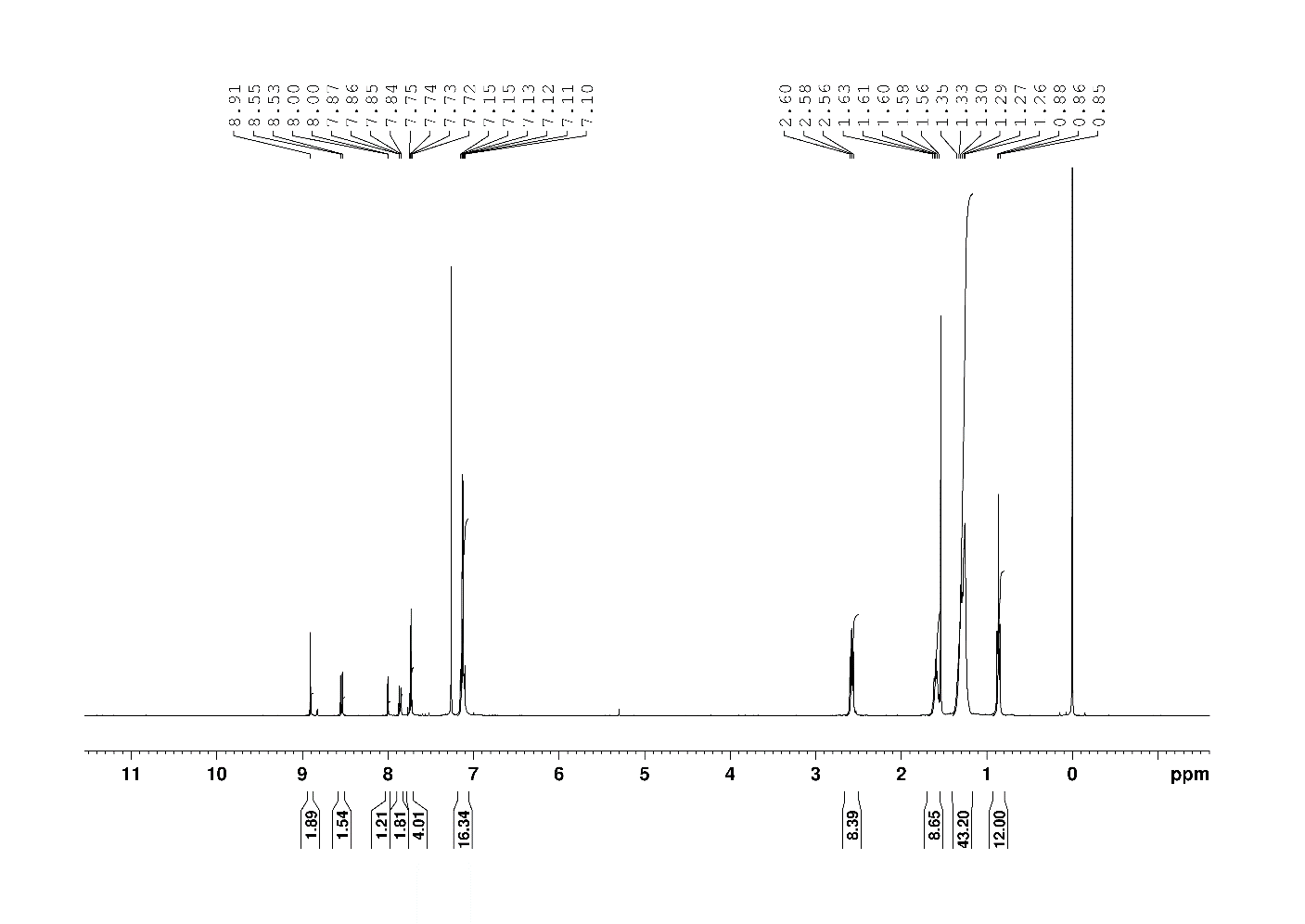


Figure S36. ^1^H NMR spectrum of compound **IDTe-2Br** in CDCl_3_ at 298 K.


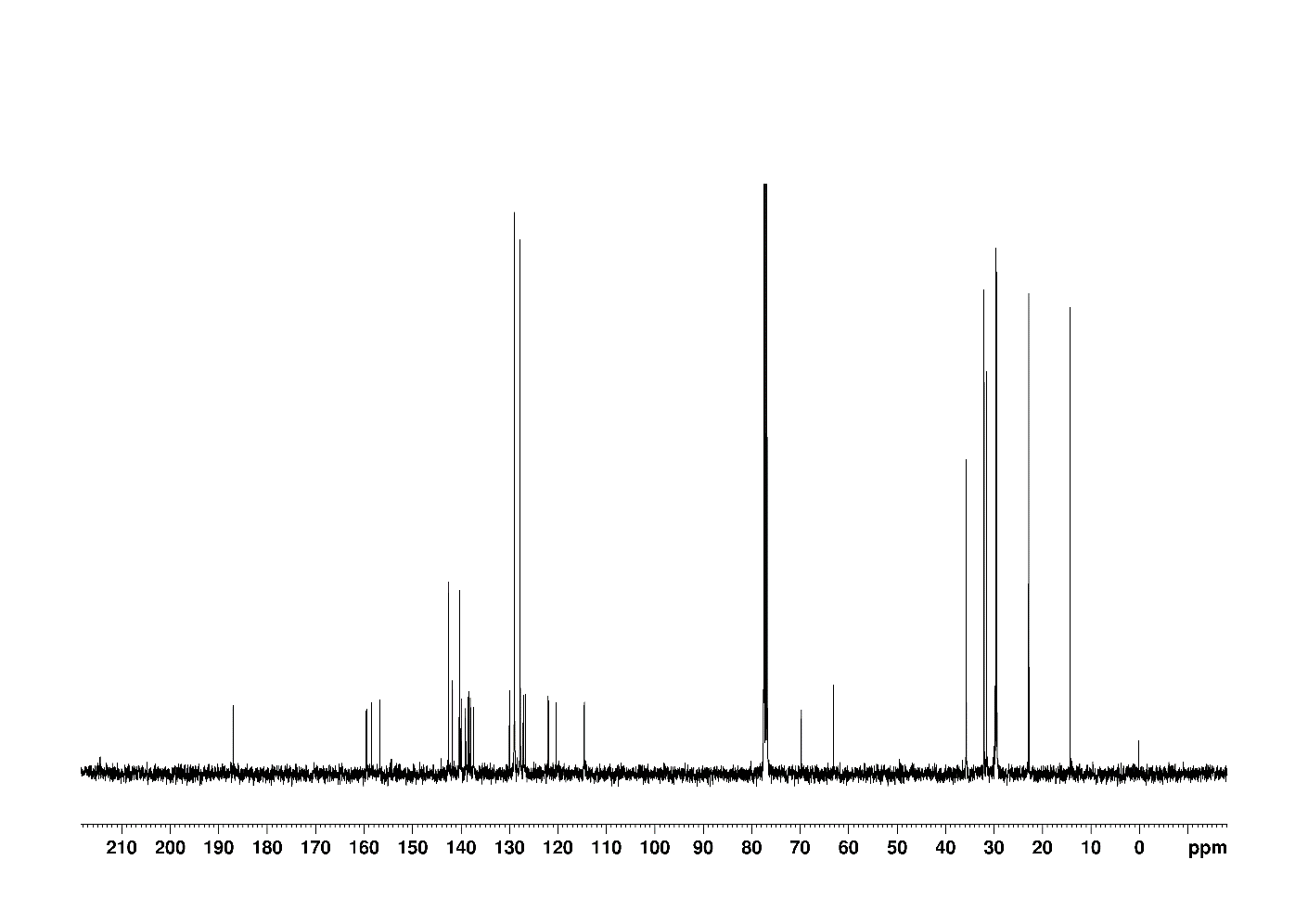


Figure S37. ^13^C NMR spectrum of compound **IDTe-2Br** in CDCl_3_ at 298 K.


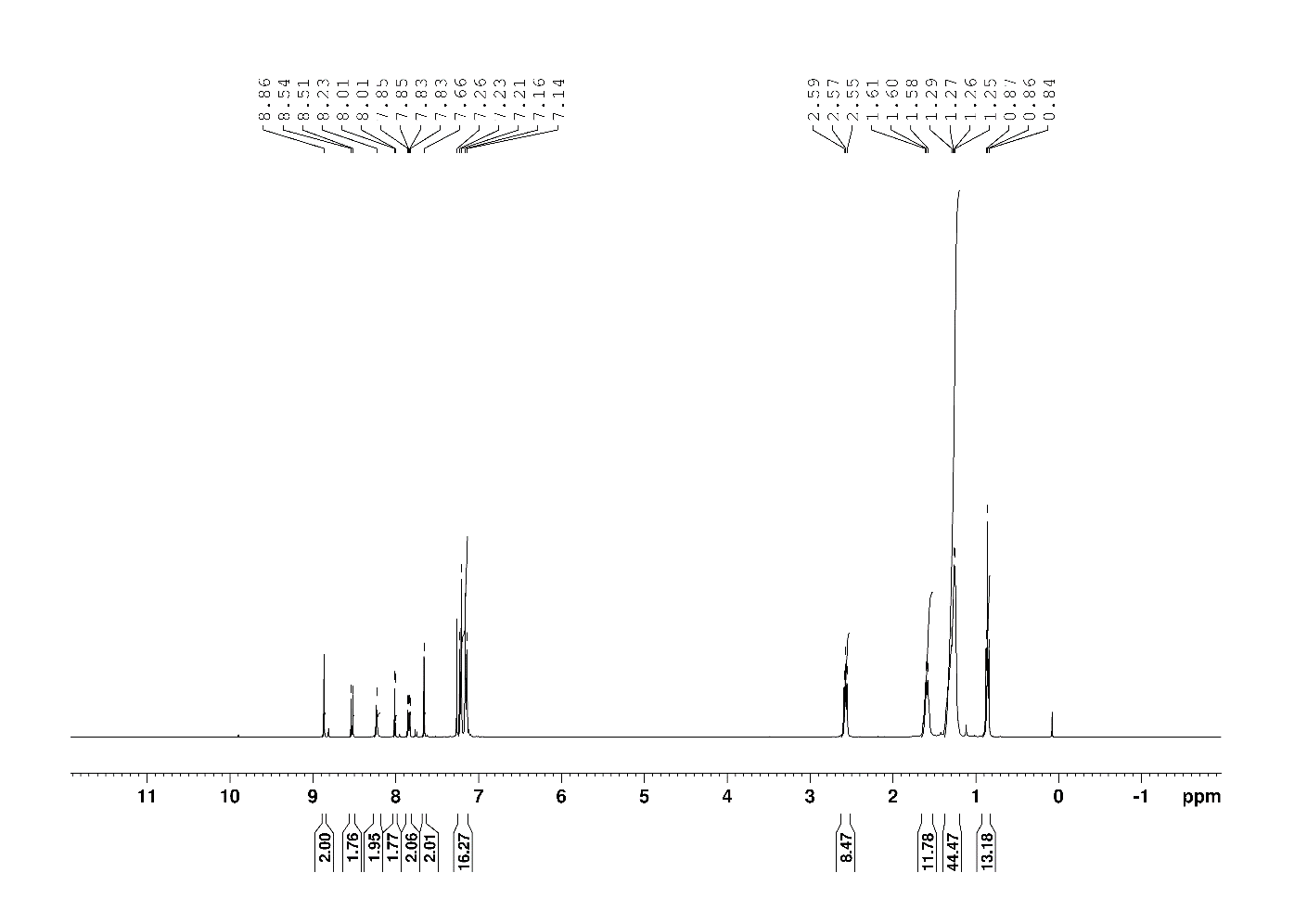


Figure S38. ^1^H NMR spectrum of compound **ITIC-2Br** in CDCl_3_ at 298 K.


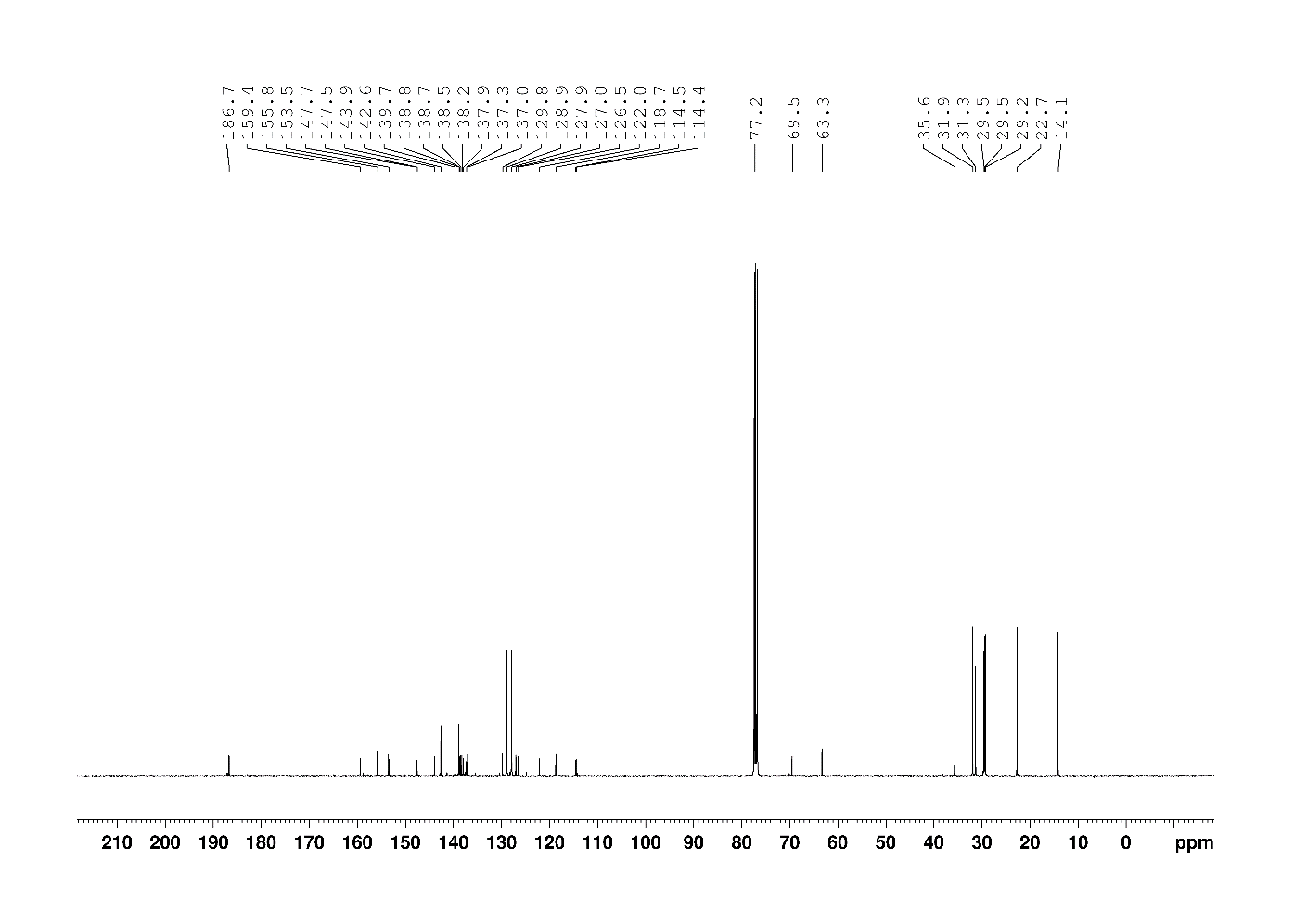


Figure S39. ^13^C NMR spectrum of compound **ITIC-2Br** in CDCl_3_ at 298 K.


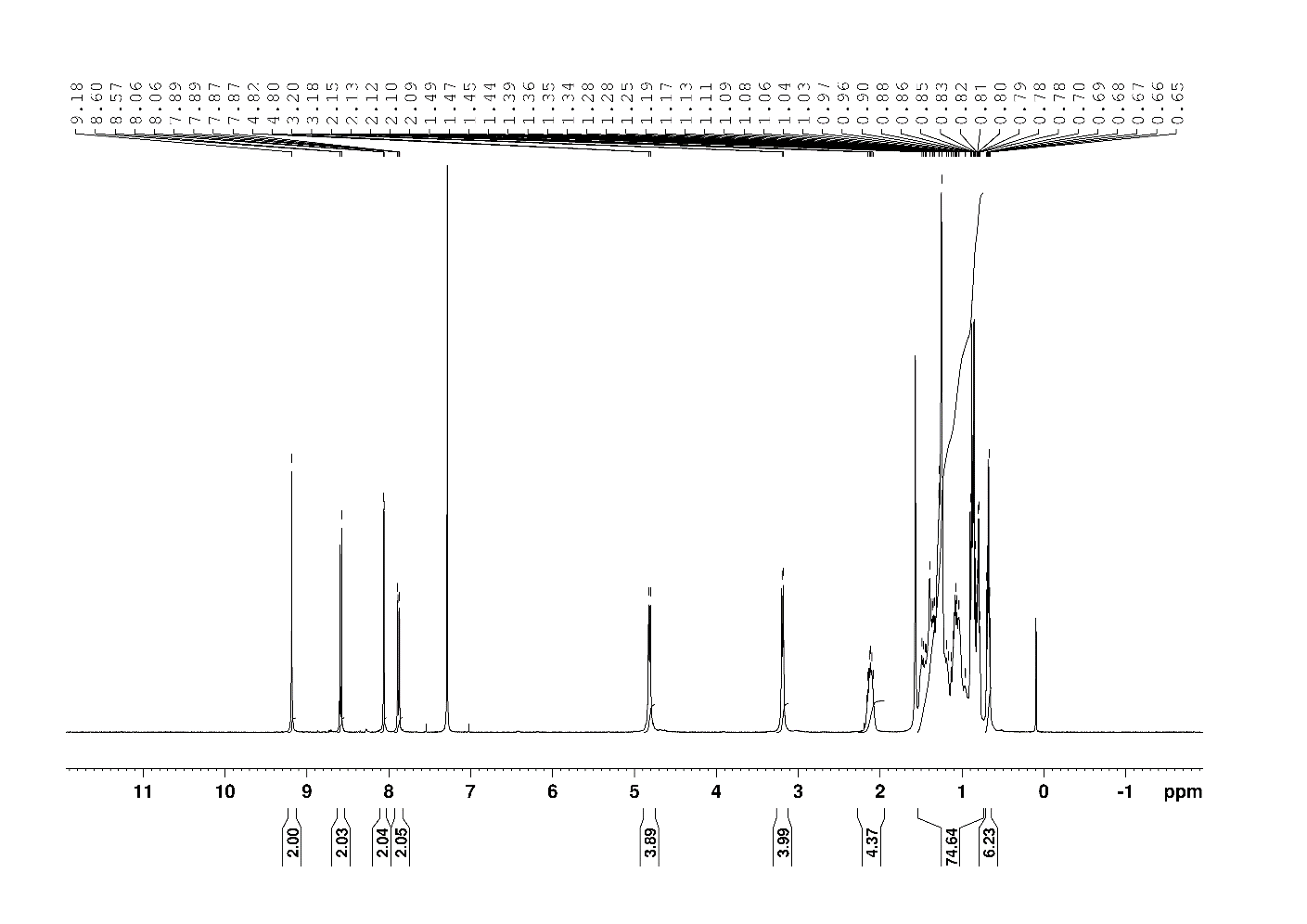


Figure S40. ^1^H NMR spectrum of compound **L8-BO-2Br** in CDCl_3_ at 298 K.


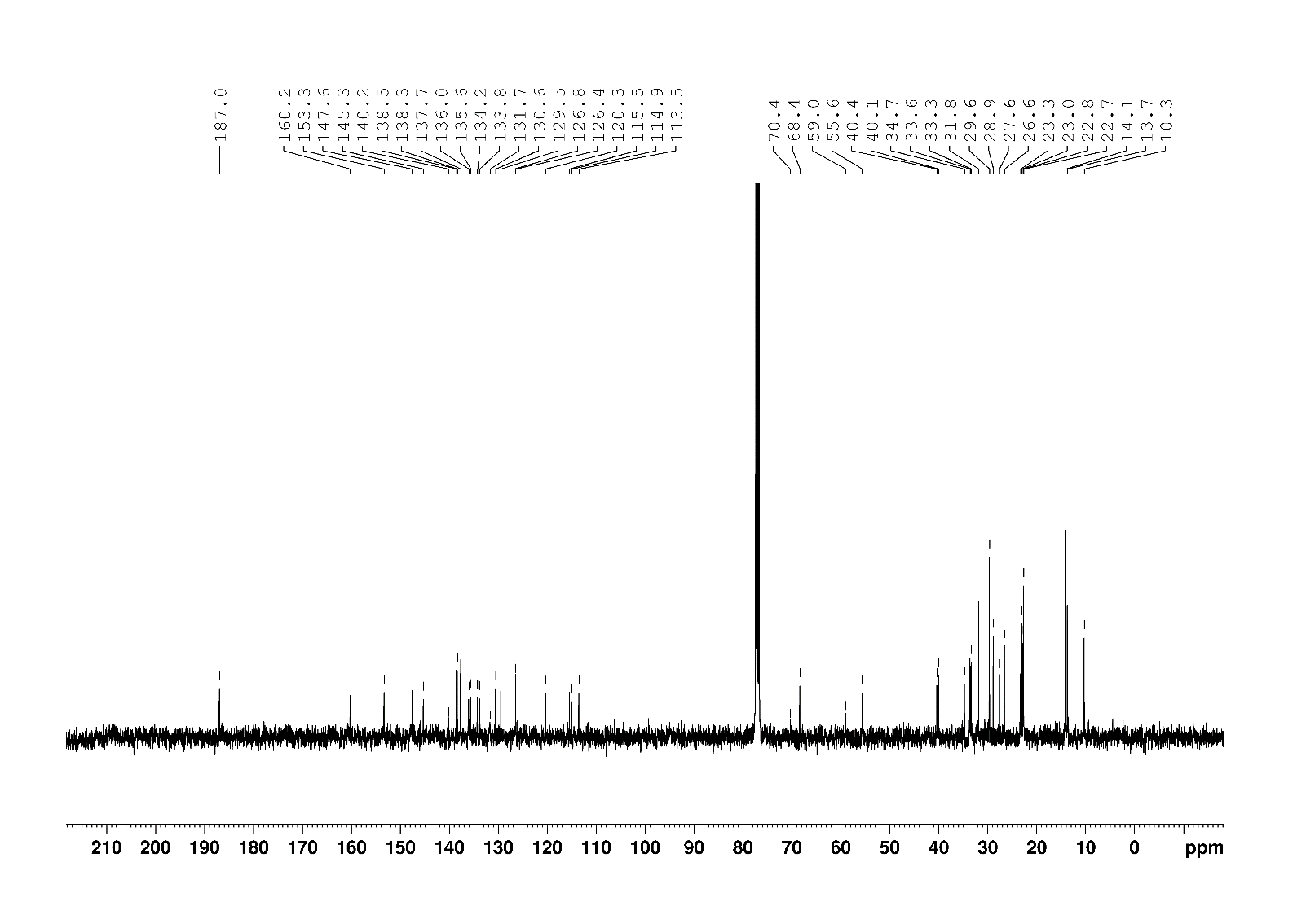


Figure S41. ^13^C NMR spectrum of compound **L8-BO-2Br** in CDCl_3_ at 298 K.


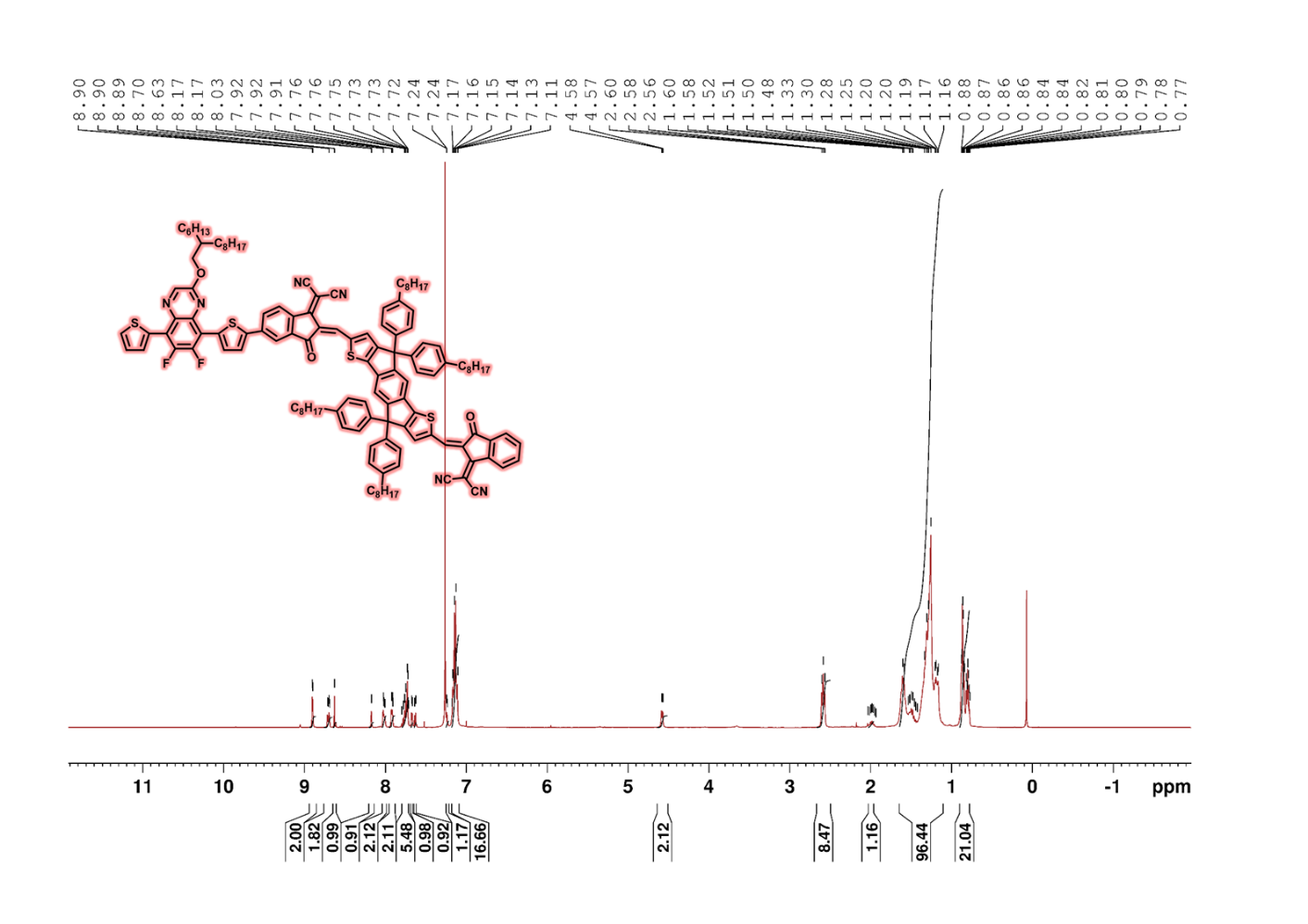


Figure S42. ^1^H NMR spectrum of compound **AJC1** in CDCl_3_ at 298 K.


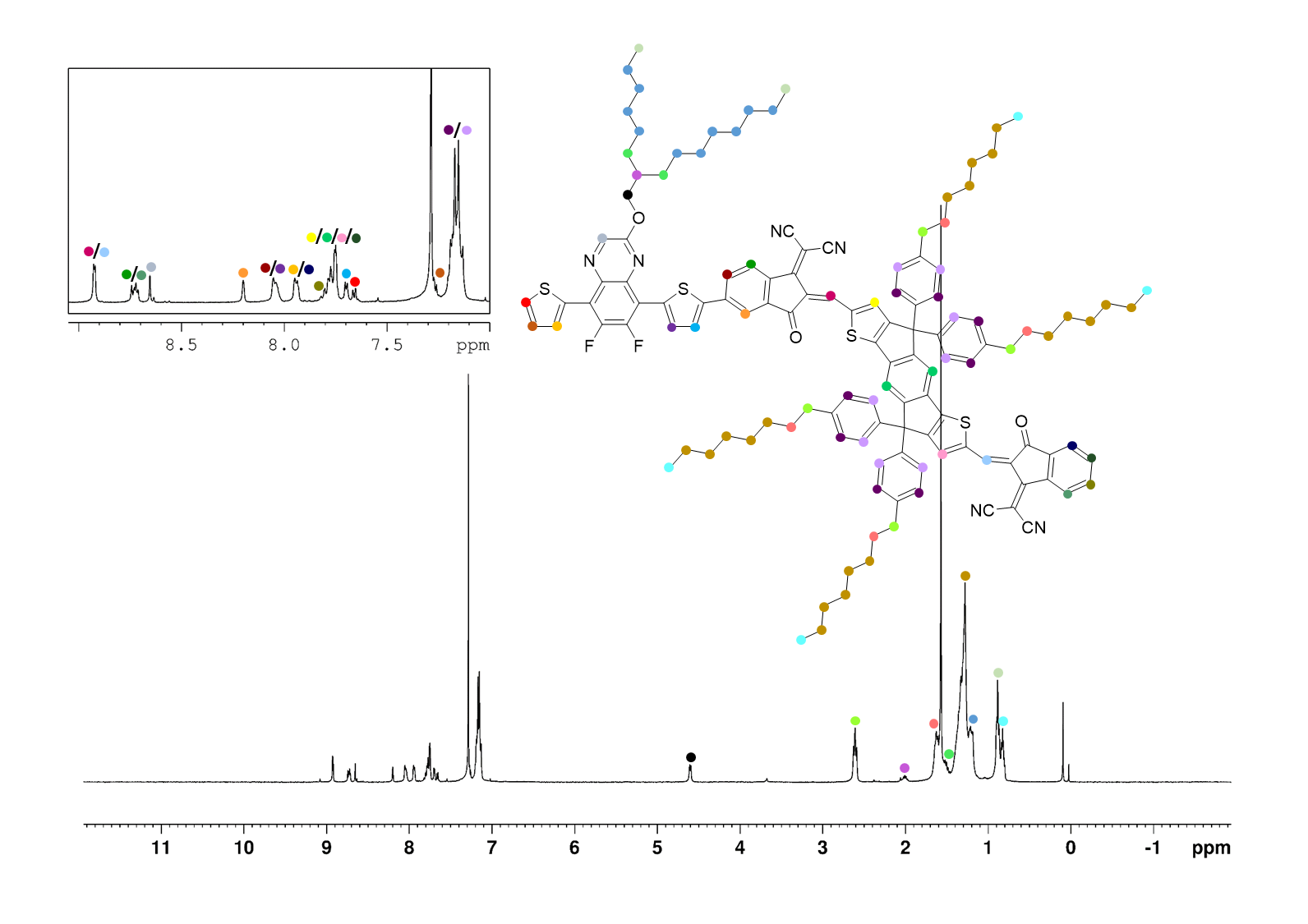


Figure S43. Total ^1^H NMR attribution for **AJC1** in CDCl_3_ at 298 K.


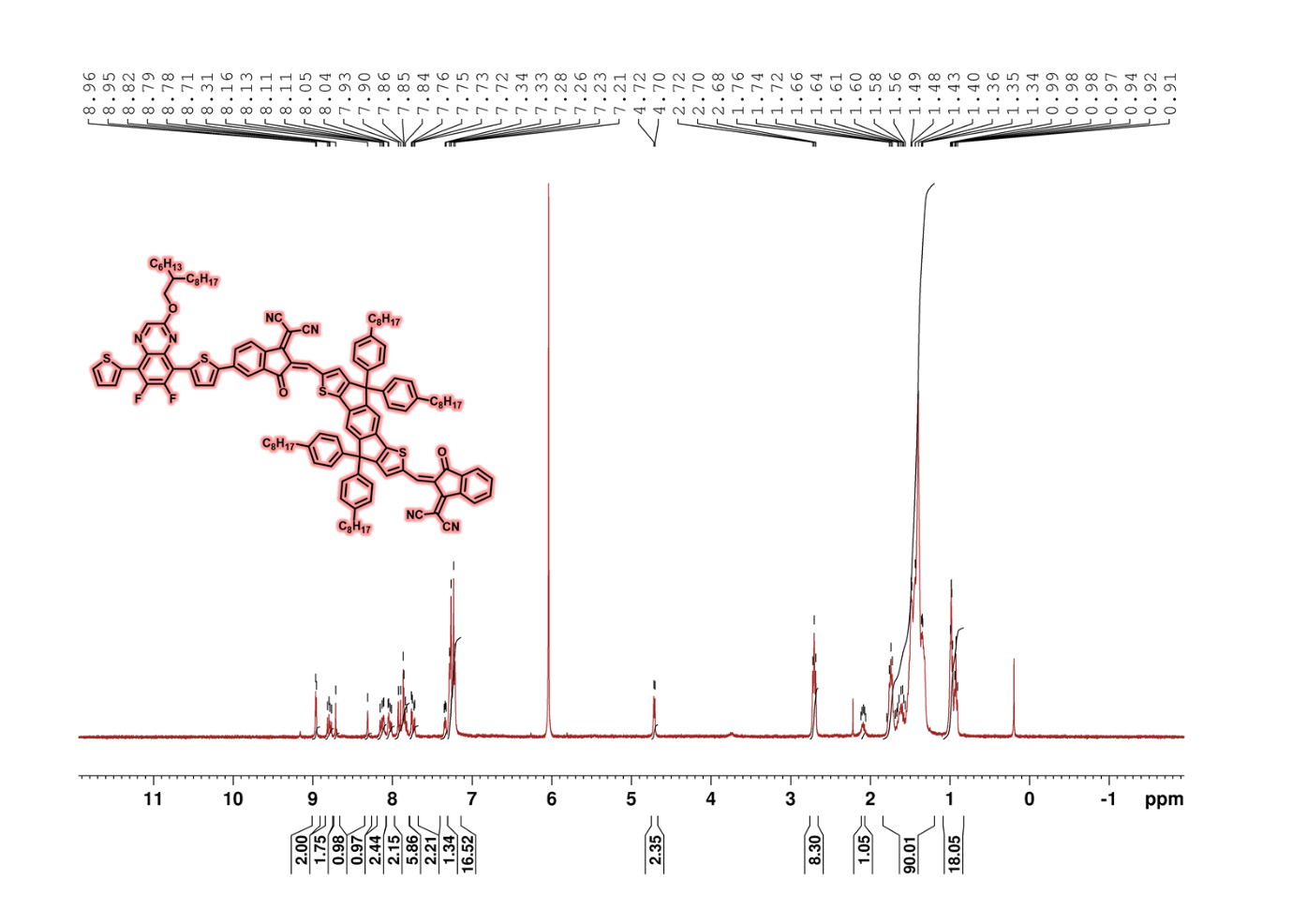


Figure S44. ^1^H NMR spectrum of **AJC1** in C_2_D_2_Cl_4_ at 393 K.


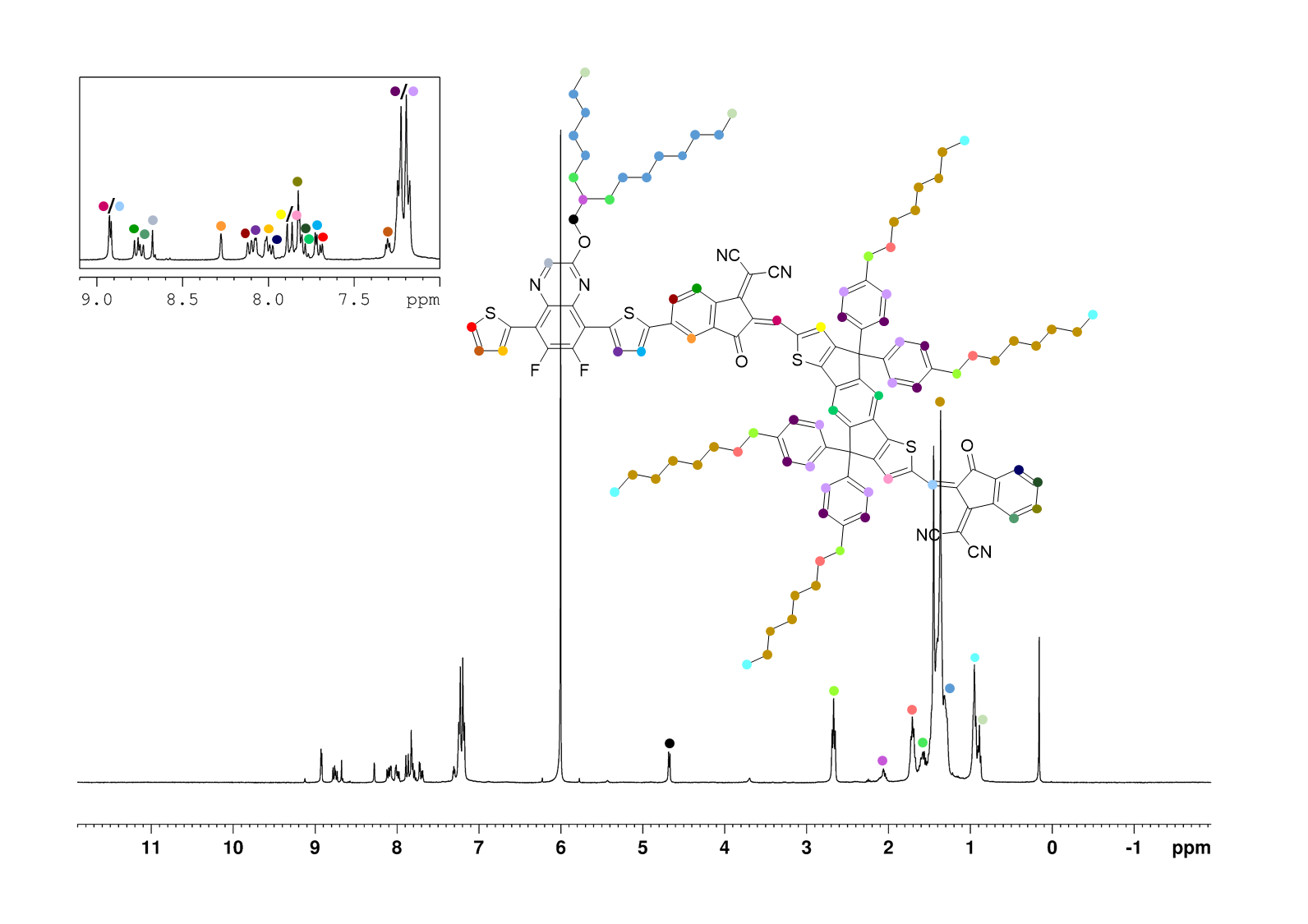


Figure S45. Total ^1^H NMR attribution for **AJC1** in C_2_D_2_Cl_4_ at 393 K.


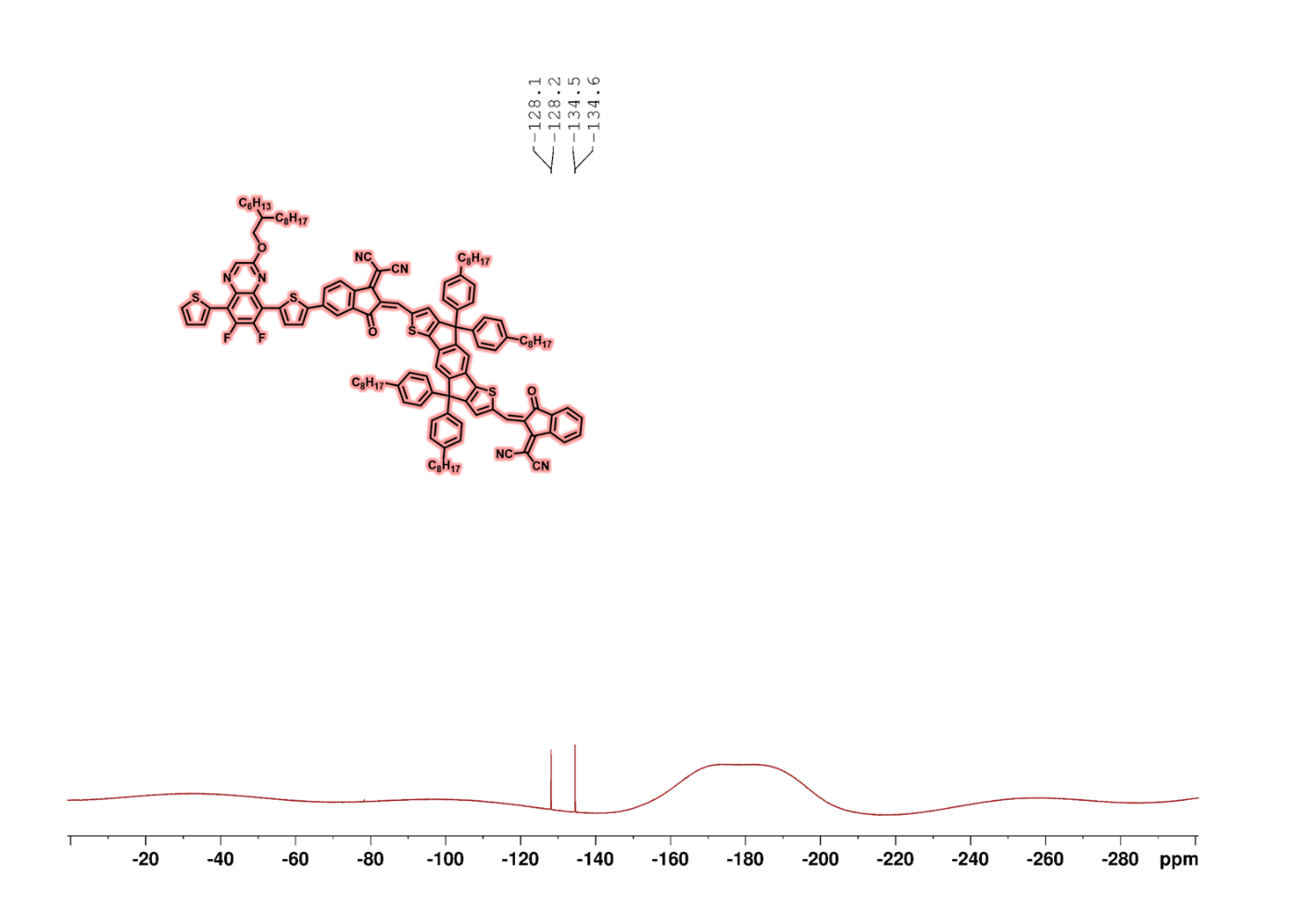


Figure S46. ^19^F NMR spectrum of compound **AJC1** in CDCl_3_ at 298 K.


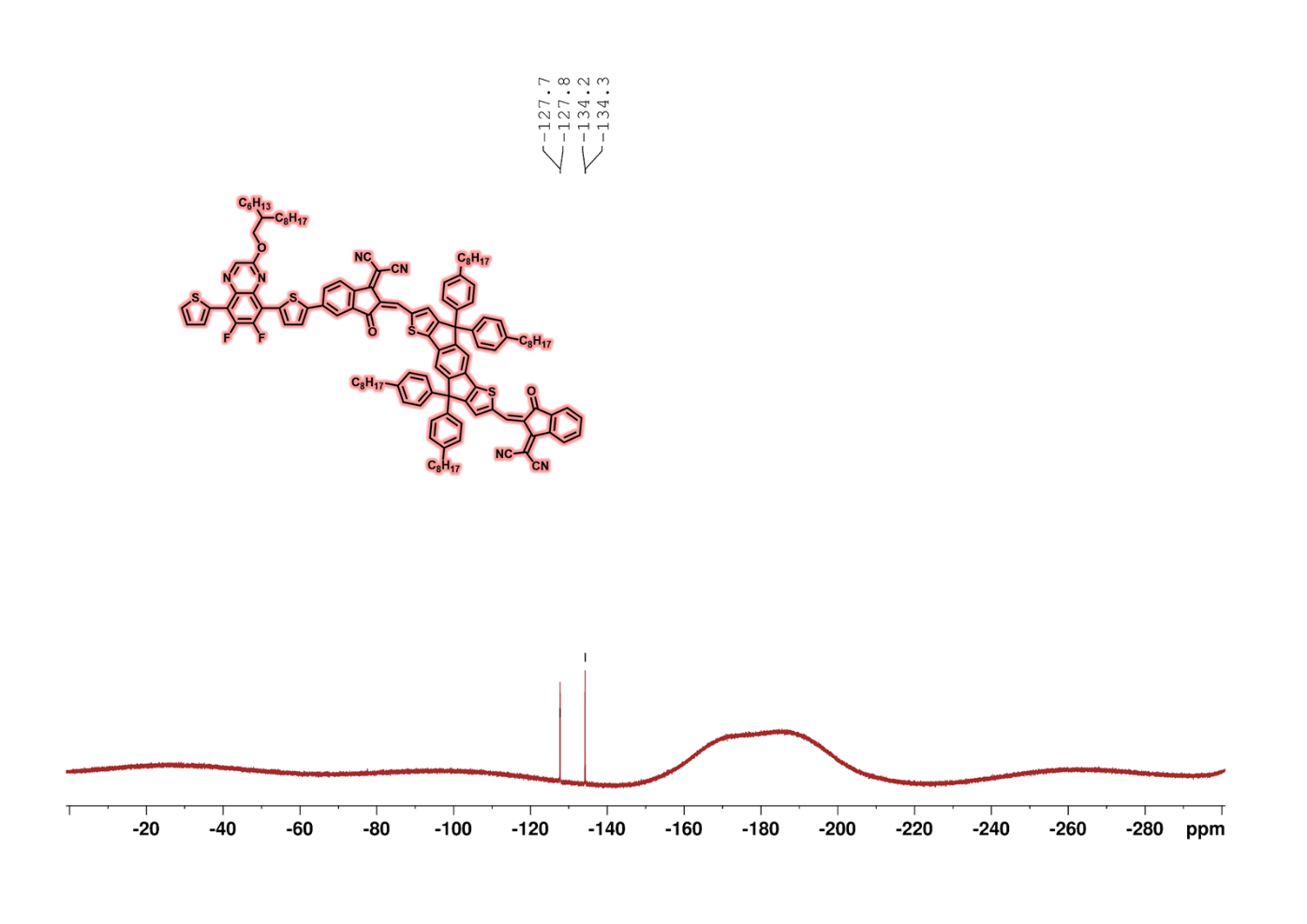


Figure S47. ^19^F NMR spectrum of **AJC1** in C_2_D_2_Cl_4_ at 393 K.


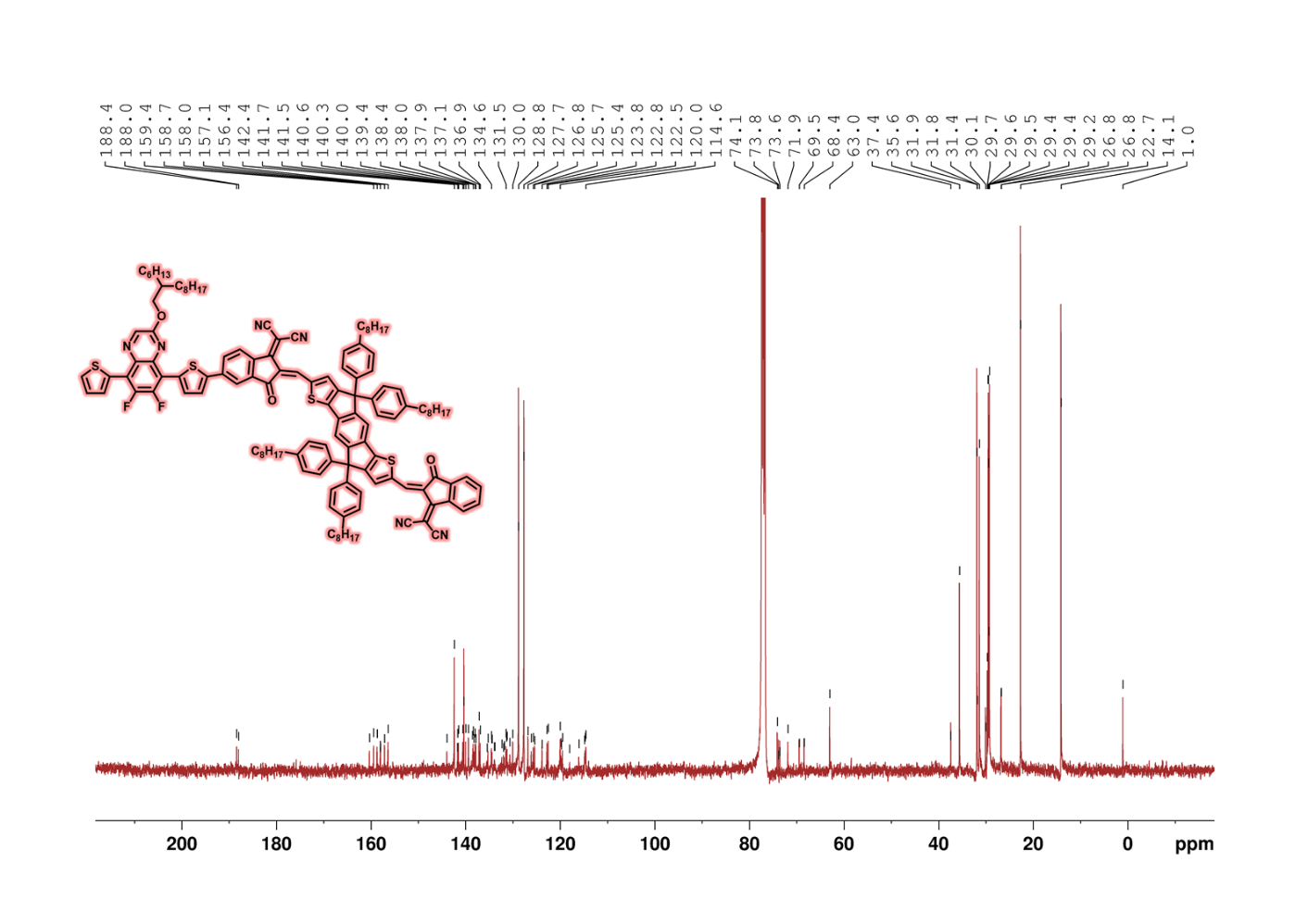


Figure S48. ^13^C NMR spectrum of **AJC1** in CDCl_3_ at 298 K.


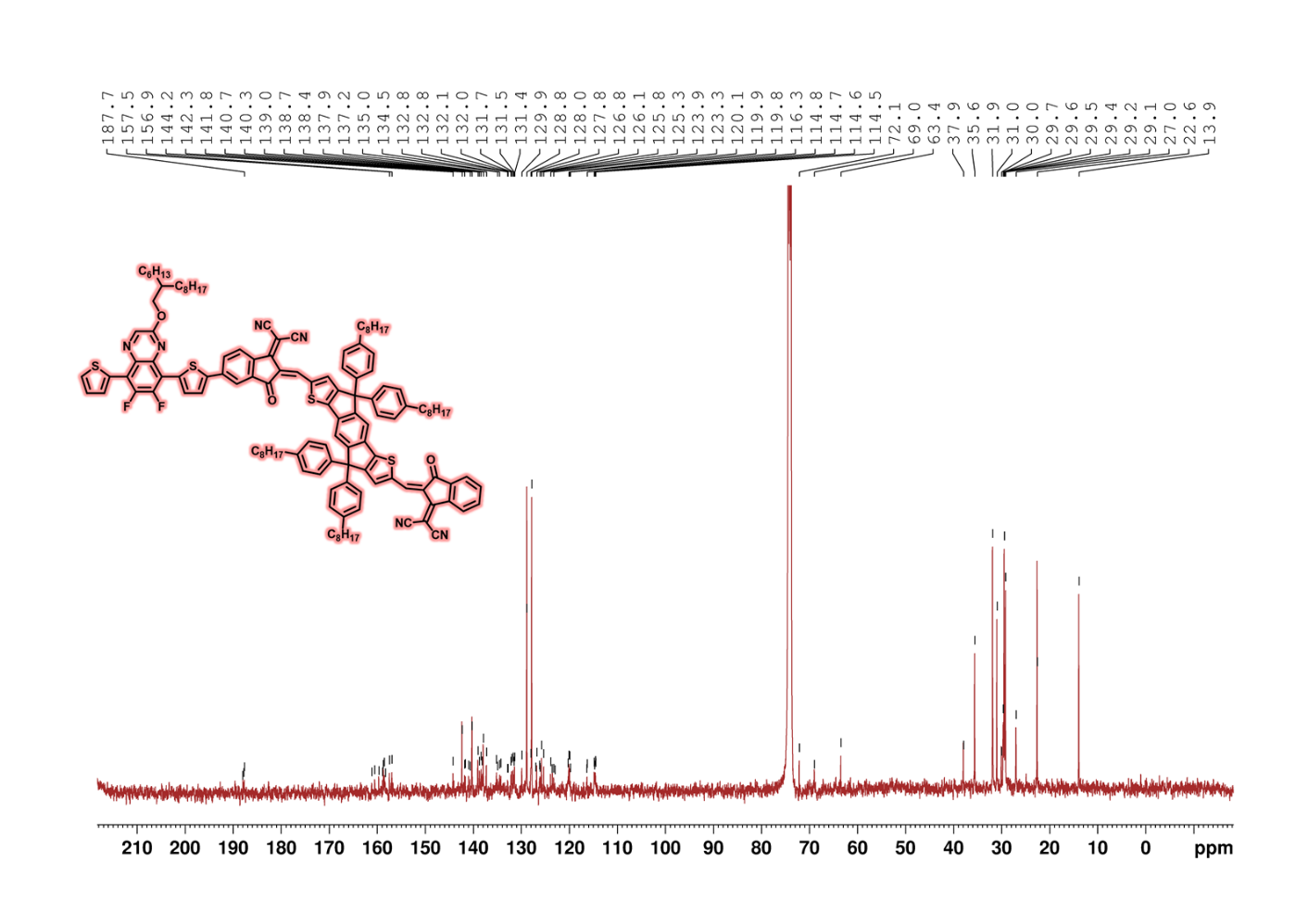


Figure S49. ^13^C NMR spectrum of **AJC1** in C_2_D_2_Cl_4_ at 393 K.


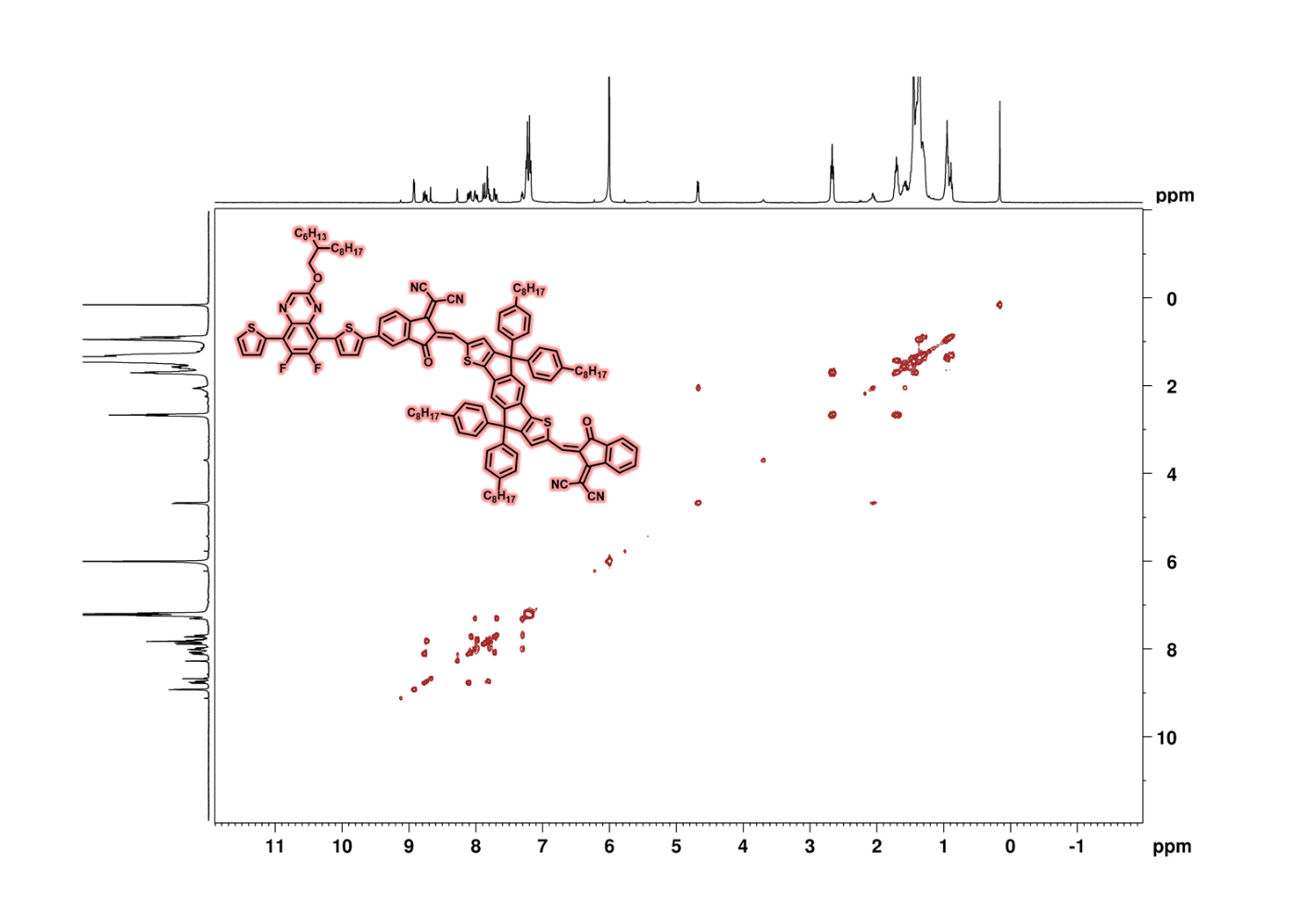


Figure S50. COSY spectrum of **AJC1** in C_2_D_2_Cl_4_ at 393 K.


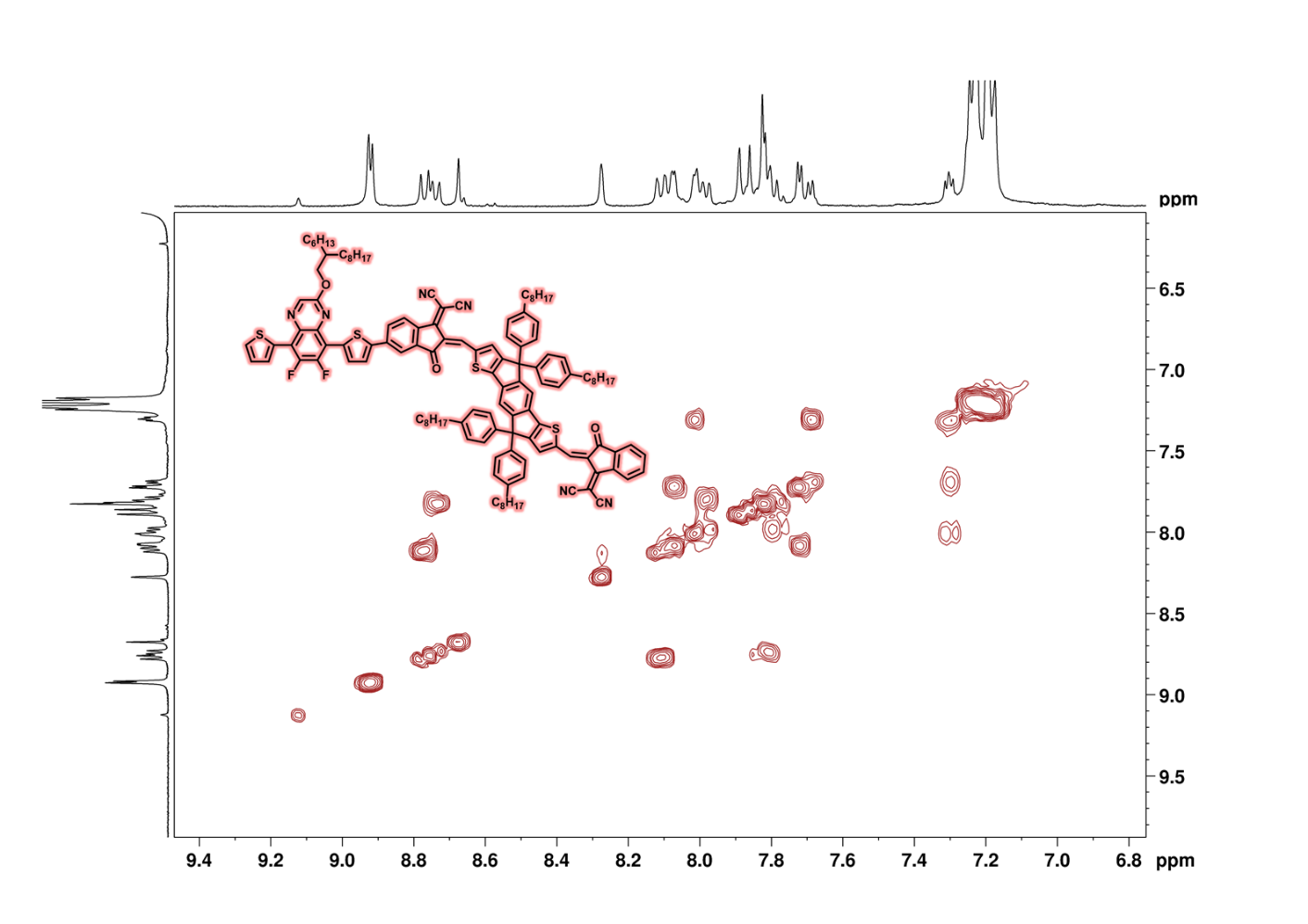


Figure S51. COSY spectrum of **AJC1** in C_2_D_2_Cl_4_ at 393 K zoomed in the aromatic region.


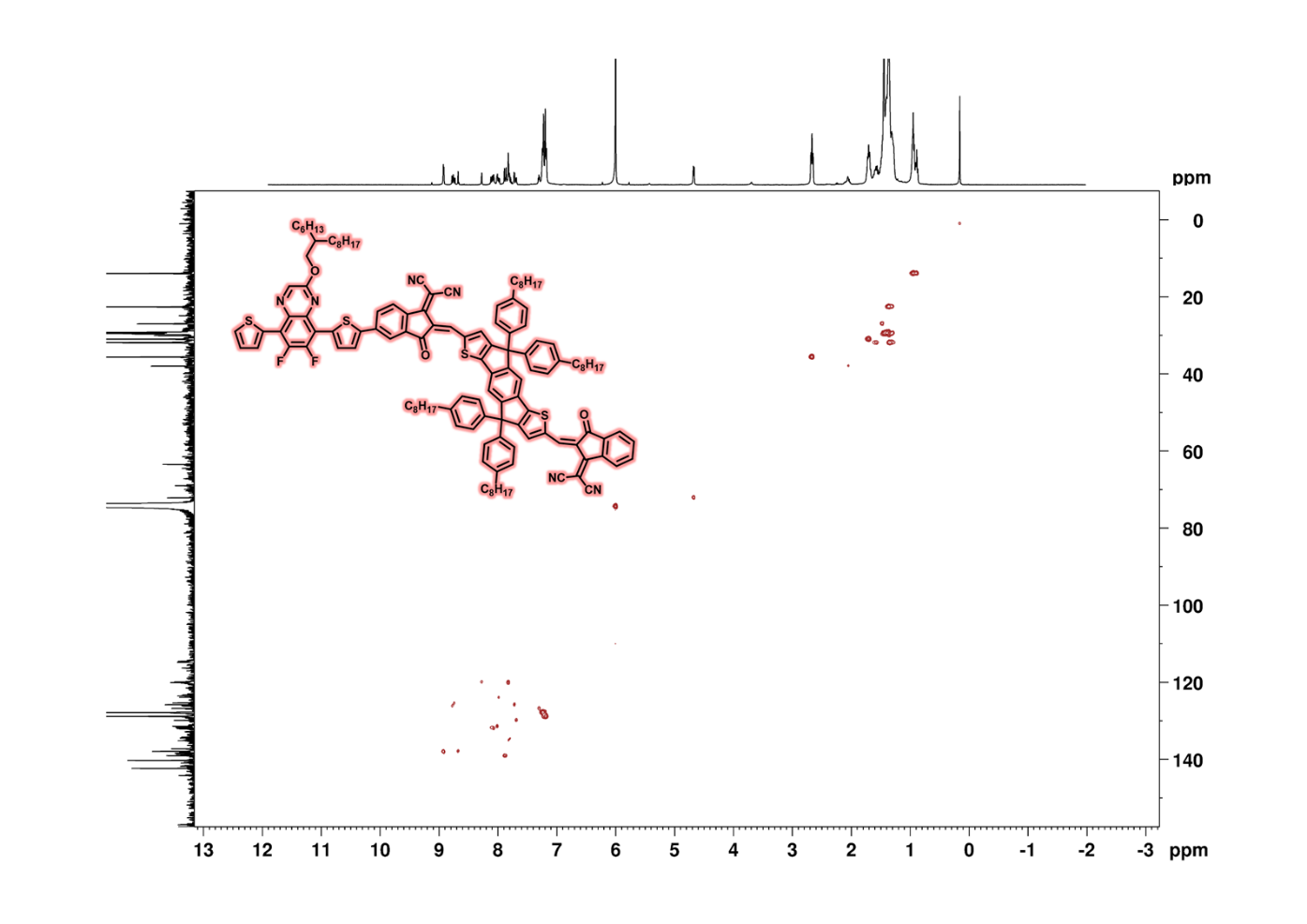


Figure S52. HSQC spectrum of **AJC1** in C_2_D_2_Cl_4_ at 393 K.


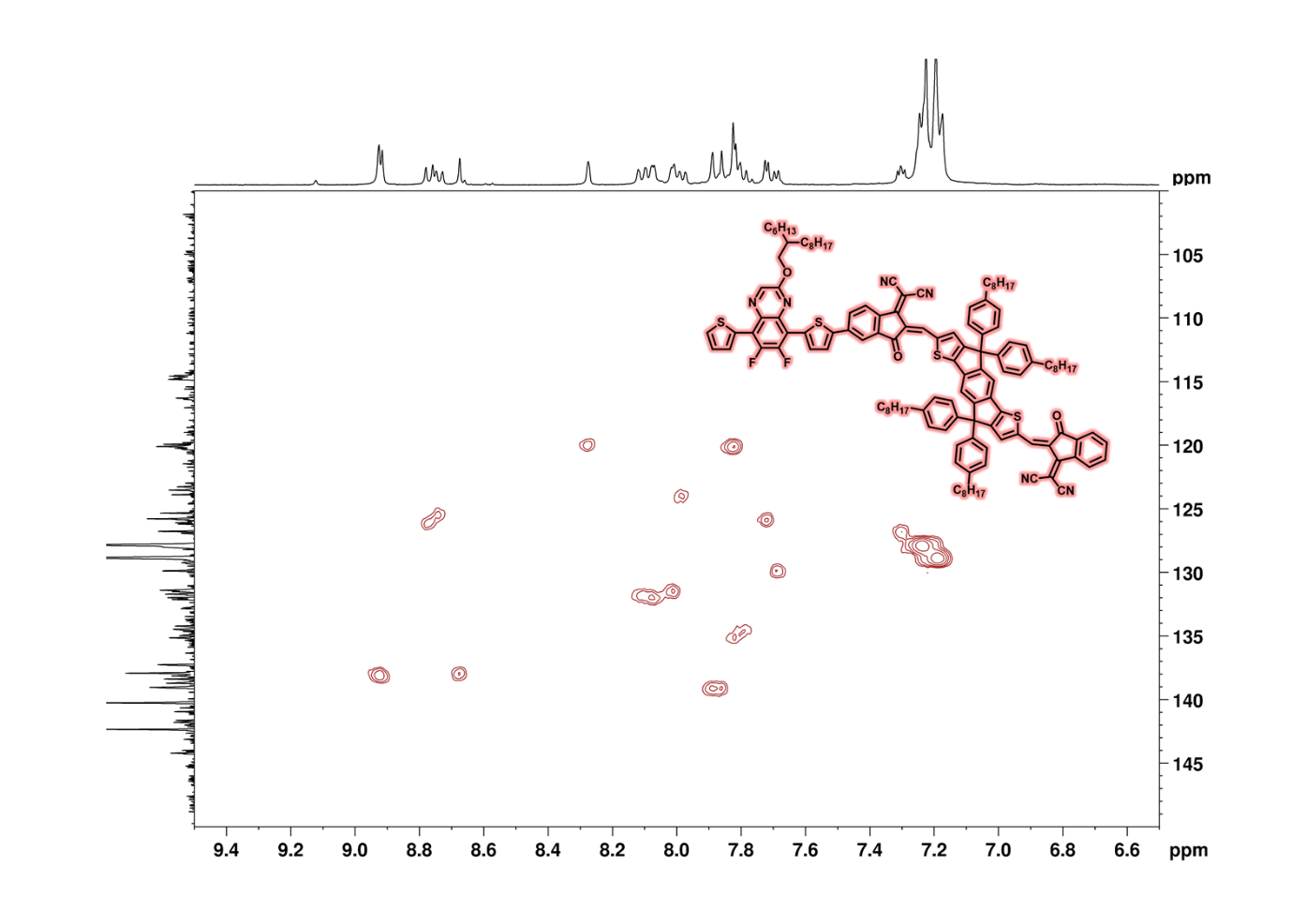


Figure S53. HSQC spectrum of **AJC1** in C_2_D_2_Cl_4_ at 393 K zoomed in the aromatic region.


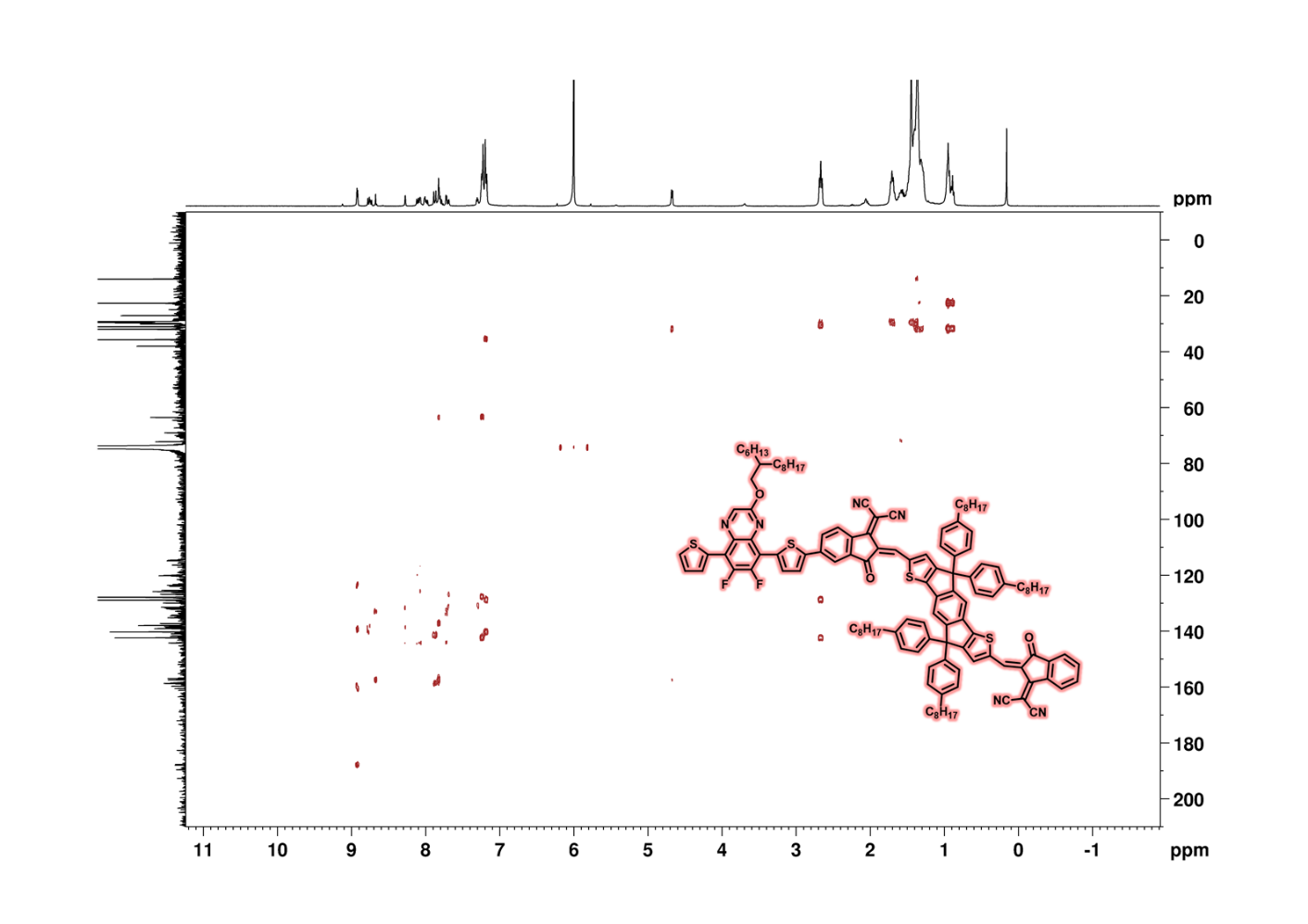


Figure S54. HMBC spectrum of **AJC1** in C_2_D_2_Cl_4_ at 393 K.


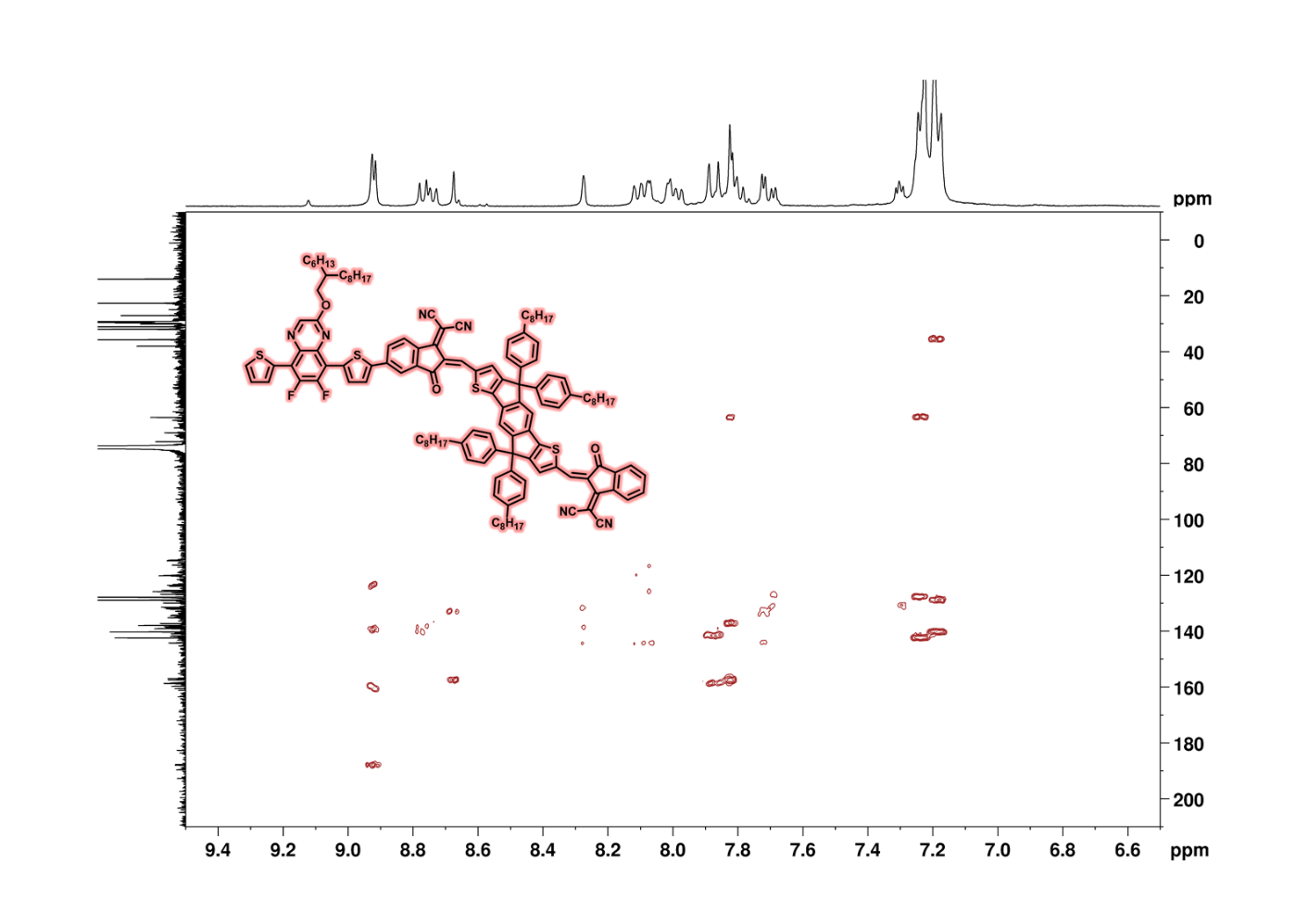


Figure S55. HMBC spectrum of **AJC1** in C_2_D_2_Cl_4_ at 393 K zoom in the aromatic region.


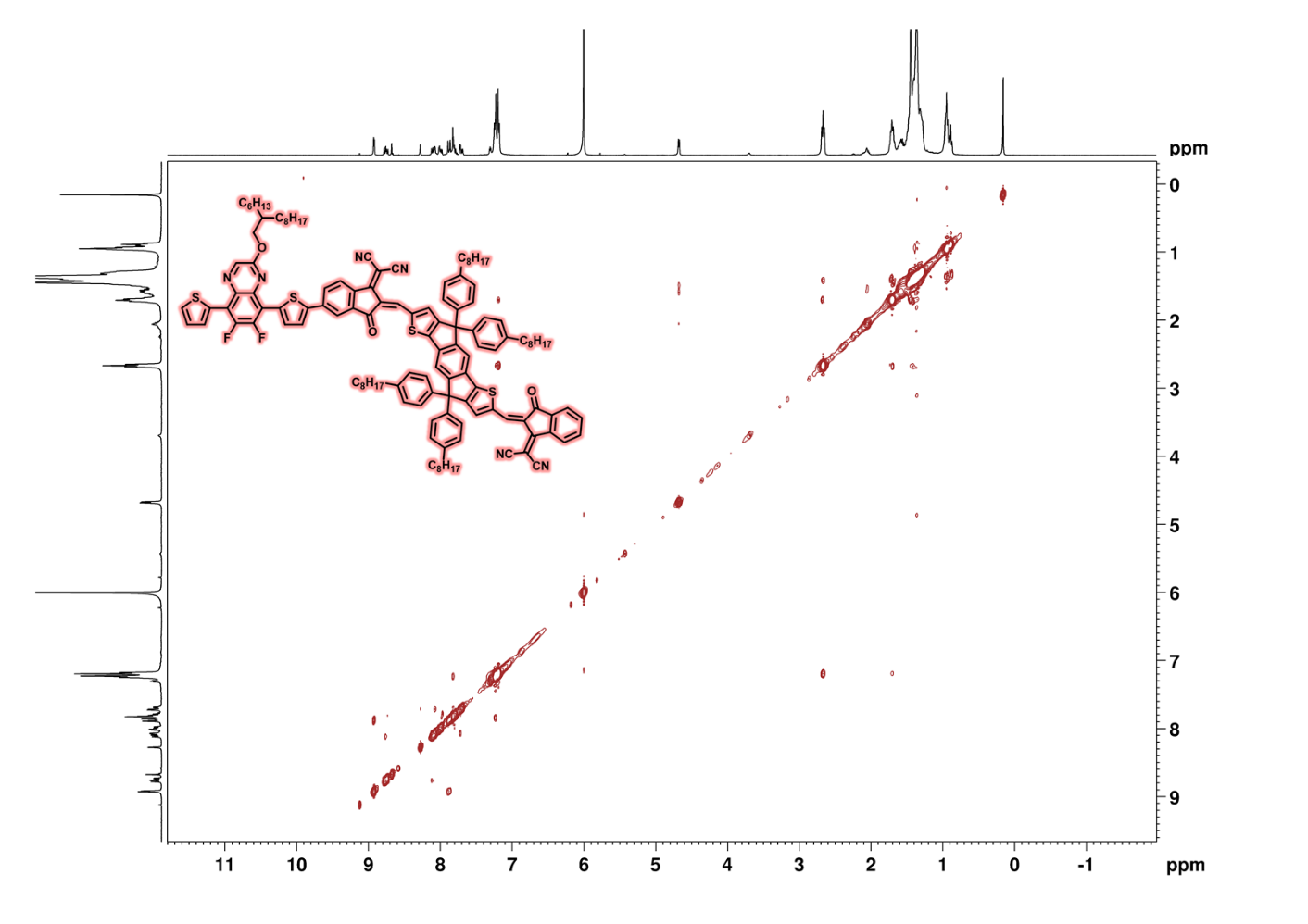


Figure S56. NOESY spectrum of **AJC1** in C_2_D_2_Cl_4_ at 393 K.


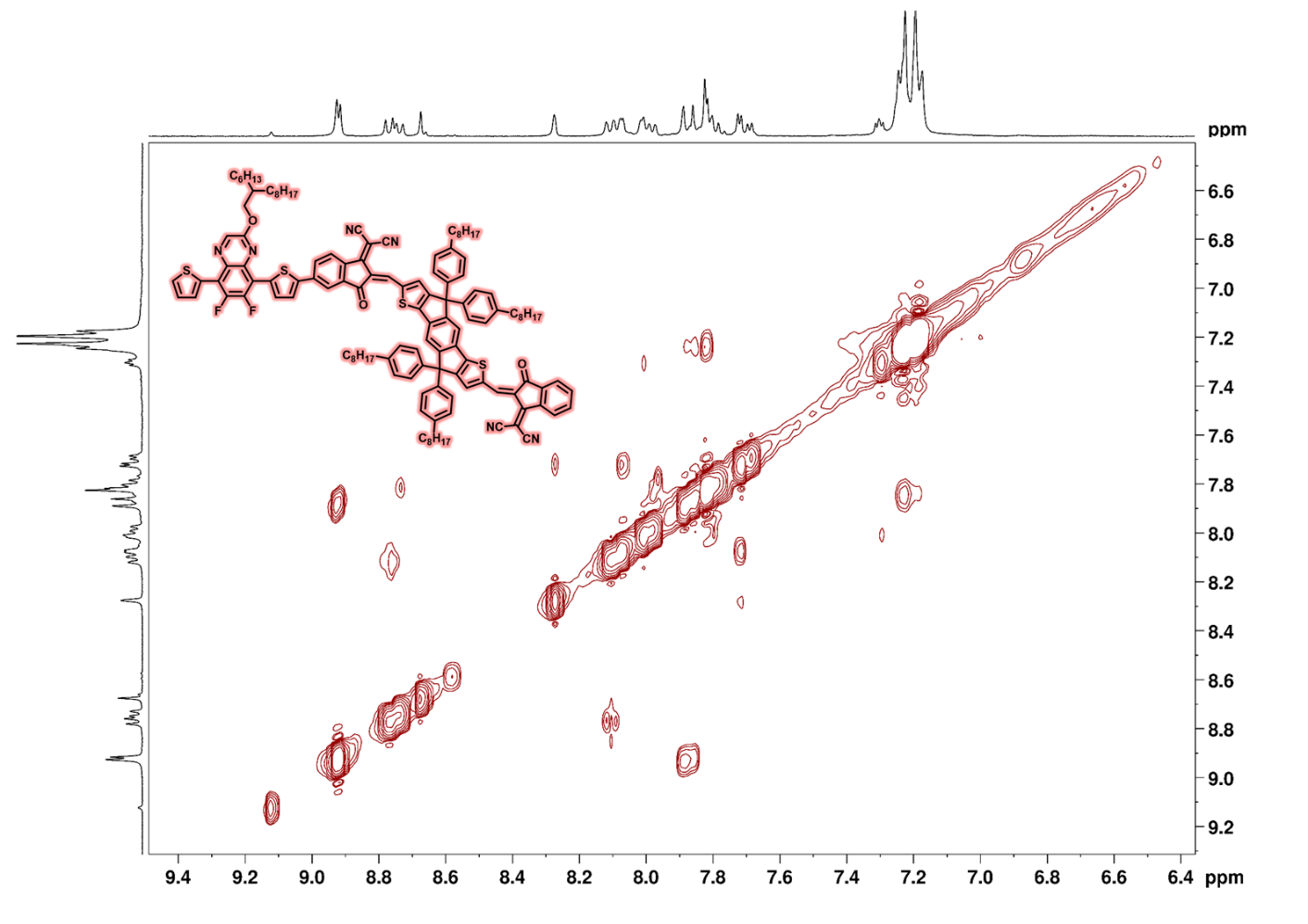


Figure S57. NOESY spectrum of **AJC1** in C_2_D_2_Cl_4_ at 393 K zoomed in the aromatic region.


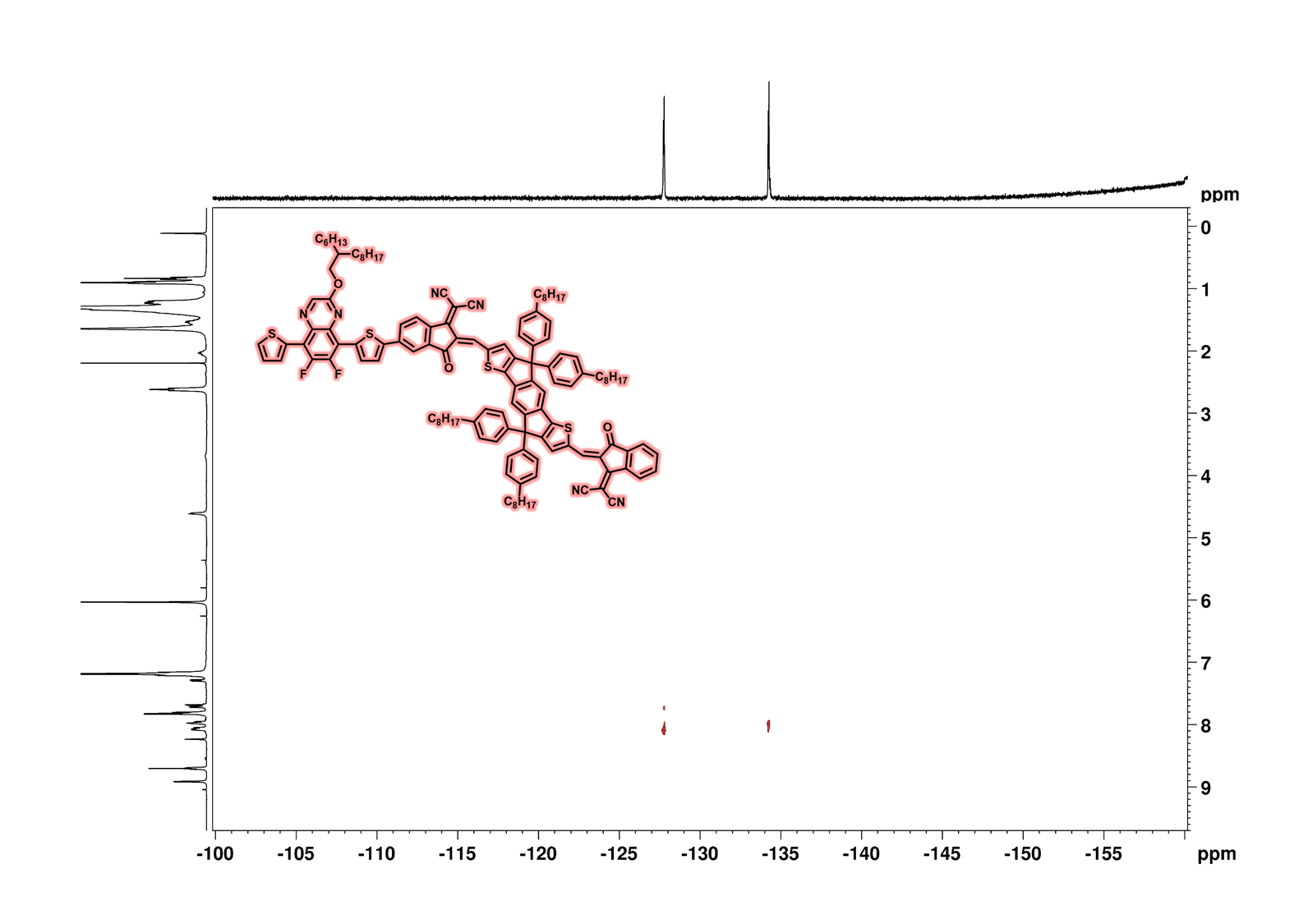


Figure S58. HOESY spectrum of **AJC1** in C_2_D_2_Cl_4_ at 298 K.


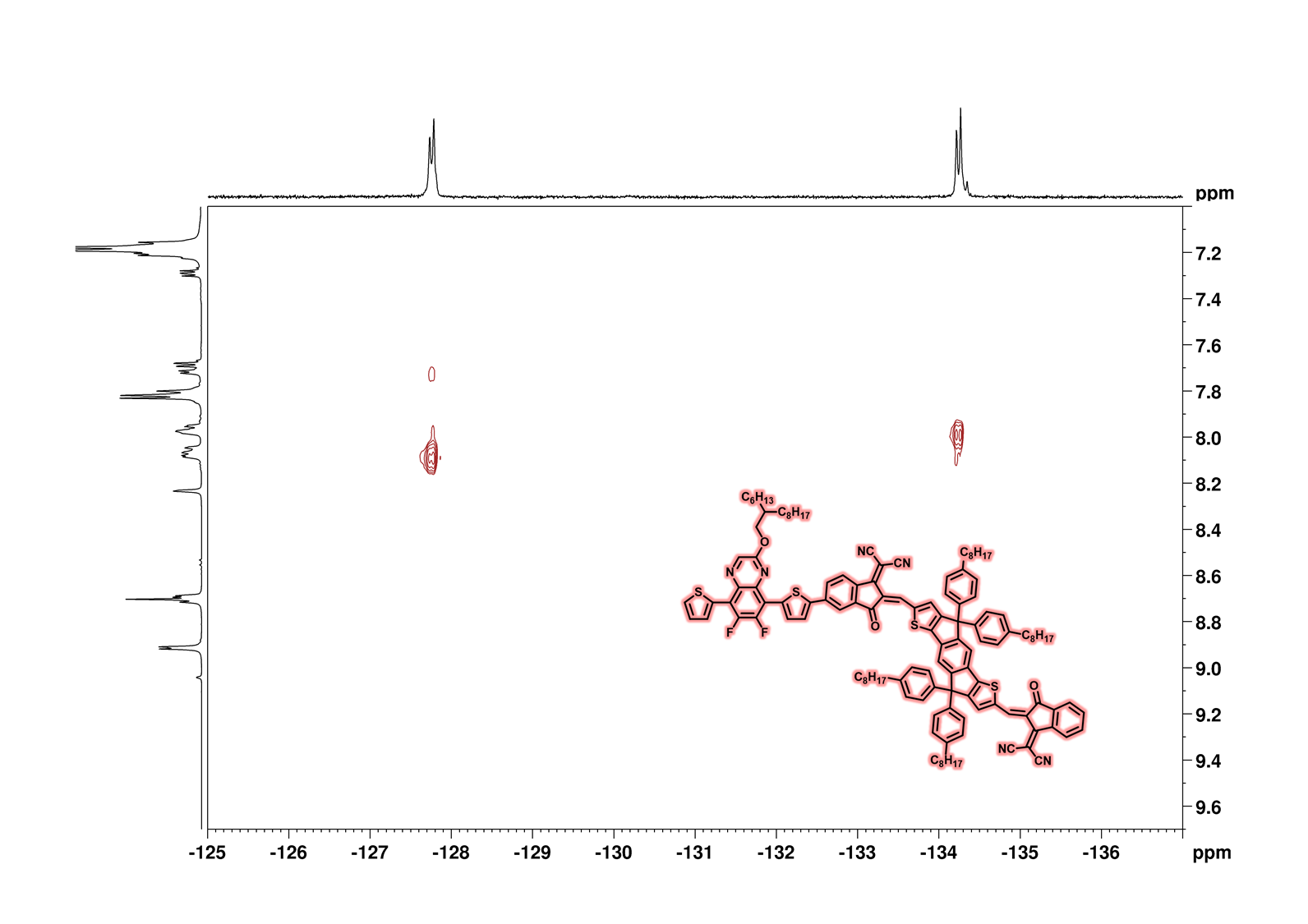


Figure S59. HOESY spectrum of **AJC1** in C_2_D_2_Cl_4_ at 298 K zoomed in the aromatic region.


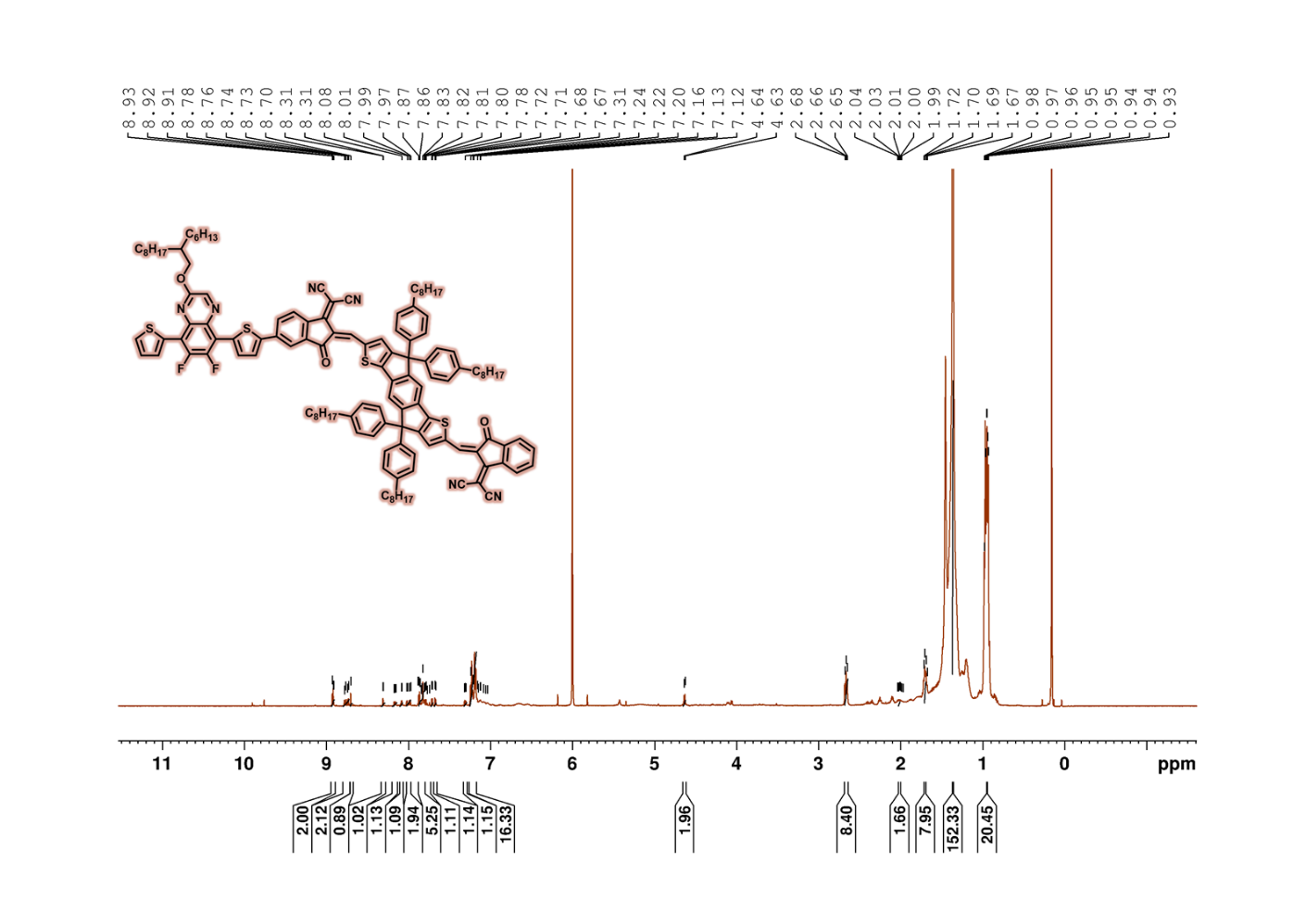


Figure S60. ^1^H NMR spectrum of **AJC2** in C_2_D_2_Cl_4_ at 393 K.


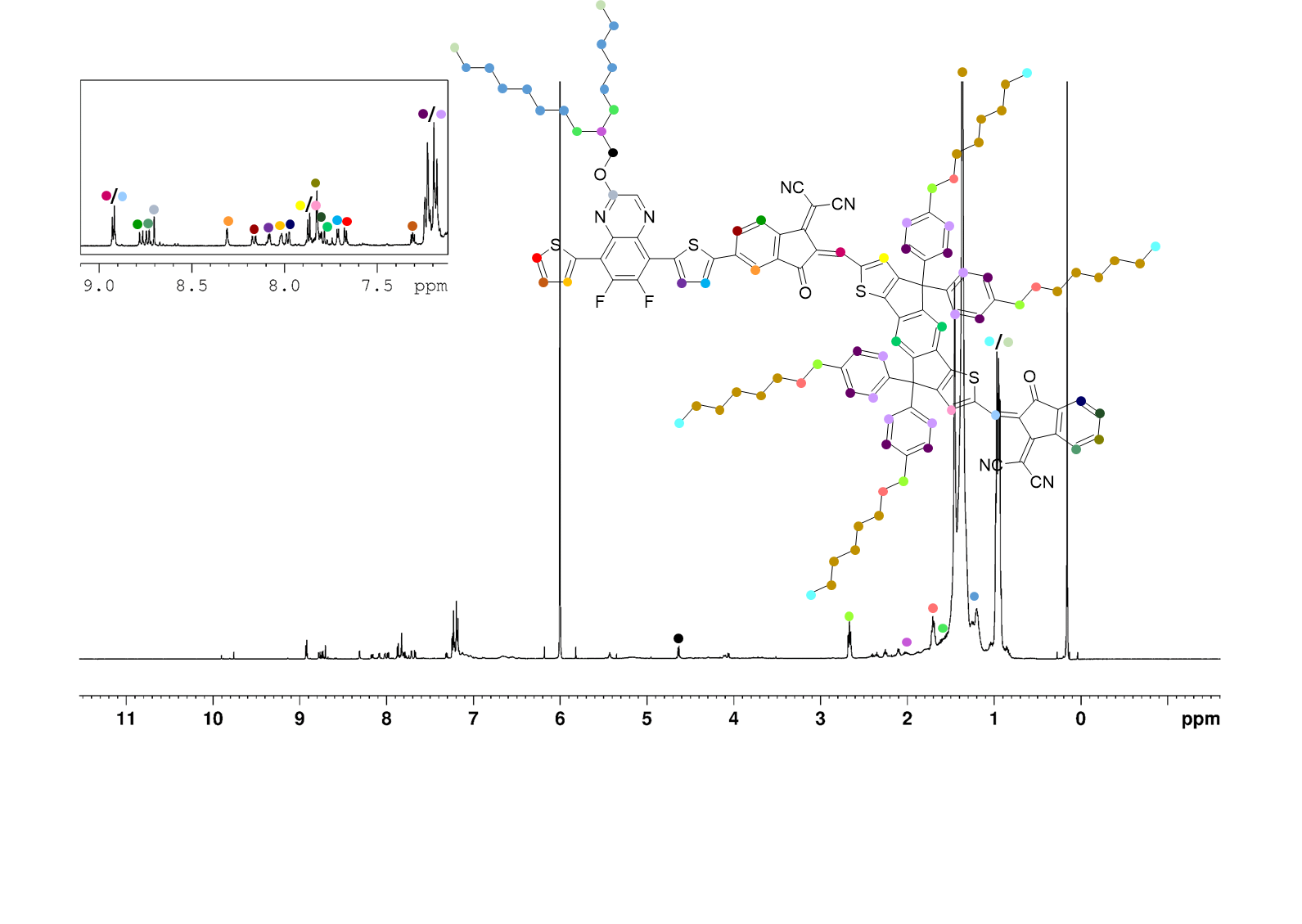


Figure S61. Total ^1^H NMR attribution for **AJC2** in C_2_D_2_Cl_4_ at 393 K.


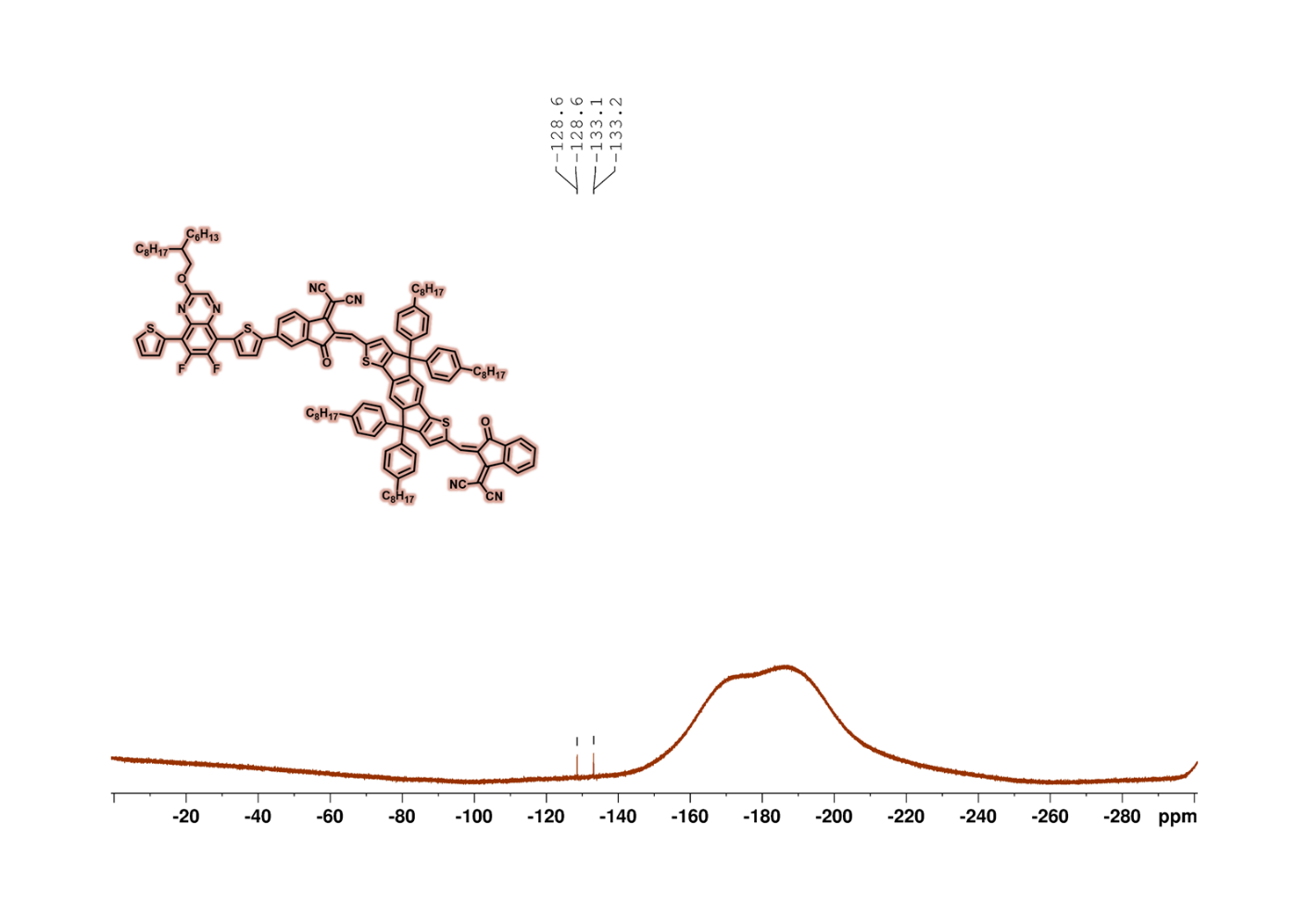


Figure S62. ^19^F NMR spectrum of **AJC2** in C_2_D_2_Cl_4_ at 393 K.


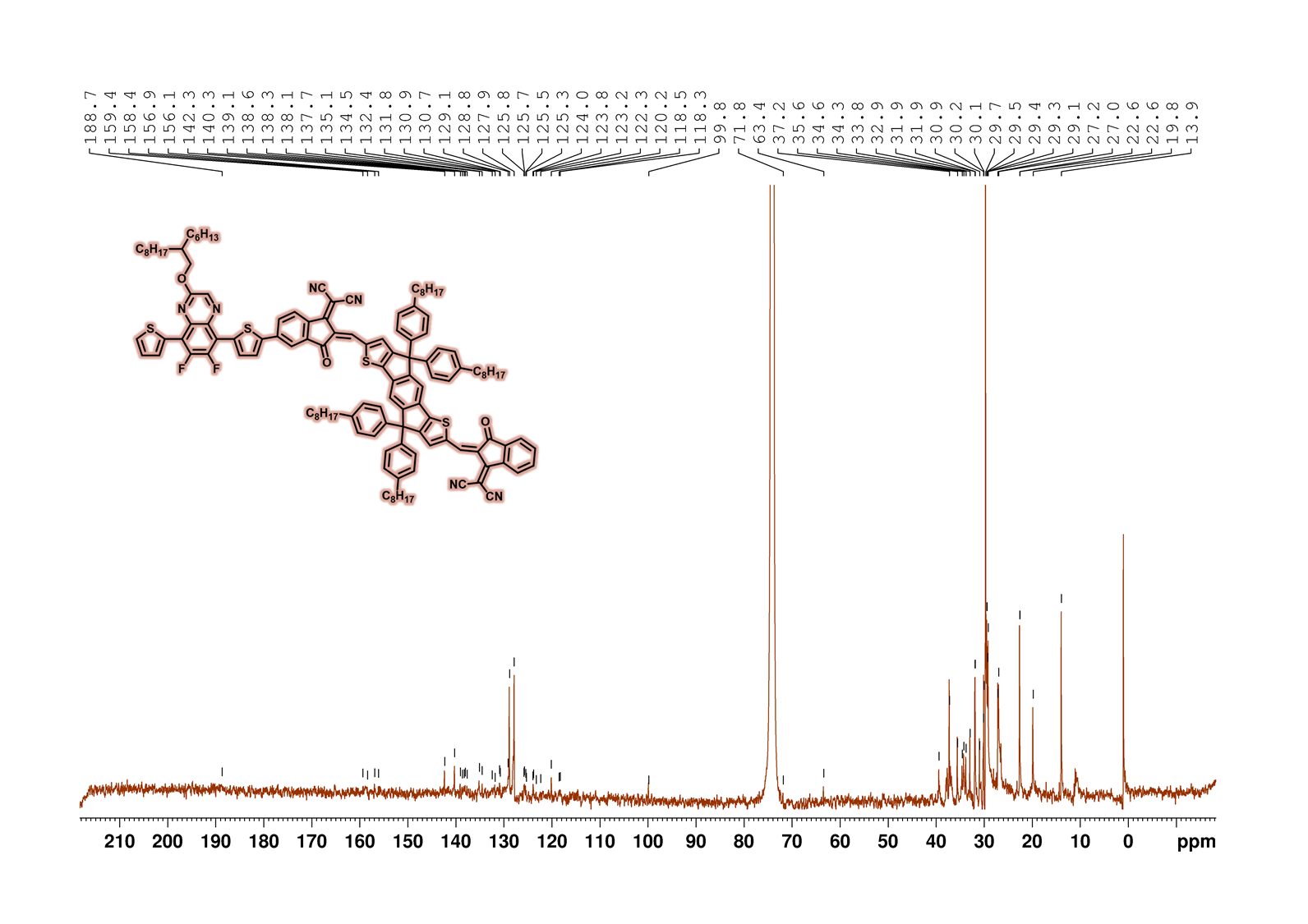


Figure S63. ^13^C NMR spectrum of **AJC2** in C_2_D_2_Cl_4_ at 393 K.


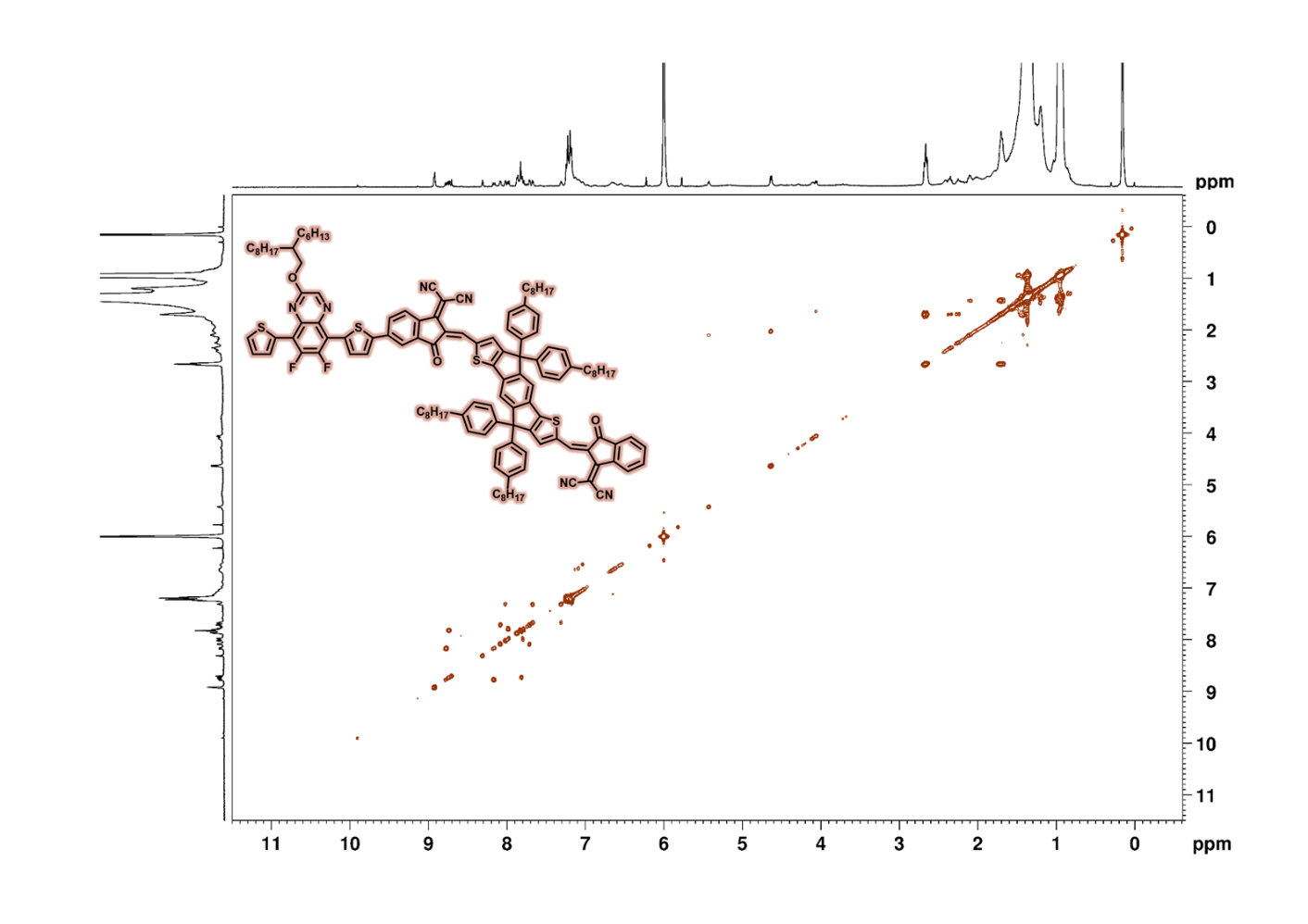


Figure S64. COSY spectrum of **AJC2** in C_2_D_2_Cl_4_ at 393 K.


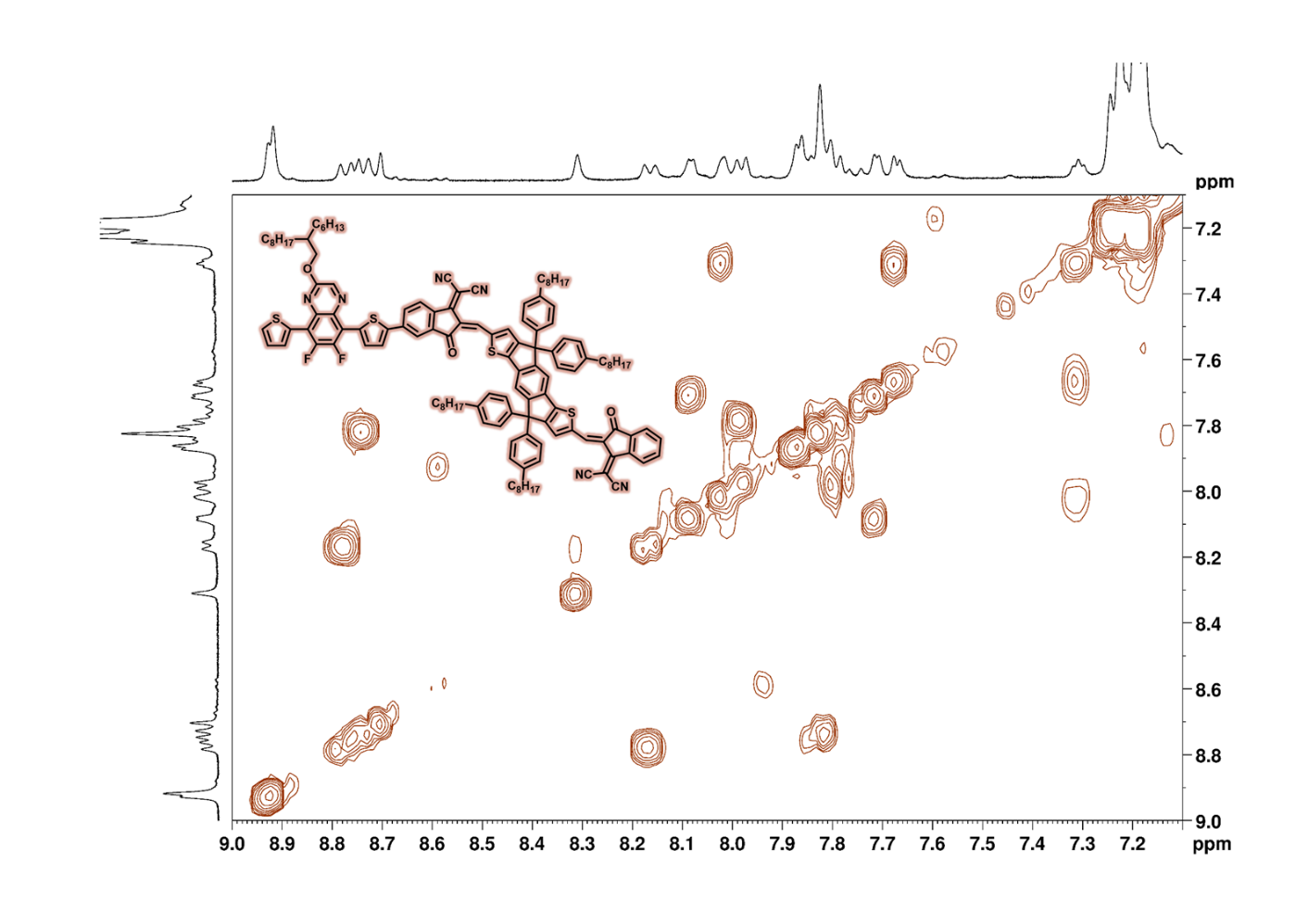


Figure S65. COSY spectrum of **AJC2** in C_2_D_2_Cl_4_ at 393 K zoomed in the aromatic region.


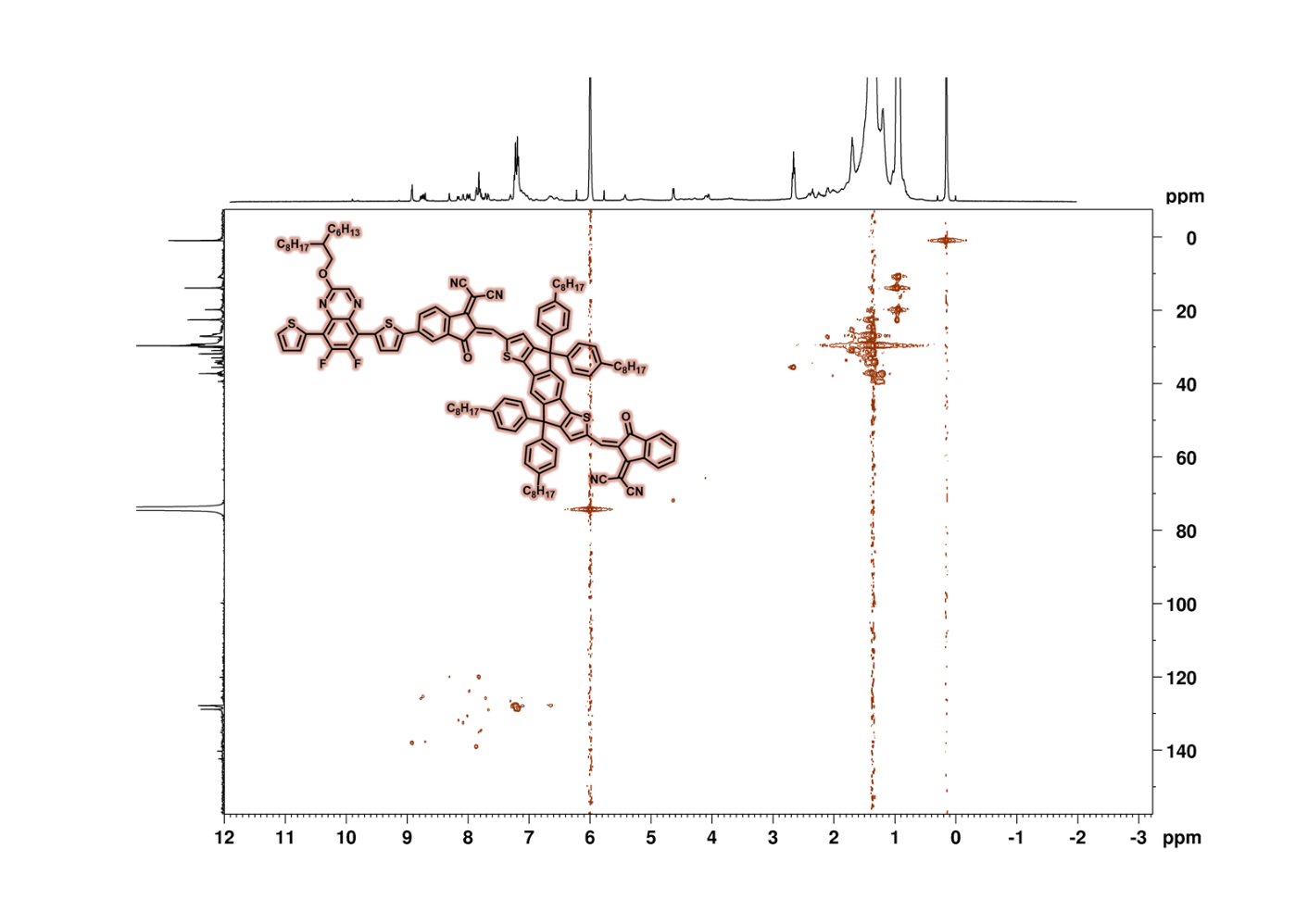


Figure S66. HSQC spectrum of **AJC2** in C_2_D_2_Cl_4_ at 393 K.


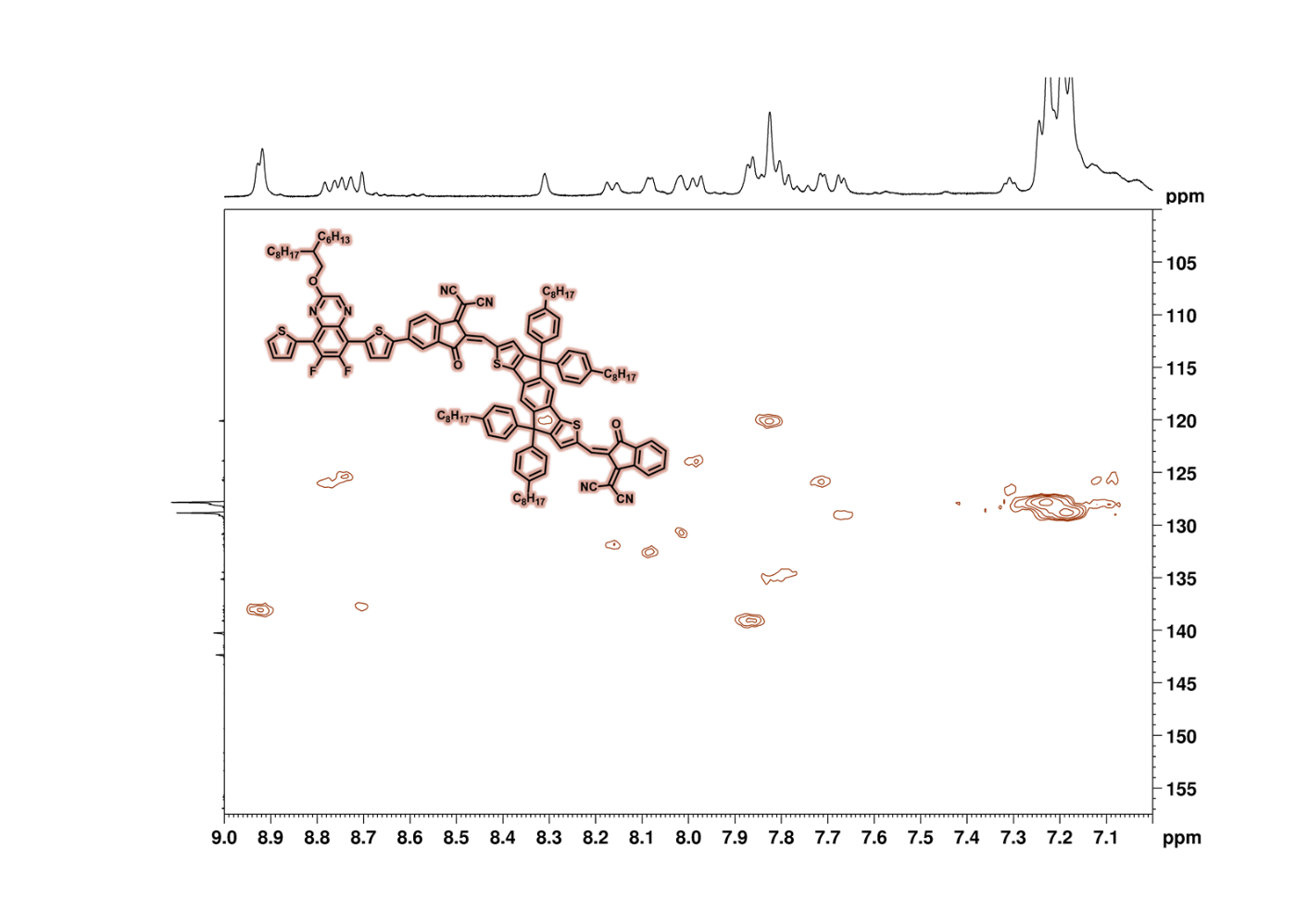


Figure S67. HSQC spectrum of **AJC2** in C_2_D_2_Cl_4_ at 393 K zoomed in the aromatic region.


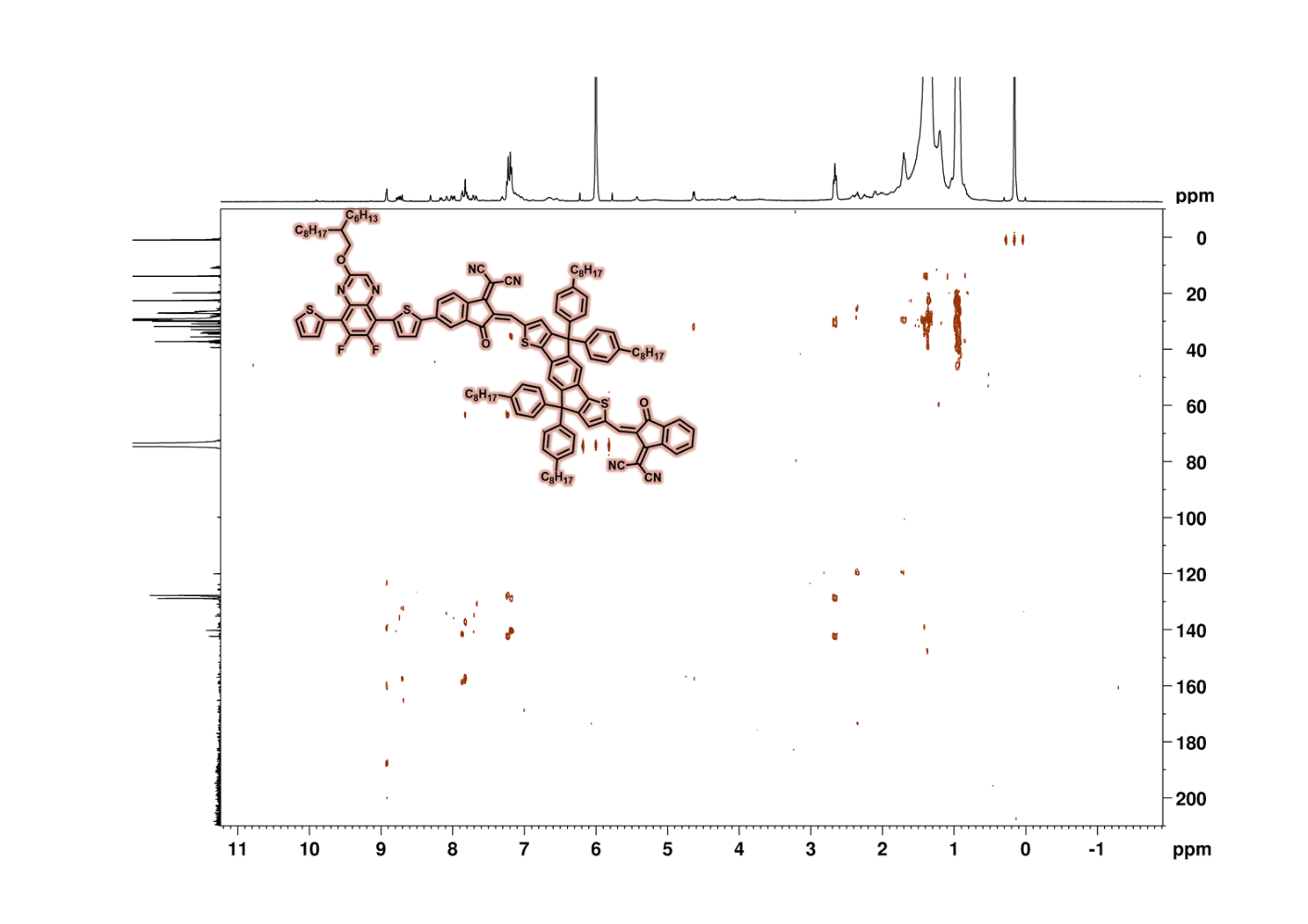


Figure S68. HMBC spectrum of **AJC2** in C_2_D_2_Cl_4_ at 393 K.


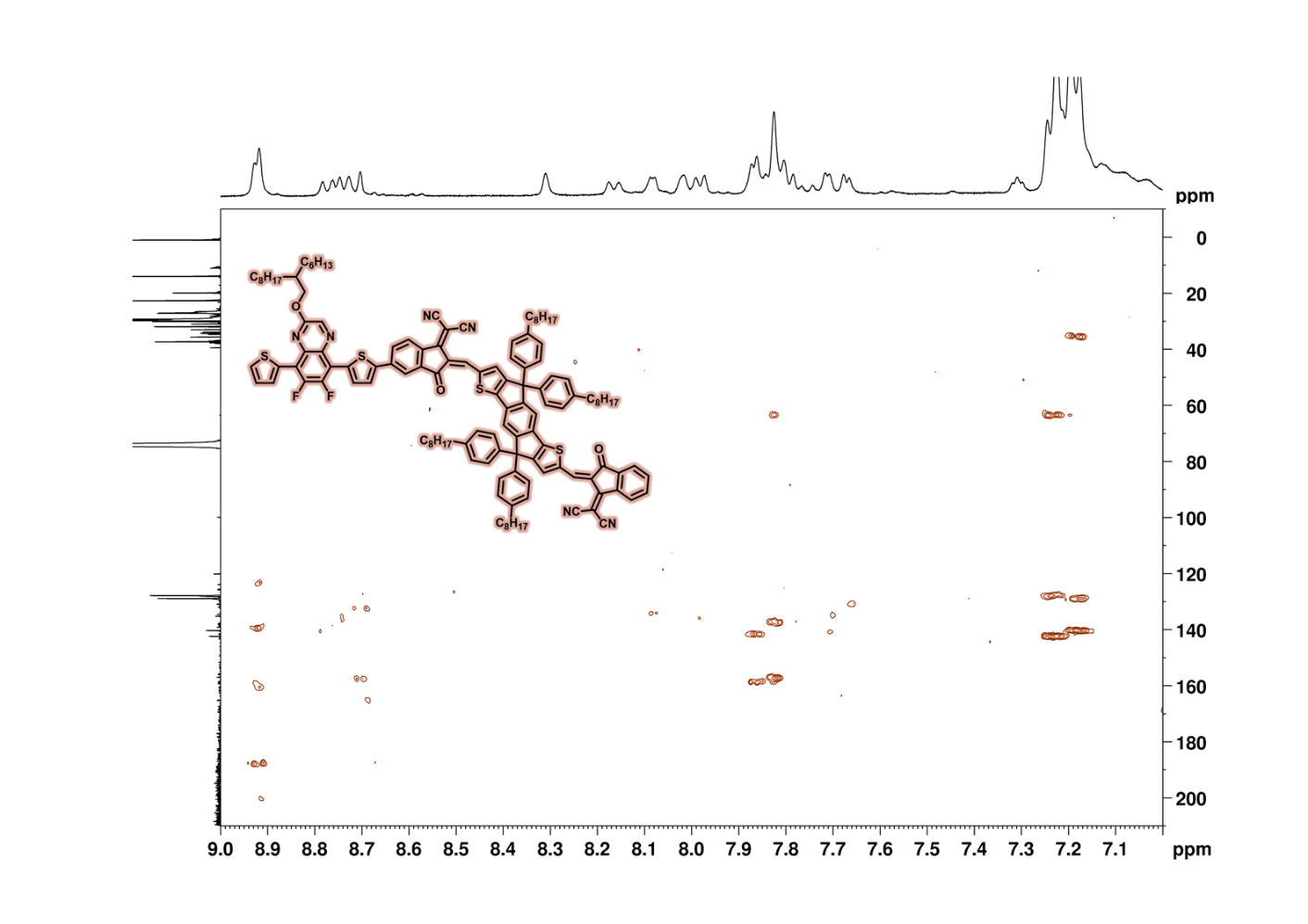


Figure S69. HMBC spectrum of **AJC2** in C_2_D_2_Cl_4_ at 393 K zoomed in the aromatic region.


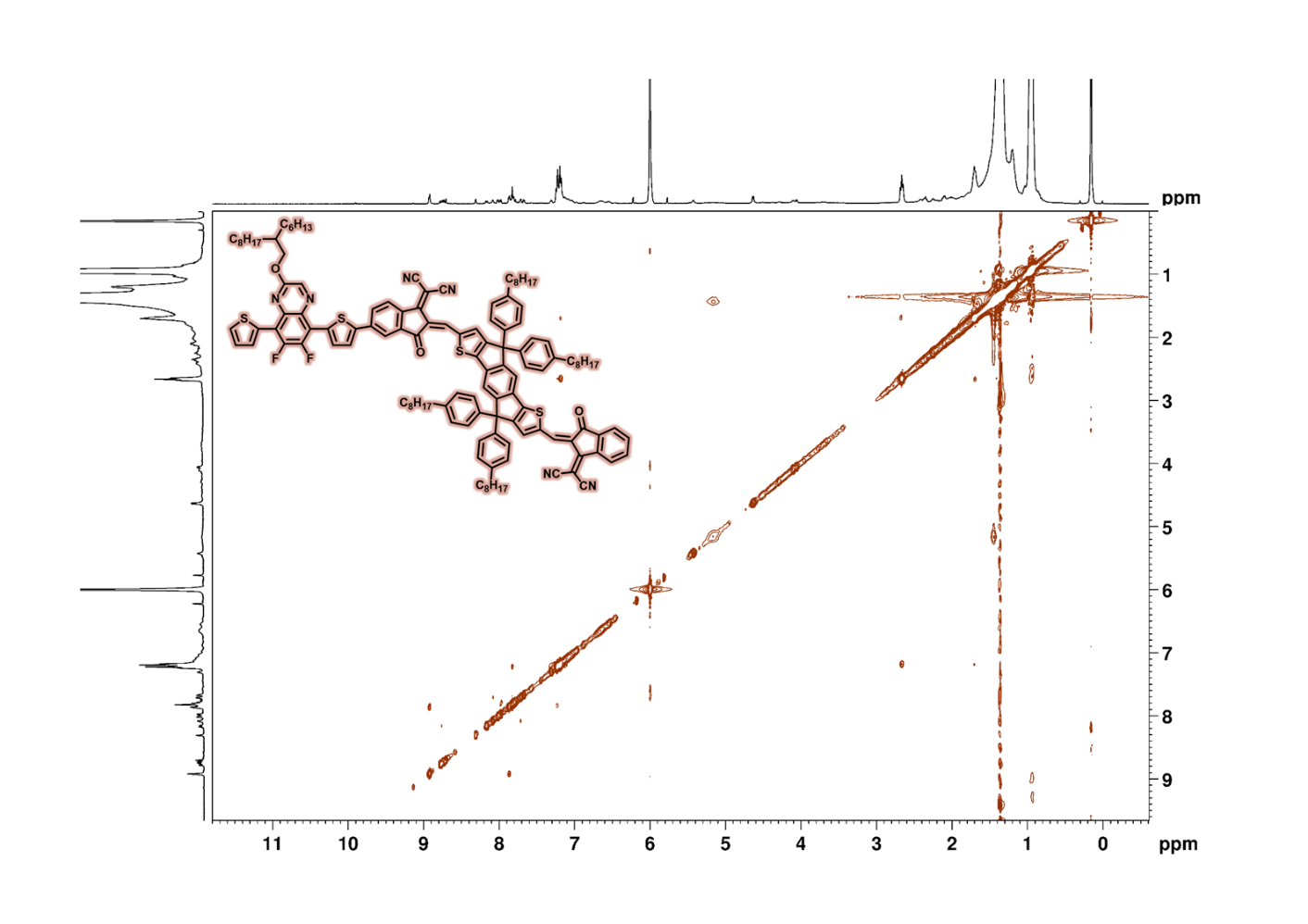


Figure S70. NOESY spectrum of **AJC2** in C_2_D_2_Cl_4_ at 393 K.


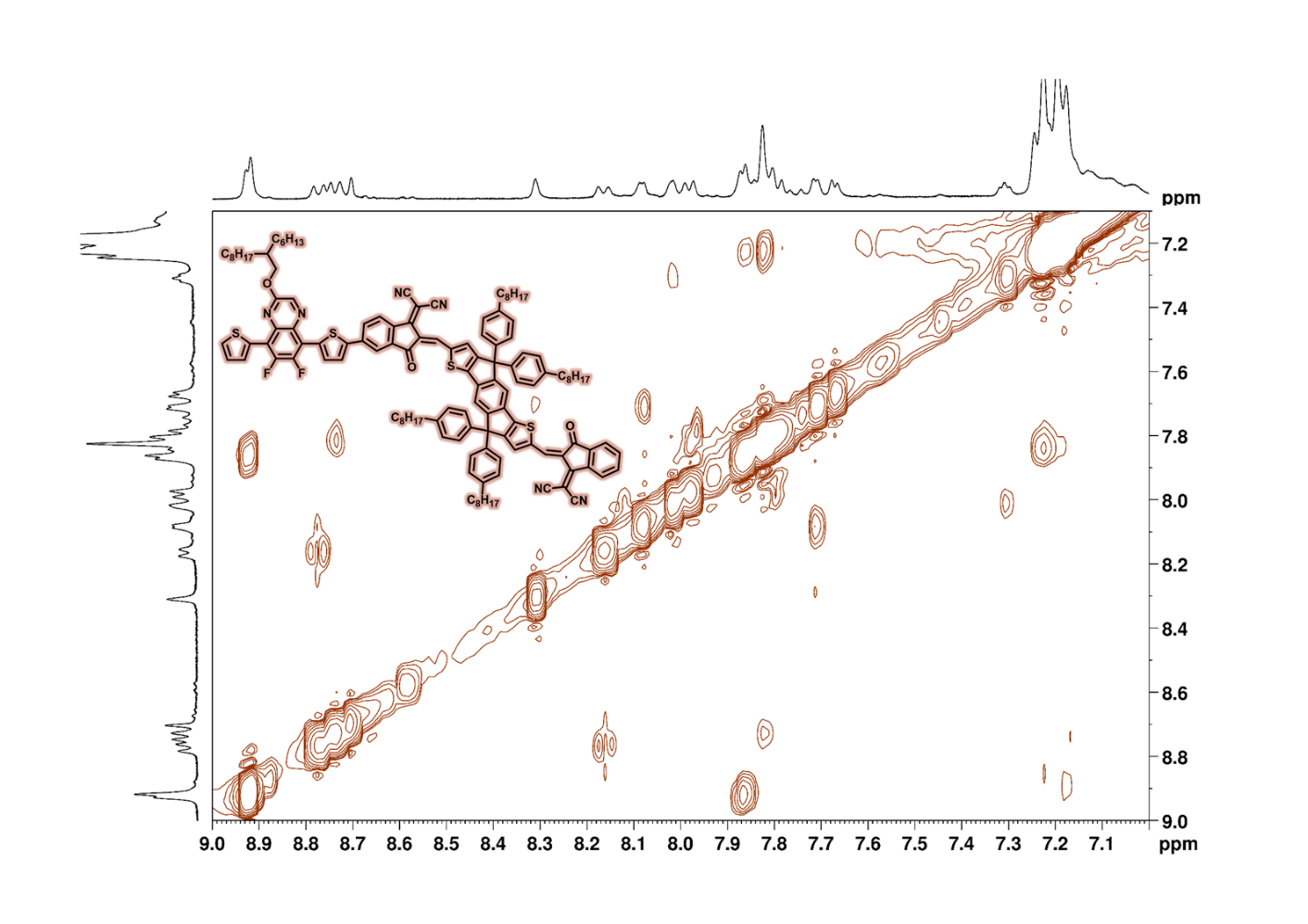


Figure S71. NOESY spectrum of **AJC2** in C_2_D_2_Cl_4_ at 393 K zoomed in the aromatic region.


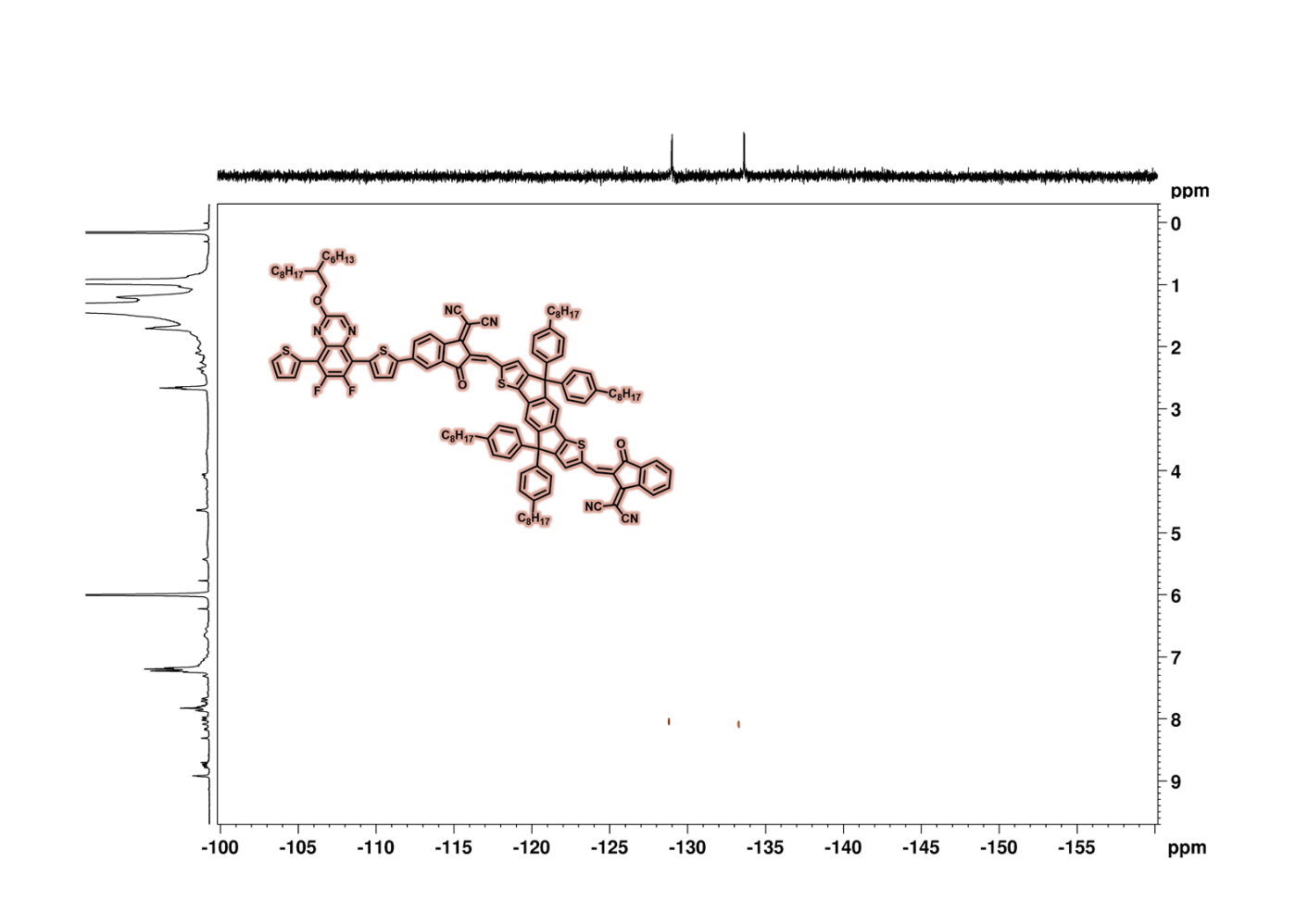


Figure S72. HOESY spectrum of **AJC2** in C_2_D_2_Cl_4_ at 393 K.


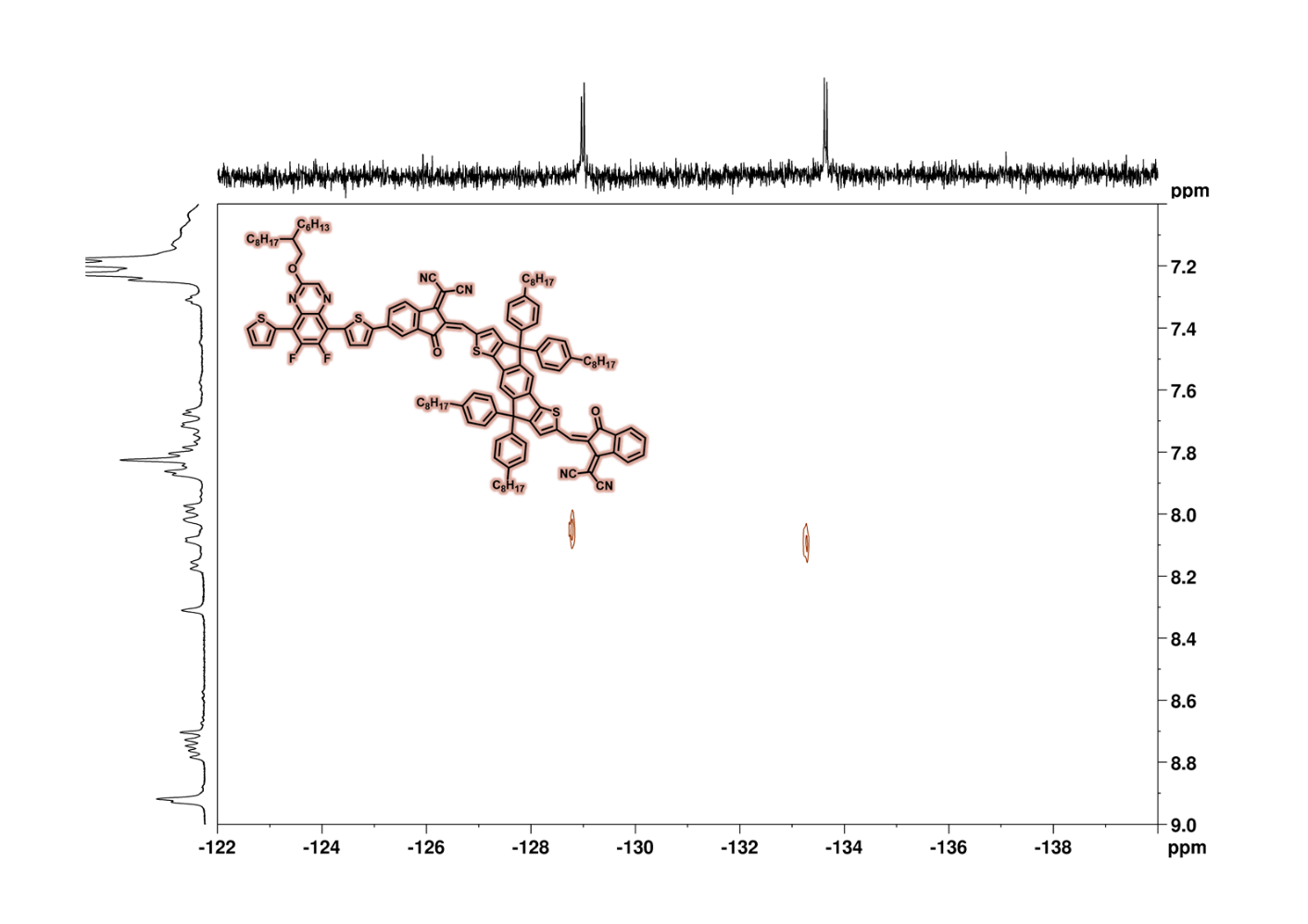


Figure S73. HOESY spectrum of **AJC2** in C_2_D_2_Cl_4_ at 393 K zoomed in the aromatic region.


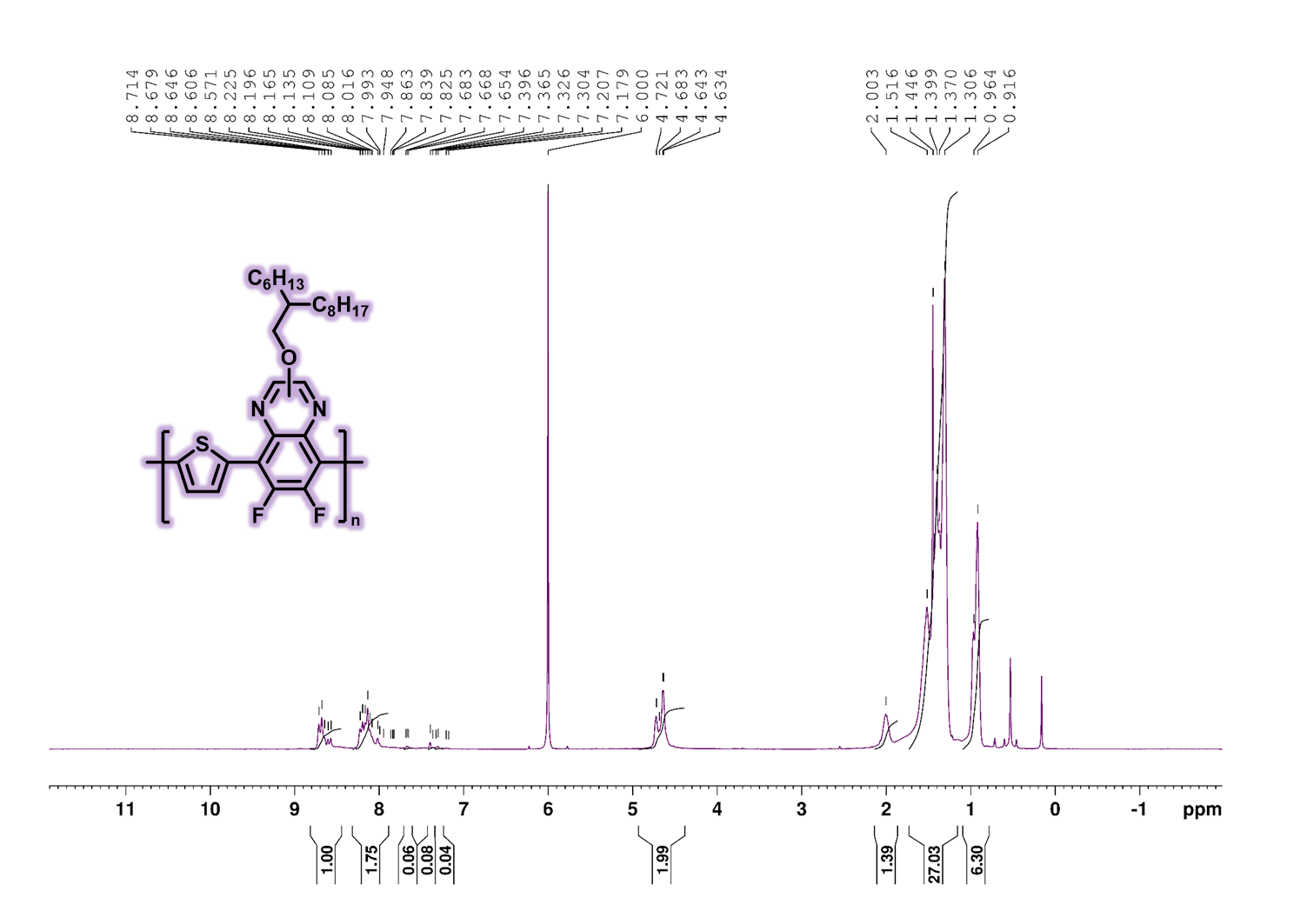


Figure S74. ^1^H NMR spectrum of PTQ10 in C_2_D_2_Cl_4_ at 393 K.


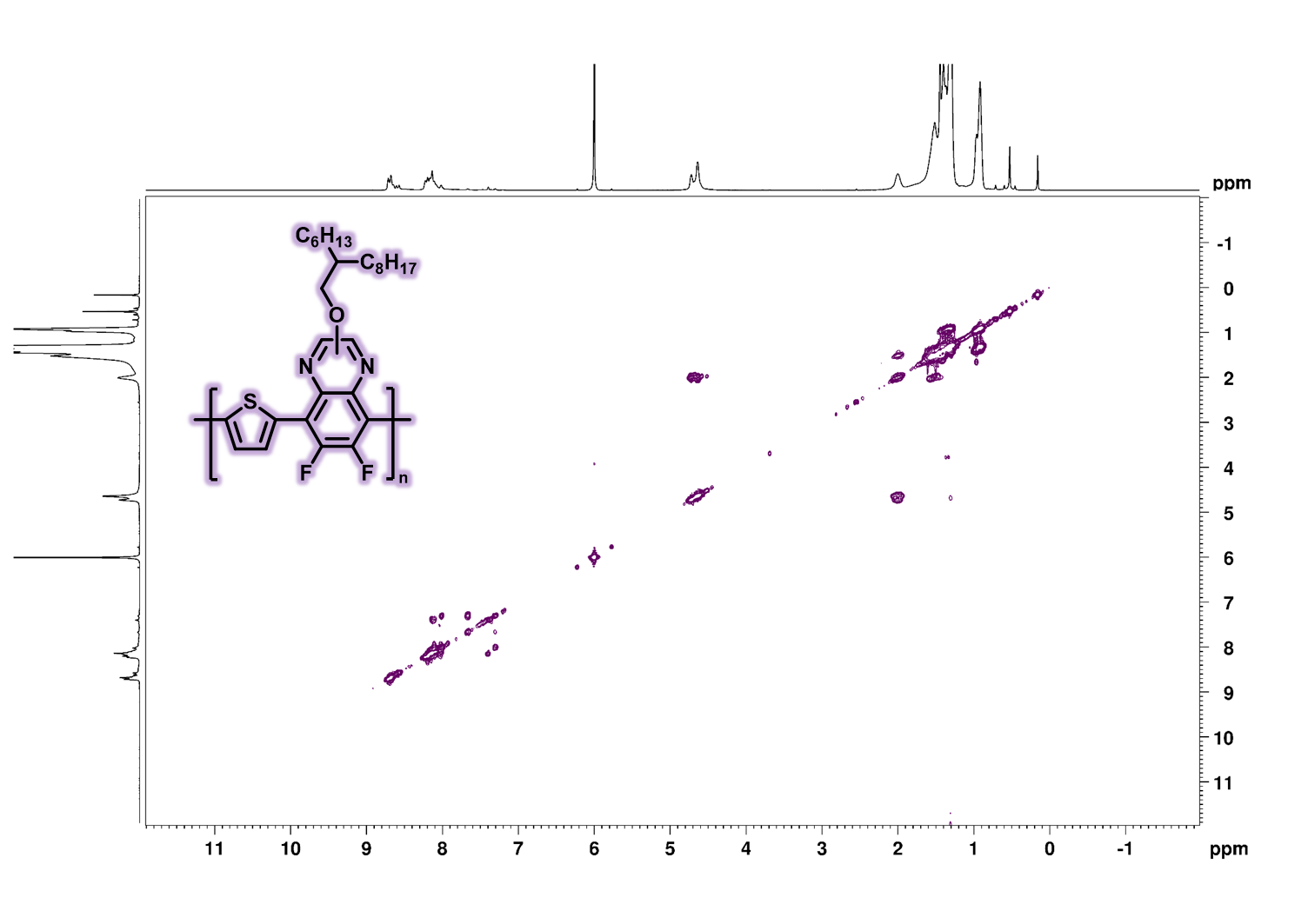


Figure S75. COSY spectrum of PTQ10 in C_2_D_2_Cl_4_ at 393 K.


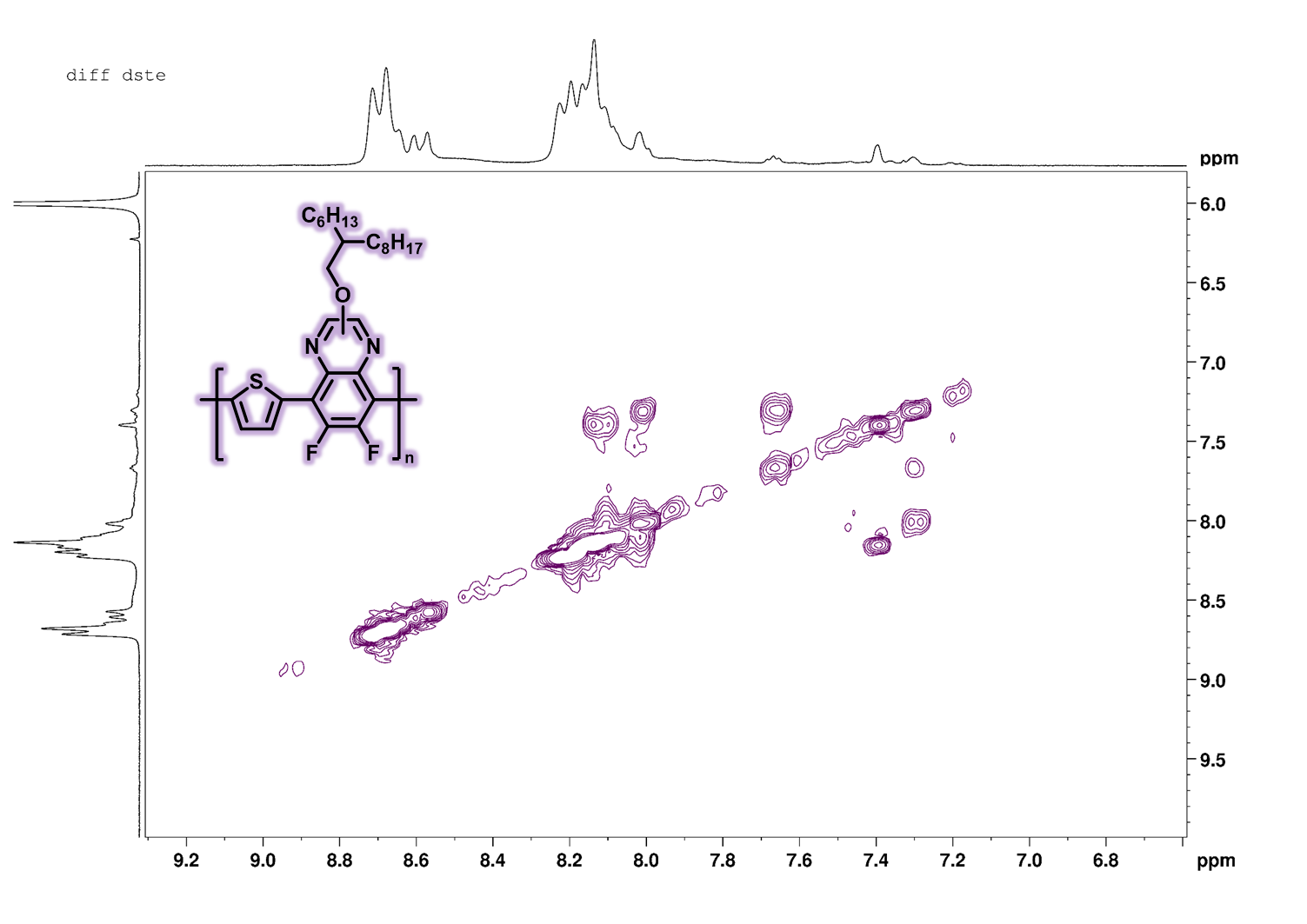


Figure S76. COSY spectrum of PTQ10 in C_2_D_2_Cl_4_ at 393 K zoomed in the aromatic region.


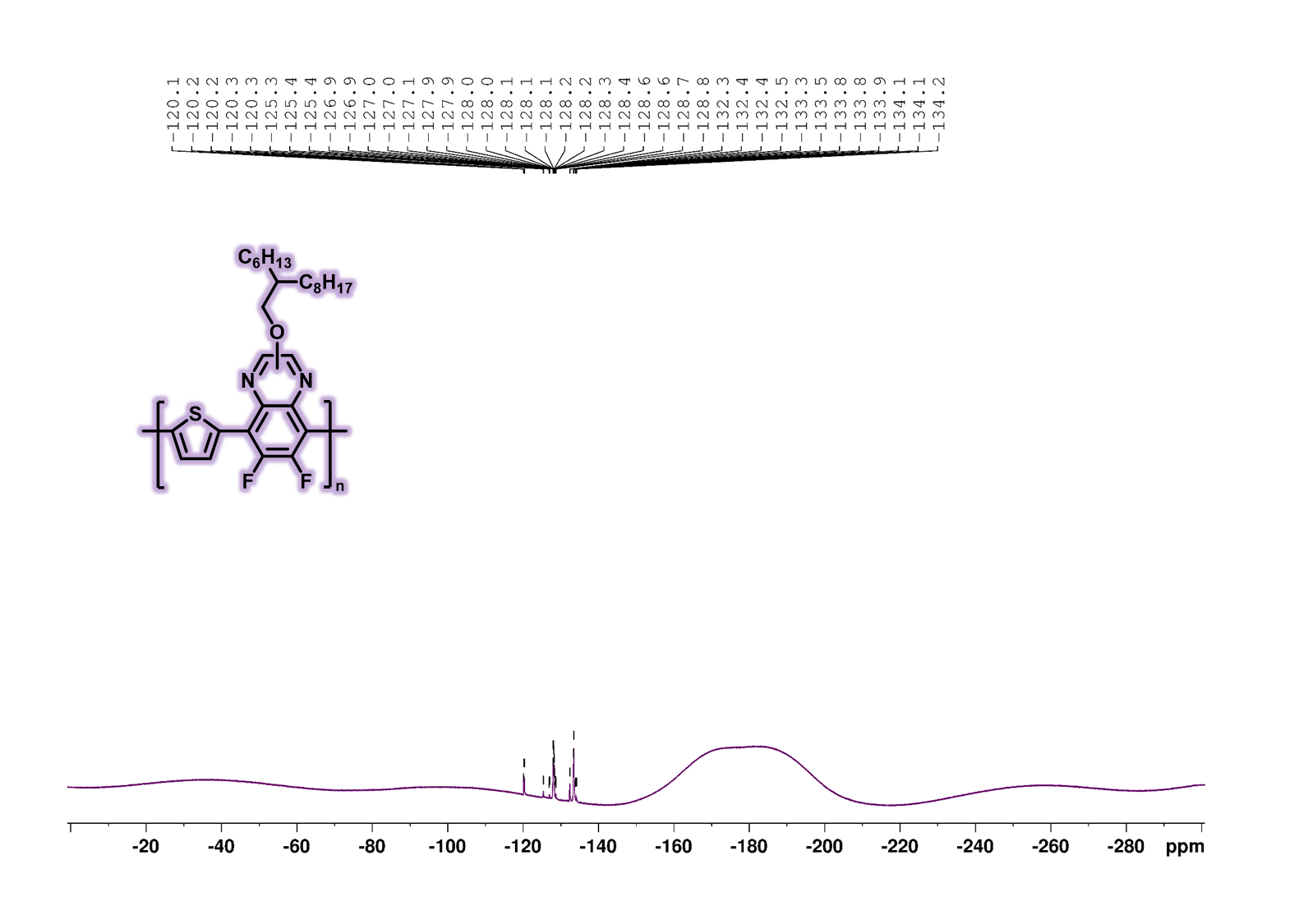


Figure S77. ^19^F spectrum of PTQ10 in C_2_D_2_Cl_4_ at 393 K.


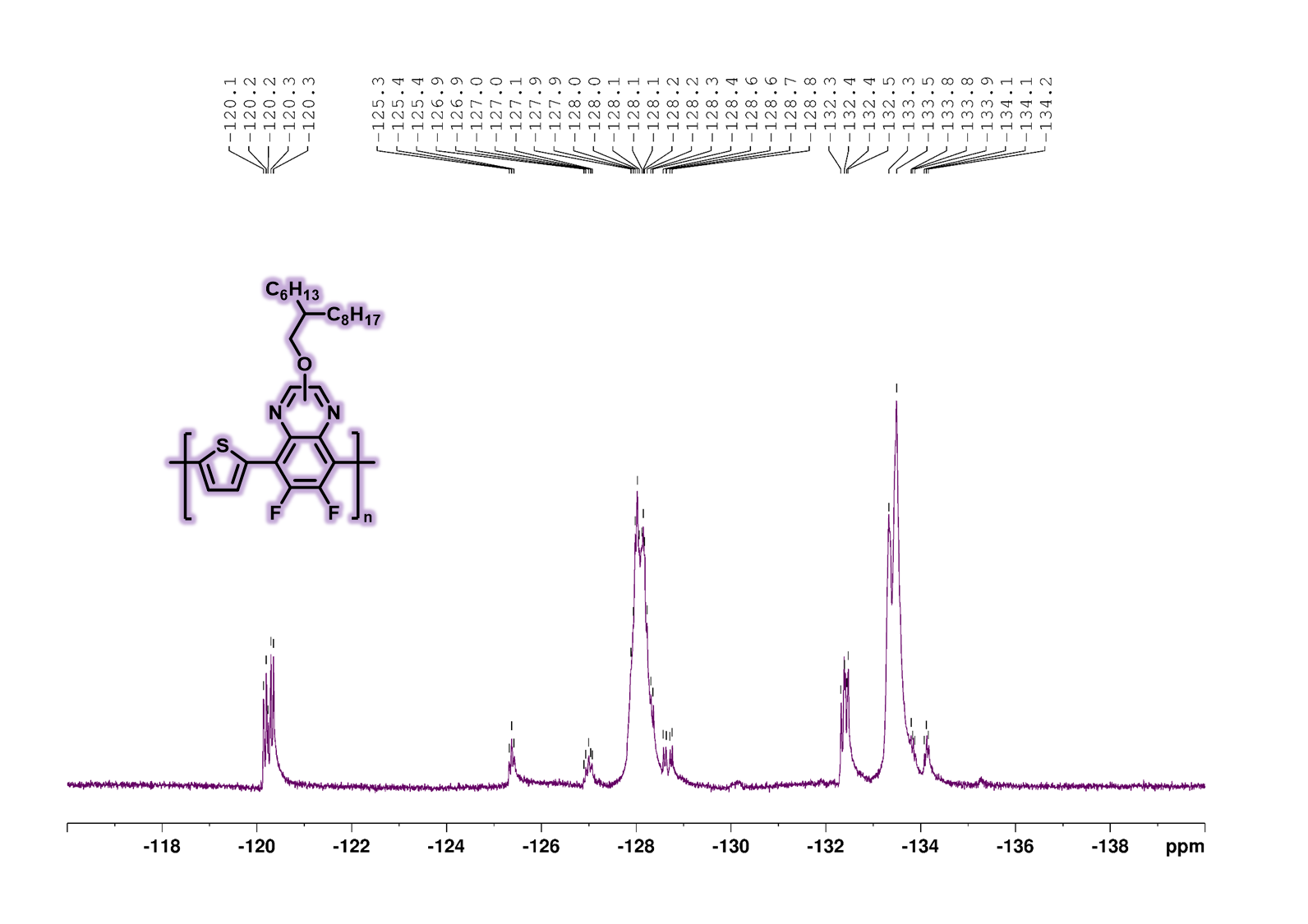


Figure S78. Zoomed ^19^F spectrum of PTQ10 in C_2_D_2_Cl_4_ at 393 K.


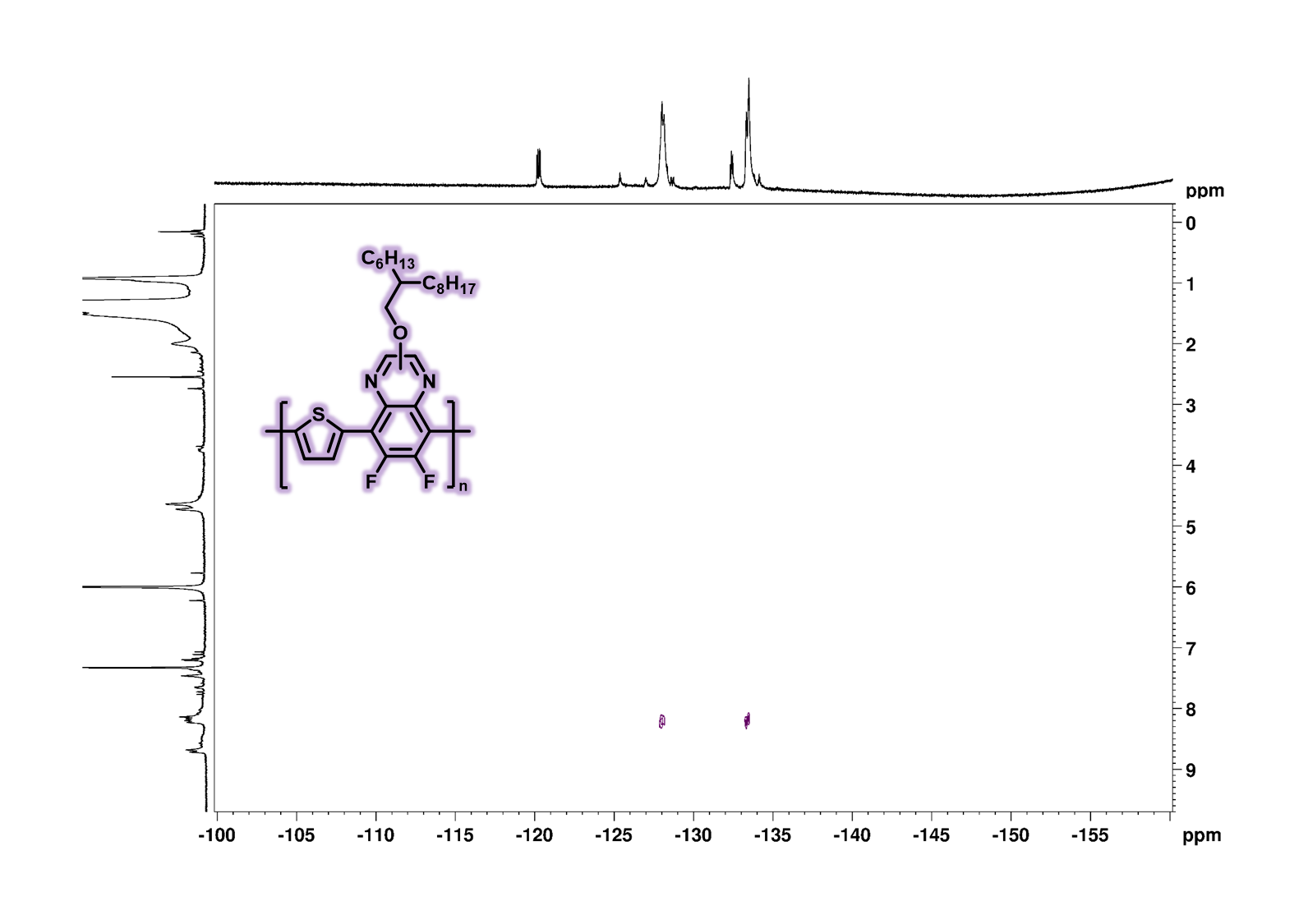


Figure S79. HOESY spectrum of PTQ10 in C_2_D_2_Cl_4_ at 298 K.


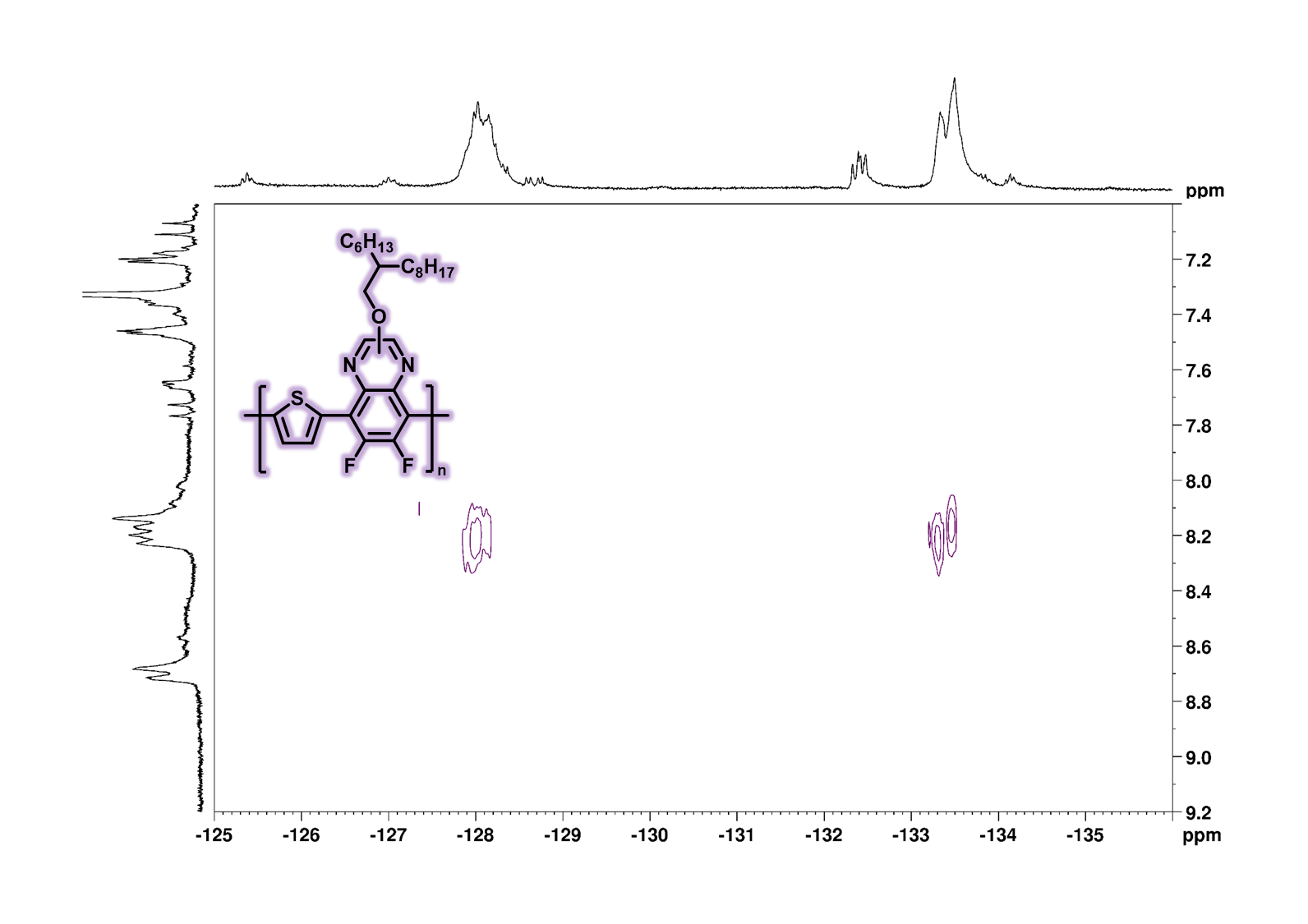


Figure S80. HOESY spectrum of PTQ10 in C_2_D_2_Cl_4_ at 298 K zoomed in the aromatic region.


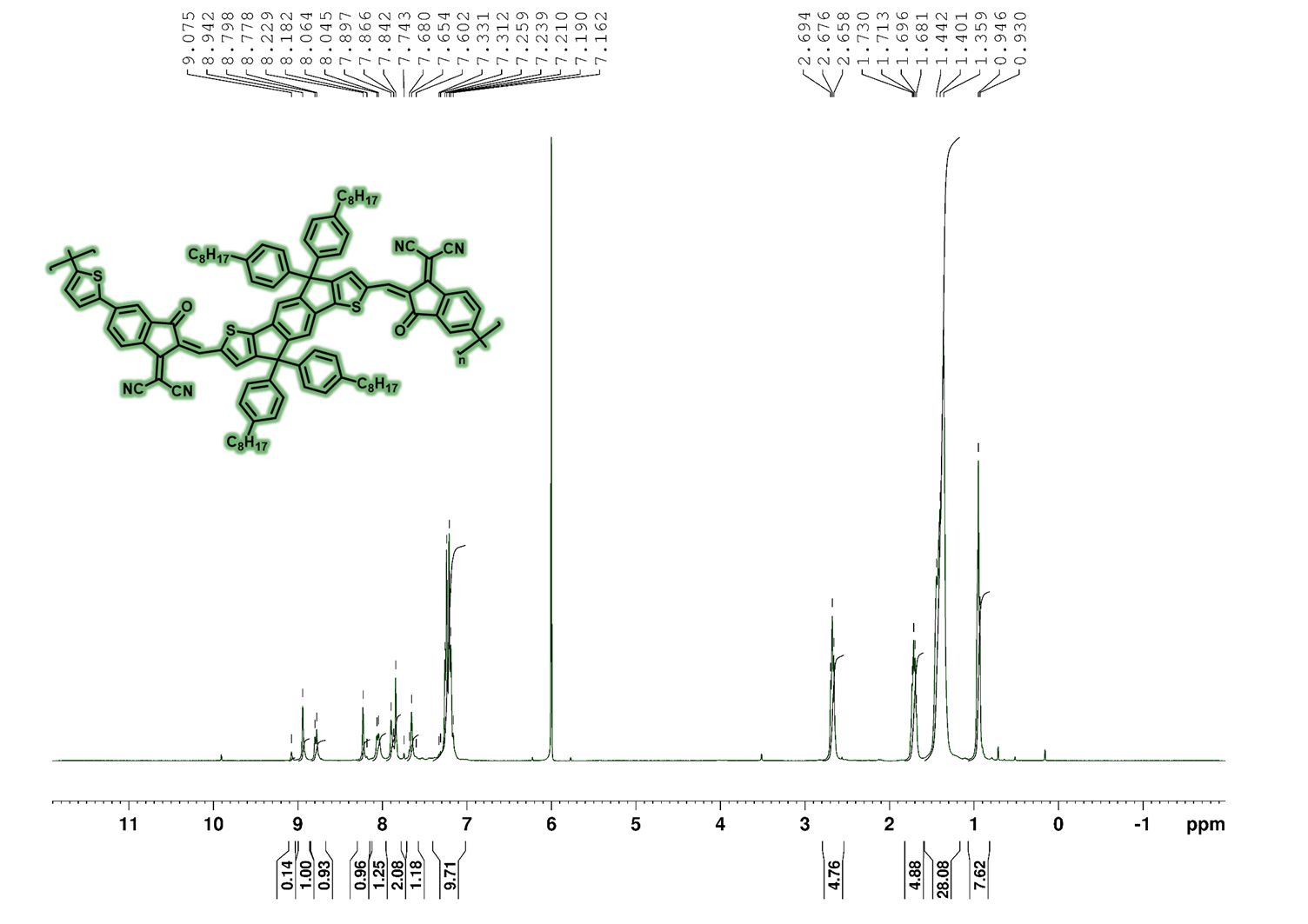


Figure S81. ^1^H NMR spectrum of PIDTe in C_2_D_2_Cl_4_ at 393 K.


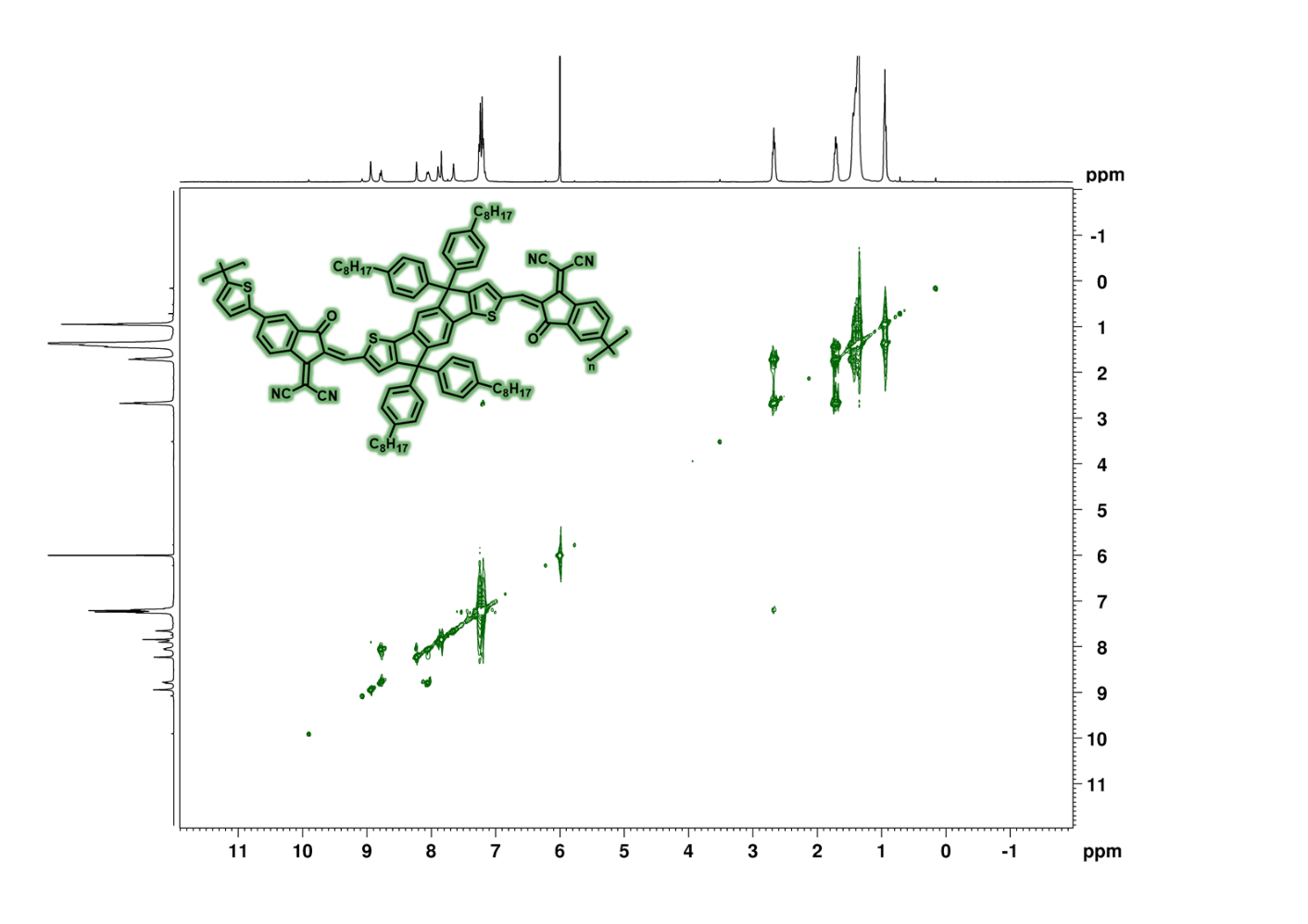


Figure S82. COSY spectrum of PIDTe in C_2_D_2_Cl_4_ at 393 K.


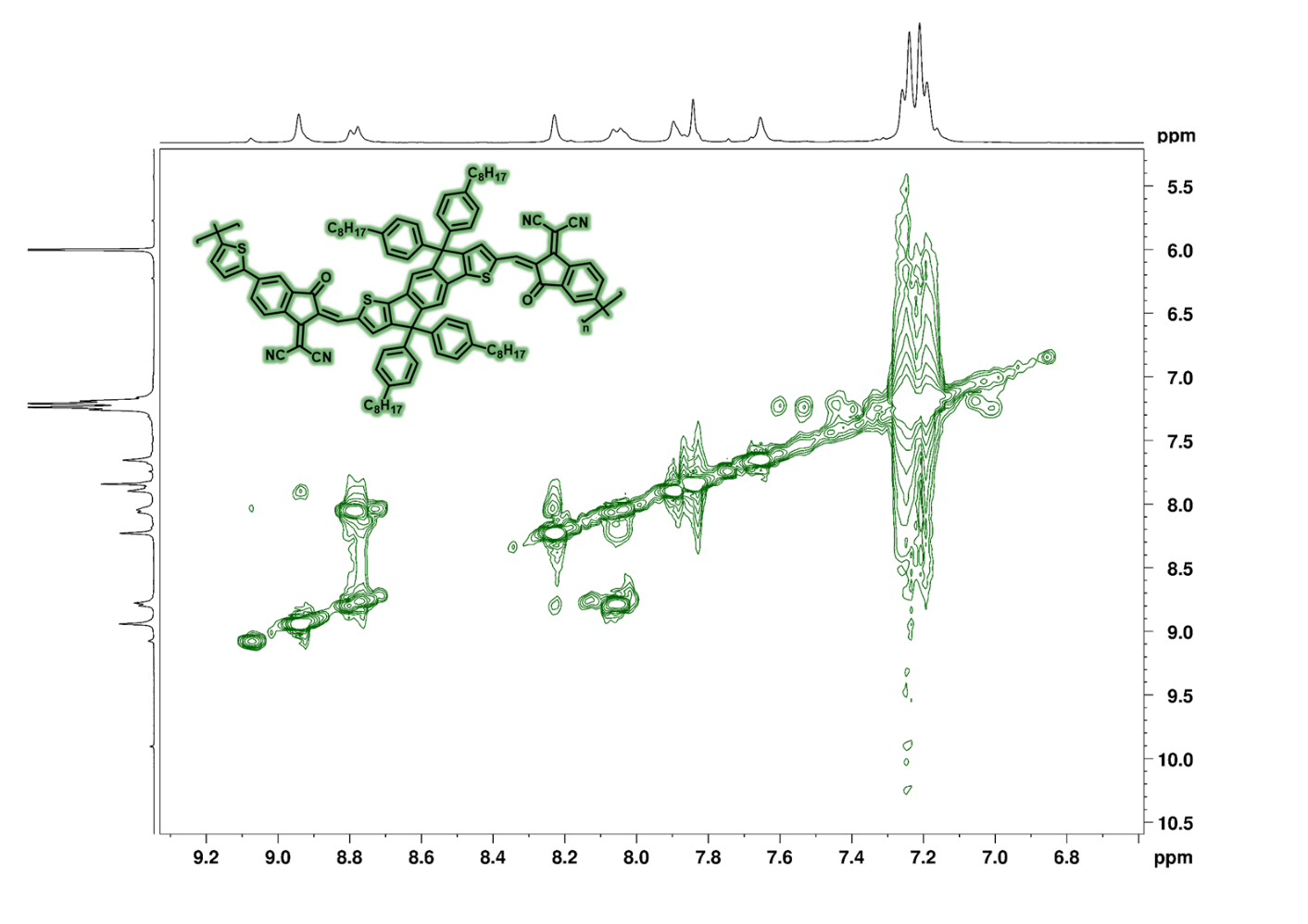


Figure S83. COSY spectrum of PIDTe in C_2_D_2_Cl_4_ at 393 K zoomed in the aromatic region.


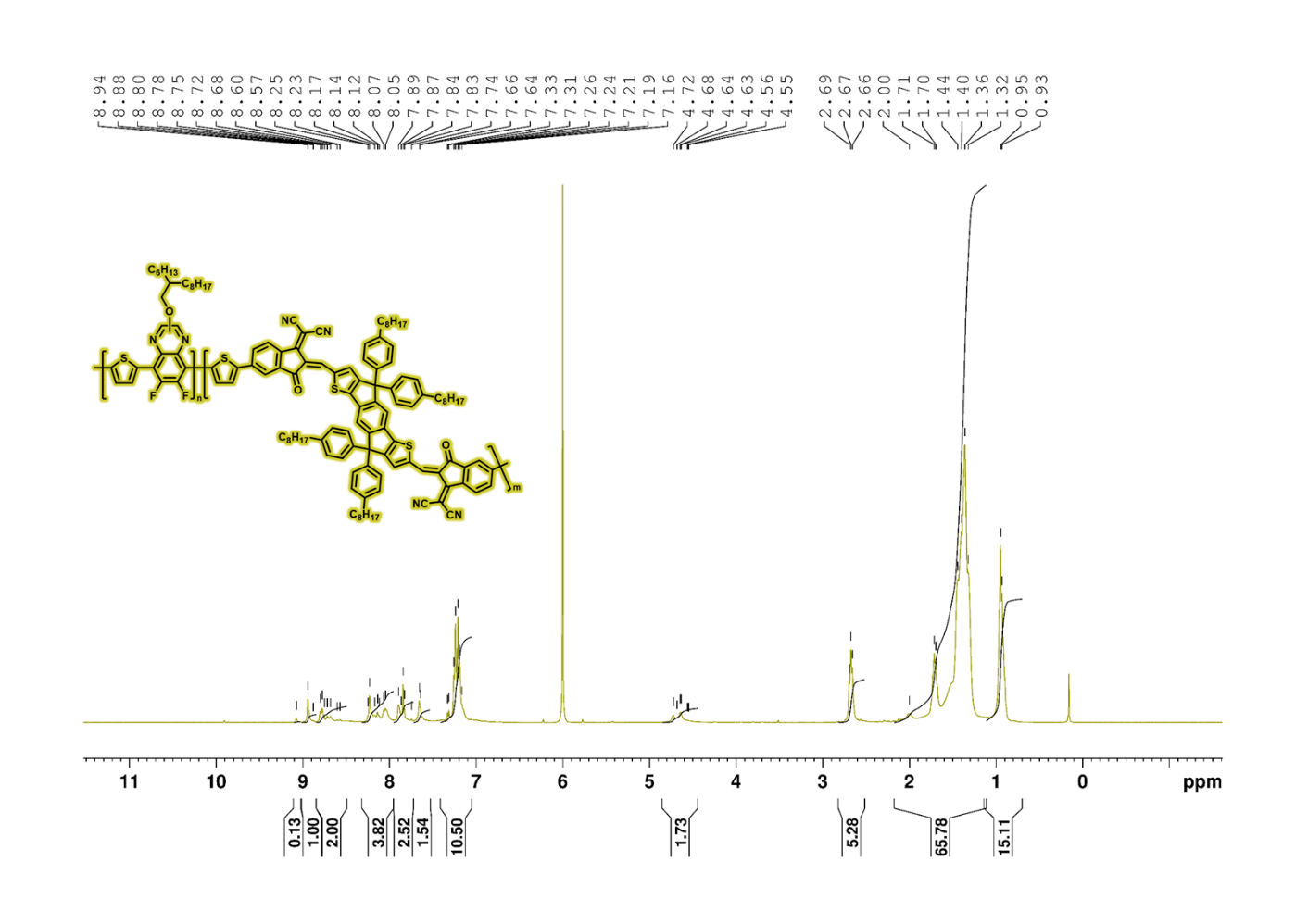


Figure S84. ^1^H NMR spectrum of TP-D in C_2_D_2_Cl_4_ at 393 K.


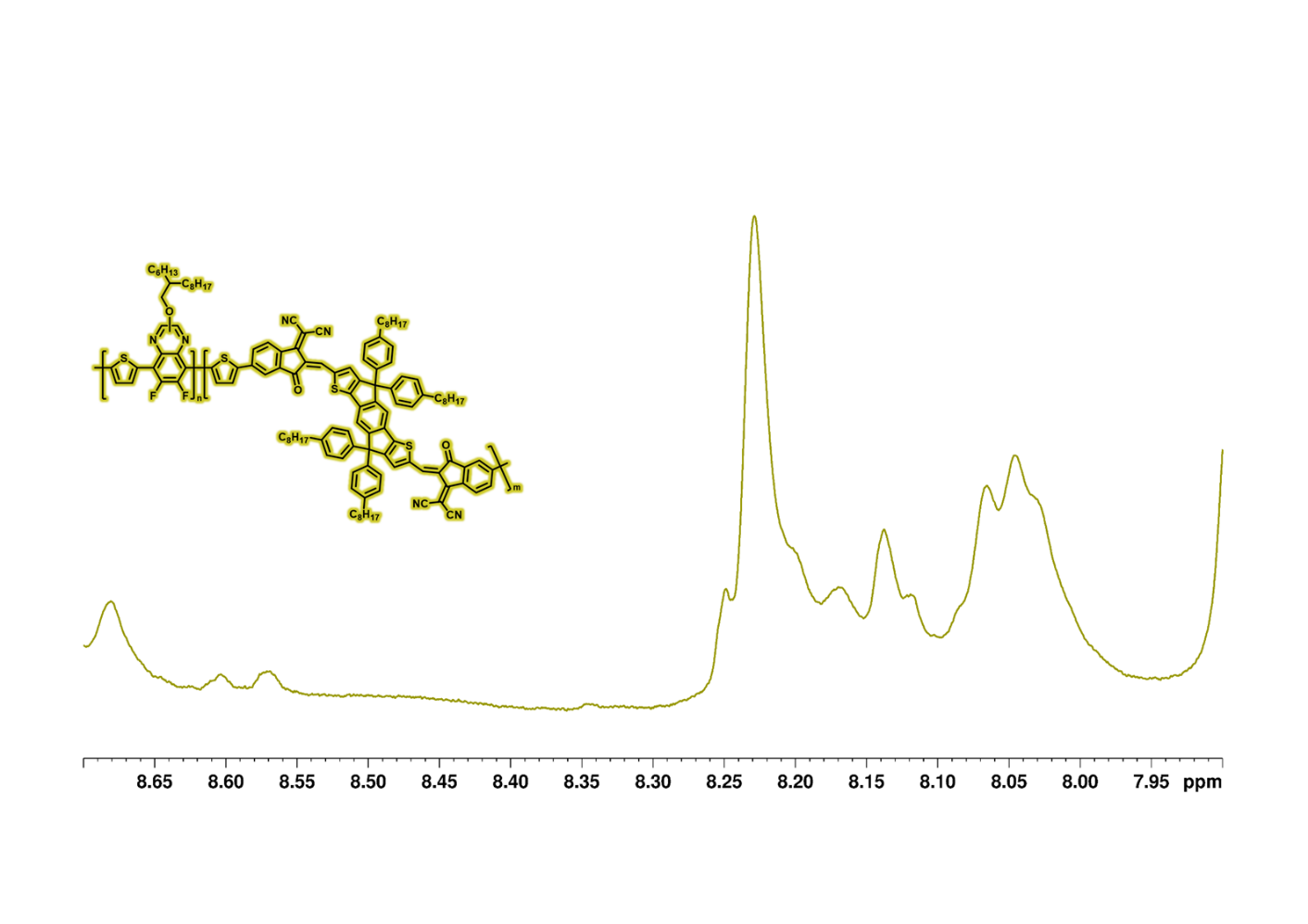


Figure S85. Zoomed ^1^H NMR spectrum of TP-D in C_2_D_2_Cl_4_ at 393 K.


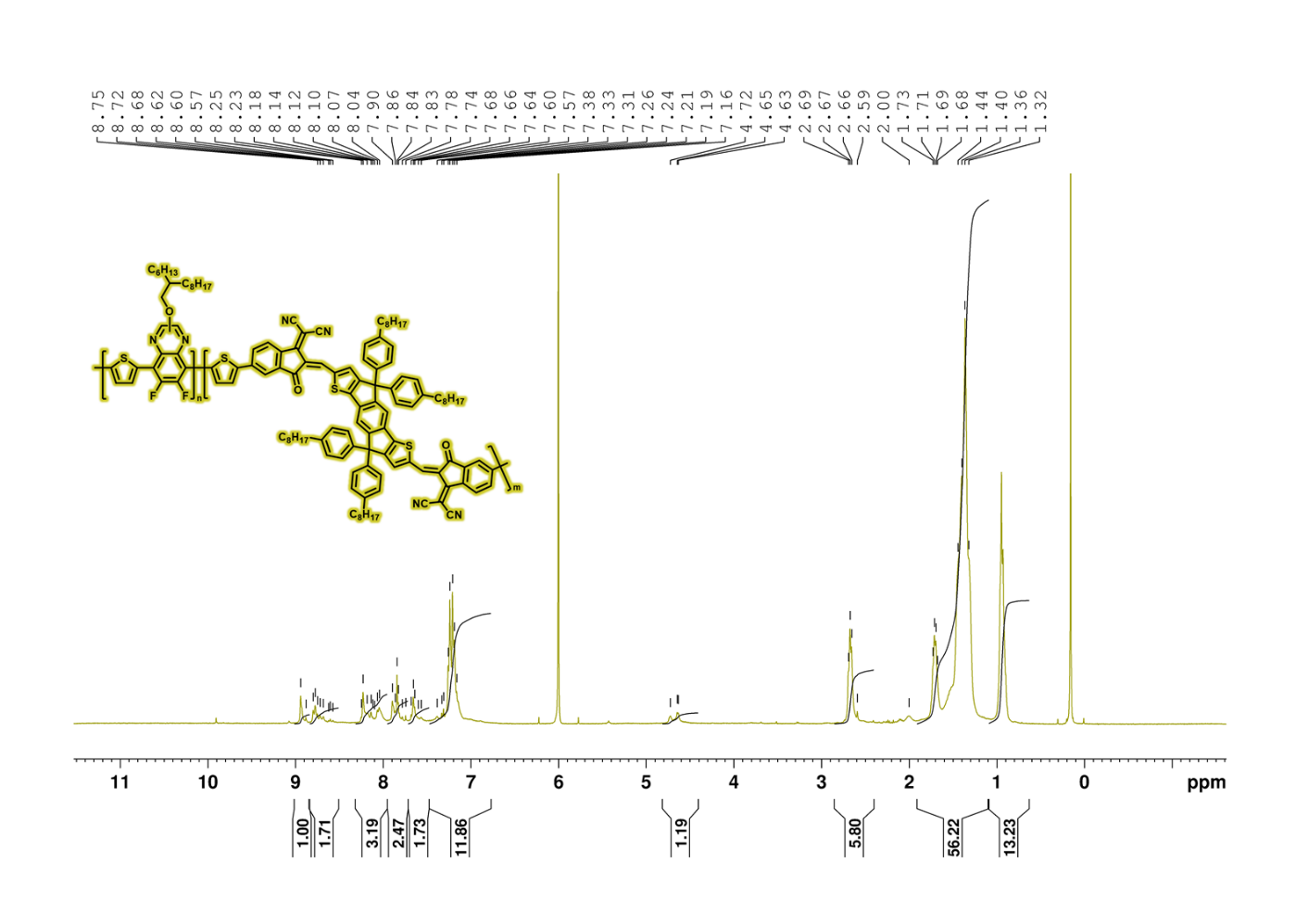


Figure S86. ^1^H NMR spectrum of the second TP-D synthesis in C_2_D_2_Cl_4_ at 393 K.


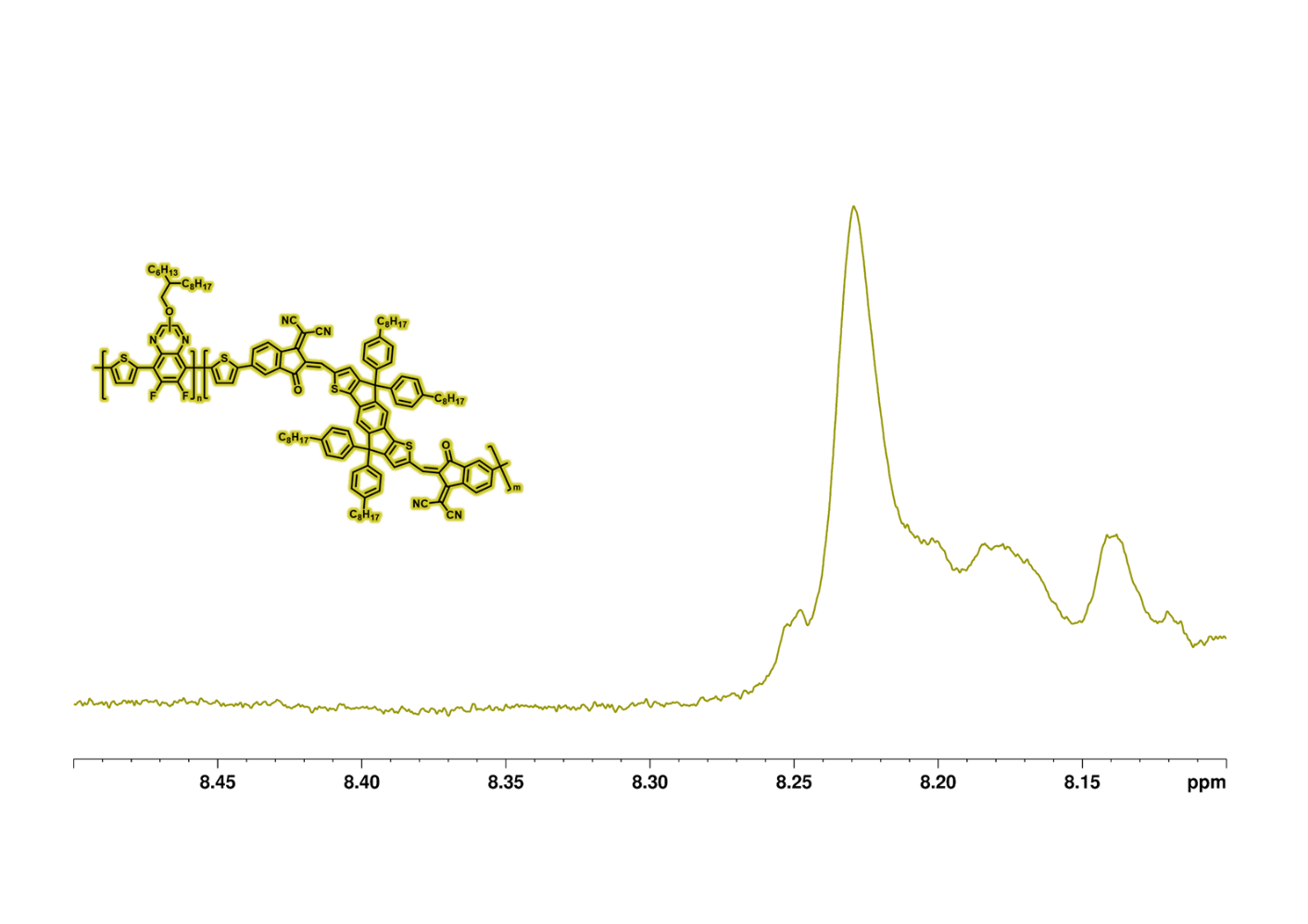


Figure S87. Zoomed ^1^H NMR spectrum of the second TP-D synthesis in C_2_D_2_Cl_4_ at 393 K.


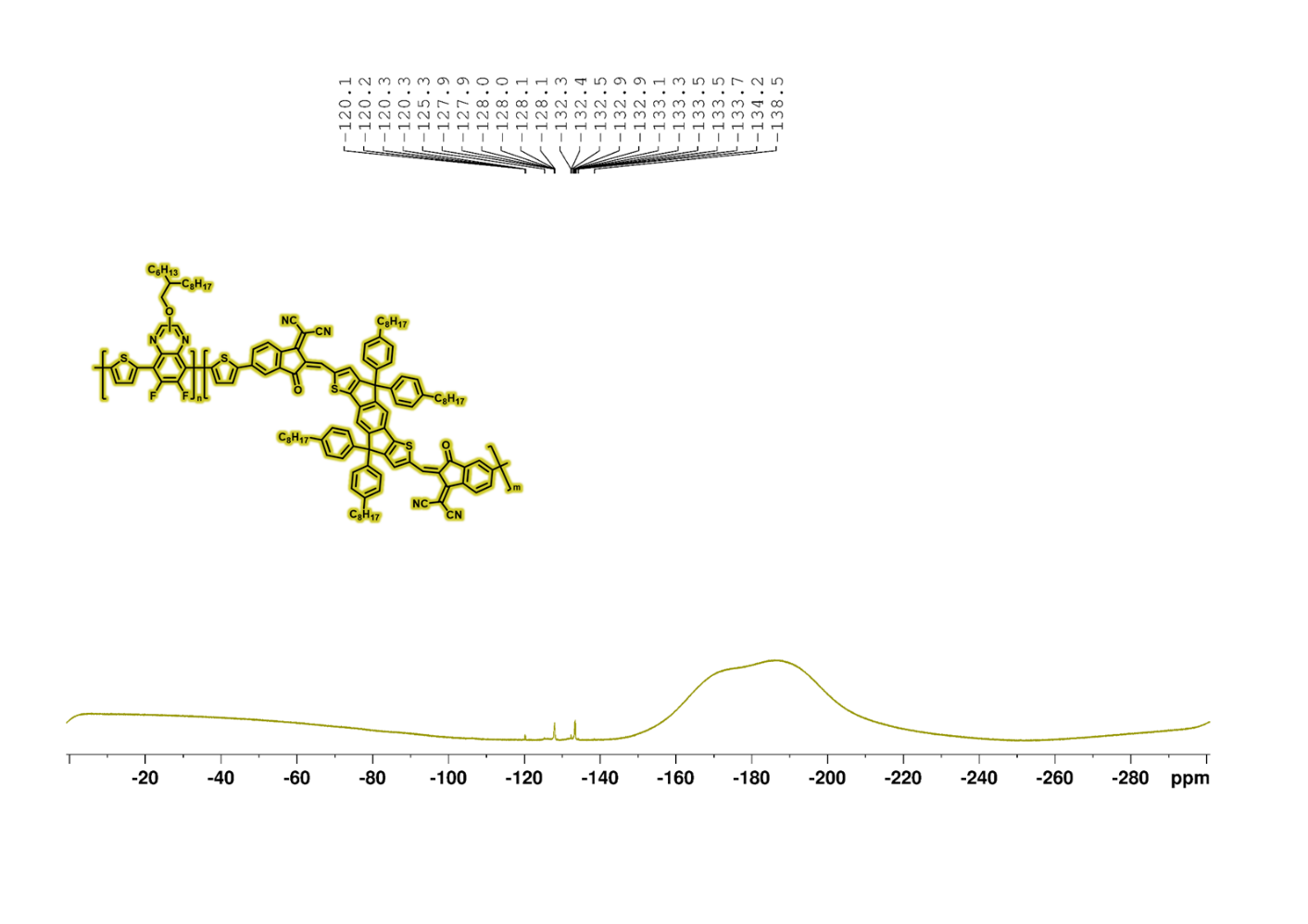


Figure S88. ^19^F NMR spectrum of TP-D in C_2_D_2_Cl_4_ at 393 K.


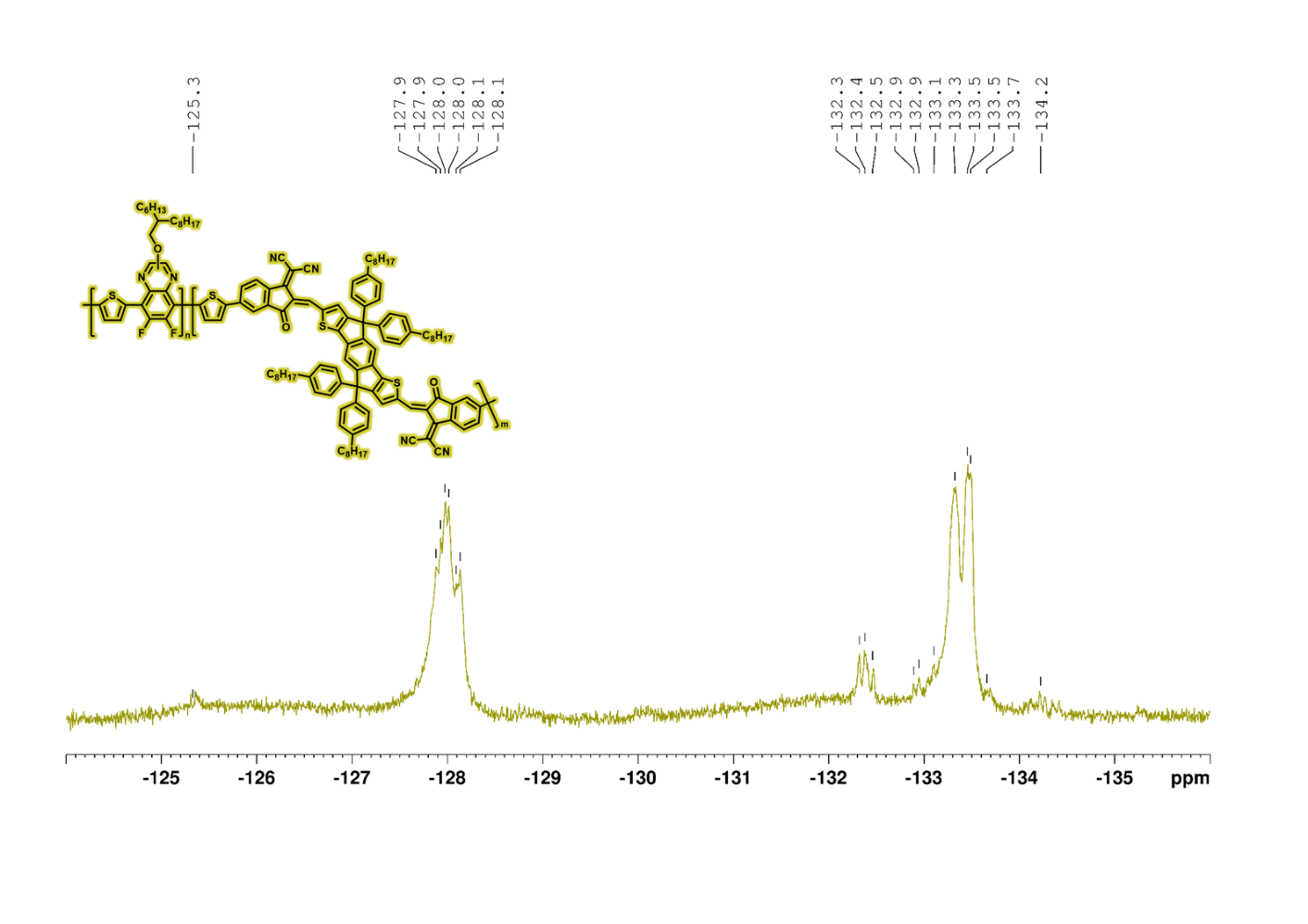


Figure S89. Zoomed ^19^F NMR spectrum of TP-D in C_2_D_2_Cl_4_ at 393 K.


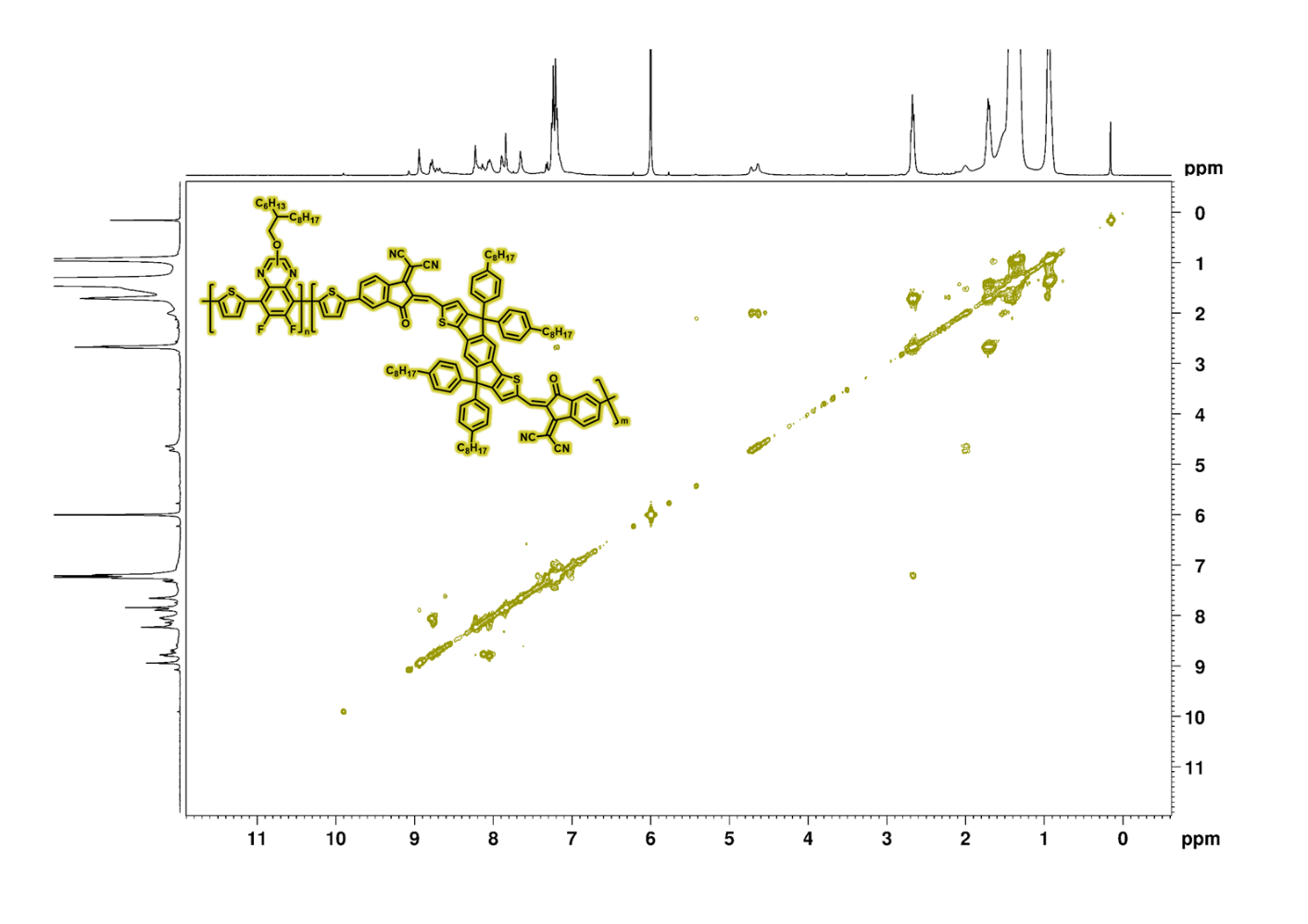


Figure S90. COSY spectrum of TP-D in C_2_D_2_Cl_4_ at 393 K.


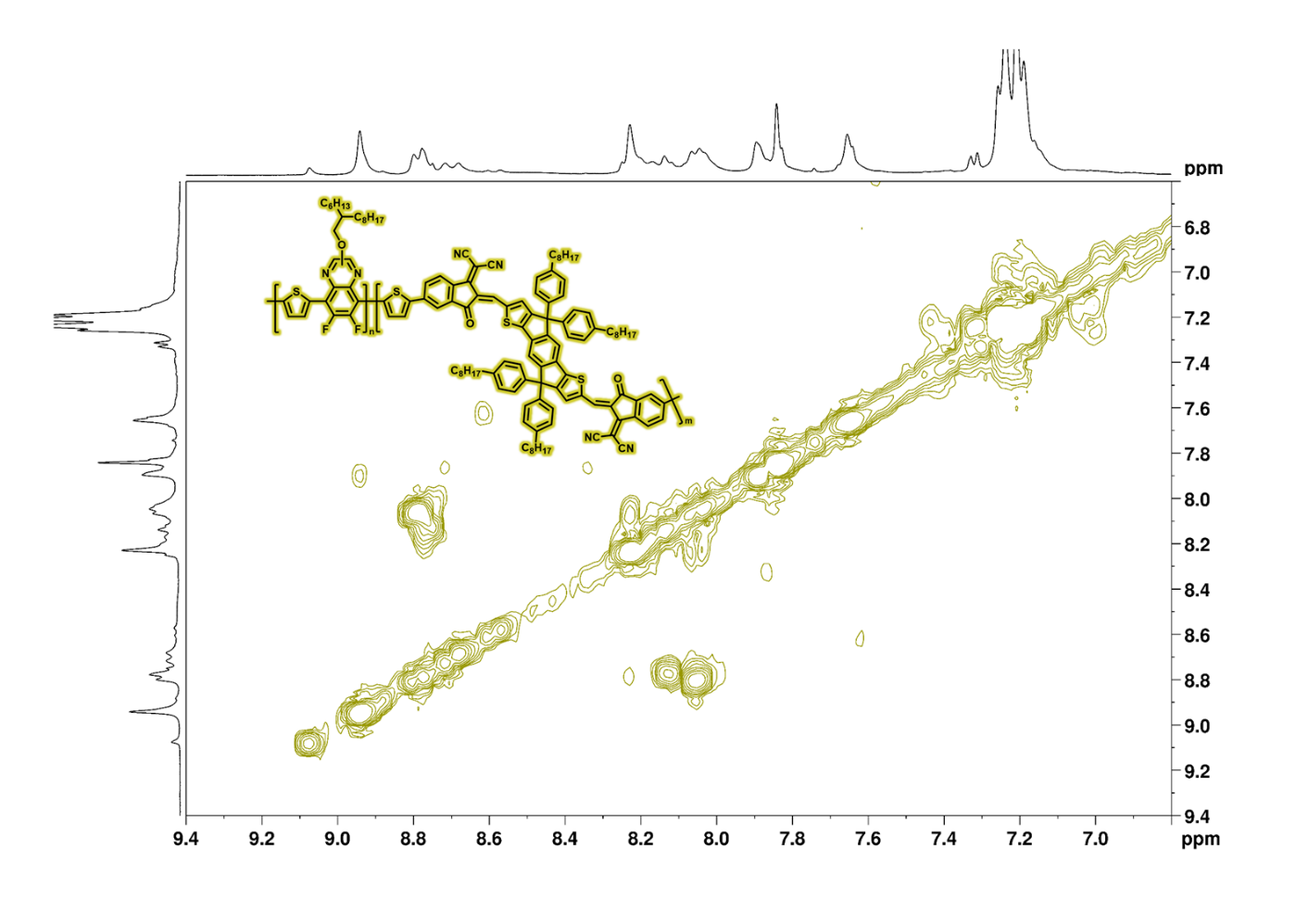


Figure S91. COSY spectrum of TP-D in C_2_D_2_Cl_4_ at 393 K zoomed in the aromatic region.


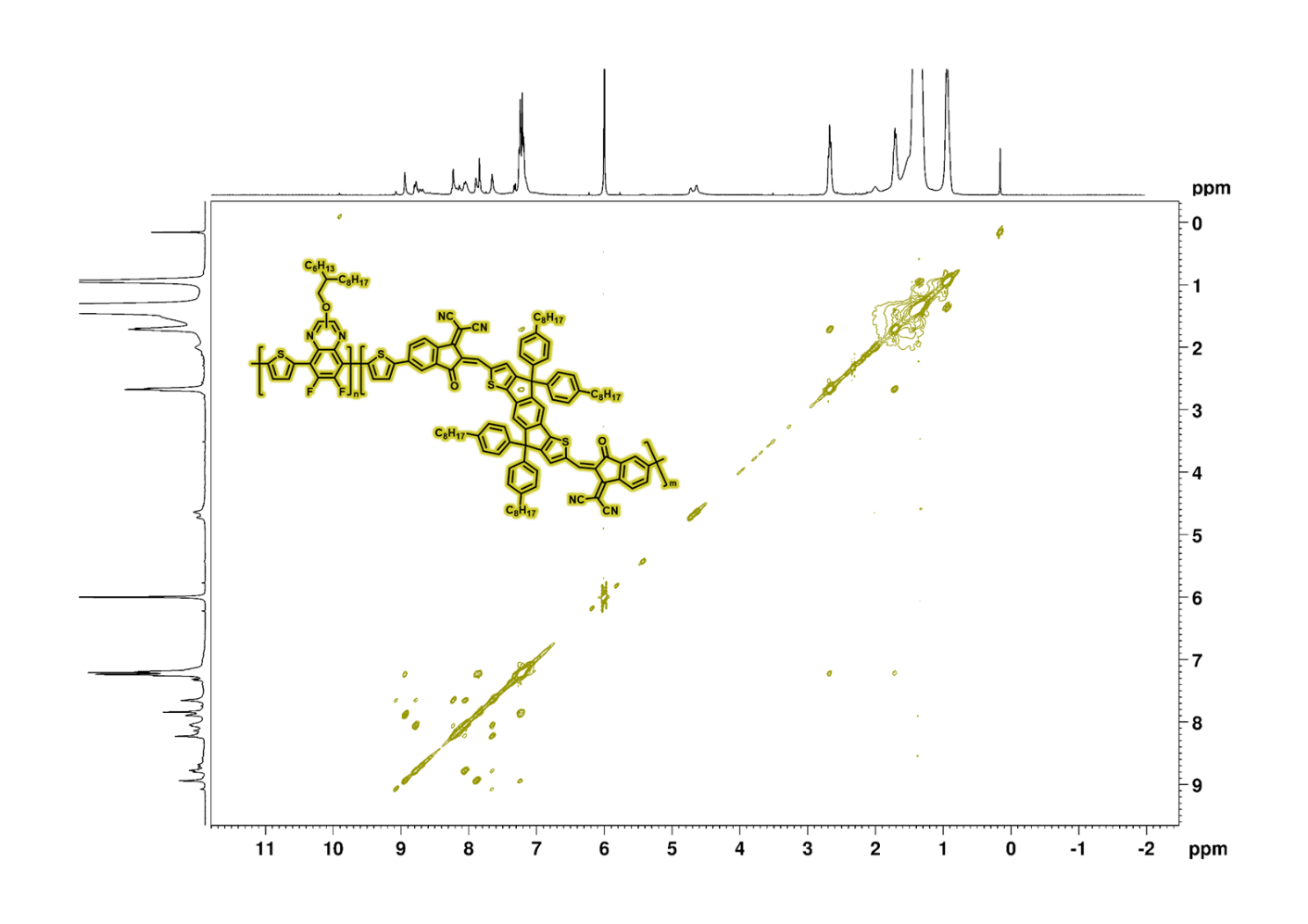


Figure S92. NOESY spectrum of TP-D in C_2_D_2_Cl_4_ at 393 K.


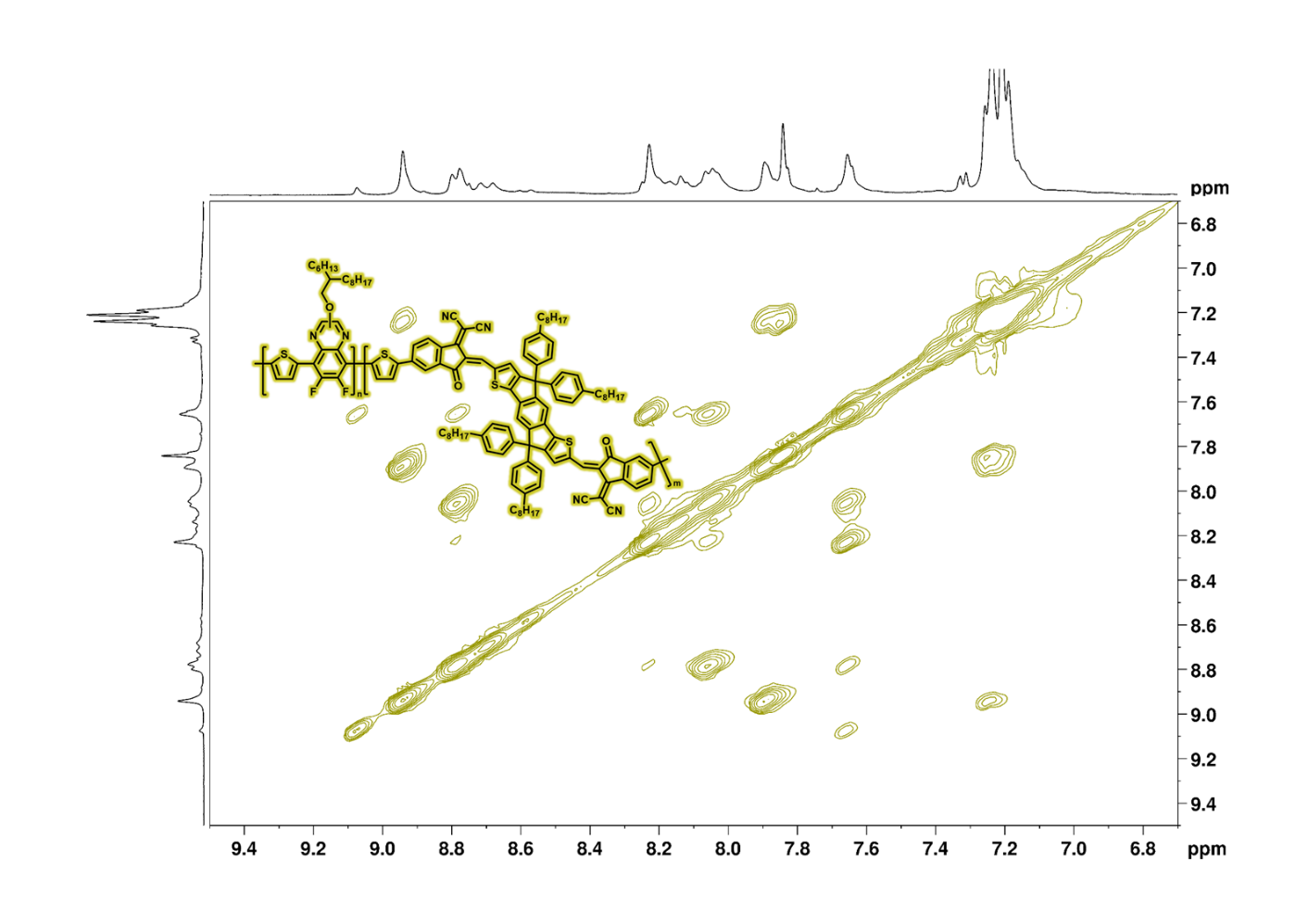


Figure S93. NOESY spectrum of TP-D in C_2_D_2_Cl_4_ at 393 K zoomed in the aromatic region.


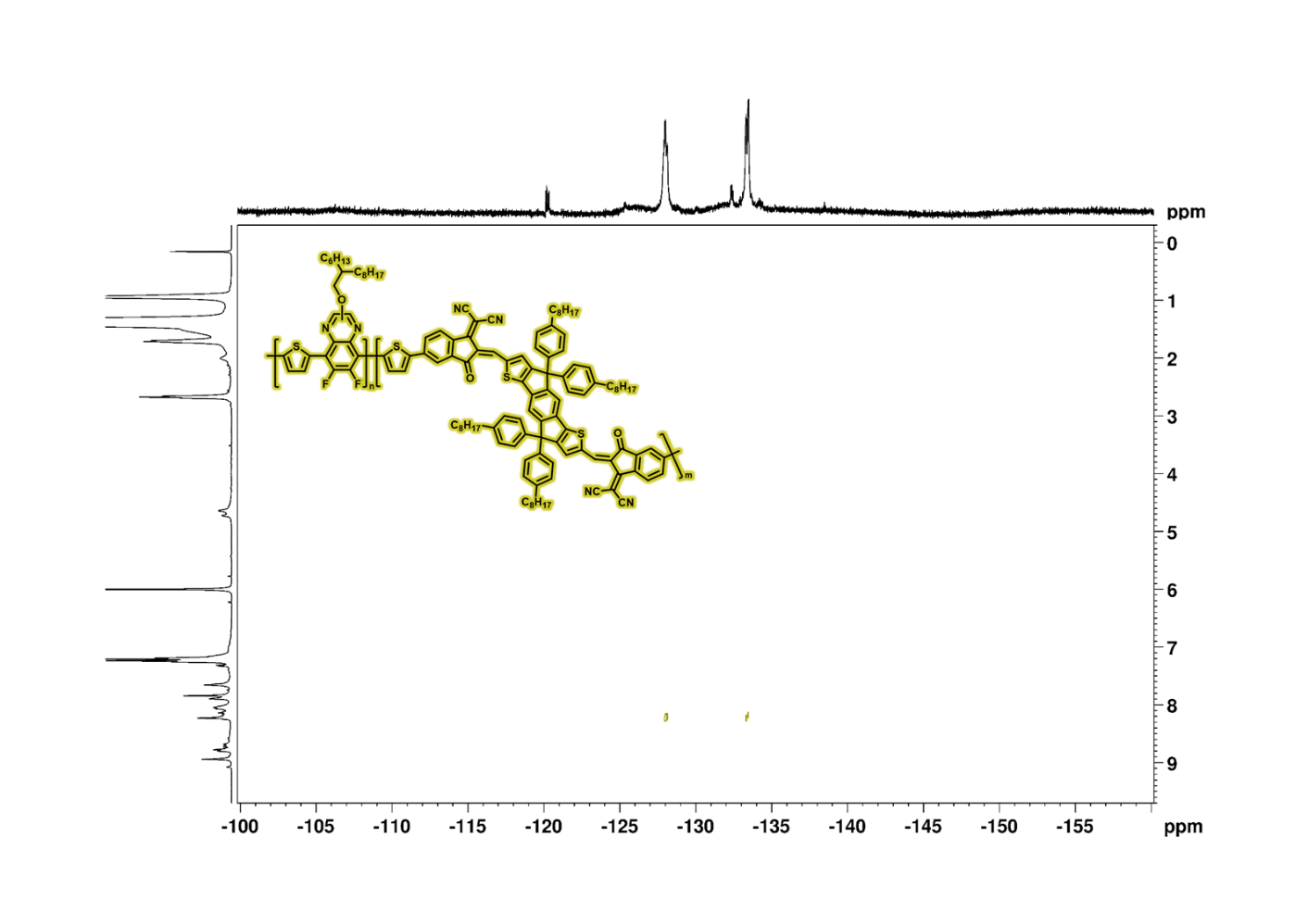


Figure S94. HOESY spectrum of TP-D in C_2_D_2_Cl_4_ at 393 K.


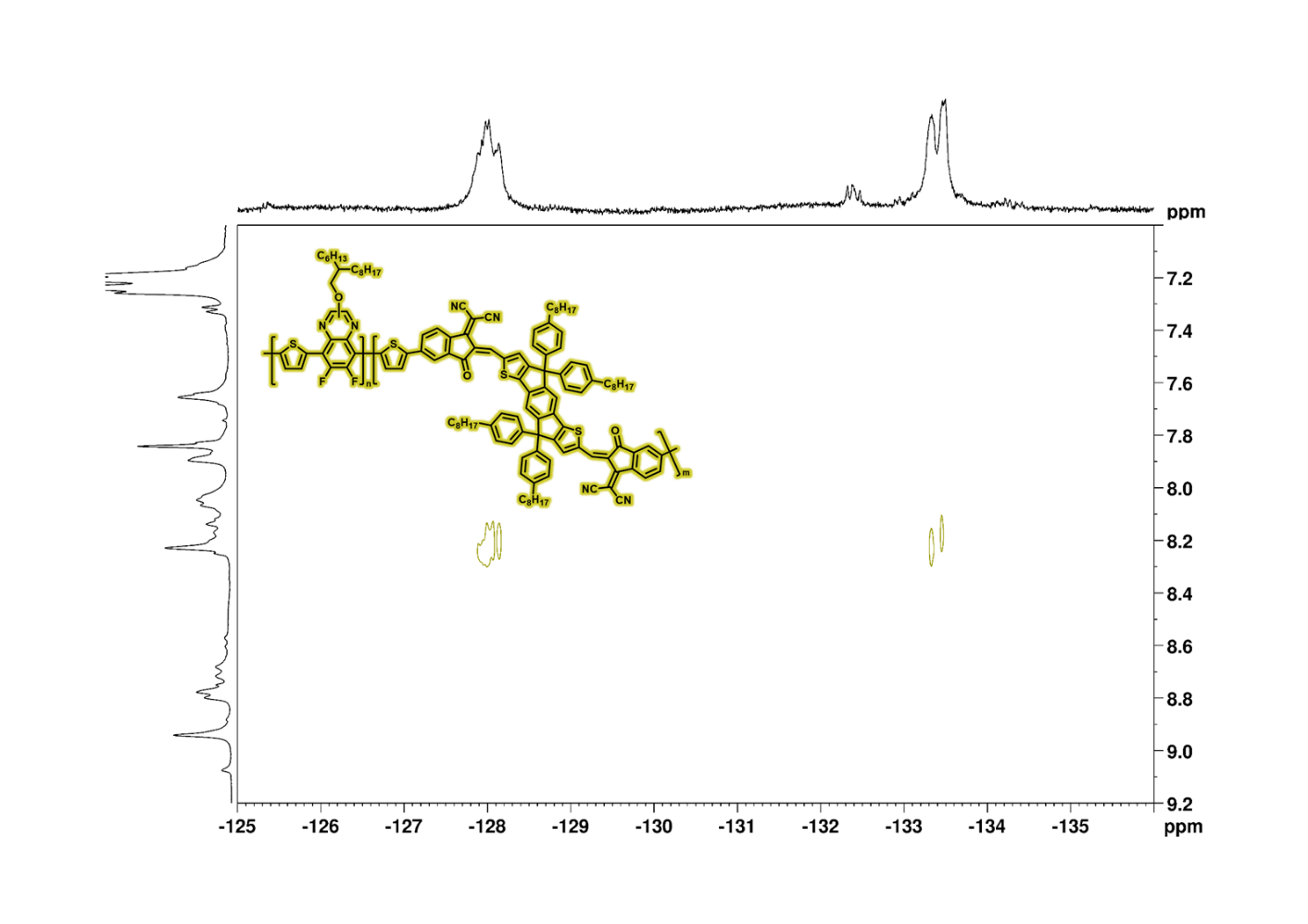


Figure S95. HOESY spectrum of TP-D in C_2_D_2_Cl_4_ at 393 K zoomed in the aromatic region.


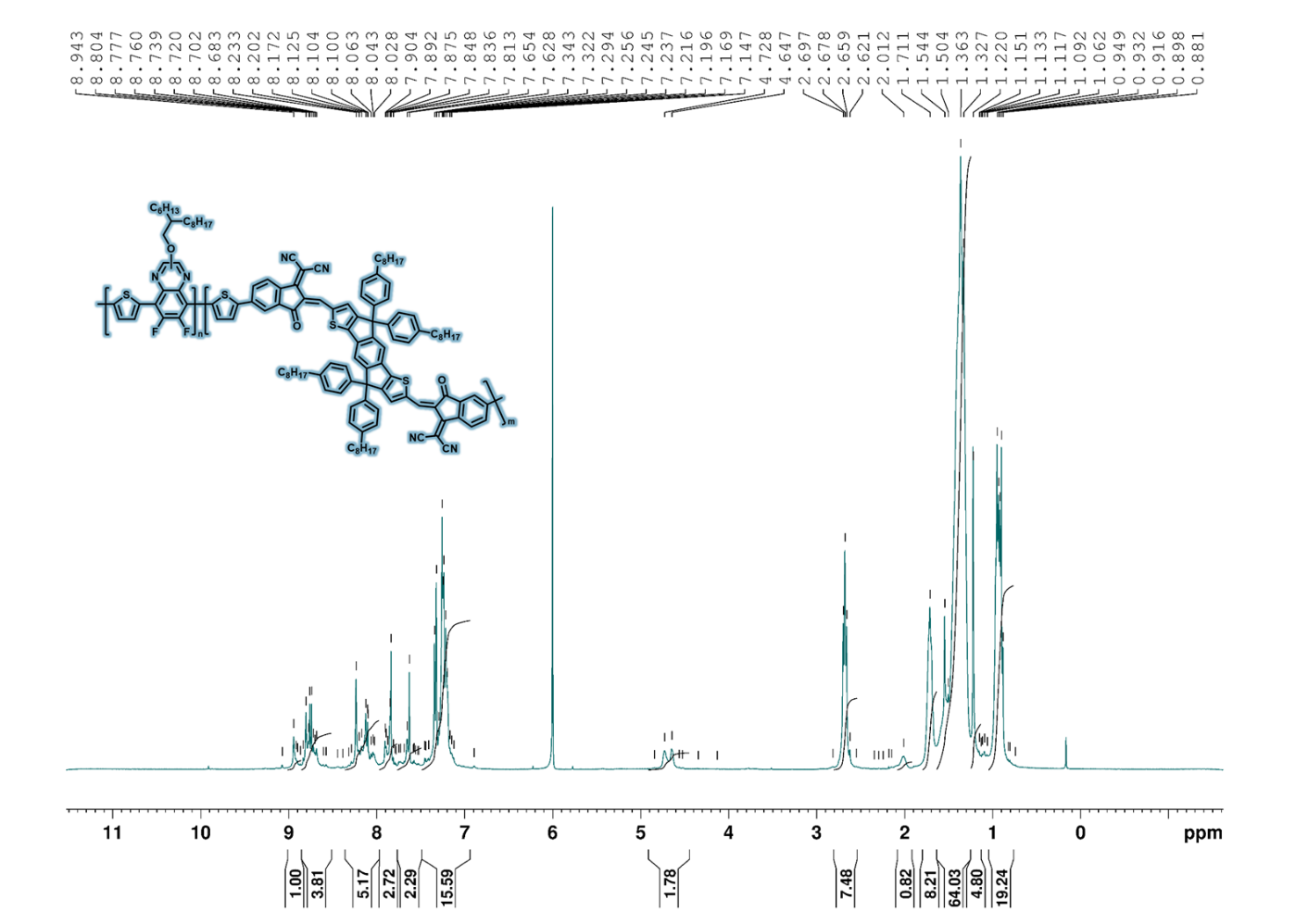


Figure S96. ^1^H spectrum of OP-D in C_2_D_2_Cl_4_ at 393 K.


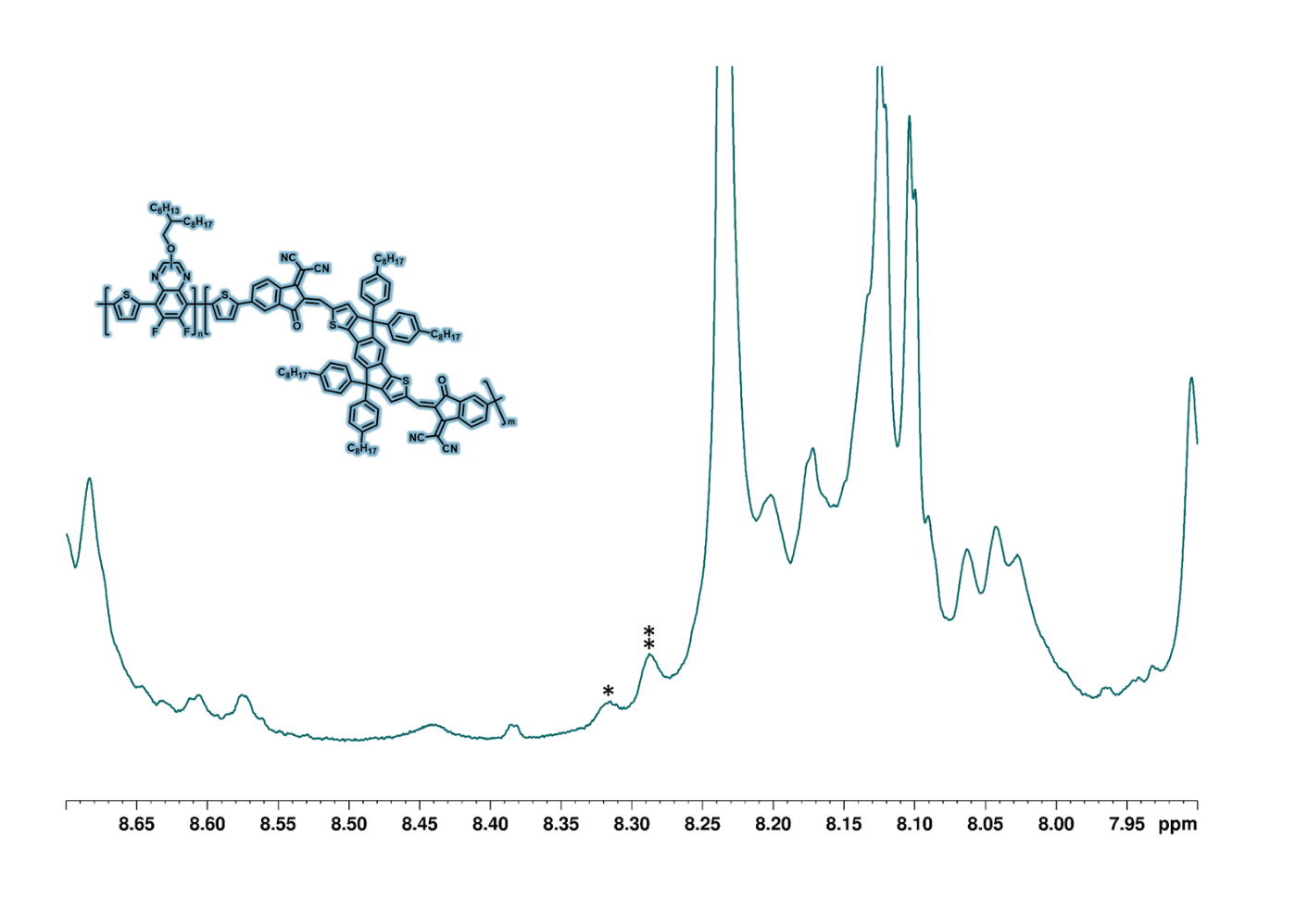


Figure S97. Zoomed ^1^H spectrum of OP-D in C_2_D_2_Cl_4_ at 393 K.


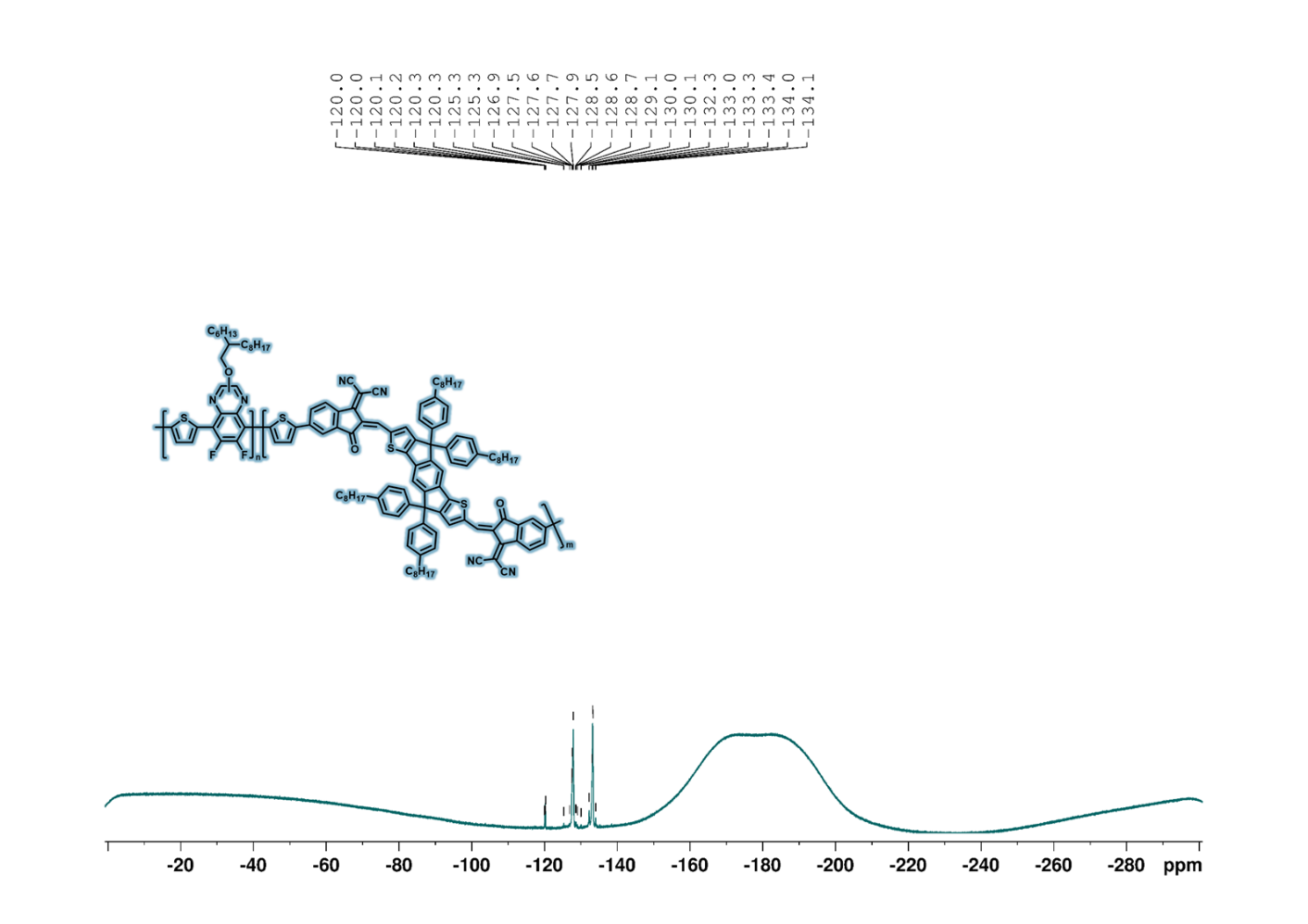


Figure S98. ^19^F spectrum of OP-D in C_2_D_2_Cl_4_ at 393 K.


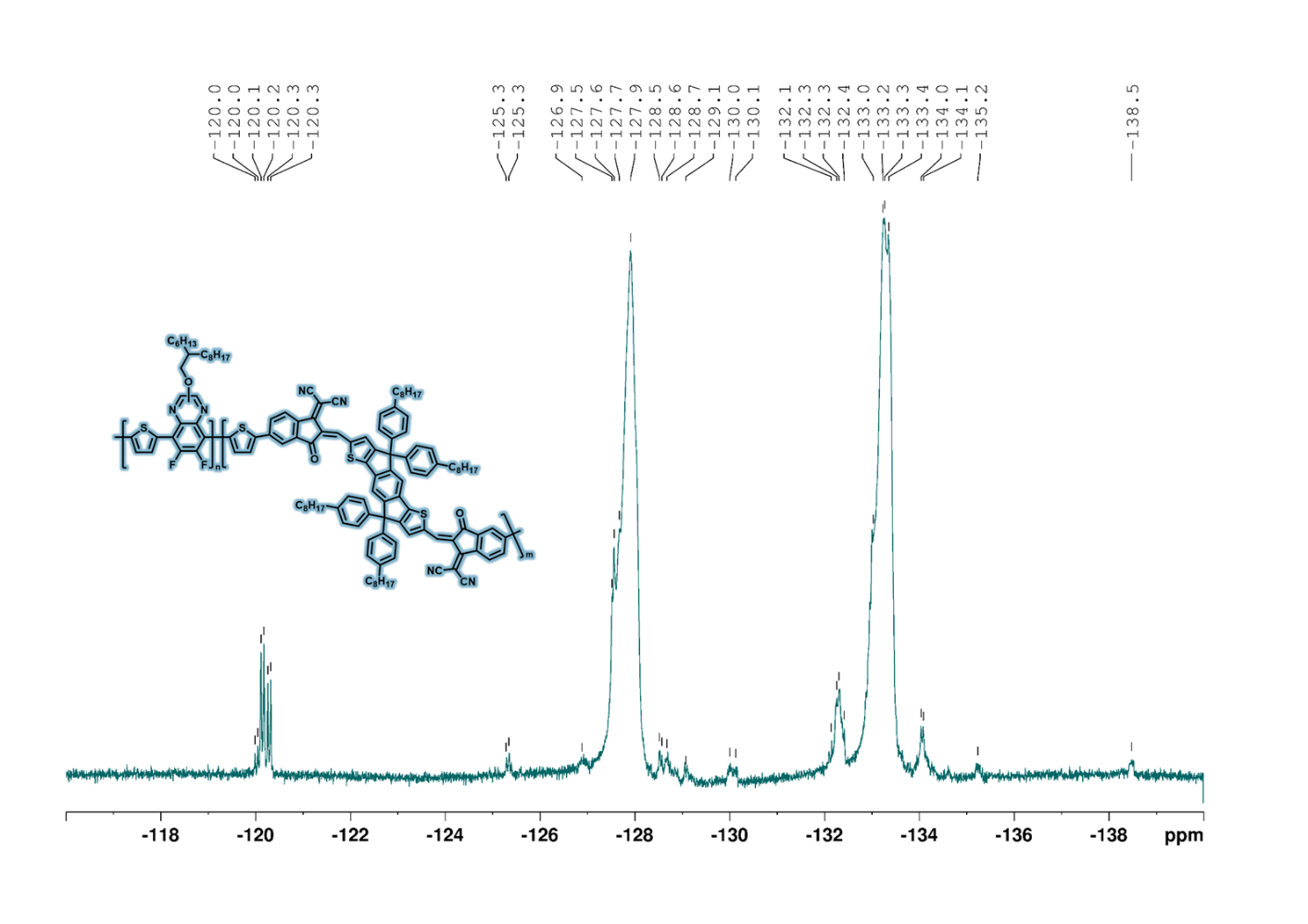


Figure S99. Zoomed ^19^F spectrum of OP-D in C_2_D_2_Cl_4_ at 393 K.

Figure S100. COSY spectrum of OP-D in C_2_D_2_Cl_4_ at 393 K.

Figure S101. COSY spectrum of OP-D in C_2_D_2_Cl_4_ at 393 K zoomed in the aromatic region.

Figure S102. NOESY spectrum of OP-D in C_2_D_2_Cl_4_ at 393 K.

Figure S103. NOESY spectrum of OP-D in C_2_D_2_Cl_4_ at 393 K zoomed in the aromatic region.

Figure S104. HSQC spectrum of OP-D in C_2_D_2_Cl_4_ at 393 K.

Figure S105. HSQC spectrum of OP-D in C_2_D_2_Cl_4_ at 393 K zoomed in the aromatic region.

Figure S106. HMBC spectrum of OP-D in C_2_D_2_Cl_4_ at 393 K.

Figure S107. HMBC spectrum of OP-D in C_2_D_2_Cl_4_ at 393 K zoomed in the aromatic region.

Figure S108. HOESY spectrum of OP-D in C_2_D_2_Cl_4_ at 393 K.

Figure S109. HOESY spectrum of OP-D in C_2_D_2_Cl_4_ at 393 K zoomed in the aromatic region.

Figure S110. ^1^H spectrum of OP-C in C_2_D_2_Cl_4_ at 393 K.

Figure S111. Zoomed ^1^H spectrum of OP-C in C_2_D_2_Cl_4_ at 393 K.

Figure S112. ^19^F spectrum of OP-C in C_2_D_2_Cl_4_ at 393 K.

Figure S113. Zoomed ^19^F spectrum of OP-C in C_2_D_2_Cl_4_ at 393 K.

Figure S114. COSY spectrum of OP-C in C_2_D_2_Cl_4_ at 393 K.

Figure S115. COSY spectrum of OP-C in C_2_D_2_Cl_4_ at 393 K zoomed in the aromatic region.

Figure S116. ^1^H spectrum of PTQ10-*b*-PITIC in C_2_D_2_Cl_4_ at 393 K.

Figure S117. ^19^F spectrum of PTQ10-*b*-PITIC in C_2_D_2_Cl_4_ at 393 K.

Figure S118. Zoomed ^19^F spectrum of PTQ10-*b*-PITIC in C_2_D_2_Cl_4_ at 393 K.

Figure S119. ^1^H spectrum of PTQ10-*b*-PL8-BO in C_2_D_2_Cl_4_ at 393 K.

Figure S120. ^19^F spectrum of PTQ10-*b*-PL8-BO in C_2_D_2_Cl_4_ at 393 K.

Figure S121. Zoomed ^19^F spectrum of PTQ10-*b*-PL8-BO in C_2_D_2_Cl_4_ at 393 K.

# NMR correlations for the principal signals

Figure S122. 3↔4 COSY correlation for AJC1, AJC2, OP-D and TP-D in C_2_D_2_Cl_4_ at 393 K.

Figure S123. 4↔5 COSY correlation for AJC1, AJC2, OP-D and TP-D in C_2_D_2_Cl_4_ at 393 K.

Figure S124. HSQC correlation of proton 3 or equivalent for AJC1, AJC2 and OP-D in C_2_D_2_Cl_4_ at 393 K zoomed in the aromatic region.

Figure S125. HMBC correlations of proton 3 or equivalent for AJC1, AJC2, OP-D and TP-D in C_2_D_2_Cl_4_ at 393 K zoomed in the aromatic region.

# NMR signal deconvolution analysis

## Supplementary Note 1: guidelines for NMR signal deconvolution

By definition, the resulting NMR line shape of a molecule produced by the Fourier transform of its spectrum is Lorentzian.^[5–7]^ In rigid systems, gaussian line shapes can be observed. In practice, solution-state NMR can be accurately modeled by Lorentzian function, while Gaussian fittings are more appropriate for solid-state NMR.^[7]^

*With TopSpin 4.4.1.*

The deconvolution of NMR signals can be achieved using the “dcon” module in the software. Accurate deconvolution of signals can be achieved with the following the guidelines: 1. Select the peaks to analyze and register them as the reference signals for the deconvolution. If these signals overlap with neighboring signals, select these as well. 2. Set the 'Default computation range per line' parameter (AZWF) to 0.1 ppm. In practice, this should correspond to approximately 5-10 times the half width of the signals. Also, set the percentage of Gaussian fitting to 0 for each signal. 3. Run a preliminary fitting test and assess the quality of the fit. 4. If necessary, manually adjust the frequency half width of the signals. Large signals may require larger half widths. Ensure that the AZWF parameter is still within the correct range of values. 5. The best fit can be achieved through trial and error, by gradually optimizing the half widths of the signals to minimize the standard deviation of their intensity and width.

*With Mestrenova 6.0 and above.*

Similar results to that obtained using TopSpin can be achieved using the Global Spectral Deconvolution (GSD) module in Mestrenova. Accurate deconvolution of the signals can be achieved by selecting the highest refinement and resolution level. This machine-learning method enables the entire spectrum to be fitted all at once. As shown in Table S7, the results provided by this method are comparable to the ones given by TopSpin. However, Mestrenova does not allow to extract uncertainties on the area calculations. In addition, due to the low intensity of its heterojunction signals, PTQ10-*b*-PL8-BO could not be analyzed using this method.

## Supplementary Note 2: NoH calculation example for OP-C and block length recalibration

Using Equation 1 and Equation 2 in the main text, the NoH of the copolymers can be trivially calculated from the area ratios extracted from their ^1^H NMR spectrum (Table S4).

For **OP-C**:

- $A_{D}$/$A_{H}$=1/0.114≈8.772
- $A_{A'}$/$A_{H}$=1/1.278≈0.782

Assuming that for two species $i$ and $j$, their area ratio $A_{i}$/$A_{j}$ is equal to their molar ratio (or number of respective molecule) $N_{i}$/$N_{j}$, Equation 2 can be rewritten as:

$M_{n}^{\mathrm{copo}}=N_{D}M_{D}$+${N'}_{A}{M'}_{A}+N_{H}M_{H}$

$M_{n}^{\mathrm{copo}}={8.772N}_{H}M_{D}$+${0.782N}_{H}{M'}_{A}+N_{H}M_{H}$

Therefore $N_{H}$ (or NoH) can be expressed as a function of the other known quantities:

$$N_{H}=\frac{M_{n}^{\mathrm{copo}}}{8.772M_{D}+0.782{M'}_{A}+M_{H}}$$

For **OP-C**, $M_{n}^{\mathrm{copo}}$=28550 g/mol, $M_{D}$=486.67 g/mol and ${M'}_{A}$=$M_{H}$=1508.11 g/mol. Therefore:

$$N_{H}=\frac{28550}{8.772*486.67+0.782*1508.11+1508.11}=4.1$$

Using this result, the NoH can be used to recalculate the average size of each block in the material. By definition, the number of blocks is equal to NoH+1, which raises the number of block of **OP-C** to 5.1. In approximation, **OP-C** is therefore composed of 90% of penta-block and 10% of hexa-block chains.

Penta-block chains can either contain three blocks of PTQ10 and two blocks of PIDTe or vice-versa. Therefore, the size of each block initially determined using the D/A NMR ratio has to be divided by 2.5 for each component. For PTQ10, the size of each block in the penta-block chains is therefore 36.0/2.5=14.4 repeating units and 7.3/2.5=2.9 repeating units.

For the hexa-block chains, the number of respective blocks is 3 each case and the size of each block is 36.0/3=12 repeating units for PTQ10 and 7.3/3=2.4 repeating units for PIDTe.

In summary, the average number of repeating unit for PTQ10 is equal to 0.9*14.4+0.1*12=14.2 for PTQ10 and 0.9*2.9+0.1*2.4=2.9 for PIDTe, equivalent to approximately 6900 g/mol and 4400 g/mol for each block respectively.

Figure S126. Example of signal deconvolution of the heterojunction peaks (left), the PTQ10 4.7 ppm -CH_2_ peak (middle) and the PIDTe 2.7 ppm -CH_2_ peak.

Table S4. Summary of the peak areas determined by signal deconvolution with TopSpin.

|  | Areas | Deconvoluted area (A.U.) | Corrected area (A.U.)^a^ |
| --- | --- | --- | --- |
| OP-D | PTQ10 (4.7 ppm, A_D_) | 277.6 | 138.8 |
|  | PIDTe (2.7 ppm, A_A_) | 1032.1 | 147.4 (132.4) |
|  | Heterojunction (AJC1-like, A_**_) | 8.9 | 8.9 |
|  | Heterojunction (AJC2-like, A_*_) | 6.2 | 6.2 |
| OP-C | PTQ10 (4.7 ppm, A_D_) | 277.8 | 138.9 |
|  | PIDTe (2.7 ppm, A_A_) | 225.4 | 28.2 (12.4) |
|  | Heterojunction (AJC1-like, A_**_) | 11.2 | 11.2 |
|  | Heterojunction (AJC2-like, A_*_) | 4.6 | 4.6 |
| ^a^The corrected area is calculated by normalizing each integral according to the number of protons contributing to the signal. For the PIDTe, the integral of the bracketed value corresponds to the corrected area minus the area of the heterojunctions. | | | |

Table S5. Summary of the peak areas determined by signal deconvolution with TopSpin.

| Material | N_D_/N_A_ (integ.) | N_D_/N_A_ (decon.) | N_D_^a^ | N_A_^a^ | N_D_/N_H_ | N’_A_/N_H_ | N_**_/N_*_ |
| --- | --- | --- | --- | --- | --- | --- | --- |
| TP-D | 1/0.76 | - | 17.5 | 13.3 | - | - | - |
| OP-D | 1/1.10 | 1/1.06 | 2.5 | 2.7 | 1/0.11 | 1/0.11 | 1/0.7 |
| OP-C | 1/0.22 | 1/0.20 | 36.0 | 7.3 | 1/0.11 | 1/1.28 | 1/0.4 |
| ^a^The N_D_ and N_A_ values are given prior to recalculation with the NoB | | | | | | | |

Table S6. Summary of the composition parameters determined by signal deconvolution for PTQ10-*b*-PL8-BO and PTQ10-*b*-PITIC.

| Material | N_D_/N_A_ (decon.) | NoH | NoB | N_D_/N_H_ | N’_A_/N_H_ | N_**_/N_*_ | N_D_ | N_A_ |
| --- | --- | --- | --- | --- | --- | --- | --- | --- |
| PTQ10-*b*-PITIC | 1/0.18 | 2.1 | 3.1 | 1/0.10 | 1/1.13 | 1/1.4 | 14.2 | 2.6 |
| PTQ10-*b*-PL8-BO | 1/0.33 | 0.1 | 1.1 | 1/0.01 | 1/0.04 | 1/0.2 | 2.8 | 0.9 |

Table S7. Comparison of the composition parameters determined by signal deconvolution with TopSpin and Mestrenova for the materials studied in this work.

| Material | Method | N_D_/N_A_ (decon.) | NoH | NoB | N_D_/N_H_ | N’_A_/N_H_ | N_**_/N_*_ | N_D_ | N_A_ |
| --- | --- | --- | --- | --- | --- | --- | --- | --- | --- |
| OP-D | TopSpin | 1/1.06 | 0.3 | 1.3 | 1/0.11 | 1/0.11 | 1/0.7 | 2.5 | 2.7 |
|  | Mestrenova | 1/1.05 | 0.4 | 1.4 | 1/0.17 | 1/0.17 | 1/0.5 | 2.6 | 2.7 |
| OP-C | TopSpin | 1/0.20 | 4.1 | 5.1 | 1/0.11 | 1/1.28 | 1/0.4 | 14.2 | 2.9 |
|  | Mestrenova | 1/0.22 | 3.9 | 4.9 | 1/0.11 | 1/1.00 | 1/0.3 | 14.2 | 3.0 |
| PTQ10-*b*-PITIC | TopSpin | 1/0.18 | 2.1 | 3.1 | 1/0.10 | 1/1.13 | 1/1.4 | 14.2 | 2.6 |
|  | Mestrenova | 1/0.18 | 1.9 | 2.9 | 1/0.06 | 1/0.50 | 1/1.5 | 15.3 | 2.8 |
| PTQ10-*b*-PL8-BO | TopSpin | 1/0.33 | 0.1 | 1.1 | 1/0.01 | 1/0.04 | 1/0.2 | 7.1 | 2.3 |
|  | Mestrenova | - | - | - | - | - | - | - | - |

# Uncertainty calculations with TopSpin

As deconvolution approximates the shape of NMR signals using Lorentzian functions to a certain degree of accuracy, a statistical analysis was performed to evaluate the uncertainty in the NoH and NoB values. As the heterojunction signals * and ** have the least defined baseline, the calculations are based on the determination of their area. The equations used to calculate the uncertainties are provided below, and the results are available in Table S8 and Table S9. The uncertainty in the area of the heterojunction signals is determined based on the standard deviation of their intensity and width relative to the true shape of the NMR signals, as provided by the fitting. The most significant finding arising from the results is that fitting the NMR spectra using the deconvolution algorithm introduces minimal uncertainty in the NoH value. In fact, with the exception of **PTQ10-*b*-PL8-BO**, all uncertainties represent no more than 7% of the original value. Remarkable accuracy of 98.6% was obtained for **PTQ10-*b*-PITIC**. In contrast, an uncertainty of around 22% was found for **PTQ10-*b*-PL8-BO**, where the heterojunction signals are weaker.

This analysis shows that the NoH and NoB of donor-acceptor conjugated block copolymers can be determined with minimal uncertainty using different methods and software with comparable results. These results broaden the scope of the study and demonstrate the robustness of the methodologies presented.

*Equations used for uncertainty calculations.*

TopSpin provides uncertainty estimates for the intensity and width of the fitted signals, relative to the true shape of the NMR signals. For a pure Lorentzian, the area of a signal is proportional to its intensity multiplied by its width.

$$Area\propto Intensity* Width$$

Therefore, uncertainties on the areas can be estimated by error propagation from the fitted peak height and linewidth.

$$\left( \frac{\sigma_{Area}}{Area} \right)^{2}\approx\left( \frac{\sigma_{Intensity}}{Intensity} \right)^{2}+\left( \frac{\sigma_{Width}}{Width} \right)^{2}$$

$\sigma_{Area}$, $\sigma_{Intensity}$ and $\sigma_{Width}$ represent the standard deviation of the area, intensity and width values, respectively. $Area$, $Intensity$ and $Width$ correspond to their absolute values. This leads to:

$$\sigma_{Area}\approx Area*\sqrt{\left( \frac{\sigma_{Intensity}}{Intensity} \right)^{2}+\left( \frac{\sigma_{Width}}{Width} \right)^{2}}$$

The uncertainties of the NoH and NoB are calculated based on those of the heterojunction signal area. The uncertainty of the area of both the * and ** signals ($\sigma_{*}$ and $\sigma_{**}$) can be estimated using the previous formula. The total area uncertainty ($\sigma_{Area}^{tot}$) can be calculated using quadrature error propagation:

$$\sigma_{Area}^{tot}=\sqrt{\sigma_{*}^{2}+\sigma_{**}^{2}}$$

The uncertainties of the NoH ($\sigma_{NoH})$ and NoB ($\sigma_{NoB}$) are ultimately determined by computing their value at the extremes of the total heterojunction area.

Table S8. Measured and calculated uncertainties of the different lineshape parameters of the fitted heterojunctions signals and calculated uncertainties on the NoH and NoB.

| Material | Signal | σ_Intensity_^a^ | Intensity^a^ | σ_Width_^a^ | Width^a^ | Area^b^ | σ_Area_^b^ | σ^tot^_Area_^b^ | σ_NoH_ | σ_NoB_ |
| --- | --- | --- | --- | --- | --- | --- | --- | --- | --- | --- |
| OP-D | * | 0.003 | 0.040 | 0.989 | 7.923 | 6.162 | 0.897 | 1.172 | 0.02 | 0.02 |
|  | ** | 0.004 | 0.092 | 0.360 | 4.962 | 8.908 | 0.753 |  |  |  |
| OP-C | * | 0.001 | 0.027 | 0.947 | 8.656 | 4.575 | 0.528 | 0.766 | 0.20 | 0.20 |
|  | ** | 0.001 | 0.052 | 0.496 | 10.898 | 11.224 | 0.555 |  |  |  |
| PTQ10-*b*-PITIC | * | 0.000 | 0.021 | 0.382 | 15.802 | 6.199 | 0.150 | 0.175 | 0.03 | 0.03 |
|  | ** | 0.000 | 0.031 | 0.158 | 7.932 | 4.506 | 0.090 |  |  |  |
| PTQ10-*b*-PL8-BO | * | 0.000 | 0.001 | 7.528 | 10.000 | 0.128 | 0.096 | 0.119 | 0.02 | 0.02 |
|  | ** | 0.000 | 0.004 | 1.235 | 10.999 | 0.619 | 0.070 |  |  |  |
| ^a^Values are given in Hz ^b^Values are given in A.U. | | | | | | | | | | |

Table S9. Uncertainty of the NoH and NoB values determined by the statistical analysis of the deconvolution results.

| Material | TopSpin | | Mestrenova | |
| --- | --- | --- | --- | --- |
|  | NoH | NoB | NoH | NoB |
| OP-D | 0.28±0.02 | 1.28±0.02 | 0.4 | 1.4 |
| OP-C | 4.10±0.20 | 5.10±0.20 | 3.9 | 4.9 |
| PTQ10-*b*-PITIC | 2.12±0.03 | 3.12±0.03 | 1.9 | 2.9 |
| PTQ10-*b*-PL8-BO | 0.09±0.02 | 1.09±0.03 | - | - |

# References

[1] J. J. Rech, J. Neu, Y. Qin, S. Samson, J. Shanahan, R. F. Josey III, H. Ade, W. You, *ChemSusChem* **2021**, *14*, 3561.

[2] H. Fu, Y. Li, J. Yu, Z. Wu, Q. Fan, F. Lin, H. Y. Woo, F. Gao, Z. Zhu, A. K.-Y. Jen, *J. Am. Chem. Soc.* **2021**, *143*, 2665.

[3] Y. Lin, J. Wang, Z.-G. Zhang, H. Bai, Y. Li, D. Zhu, X. Zhan, *Adv. Mater.* **2015**, *27*, 1170.

[4] Prediction of 19F NMR Chemical Shifts for Fluorinated Aromatic Compounds, .

[5] K. Chen, *International Journal of Molecular Sciences* **2020**, *21*, 5666.

[6] M. H. Levitt, *Spin Dynamics: Basics of Nuclear Magnetic Resonance*, John Wiley & Sons **2008**.

[7] L. Petrakis, *J. Chem. Educ.* **1967**, *44*, 432.
